# Proton Solvation in Water and Selected Organic Solvents: A Critical Assessment of Experimental and Theoretical Approaches to Extend the Solvent‐Independent Unified Acidity Scale

**DOI:** 10.1002/cphc.202500349

**Published:** 2026-04-25

**Authors:** Regina Stroh, Niklas Gebel, Timo Kienzle, Valentin Radtke, Ingo Krossing

**Affiliations:** ^1^ Institut für Anorganische und Analytische Chemie and Freiburger Materialforschungszentrum (FMF) Universität Freiburg Freiburg Germany

**Keywords:** cluster continuum model, “Ideal” ionic liquid salt bridge, proton solvation, single‐ion transfer, unified acidity scale

## Abstract

The proton's Gibbs solvation energy in various organic solvents was determined by experimental and theoretical means to serve as anchor points for the solvent‐independent Unified Acidity Scale (UAS) and the Protoelectric Potential Map (PPM). Experimentally, potential differences between two half‐cells connected by an “ideal” ionic liquid salt bridge (ILSB) were measured. Here, our established setup with the “ideal” Ionic Liquid [N_2225_][NTf_2_] in the ILSB ([N_2225_] = [N(C_2_H_5_)_3_(C_5_H_11_)]; Tf = SO_2_CF_3_) was refined to enable measurements of the Gibbs energies of proton transfer Δ_tr_ *G*°(H^+^, S_1_→ S_2_) between different solvents S_1_ and S_2_, such as water, methanol, ethanol, acetonitrile and methyl formate. These results were further evaluated by extensive theoretical studies using the double‐hybrid functional DSD‐BLYP for structure optimization and highly accurate coupled cluster DLPNO‐CCSD(T) calculations that were extrapolated to the basis set limit (CBS) for the gas phase contributions. Solvation effects were added implicitly with the Conductor‐like Polarizable continuum Model (CPCM) in a Cluster Continuum approach, including a scheme to use the CPCM with coupled cluster calculations in ORCA's newest version. A monomer and a cluster thermodynamic cycle were used to calculate the proton's solvation energy from the calculated solvent clusters. The cluster cycle performed well with consistent improvement at higher levels of theory, while the monomer cycle suffered from inadequate error compensation. Yet, the monomer cycle results can be significantly improved by the inclusion of experimental data, such as Gibbs energies of evaporation. However, it remains clear that even when using the most sophisticated of all currently available methods, the error bars of the calculations may at best reach about 10–15 kJ mol^–1^ and, given low polarity solvents like methyl formate with *ε*_r_ = 8.838, may even substantially exceed this error bar. Analysis shows that one of the largest problems arising for the calculations is the lack of reliable experimental structural data in solution to know how the dissolved particles governing the protochemical potential exactly prevail in solution. The delicate balance of subtle and relatively weak hydrogen bonds or dispersive interactions may lead to large structural differences between the structures known in the solid state (e.g., from single crystal structure determinations) and those actually being thermodynamically relevant in (dynamic) equilibrium in solution. We present some problems encountered during our work on this subject. At least for the more polar “classical” solvents, experimental ILSB‐ as well as computationally obtained results are in good agreement with the known values from the TATB assumption, which is currently one of the most widely used assumptions for determining single‐ion transfer energies. However, with none of the described in part rather sophisticated calculations, it is possible to achieve “chemical accuracy” in solution, as one can with the used “gold standard” coupled cluster methods in the gas phase (i.e., ±4 kJ mol^–1^).

## Graphical abstract

The (solvated) proton is the most elusive chemical entity, both by experimental and by theoretical means. Visit this special exhibition, explore the proton´s structure and chemical potential in water and various organic solvents. Let yourself be fascinated by its many facets and be there when it is unified.

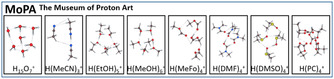

**Abbreviations**
[C_8_MIm][*FAP*]: (1‐methyl‐3‐octylimidazolium tris(pentafluoroethyl)trifluorophosphate)
[N_2225_][NTf_2_]: Triethylpentylammonium bis(trifluoromethanesulfonyl)imide
[NEt_4_][Pic]: Tetraethylammonium picrate
B3LYP: Hybrid functional with 20% HF exchange
BLYP: GGA functional with Becke 88 exchange and Lee‐Yang‐Parr correlation
BP/BP86: GGA functional with Becke 88 exchange and Perdew 86 correlation
CBS: Basis set limit
CC: Coupled cluster
cc‐pVQZ: Correlation‐consistent polarized quadruple‐zeta basis set
CCSD(T): Coupled cluster with single, double and a numerical evaluation of the triple excitation
CPCM: Conductor‐like Polarizable continuum Model
D3BJ: Atom‐pairwise dispersion correction with Becke‐Johnson damping
def2‐QZVPP: Quadruple‐ζ Ahlrichs basis set, double polarization functions
def2‐TZVPP: Triple‐ζ Ahlrichs basis set, double polarization functions
DFT: Density functional theory
DH: Double‐hybrid functional
DMF: N,N‐Dimethylformamide
DMSO: Dimethyl sulfoxide
DSD‐BLYP: Martin's general purpose double‐hybrid functional, including B88 exchange, LYP correlation and SCS‐MP2 mixing
DSD‐PBE86: Martin's general purpose double‐hybrid functional, including PBE exchange, P86 correlation and SCS‐MP2 mixing
EtOH: Ethanol
GB: Gas‐phase basicity
GGA: Generalized gradient approximations
IL: Ionic liquid
ILSB: Ionic liquid salt bridge
LJP: Liquid junction potential
M06: Minnesota‐Hybrid functional 06 with 27% HF exchange
MAE: Mean absolute error
MeCN: Acetonitrile
MeFo: Methyl formate
MeOH: Methanol
MP2: Second order Møller‐Plesset perturbation theory
PC: Propylene carbonate
PES: Potential energy surface
PPM: Protoelectric potential map
SCF: Self‐consistent field
SSE: Silver/Silver sulfate electrode
TATB: Tetraphenylarsonium tetraphenylborate
TPSS0: Hybrid version of the TPSS functional, which includes 25% HF exchange
TPSSh: Hybrid version of the TPSS functional, which includes 10% HF exchange
UAS: Unified acidity scale
ωB97X‐D3BJ: Modified version of the range‐separated hybrid functional ωB97X‐V, including the D3BJ correction by Najibi and Goerigk

## 1 Introduction

In 2010, our group introduced the solvent‐independent Unified Acidity Scale pH_abs_ (UAS, at the end of the article, we provide a list of abbreviations), which allows acidity to be compared across different solvents, media, and states [1, 2]. The Protoelectric Potential Map (PPM) [1, 2, 3] merges this scale with the unified reducity scale pe_abs_ into a single two‐dimensional representation—a unified, solvent‐independent water window—that transforms the reducity and acidity of a system into a visual plot that allows to quickly rationalize, for example, the pH dependence of redox reactions and the solvent's stability window in different media on one view. However, the PPM relies on single‐ion quantities, such as single‐ion transfer and single‐ion solvation, which are challenging to determine both experimentally and theoretically. Unfortunately, single‐ion properties are not directly accessible through experimental procedures due to the requirement of overall charge neutrality of the macroscopic system, which prevents individual ions from existing independently [4].

### 1.1 Measuring Single‐Ion Magnitudes

The only way to measure single‐ion quantities involves making extra‐thermodynamic assumptions, some of which enable the separation of the contribution of a salt into equal values for each ion. These assumptions are, while reasonable, not actually provable [5]. The TATB (tetraphenylarsonium tetraphenylborate, [AsPh_4_]^+^[BPh_4_]^–^) assumption is the most notable representation of this premise, claiming that the solvation and transfer energies of both [TA]^+^ and [TB]^−^ are equivalent, regardless of their opposite charge, i.e. Δ_tr_ *G*°([TA]^+^) = Δ_tr_ *G*°([TB]^−^) for all solvents. Thus, the Gibbs energy of transfer of an individual ion i Δ_tr_ *G*°(i, S_1_ → S_2_) can be determined by measuring the transfer of the respective [TA]^+^ or [TB]^−^ salts. Despite being regarded as the most accurate approximation, the TATB assumption has faced criticism and its error estimates range from 3 to 6 kJ mol^−1^ to 115 kJ mol^−1^ [6, 7, 8, 9, 10, 11, 12]. Many other assumptions to obtain the single‐ion transfer Δ_tr_ *G*°(i, S_1_→ S_2_) have emerged over the years, all with a varying, but ultimately unknown extent of uncertainty [5].

### 1.2 The Ionic Liquid Salt Bridge (ILSB)

Another extra‐thermodynamic assumption targets the salt bridge used in the electrochemical determination of the Gibbs energy of a single‐ion transfer Δ_tr_ *G*°. The intricacy with these measurements arises from the liquid junction potential (LJP), *E*_j_, that emerges at the two liquid–liquid interfaces in a setup of two electrochemical half‐cells connected by a salt bridge. This affects the measured potential by an unknown amount. The LJP is a steady state that can be quantified based on Onsager's reciprocal relations [13]. However, the resulting formula contains Gibbs transfer energies of single ions: a vicious circle that cannot be resolved within the thermodynamic formalism. Parker et al. were the first to make significant progress with their negligible LJP assumption and the tetraethylammonium picrate ([Et_4_N][Pic]) salt, see for example Ref [14]. Later, Kakiuchi and his team [15, 16, 17, 18, 19, 20, 21] were able to reduce the LJP to a negligible amount in aqueous systems with an ionic liquid salt bridge (ILSB). Although the LJP still prevails in this setup, it is almost equal on both sides of the salt bridge, resulting in a cancelation.

Building on Kakiuchi's work, the Krossing group developed the “ideal” ILSB approach that also applies to various organic solvents using the [N_2225_][NTf_2_] (amyltriethylammonium bis(trifluoromethanesulfonyl)imide) as an “ideal” ionic liquid [22]. An ionic liquid is considered “ideal” when the diffusion coefficients of both cation and anion are virtually equal in both solvents and the ionic liquid itself. Hence, their contribution to the diffusion potential at the two liquid junctions is minimal. In addition, the investigation of the Ag^+^ ion [23, 24] and the Cl^−^ ion [25] proved that the sum of the LJPs, *E*_j, 1_ + *E*_j, 2_, is consistently constant in the different implementations of cells I (Ag^+^) and II (Cl^−^). Thus, using the “ideal” ILSB approach, thermodynamic magnitudes, that is, the Gibbs transfer energy of the electroneutral salt AgCl, may be assessed from the measured single ion magnitudes between six different solvents with twelve half‐cells and within an error bar of less than 1 kJ mol^–1^ [25]. This is a consequence of the large excess of IL ions, so that neither the ions (providing an adequate concentration *c*) nor the solvents in the half‐cells have a significant influence on the LJPs.
(cell I)

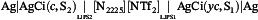






(cell II)

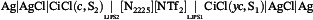




Ci is an appropriate counterion, which can be the same or different in the half‐cells. S_1_ and S_2_ are different solvents from the series of solvents investigated experimentally: in this work, water, methanol, ethanol, acetonitrile, and methyl formate. If the transference numbers *t*_+_ and *t*_−_ of the [N_2225_]^+^ and the [NTf_2_]^−^ ions are equal or almost equal (which is the case) [23, 24, 25], the sum of both LJPs can be approximated by (Equation 1) [24].

(1) $$\left(E_{j,1}+E_{j,2}\right)\cdot F=\frac{1}{2}{\text{Δ}}_{\mathrm{tr}}G{}^{\circ}\left({\left[N_{2225}\right]}^{+},S_{1}\to \;S_{2}\right)-\frac{1}{2}{\text{Δ}}_{\mathrm{tr}}G{}^{\circ}\left({\left[{\mathrm{NTf}}_{2}\right]}^{-},S_{1}\to \;S_{2}\right)$$

All hitherto performed measurements suggest that the individual LJPs in cells I and II add up to almost zero, meaning that they are negligible. In this case, the ILSB assumption emerges as assumption, that the Gibbs transfer energies of the cation and the anion of the IL are equal, Δ_tr_ *G*°([N_2225_]^+^) = Δ_tr_ *G*°([NTf_2_]^−^), and is therefore similar to the TATB assumption. The Gibbs transfer values obtained with the ILSB approach reproduce the values obtained with the TATB assumption (within the stated uncertainty of 6 kJ mol^−1^). Further, they even reflect the asymmetric solvation of the [TA]^+^ cation and the [TB]^−^ anion in water, which was determined by Raman measurements to amount to about 5 kJ mol^−1^ [12]. Since water forms fewer and weaker π‐H bonds to [TA]^+^ than to [TB]^−^, Δ_tr_ *G*°(i, H_2_O → S) values determined with the TATB assumption for single ion transfer from water to organic solvents appear by about 2.5 kJ mol^−1^ too positive for cations and by about 2.5 kJ mol^−1^ too negative for anions. A detailed analysis showed that the TATB Gibbs transfer energies of the Ag^+^ ion from water to organic solvents deviate by + 2.3 kJ mol^−1^ and of the Cl^−^ ion by −2.7 kJ mol^−1^ from the ILSB Gibbs transfer energies. Concerning the Gibbs transfer energies between organic solvents, these deviations are −0.1 kJ mol^−1^ and + 0.6 kJ mol^−1^, respectively. According to Occam's razor, the simplest explanation for the aforementioned results is that both the TATB assumption (for S = water within 5 kJ mol^−1^) and the ILSB assumption are applicable [26]. Irrespective of this, the constancy of the LJP sum allows other redox pairs to be measured in order to obtain a consistent data set.

### 1.3 Proton Transfer With the “Ideal” ILSB

This work introduces the ILSB method to determine Gibbs transfer energies of the proton between aqueous and several organic solvents with cell III,



(cell III)

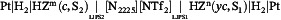




and using the acids HZ^m^ and HZ^n^, which have the anions [Z^m^]^−^ and [Z^n^]^−^, respectively. If the sum of both LJPs of cell III approximates zero, the Gibbs transfer energy of the proton Δ_tr_ *G*°(H^+^, S_1_ → S_2_) would result from the cell potential *E*_III_:

(2) $${\text{Δ}}_{\mathrm{tr}}G{}^{\circ}{\left(H^{+},S_{1}\to \;S_{2}\right)}_{\mathrm{III}}=-nFE_{\mathrm{III}}$$

The electron number of the cell reaction is denoted by *n* and *F* represents the Faraday constant. If the solvation energy of the single‐ion in one of the examined solvents is known, it is possible to gain the solvation energy in any other solvent from the transfer energy Δ_tr_ *G*°(H^+^, S_1_ → S_2_):

(3) $${\text{Δ}}_{\text{solv}}G{}^{\circ}\left(H^{+},S_{2}\right)={\text{Δ}}_{\text{solv}}G{}^{\circ}\left(H^{+},S_{1}\right)+{\text{Δ}}_{\mathrm{tr}}G{}^{\circ}\left(H^{+},S_{1}\to \;S_{2}\right)$$

Within the framework of the UAS, the solvation energy of the proton Δ_solv_ *G*°(H^+^, S) represents its chemical potential *µ*_abs_°(H^+^, S):

(4) $${\text{Δ}}_{\text{solv}}G{}^{\circ}\left(H^{+},S\right)=\mu_{\mathrm{abs}}^{{}^{\circ}}\left(H^{+},S\right)$$

The ideal proton gas at 1 bar and 298.15 K is the reference state of the UAS, and its chemical potential is set to zero. Thus, the pH_abs_ value can be obtained as:

(5) $${\mathrm{pH}}_{\mathrm{abs}}=-\frac{\mu_{\mathrm{abs}}\left(H^{+},S\right)}{RT\ln\;10}$$

To simplify the understanding of this scale, its zero point was aligned with the pH scale of water and labeled the ${\mathrm{pH}}_{\mathrm{abs}}^{H_{2}O}$ scale:

(6) $${\mathrm{pH}}_{\mathrm{abs}}^{H_{2}O}={\mathrm{pH}}_{\mathrm{abs}}-193.5={\mathrm{pH}}_{\mathrm{abs}}+\frac{\mu_{\mathrm{abs}}^{{}^{\circ}}\left(H^{+},H_{2}O\right)}{RT\ln\;10}\;\;\;\;\text{at 298.15 K}$$

The ${\mathrm{pH}}_{\mathrm{abs}}^{H_{2}O}$ scale depends on the proton's Gibbs hydration energy. The widely accepted value of −1104.5 kJ mol^−1^ was determined by Tissandier et al. through the cluster pair approximation [27] with an estimated uncertainty of ±8 kJ mol^−1^ [28], and is recommended by Camaioni et al. [29]. Hence, the ${\mathrm{pH}}_{\mathrm{abs}}^{H_{2}O}$ scale is solvent independent, and is thecontinuation of the conventional pH values in water ${\text{pH}}_{H_{\text{2}}O}$, thus, allows for putting any acid in any solvent S on to the same scale.

## 2 Quantum‐Chemical Calculations of Solvation Energies: The Cluster Continuum Model

In contrast to electrochemical measurements, computational chemistry is not bound by electroneutrality, enabling direct calculation of the solvation energies of individual ions. The primary problem lies in the insufficient theoretical description of the solvent and solvation effects. Continuum solvation models are the most efficient way to conduct these calculations. They describe the solvent only implicitly as a continuous electric field with its strength corresponding to the static permittivity *ε*_r_ of the solvent. A molecule‐shaped cavity within the continuum contains the solute with its charge distribution mapped onto the surface of the cavity. This charge distribution interacts with the electric field and results in the electrostatic contribution to the solvation energy, with additional terms for cavitation and dispersion. This simplified representation of the solvent can lead to high inaccuracies for ionic solutes, especially single ions, as they form strong interactions with the solvent in their first coordination sphere, and the continuum does not include molecular properties. One way to avoid this is to explicitly add a finite number of solvent molecules to the solute to form a cluster and place this cluster inside a continuum, an approach commonly termed cluster continuum model [2, 30, 31, 32, 33, 34, 35, 36, 37].

### 2.1 Thermodynamic Monomer and Cluster Cycles

**FIGURE 1:**
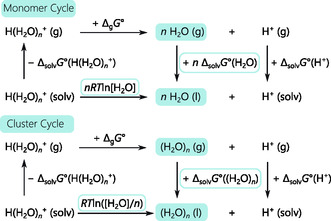
Thermodynamic cycles to calculate the Gibbs energy of solvation of the proton through a cluster continuum approach. Monomer cycle (top) using the solvation energy of a single solvent molecule and cluster (bottom) cycle using a solvent cluster.

Recovering the solvation energy of the proton from these clusters requires thermodynamic cycles. As illustrated in Figure 1 for water, Bryantsev et al. categorized these cycles into monomer (top) and cluster (bottom) types [38].

Careful considerations of the standard states that are referenced by all calculated energies are needed when working with these cycles. The Gibbs energy of solvation Δ_solv_ *G*°, that is determined experimentally and marked by the superscript circle, represents the transition of 1 bar of an ideal gas into an ideal solution of 1 mol L^−1^. While the computed gas phase energy is already at a state of 1.013 bar (= 1 atm) gas, the calculated solvation energy Δ_solv_ G* references 1 mol L^−1^ for both gas phase and solution. A superscript asterisk denotes this reference state. The change in free energy of an ideal gas transitioning from 1.013 bar (24.79 mol L^−1^)^1^ to 1 mol L^−1^ at a temperature of 298.15 K is:

(7) $$\text{Δ}G^{*\;\to \;{}^{\circ}}=RT\ln\;\left(\frac{V^{*}}{V_{0}}\right)=RT\ln\;\left(24.79\right)=7.96\;{\text{kJ mol}}^{-1}$$

All calculated solvation energies require this correction to adjust the reference state [38, 39]:

(8) $${\text{Δ}}_{\text{solv}}G{}^{\circ}={\text{Δ}}_{\text{solv}}G^{*}+7.96\;{\text{kJ mol}}^{-1}$$

However, the solvation energy of the pure solvent requires an additional adjustment. The energy of the cluster formation in the lower part of both thermodynamic cycles is zero for a reaction that occurs in the pure solvent, such as pure water with a concentration of 55.34 mol L^−1^. However, the calculated energy of the solvent refers to a solvated state of 1 mol L^−1^, so a correction needs to be added to the lower part of the monomer cycle

(9) $$\text{Δ}G{}^{\circ}=nRT\ln\;\left[H_{2}O\right]$$

Analogously, the correction to every solvent cluster (H_2_O)_*n*_ in the cluster cycle is:

(10) $$\Delta G{}^{\circ}=RT\ln([H_{2}O]/n)$$

The solvation energy of the proton in water from either the monomer or the cluster cycle can be calculated using (Equation 11) and (Equation 12), respectively.

(11) $${\text{Δ}}_{\text{solv}}G{}^{\circ}\left(H^{+}\right)={\text{Δ}}_{g}G{}^{\circ}+{\text{Δ}}_{\text{solv}}G{}^{\circ}\left(H{\left(H_{2}O\right)}^{+}\right)-n{\text{Δ}}_{\text{solv}}G{}^{\circ}\left(H_{2}O\right)-nRT\ln\;\left[H_{2}O\right]$$

(12) $$\Delta_{\mathrm{solv}}G{}^{\circ}(H^{+})=\Delta_{g}G{}^{\circ}+\Delta_{\mathrm{solv}}G{}^{\circ}\left(H{\left(H_{2}O\right)}^{+}\right)-\Delta_{\mathrm{solv}}G{}^{\circ}({\left(H_{2}O\right)}_{n})-nRT\ln(\frac{\left[H_{2}O\right]}{n})$$

This is outlined for water as an example, but has been applied analogously to all investigated solvents S. For single ions, the error in the solvation energy decreases with an increasing number of solvent molecules until a *n* is reached, where the energy no longer improves. This number usually varies depending on the system at hand and the exact theoretical method applied. Note that in the monomer cycle, the error of the protonated solvent clusters improves with *n*, while it remains unchanged for the solvation energy of the solvent itself. In contrast, the cluster cycle has a decreasing error in both the protonated solvent clusters and the solvent clusters, leading to an overall better error reduction.

With this work, we use the “ideal” ILSB method to determine Gibbs transfer energies of the proton Δ_tr_ *G*°(H^+^, S_1_ → S_2_) between different organic solvents and water. In addition, we evaluate our results with high‐level quantum chemical computations for these systems and critically assess limitations.

## 3 Results and Discussion

This article is divided into two parts: Section 3.1 and Section 3.3. Section 3.1 describes the experimental methodology and the measurements leading to the determination of the Gibbs transfer energies of the proton Δ_tr_ *G*°(H^+^, S_1_ → S_2_). In Section 3.2, the Gibbs solvation energies of the proton Δ_solv_ *G*°(H^+^, g → S) are derived after some discussion. Section 3.3 describes the computational efforts to reproduce the determined magnitudes. In Section 3.4, a detailed benchmarking and troubleshooting investigation for the intended development of the gas phase thermodynamics as a function of the quality of the structure optimization, using first water and then also methanol as prototype solvents, was performed. With the selected methods, the performance of the solvation calculations was further investigated, delivering in Section 3.8, a set of selected methods applied in Section 3.9 to calculate and discuss the relevant Gibbs energies for all solvents of this study, also with respect to experimental or other computational approaches.

### 3.1 Experimental Gibbs Energies of Transfer With the ILSB

We assembled eight different implementations of Cell III utilizing five half‐cells containing the solvents water (H_2_O), acetonitrile (MeCN), ethanol (EtOH), methanol (MeOH), and methyl formate (MeFo). HCl or HNTf_2_ (Tf = SO_2_CF_3_) was used in the aqueous half‐cell, and exclusively HNTf_2_ in the organic solvent half‐cells; the concentration *c* was 0.001 mol L^−1^. Because of the unknown proton radii in the respective solvents the activity coefficients were not estimated; however, for the Ag^+^ and Cl^−^ ions investigated earlier, only differences < 2 mV were found between concentration‐ and activity‐based values [24, 25].

**FIGURE 2:**
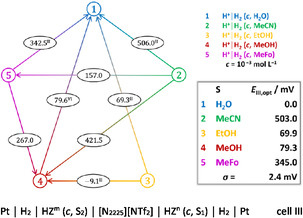
Visual representation of the network of half‐cells, that is the redox system H^+^(solv)/H_2_ within the solvents indicated. The measured cell potential values *E*_III_ are given in the circles superimposed on the arrows, it is the mean of measurements as given by the superscript Roman number. The direction of an arrow indicates the reaction of cell III, H^+^(solv, S_1_) → H^+^(solv, S_2_), thus the transfer of H^+^ from the right half‐cell to the left half‐cell. Within the box the optimized half‐cell potential values *E*_III, opt_ are listed as the result of the least‐square method; they are given with respect to half‐cell 1, i.e., as if water is consistently the solvent of the left half‐cell.

A network with the half‐cells as nodes and the measured values between them as connecting lines can be generated, see Figure 2. The mathematical equivalent of this network is an overdetermined system of linear equations with the half‐cell potentials as unknowns. It can be solved with the method of least squares, leading to optimal approximations of the unknowns together with an individual residual for each equation and a measure *σ* reflecting the precision and consistency of the measured values [24, 25]. Table 1 summarizes the experimental findings in terms of Gibbs transfer energies of the proton between said solvents. With *σ* = 2.4 mV, the precision is good. The measured data are included with the Supporting Information (Section 1.2).

**TABLE 1:** Measured, *E*_III_, and optimized, *E*_III, opt_, potential differences of cell III.

S_1_	→	S_2_	−*E*_III_ / mV	−*E*_III, opt_ / mV	Δ_tr_ *G*°(H^+^, S_1_→ S_2_)_III_ / kJ mol^−1^	Δ_tr_ *G*°(H^+^, S_1_→ S_2_)_TATB_ / kJ mol^−1^ a
H_2_O	→	MeCN	−506.0	−503.0	48.5	46.4
H_2_O	→	EtOH	−69.3	−69.9	6.7	11.1
H_2_O	→	MeOH	−79.5	−79.3	7.7	10.4
H_2_O	→	MeFo	−342.5	−345.0	33.3	n.a.
MeOH	→	MeCN	−421.5	−423.8	40.9	(36.0)
MeOH	→	EtOH	9.1	9.4	−0.9	(0.7)
MeOH	→	MeFo	−267.0	−265.8	25.6	n.a.
MeFo	→	MeCN	−157.0	−158.0	15.2	n.a.
*σ*			—	2.4 mV	0.2 kJ mol^−1^	n.a.
max. residual			—	3.0 mV	0.3 kJ mol^−1^	

a Ref [40], (values in parenthesis were calculated from the first three entries).

*Note:* Optimized cell potentials were obtained by analysis of the network shown in Figure 2. Gibbs transfer energies of the proton Δ_tr_ *G*°(H^+^)_III_ obtained with the optimized values were compared with the values obtained with the TATB assumption Δ_tr_ *G*°(H^+^)_TATB_ in the mol L^−1^ scale.

Despite the limited data, we again observe that the transfer of a positively charged ion from water to an organic solvent appears more positive with the TATB method than with the ILSB method. An exception is the transfer into MeCN. However, the corresponding TATB value was only determined indirectly, i.e., from p*K*_a_ values and solubilities of picric acid as well as the Δ_tr_ *G*°([Pic]^−^, H_2_O → MeCN)_TATB_ value of the picrate ion [Pic]^−^ [41]. We therefore consider the TATB Gibbs transfer energy values of the proton to be less accurate than those for silver and chloride ion transfers, which are determined by solubility measurements of Ag[TB] and [TA]Cl, respectively. Eventually, Marcus et al. state 6 kJ mol^−1^ as the uncertainty of the TATB assumption [42], so it cannot be expected per se that the asymmetric hydration of TATB discussed above can be detected.

### 3.2 Independent Evaluation and Assessment of Gibbs Solvation Energies of the Proton Δ_solv_ *G*°(H^+^, S)

Another assessment of the obtained values is the comparison of *E*_S_° values, which are the redox potentials of the Ag^+^/Ag and the Cl_2_/Cl^–^ redox systems in the potential scale of the solvent S. On the one hand, these values can be measured directly, e.g., with cell IV for the Ag^+^/Ag system.

(cell IV) $$\mathrm{Pt}|H_{2}|{\mathrm{HZ}}^{m}\left(c,S\right)|\left[N_{2225}\right]\left[{\mathrm{NTf}}_{2}\right]|{\mathrm{AgZ}}^{n}\left(c,S\right)|\mathrm{Ag}$$

In cell IV, HNTf_2_ was used as strong acid with an experimental p*K*_a_‐value of 0.3 in MeCN [37], and we approximate the activity coefficient of the proton as unity since the acid concentration is rather small (*c* = 0.001 mol L^−1^). In the right half‐cell of cell IV, the activity coefficient of the Ag^+^ ion is approximated as unity, likewise, and we expect that the uncertainties resulting from this for both half‐cells will be largely compensated for in cell IV. Alternatively, the *E*_S_° values can be calculated from the Gibbs transfer energy values according to (Equation 13) (Ag^+^/Ag) and (Equation 14) (Cl_2_/Cl^−^).

(13) $$E_{S}^{{}^{\circ}}\left({\mathrm{Ag}}^{+}/\mathrm{Ag}\right)=E_{H_{2}O}^{{}^{\circ}}\left({\mathrm{Ag}}^{+}/\mathrm{Ag}\right)+\frac{{\text{Δ}}_{\mathrm{tr}}G^{{}^{\circ}}\left({\mathrm{Ag}}^{+},H_{2}O\;\to \;S\right)-{\text{Δ}}_{\mathrm{tr}}G^{{}^{\circ}}\left(H^{+},H_{2}O\;\to \;S\right)}{F}$$

(14) $$E_{S}^{{}^{\circ}}\left({\mathrm{Cl}}_{2}/{\mathrm{Cl}}^{-}\right)=E_{H_{2}O}^{{}^{\circ}}\left({\mathrm{Cl}}_{2}/{\mathrm{Cl}}^{-}\right)-\frac{{\text{Δ}}_{\mathrm{tr}}G^{{}^{\circ}}\left({\mathrm{Cl}}^{-},H_{2}O\;\to \;S\right)+{\text{Δ}}_{\mathrm{tr}}G^{{}^{\circ}}\left(H^{+},H_{2}O\;\to \;S\right)}{F}$$

Table 2 summarizes the results. It becomes clear that the Gibbs transfer energies obtained with the ILSB method reflect the directly measured *E*_S_° values more accurately than the Gibbs transfer values obtained with the TATB method.

**TABLE 2:** Values *E*_S_° of the redox systems Ag^+^/Ag and Cl_2_/Cl^−^ as obtained from Gibbs transfer energies of the appropriate ions according to (Equations 13) and (14) (with $E_{H_{2}O}^{{}^{\circ}}\left({\mathrm{Ag}}^{+}/\mathrm{Ag}\right)$ and $E_{H_{2}O}^{{}^{\circ}}\left({\mathrm{Cl}}_{2}/{\mathrm{Cl}}^{-}\right)$ taken from literature [43]), and from direct measurements, e.g., cell IV.

S	$E_{S}^{{}^{\circ}}\left({\mathrm{Ag}}^{+}/\mathrm{Ag}\right)$ / V		$E_{S}^{{}^{\circ}}\left({\mathrm{Cl}}_{2}/{\mathrm{Cl}}^{-}\right)$ / V	
	Eq 13	Cell IV	Eq 14	
MeCN	0.041_ILSB_ a ^,^ b (0.078_TATB_)^c^	0.034^b^ (0.100 or 0.046)^d^	0.403_ILSB_ a ^,^ b (0.443_TATB_)^c^	—
EtOH	0.749_ILSB_ a ^,^ b (0.735_TATB_)^c^	0.745^b^ (0.749)^e^	1.056_ILSB_ a ^,^ b (1.013_TATB_)^c^	(1.060)^e^
MeOH	0.762_ILSB_ a ^,^ b (0.760_TATB_)^c^	0.758^b^ (0.764)^f^	1.119_ILSB_ a ^,^ b (1.093_TATB_)^c^	(1.116)^g^

a ILSB‐Gibbs transfer energy values from Ref [25].

b This work.

c TATB‐Gibbs transfer energy values from Ref [40].

d Value from Ref [44]; note that we hardly can estimate the uncertainty of this value but it can be re‐evaluated as 0.046 V, see the Supporting Information, Section 1.4.

e Value from Ref [45].

f Value from Ref [46].

g Value from Ref [47].

*Note* that *E*_S_°(H^+^/H_2_) by definition is zero for all S, as it is the standard hydrogen electrode SHE_S_ in the solvents S. All values are given in V. Values enclosed in parenthesis are obtained with literature data as indicated.

The discrepancy of the value of cell IV implemented with S = MeCN and the value published by Kolthoff et al. is discussed in detail in the Supporting Information, Section 1.4. At this point, it should be noted that this potential value can also be determined in an alternative approach from the original data as described in the Supporting Information, leading to *E*_MeCN_°(Ag^+^/Ag) = 0.046 V, which is significantly closer to our measured value with Cell IV.

We emphasize that this assessment is aimed at the precision of the ILSB method and not its accuracy. The TATB method also provides, within its stated accuracy of about 6 kJ mol^−1^ or 60 mV, a value that is in agreement with the values obtained with cell IV. Again, we apply Occam's razor to explain the correspondence found between the two methods: The simplest explanation is that both assumptions are justified [26] (in case of Ag^+^ and Cl^−^ within about 0.6 kJ mol^−1^, and in case of H^+^ within about 5 kJ mol^−1^, however, as discussed above, we consider the proton´s TATB values as less accurate). The next, less simple explanation is that both assumptions are incorrect, and in addition, both errors are identical [26]. We consider this case to be less likely than the first case. Nevertheless, we are currently investigating both possibilities in more detail.

The accuracy given in the literature for the asymmetric hydration of [TA]^+^ and [TB]^−^ is approximately ±0.35 kJ mol^−1^ [12]. This corresponds roughly to the precision of ±0.25 kJ mol^−1^ found for the ILSB method. Thus, we estimate the accuracy for the ILSB method somewhat higher than its precision to ±0.6 kJ mol^−1^, but we note that this is subject to continuous work and adjustment.

### 3.3 Gibbs Energies of Solvation Computed Within the Cluster Continuum Model

The solvation energies Δ_solv_ *G*°(H^+^, S) of all species were computed using the CPCM solvation model within the cluster continuum approach, incorporating the thermodynamic cycles of Figure 1 to determine the solvation energy of the proton. All calculations and all constants used here refer to a temperature of 298.15 K and a pressure of 1 bar. The Supporting Information provides the exact computational details and the references used for this section. In addition to the here measured solvents water (H_2_O; *ε*_r_ = 78.355 [48]), acetonitrile (MeCN; *ε*_r_ = 35.688 [48]), ethanol (EtOH; *ε*_r_ = 24.852 [48]), methanol (MeOH; *ε*_r_ = 32.613 [48]), and methyl formate (MeFo; *ε*_r_ = 8.838 [48]), the Gibbs solvation energies for the aprotic solvents N, N‐dimethylformamide (DMF; *ε*_r_ = 37.219 [48]), Dimethyl sulfoxide (DMSO; *ε*_r_ = 46.826 [48]), and Propylene carbonate (PC; *ε*_r_ = 64.92 [49]) were computed. Table S5 in the Supporting Information includes all solvent descriptors and variables required for the calculations.

#### 3.3.1 Schematic Workflow

**FIGURE 3:**
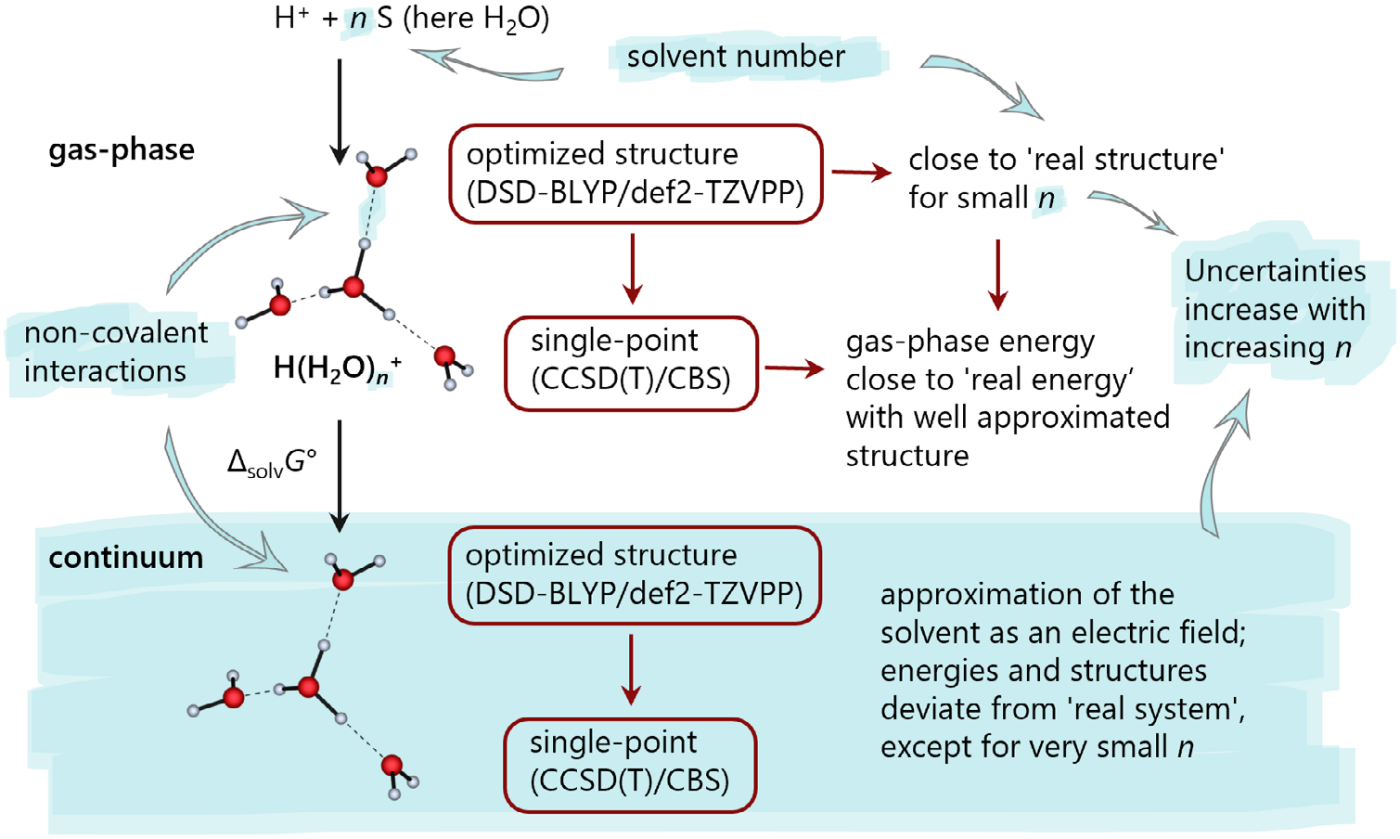
Simplified schematic outline of the workflow and the main issues related to the cluster continuum model. Protonated solvent clusters of various sizes were modeled and optimized using, among other methods, DSD‐BLYP/def2‐TZVPP, both in the gas‐phase and in the continuum. Single‐point DLPNO‐CCSD(T)/CBS calculations were then performed on these optimized structures, again in the gas‐phase and in the continuum. Some of the key issues associated with the model are highlighted in blue, including continuum calculations in general, the solvent number *n* and the description of the non‐covalent interactions, as well as the deviation of the gas‐phase and continuum structures from the real structure, which is quasi unknown for the solvated state.

In the following, we will evaluate the performance of the thermodynamic monomer and cluster cycles in Figure 1 with respect to the level of theory and focus on common issues surrounding the cluster continuum model, such as the number of explicit solvent molecules, the structure of protonated and non‐protonated solvent clusters, and the effect of isomers. Our approach involves the common strategy to optimize structures using a lower‐level method, followed by a single‐point calculation with an advanced method to obtain a better description of the electronic energy. Three methods were chosen for these calculations because they performed best in the test calculations presented in the next section. The simple GGA functional BP86 [50, 51, 52] (referred to as BP) and the better double‐hybrid DSD‐BLYP [53] were both paired with Ahlrichs’ triple‐&zeta [54]; basis set def2‐TZVPP. All optimized structures were calculated with D3BJ dispersion correction [55, 56] and cleared of imaginary modes unless stated otherwise. The subsequent DLPNO‐CCSD(T) [57, 58] single‐points on the optimized double‐hybrid geometry use Dunning's correlation consistent quadruple‐ζ cc‐pVQZ [59, 60] and quintuple‐ζ cc‐pV5Z [59, 60] basis sets for an extrapolation to the complete basis set limit (CBS). All structures were optimized, both in the gas‐phase and with the CPCM model, followed by a DLPNO‐CCSD(T)/CBS single‐point calculation on the optimized DSD‐BLYP/def2‐TZVPP structure. To assess the performance of the double hybrid DFT method [61], DLPNO‐CCSD(T)/CBS single‐point calculations were also conducted on a subset of structures optimized only by BP/def2‐TZVPP. Figure 3 presents a simplified workflow schematic, emphasizing key challenges of the cluster continuum approach.

Note that, unlike gas‐phase structures, the quality of solvated structures is difficult to evaluate because of the lack of experimental data, both of the cluster structures and their solvation energies. The description of gas‐phase and continuum structures is less problematic for small solvent numbers *n*, when the bonds are still strong, but, since the cluster continuum model requires a larger number of partly rather weakly bound explicit solvent molecules, that may present challenges. For this reason and our prior work [2, 30, 31, 32, 33, 34, 35, 36, 37], the investigation was stopped at a number of *n* = 7 water molecules.

### 3.4 Benchmarking and Method Selection

Accurate experimental gas‐phase basicities (GBs) exist for almost all relevant solvents. Hence, as a first test of the quality of the methods, the GBs were assessed.

#### 3.4.1 Dependence of the Structure Optimization on the Magnitude of the Computed GBs

Table S7 in the Supporting Information summarizes all calculated and experimental [62] GBs together with the associated MAEs for each method and the differences in the bond lengths Δ*d*(H^+^–X) resulting from the two optimization methods. Despite being at the lower end of Jacob's ladder, the GGA functional BP still provides results close to experimental gas‐phase basicities. Although its mean absolute error (MAE, Supporting Information, Section 3.1) is slightly higher at 6.8 kJ mol^−1^, it remains a practical method for describing gas‐phase basicities, particularly given its faster computational time than more sophisticated methods. The energies improve further as the methods advance to the double‐hybrid functional DSD‐BLYP/def2‐TZVPP with an MAE of 3.9 kJ mol^−1^ and, especially, the coupled cluster calculations. With an MAE of 1.3 kJ mol^−1^, all DLPNO‐CCSD(T)/CBS energies are well within the commonly accepted ’chemical accuracy’ threshold of 1 kcal mol^−1^ (4.18 kJ mol^−1^).

### 3.5 Exemplary Investigation of the Effects of Clustering Protonated Water

**FIGURE 4:**
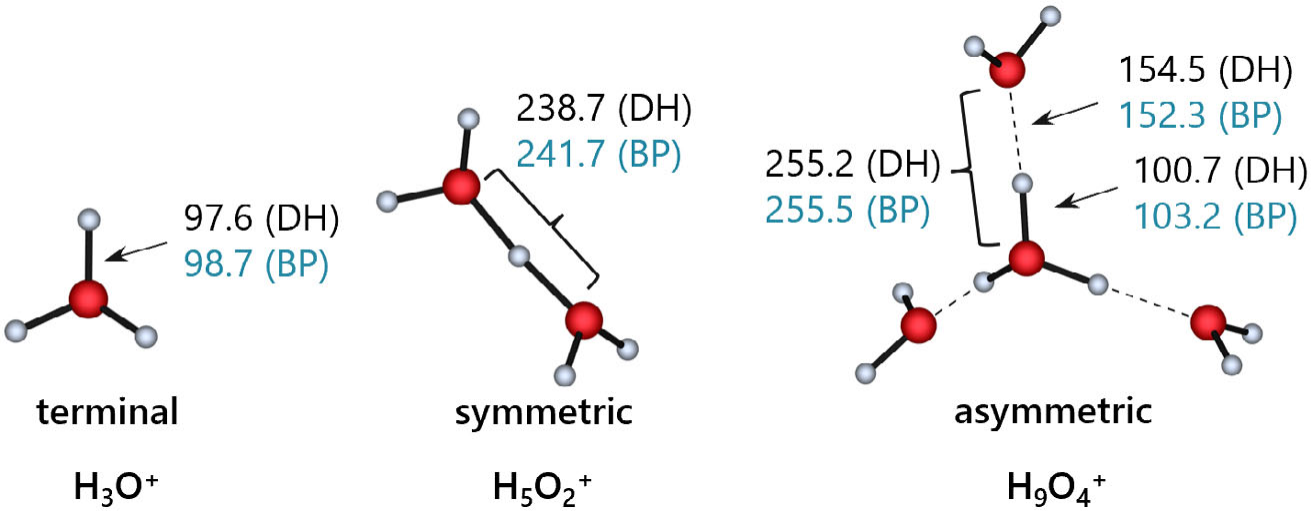
Comparison of the hydrogen bonded structures in H_3_O^+^, the symmetric hydrogen bond of H_5_O_2_^+^ and the asymmetric hydrogen bond of H_9_O_4_^+^ as a function of the method DSD‐BLYP/def2‐TZVPP (DH) and BP/def2‐TZVPP (BP), with distances given in pm.

As all these singly protonated molecules are small, closed‐shell systems with strong polar bonds and lack challenging features, such as bond‐stretch isomers, lower‐level DFT functionals provide sufficient accuracy. Thus, the difference Δ*d*(H^+^–X) of the bond lengths of the protonated donor atoms H^+^–X calculated with BP/def2‐TZVPP and DSD‐BLYP/def2‐TVPP methods only lies between 0.8 and 1.5 pm (Supporting Information, Table S7). Consequently, these structures are close enough to the “true” structure that coupled cluster single‐point calculations further improve the energies. Figure 4 shows the evolution of the hydrogen bond and O···O distances in going from the terminal hydrogen bond in H_3_O^+^ to the symmetric hydrogen bond in the H_5_O_2_^+^ Zundel [63] ion and then to the asymmetric hydrogen bonds in the H_9_O_4_^+^ Eigen [64] cation calculated by DSD‐BLYP/def2‐TZVPP and BP/def2‐TZVPP. With increasing number of solvent molecules *n*, the variations of the hydrogen bonds increase with the two methods, as well as in their gas‐phase clustering energies Δ_g_ *G*° summarized in Table 3. Although both methods assume a symmetric hydrogen bond, there is a 3 pm discrepancy in their description of the O···O distance within the H_5_O_2_^+^ ion and an about 2 pm variation of the asymmetric hydrogen bonds in H_9_O_4_^+^.

**TABLE 3:** Gas‐phase clustering energies Δ_g_ *G*° at 1 bar pressure of H_3_O^+^, H_5_O_2_^+^ and H_9_O_4_^+^ with terminal, symmetric, and asymmetric hydrogen bonds.

Cluster/H‐bond method	H_3_O^+^ terminal	H_5_O_2_^+^ symmetric	H_9_O_4_^+^ asymmetric
BP/def2‐TZVPP	665.8	789.4	891.1
DLPNO‐CCSD(T)/CBS//BP	657.7	761.4	851.1
DSD‐BLYP/def2‐TZVPP	663.3	779.2	879.2
DLPNO‐CCSD(T)/CBS//DH	657.0	763.3	854.7
Experiment	660.0 [62]	761.7 [65] /764.6 [66]	855.8 [65] /857.1 [66]

*Note:* The energies were obtained from optimizations using the BP/def2‐TZVPP and DSD‐BLYP/def2‐TZVPP methods and a subsequent DLPNO‐CCSD(T)/CBS single‐point on the respective optimized structures. Experimental data for the H_5_O_2_^+^ and H_9_O_4_^+^ ions was calculated through addition of the incremental gas‐phase energies −ΔΔ_g_ *G*°(*n* − 1, *n*) of the reaction H^+^(H_2_O)_*n*_ → H_2_O + H^+^(H_2_O)_*n*−1_ to the gas‐phase basicity of water at 300°K and 1 atm [62]. All energies are provided in kJ mol^−1^.

The difference per O‐H bond between the methods increases from 1.1 (H_3_O^+^), over 1.5 (H_5_O_2_^+^) to 2.5 pm (H_9_O_4_^+^), which is still quite small. Yet, it is reflected in the gas‐phase clustering energies at the DLPNO‐CCSD(T)/CBS level of theory, where the energies of the single points on the respective BP or double hybrid (DH) structures differ increasingly by 0.7, 1.9, and 3.6 kJ mol^−1^ for increasing ion size in H_3_O^+^, H_5_O_2_^+^, and H_9_O_4_^+^ (Table 3). Note that the better agreement with available experimental data for the two larger ions is achieved with coupled cluster single points on the DH structures.

Although the effects are still small, the BP/def2‐TZVPP method underestimates the asymmetric hydrogen bond of the Eigen cation and overestimates the covalent bonds within the central H_3_O^+^ unit. Optimized DSD‐BLYP/def2‐TZVPP structures in Table 3 improve results of the DLPNO‐CCSD(T)/CBS single‐point calculations in the clusters. A later example of the H_13_O_6_^+^ structure (see “Isomers and Their Weighting” below) also indicates that the double‐hybrid method is better suited to model structures with asymmetric hydrogen bonds. Yet, especially for the larger investigated organic solvent molecules the interactions may become increasingly more delicate, with dispersion and polar bonding being involved, and, therefore, introduce larger errors. As the exact solution structures of the particles in question are yet to be determined, this deficiency cannot be investigated in depths. Nevertheless, the double‐hybrid structures appear to be a good basis for the coupled cluster single‐point calculation.

#### 3.5.1 Larger Water Clusters

**FIGURE 5:**
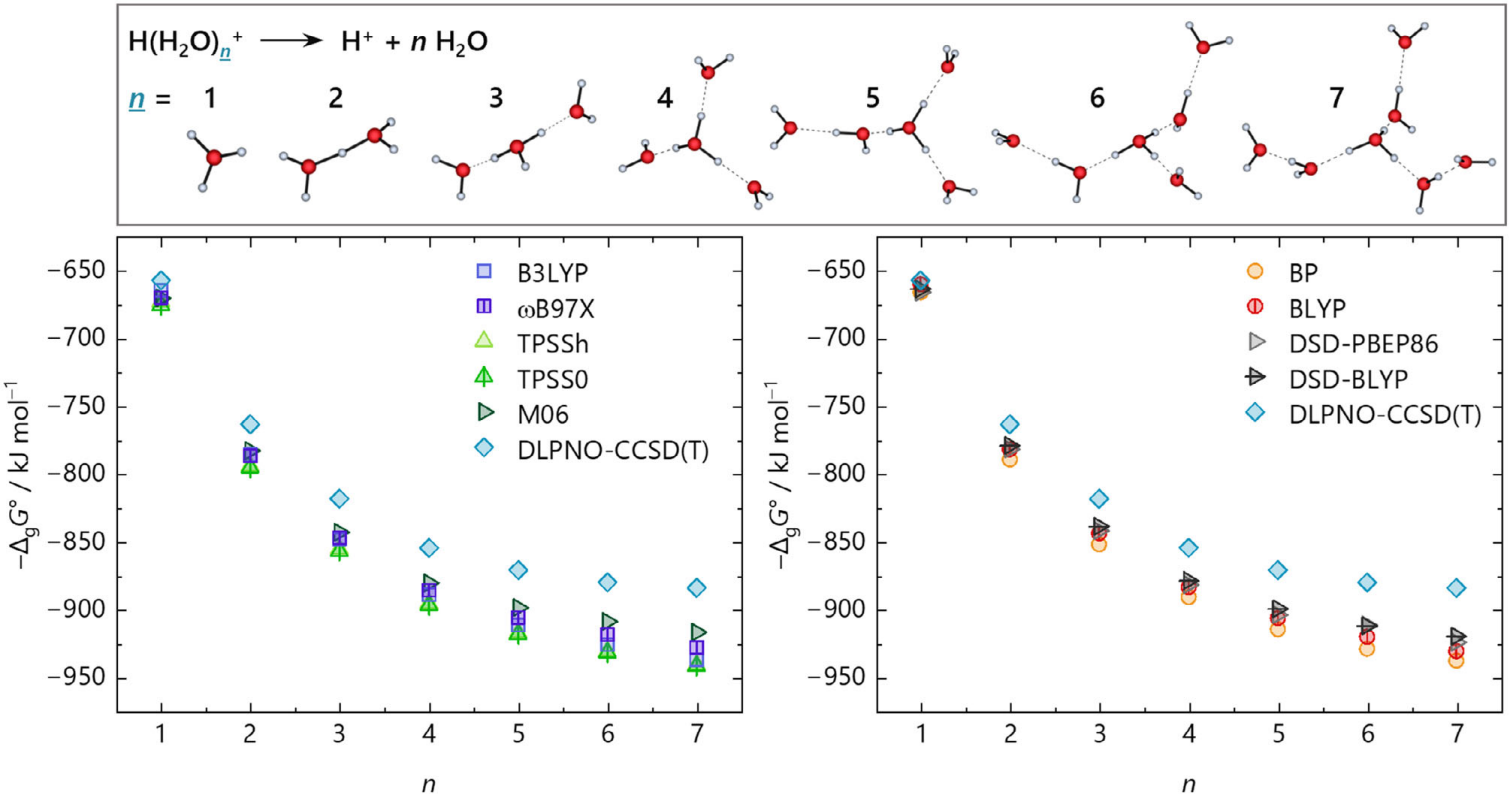
Gas‐phase clustering energies −Δ_g_ *G*° (monomer cycle) for the reaction H(H_2_O)_*n*_^+^ → H^+^ + *n* H_2_O of the selected protonated water clusters with *n* = 1–7 shown at the top as optimized with the density functionals BP, BLYP, DSD‐PBEP86, and DSD‐BLYP on the right side and ωB97X, B3LYP, TPSSh, TPSS0, and M06 on the left side. All functionals used the def2‐TZVPP basis set. DLPNO‐CCSD(T)/CBS single‐points only used the optimized DSD‐BLYP structures. The structures illustrated at the top were optimized at the DSD‐BLYP/def2‐TZVPP level of theory.

The difference in the gas‐phase clustering energies between the two density functionals is notably larger at 10.2 and 11.9 kJ mol^−1^ for the H_5_O_2_^+^ and the H_9_O_4_^+^ ions, respectively. This trend continues for the larger water clusters, where the differences between the methods become more pronounced at higher numbers of explicit solvent molecules *n*, shown in Figure 5 for protonated water clusters with up to seven water molecules. Here, the gas‐phase clustering energy −Δ_g_ *G*° (monomer cycle, Figure 1), calculated by structure optimizations with several methods and def2‐TZVPP basis set, as well as the “best” value from a DLPNO‐CCSD(T)/CBS single‐point calculation on the respective DSD‐BLYP structure, is plotted versus *n*. For simplicity, only one isomer per *n* value was considered in these calculations. Therefore, each *n* represents the same isomer, with method‐specific differences in bond lengths and angles. The DSD‐BLYP/def2‐TZVPP structures are shown at the top of Figure 5. In addition to the methods used in the previous section, the new calculations^2^ included structure optimizations with the GGA functional BLYP [52, 67], several hybrid and meta‐hybrid functionals, such as ωB97X‐D3BJ [68, 69, 70, 71], B3LYP [67, 72, 73, 74], TPSSh [75, 76], TPSS0 [77], the parameterized meta‐hybrid M06 [78], and the double‐hybrid DSD‐PBEP86 functional [79]. Note that for structure optimization purposes, typically a triple‐ζ basis set is large enough to be used with DFT calculations for light main group elements.

The discrepancies in the calculated energies with respect to the selected CCSD(T)/CBS reference energies increase with *n*. There is a noticeable up to 70 kJ mol^–1^ difference in −Δ_g_ *G*° between the functionals and the coupled cluster benchmark to evaluate the accuracy of the density functionals. This suggests either that the electronic description of the structures is much more complex than expected, leading to an overestimation of the energy by the density functionals, or that the structure differs substantially from what a structure optimization with DLPNO‐CCSD(T)/CBS would produce.

**FIGURE 6:**
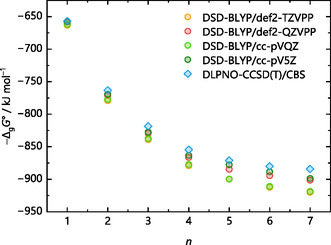
Gas‐phase clustering energies −Δ_g_ *G*° (monomer cycle) for the reaction H(H_2_O)_*n*_^+^ → H^+^ + *n* H_2_O of the selected protonated water clusters with *n* = 1–7 illustrated at the top of Figure 5. Energies result from geometry optimization with the DSD‐BLYP functional and the basis sets def2‐TZVPP, def2‐QZVPP, cc‐pVQZ, and cc‐pV5Z as well as a DLPNO‐CCSD(T) single‐point calculation on the optimized DSD‐BLYP/def2‐TZVPP structures.

The gas‐phase energies closest to the coupled cluster benchmark are both Martin's general purpose double‐hybrids [53, 79], especially for smaller values of *n*. While the Ahlrichs’ triple‐ζ basis set is perfectly fine for GGA, meta‐GGA, and even hybrid functionals, it should be mentioned that the MP2 component of the DSD‐BLYP method usually benefits from a larger basis set [53]. Again, using the protonated water clusters, Figure 6 demonstrates this for geometry optimizations with the DSD‐BLYP functional and Ahlrichs’ triple‐ζ def2‐TZVPP and quadruple‐ζ def2‐QZVPP basis sets as well as Dunning's quadruple‐ζ cc‐pVQZ and quintuple‐ζ cc‐pV5Z basis sets.

Hence, for the more advanced double‐hybrid functionals that incorporate MP2 correlation [80] and for larger clusters, the (larger) size of the basis set appears relevant, potentially due to less problems with respect to basis set superposition error, size consistency issues, or dispersion [81] correction. Yet, this is computationally prohibitive to achieve for the (larger) solvent systems of interest here.

### 3.6 Handling of Isomers in the Gas Phase, in Solution, and Their Weighting

In a real solvent S, various protonated species H(S)_*n*_^+^ may coexist at any given time, including different isomers and different solvent numbers *n*. The goal was to find the most stable structure for each *n* and its immediate neighbors, both in the gas‐phase and in the continuum environment, rather than a full exploration of the PES including all **high‐energy** isomers. Extensive searches have identified the most relevant isomers for the solvent clusters, especially for small values of *n*, yet the potential for more isomers remains. In addition, the most favorable structure is not absolute, but refers to the set of isomers identified by a particular method. This is a general shortcoming of the cluster continuum approach when high values of *n* are considered. However, only the global minimum and structures within its immediate vicinity are thermodynamically relevant. The total energy then determines the ranking of the isomers that share the same solvent number *n*, optimization method, and phase. Hence, the isomer with the most favorable energy in this phase starts at one, which is given after the chemical formula in parentheses: i.e., H_15_O_7_^+^ (2) is the energetically second most favorable isomer structure in the indicated phase. In the gas phase, the energetic order is related to the gas‐phase clustering energy Δ_g_ *G°*. For the neutral gaseous solvent clusters (S)_*n*_, this energy is the gas–phase interaction energy:

(15) $${\text{Δ}}_{\mathrm{int}}G{}^{\circ}=G{}^{\circ}({\left(S\right)}_{n})-nG{}^{\circ}(S)$$

Within the continuum, the structures of the isomers are ranked by their total electronic energies *E*_tot_(CPCM), with the structure of the isomer with the lowest electronic energy starting at one. The solvation energy of each cluster is calculated by subtracting the electronic energy *E*_tot_ of the structures in the gas phase from the electronic energy of the structure ranked (1) in the CPCM model. Finally, a correction factor of 7.96 kJ mol^–1^ (see (Equations 7) and (8)) must be added to obtain standard conditions for the transition from 1 bar gas to an ideal 1 mol L^−1^ solution:

(16) $${\text{Δ}}_{\text{solv}}G{}^{\circ}=E_{\mathrm{tot}}(\text{CPCM})+7.96-E_{\mathrm{tot}}(\text{gas‐phase})$$

Several structures were calculated both in the gas phase and in the continuum. Yet, only the isomer with the lowest electronic energy in the continuum *E*_tot_(CPCM) was used to obtain the solvation energies of the different gas‐phase clusters. Consequently, the solvation energy for each gas‐phase isomer (1)…(*n*) with the same value of *n* is calculated with respect to the same, most favorable, continuum isomer (1). The energies of the other isomers are listed in the Supporting Information, Section 3.3, but were not used for further calculations of the proton solvation energies. For gas‐phase isomers with the same *n* value, the calculated solvation energies usually show minor variations (2–6 kJ mol^−1^), with a few exceptions showing greater differences of 10 kJ mol^−1^.

Both gas‐phase and continuum structures of the neutral solvent clusters received identical treatment. However, as with the continuum structures, only the most favorable isomers for each *n* were used in the cluster cycle to determine the proton solvation energy. Protonated clusters with more than one explicit solvent molecule that lack a complete first coordination sphere have been systematically excluded from consideration, as they conflict with the concept of the cluster continuum model. Adding the strong interactions present in the immediate vicinity of the solute is the primary goal of the cluster continuum model. The final proton solvation energies calculated through the thermodynamic cycle for each isomer and solvent were Boltzmann‐weighted using (Equation 17) [2].

(17) $${\text{Δ}}_{\text{solv}}G{}^{\circ}\left(H^{+}\right)=-RT\ln\;\left(\sum_{i=1}^{n}\exp\;\left(\frac{-{\text{Δ}}_{\text{solv}}G_{i}^{{}^{\circ}}\left(H^{+}\right)}{RT}\right)\right)$$

The total weighted solvation energy is determined by the gas‐phase and the continuum structures, which result in the highest proton solvation energy Δ_solv_ *G*°(H^+^), rendering isomers that produce lower proton solvation energies insignificant. Consequently, the solvation energy of the proton in each solvent is defined by a specific *n* that leads to the highest solvation energy. According to (Equation 17), the isomers only contribute to the weighted solvation energy if the difference in their respective Δ_solv_ *G*°(H^+^) is less than 5 kJ mol^−1^. While ethanol had the most isomers for each *n* value, their effect on the final proton solvation energy was negligible.^3^

### 3.7 Exemplary Study of Structure and Energetics of Water Clusters

**FIGURE 7:**
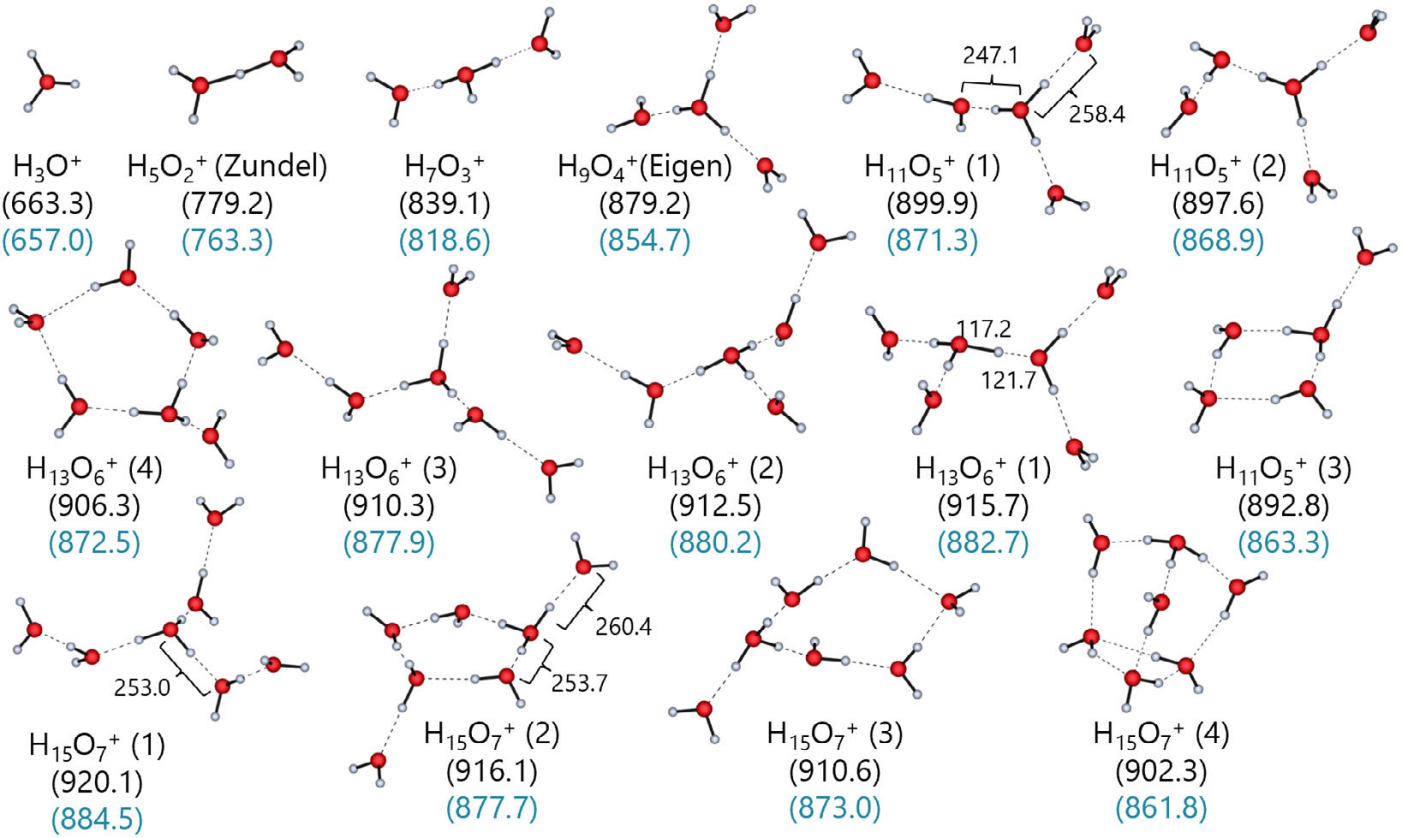
Optimized DSD‐BLYP/def2‐TZVPP gas‐phase structures of the protonated water clusters H(H_2_O)_*n*_^+^ for *n* = 1–7. The gas‐phase clustering energies Δ_g_ *G*° (H(H_2_O)_*n*_^+^ → H^+^ + *n*H_2_O) at 1 bar pressure are provided in kJ mol^−1^ at the DSD‐BLYP/def2‐TZVPP level of theory (black) and the subsequent DLPNO‐CCSD(T)/CBS single‐point calculation on the optimized structure (blue). Key interatomic distances (in pm) are shown for some of the most stable isomers.

In this work, three protic and five aprotic solvents were included. All examined solvent clusters are mostly stabilized by a network of hydrogen bonds. The stability of the clusters varies with the strength of these intermolecular interactions. The stronger the hydrogen bond network, the more consistent are the structures across different methods, as the non‐covalent interactions are more sensitive to the method. Water is known for its particularly strong hydrogen bond network, which is also the best researched one and was, therefore, selected as test case [82, 83, 84, 85, 86, 87]. Most of the calculated protonated water clusters have a more or less distorted Eigen‐ or Zundel‐ion at their center. Figure 7 shows the optimized DSD‐BLYP/def2‐TZVPP gas‐phase structures of the protonated water clusters H(H_2_O)_*n*_^+^ with the respective Δ_g_ *G*° energies obtained at the same level and the DLPNO‐CCSD(T)/CBS single‐point energies on the optimized DSD‐BLYP/def2‐TZVPP structures highlighted in blue.

Note that the double‐hybrid's energetic ordering of all isomer structures is confirmed by the DLPNO‐CCSD(T)/CBS single‐point calculations on the optimized structure shown in blue.

By contrast, and although the isomers identified by the BP/def2‐TZVPP method are visually indistinguishable to the double hybrid optimized versions, their gas‐phase clustering energies Δ_g_ *G*° presented in the Supporting Information in Section 2.7 show more pronounced differences compared to the energies calculated with the DSD‐BLYP functional, which again become more prominent with increasing *n*. For example, BP/def2‐TZVPP finds the H_13_O_6_^+^ (1) isomer in a Zundel‐type structure, with two symmetric hydrogen bonds (Figure 7) and not an asymmetric one like in the double hybrid structure. The two coupled cluster single‐point calculations on the respective H_13_O_6_^+^ (1) structures differ by 8.6 kJ mol^−1^. Note that indirect vibrational spectroscopy data by Stoyanov et al. suggest H_13_O_6_^+^ to be the main species in acidic aqueous solutions [88]. The proposed structure is of an distorted Zundel type with some Eigen‐character, supposed to have a long O···O separation largely exceeding 243 pm. Both, DSD‐BLYP/def2‐TZVPP and the BP/def2‐TZVPP found this “type” of structure to be the most favorable isomer among the four H_13_O_6_^+^ isomers, yet with a shorter O···O separation of 239.9 (DH) and 242.7 pm (BP).^4^

More importantly, the energetic order assigned to the isomer structures by their DLPNO‐CCSD(T)/CBS single‐points differs from the order determined by BP/def2‐TZVPP for six out of 15 clusters in Figure S15. The isomer order for *n* ≥ 6 from the coupled cluster single‐points matches that from DSD‐BLYP/def2‐TZVPP optimizations, which consistently agrees with the single‐point calculations on the optimized DSD‐BLYP/def2‐TZVPP structures. In essence, the BP/def2‐TZVPP method models the structures adequately, but its gas‐phase energy calculations lack the precision to rank the isomers correctly. An assessment of the incremental gas‐phase energies −ΔΔ_g_ *G*°(*n* − 1, *n*) obtained with DSD‐BLYP/def2‐TZVPP and BP/def2‐TZVPP, together with the energies from the respective DLPNO‐CCSD(T)/CBS single‐point calculations on the optimized structures and the experimental energies in Table 4 leads to the same conclusion. The DLPNO‐CCSD(T)/CBS single‐point calculations on the DSD‐BLYP structures provides the most accurate gas‐phase energies. The DLPNO‐CCSD(T)/CBS single‐point calculations on the double‐hybrid structures have the lowest MAE values of 3.1 and 2.3 kJ mol^−1^ for both experimental datasets. However, substantial structural differences remain significant, as suggested by the (6, 7) energy in Table 4.

**TABLE 4:** Incremental gas‐phase energies −ΔΔ_g_ *G*°(*n* − 1, *n*) of the reaction H^+^(H_2_O)_*n*_ → H_2_O + H^+^(H_2_O)_*n*−1_ at 1 bar pressure for the protonated water clusters obtained from optimizations with the BP/def2‐TZVPP and DSD‐BLYP/def2‐TZVPP methods and the DLPNO‐CCSD(T)/CBS single‐point calculations on the respective optimized structures with two sets of experimental data.

(*n* − 1, *n*)	DSD‐BLYP/def2‐TZVPP	DLPNO‐CCSD(T)/CBS//DH	BP/def2‐TZVPP	DLPNO‐CCSD(T)/CBS//BP	Exp^a^	Exp^b^
1, 2	115.80	106.32	123.64	103.71	101.67	104.60
2, 3	59.95	55.24	62.89	54.70	54.39	56.90
3, 4	40.09	36.12	38.79	35.03	39.75	35.56
4, 5	20.71	16.58	23.78	15.44	23.43	23.01
5, 6	15.82	19.43	14.96	16.12	17.15	16.32
6, 7	4.40	11.98	8.64	3.84	12.55	11.72
MAE^a^	5.4	3.1	6.3	4.1	—	—
MAE^b^	4.8	2.3	5.6	3.2	—	—

a Obtained at 300 K and 1 atm [65].

b Obtained at 300 K and 1 atm [66].

*Note:* All values are given in kJ mol^−1^.

A large energy difference between the experimental (6, 7) value and the calculated BP/def2‐TZVPP‐derived coupled cluster single‐point energy suggests that one of the gas‐phase structures is poorly represented. The two density functionals gave conflicting rankings of the H_15_O_7_^+^ isomers.

Overall, the preceding sections demonstrated that the DSD‐BLYP/def2‐TZVPP method, combined with the coupled cluster single points extrapolated to the basis set limit, is the method of choice.

### 3.8 Calculated Structures and Energies in Solution

Before turning to all solvents, we will again use the water system as an exemplary test case, and then transfer the methodology first to the closest relatives of water, the alcohols MeOH, and EtOH and then also to the aprotic solvents.

#### 3.8.1 Exemplary Investigation of Solvated Water Clusters

**FIGURE 8:**
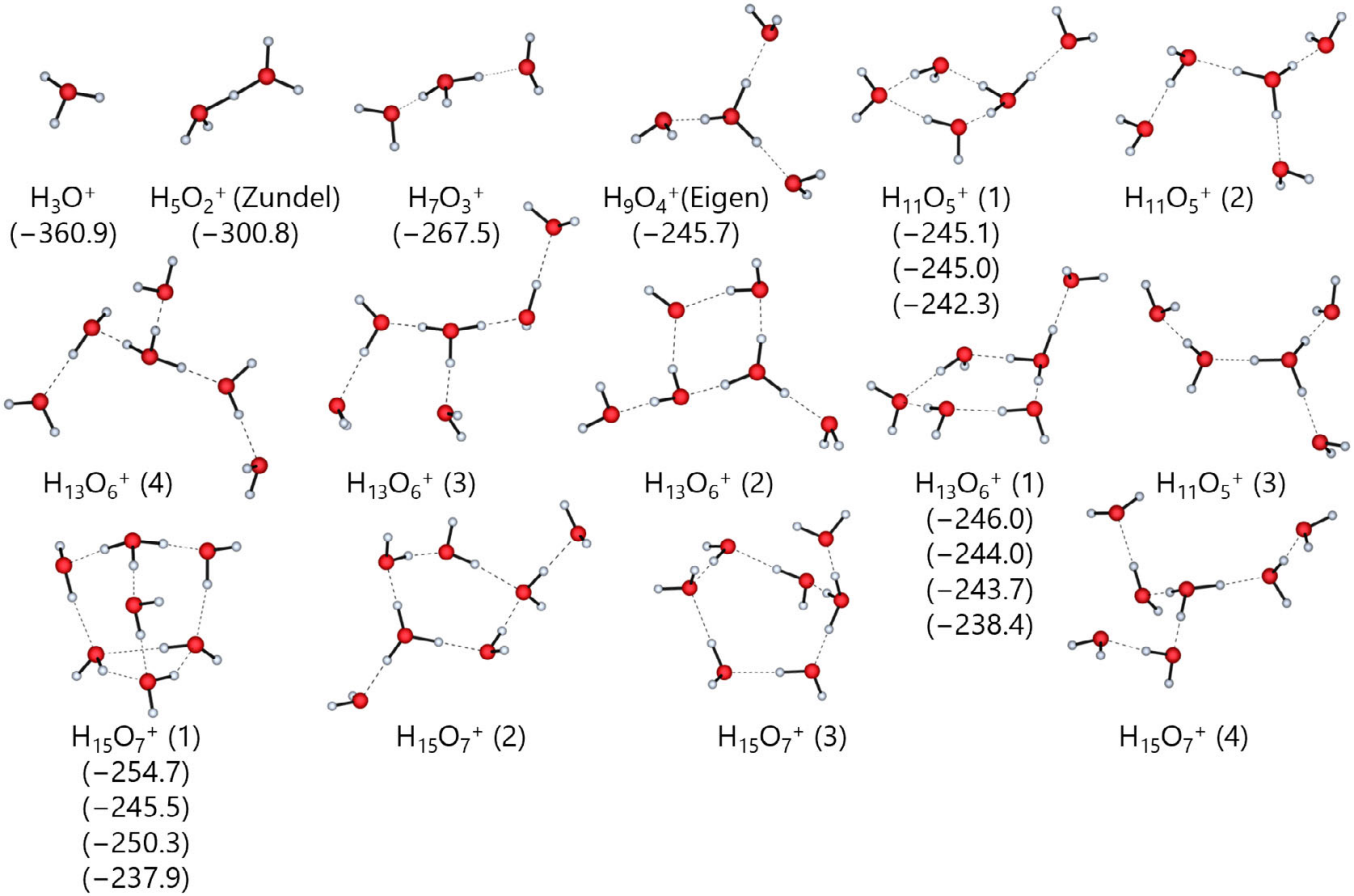
Optimized DSD‐BLYP/def2‐TZVPP‐CPCM structures of the protonated water clusters H(H_2_O)_*n*_^+^ for *n* = 1–7 with the respective standard solvation energies Δ_solv_ *G*° in kJ mol^−1^. All calculated isomers are shown, but only the isomers with the lowest electronic energy labeled with (1) were used to calculate the solvation energies.

Figure 8 shows the CPCM continuum structures optimized by DSD‐BLYP/def2‐TZVPP together with the calculated Δ_solv_ *G*° solvation energies. In small clusters, these structures resemble their gas‐phase counterparts, but their appearance gradually changes as more solvent molecules are added. The structures still derive from the Zundel and Eigen ions, but show increasing distortions: The continuum favors cage‐ and ring‐like structures over the chain‐like structures found in the gas‐phase, with no exception to the exemplarily discussed H_13_O_6_^+^ ion. A solution structure of this ion between the Eigen and Zundel types was proposed [88]. However, such a type is not present among the investigated CPCM structures, unless the isomer H_13_O_6_^+^ (2) is considered to be a further distorted variant of the experimental structure. Regardless, the isomer calculated to be the most stable is the ring‐like H_13_O_6_^+^ (1) isomer.

Apparently, these clusters are only an approximation of their real‐world counterparts, because in a continuum, they lack the full three‐dimensional hydrogen bonding network found in a real solvent. This stabilizes certain ring and cage structures more than they would be in a real solvent. The very limited data available on real solvated structures prevents further discussion, but the fact that the continuum model is a crude approximation of a solvent rather than a true solvent raises the question of whether the resulting structures need to reflect real solvated structures to be able to describe the thermodynamics of the solution. A real solvent is highly dynamic, containing multiple coexisting and interacting species, which cannot be captured by the static calculations in this study. Hence, a properly applied thermodynamic cycle may possibly eliminate the impact of these structural inconsistencies as well as other errors in the methods applied to obtain the final proton solvation energies. Some of these points also extend to the gas‐phase structures, particularly within the thermodynamic cycles. Nevertheless, the cluster continuum description seems to capture the general trends in the solvation energies of the clusters, as illustrated by the increase in the solvation energies from the H_9_O_4_^+^ to the H_13_O_6_^+^ clusters, even though the experiment, as stated above, suggests a different structure [88].

##### 3.8.1.1 Solvation Versus Gas Phase Clustering Energies

**FIGURE 9:**
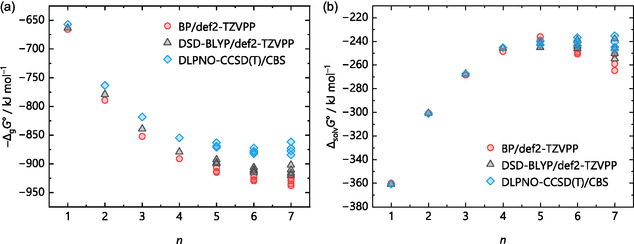
(a) Standard gas‐phase clustering energies Δ_g_ *G*° (H(H_2_O)_*n*_^+^ → H^+^ + *n* H_2_O) and (b) standard solvation energies Δ_solv_ *G*° of the protonated water clusters H(H_2_O)_*n*_^+^ with *n* = 1–7 calculated at the BP/def2‐TZVPP, DSD‐BLYP/def2‐TZVPP levels as well as DLPNO‐CCSD(T)/CBS single‐point calculations on the optimized DSD‐BLYP/def2‐TZVPP structures.

Fortunately, the solvation energies of the protonated clusters display similar patterns to the gas‐phase clustering energies of the respective gas‐phase structures, but are less sensitive to changes in the computational method or subtle structural alterations. Figure 9 shows a comparison of the gas‐phase clustering energies −Δ_g_ *G*° of the protonated water clusters with (a) *n* = 1–7 and the corresponding standard solvation energies of these clusters (b) Δ_solv_ *G*°. These energies were obtained from the BP/def2‐TZVPP, DSD‐BLYP/def2‐TZVPP optimizations as well as the DLPNO‐CCSD(T)/CBS single‐points on the DSD‐BLYP/def2‐TZVPP structure.

Figure 9 shows that the three applied methods have a significantly greater effect on the gas‐phase energies than on the solvation energies. This can be attributed to the mainly electrostatic nature of the solvation energy, which makes it less dependent on the functional and the basis set. Although the differences in the solvation energies calculated by the different methods increase with the number of solvent molecules, the overall variations remain small. In fact, the solvation energies are almost identical for clusters with *n* < 4. For the most stable clusters with *n* = 7, the difference between the solvation energies of the double‐hybrid and the subsequent coupled cluster single‐point calculation is 7.5 kJ mol^−1^. The difference between the same single‐point and BP/def2‐TZVPP is only 3.5 kJ mol^−1^. By contrast, the gas phase Δ_g_ *G*° values differ by 35.7 / 53.6 kJ mol^−1^ between the most favorable isomers with *n* = 7 for the double‐hybrid / the BP structure and its coupled cluster single‐point.

#### 3.8.2 Structures and Energies of the Alcohol‐ Versus Water‐Clusters

**FIGURE 10:**
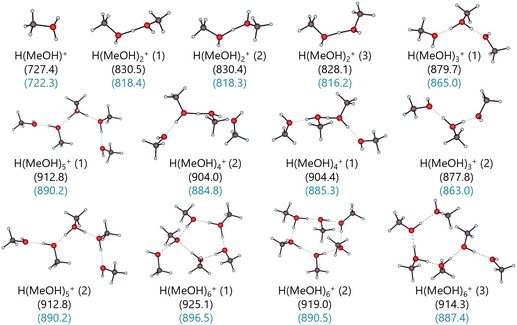
Optimized DSD‐BLYP/def2‐TZVPP gas‐phase structures of the protonated methanol clusters H(MeOH)_*n*_^+^ for *n* = 1–6. The gas‐phase clustering energies Δ_g_ *G*° (H(MeOH)_*n*_^+^ → H^+^ + *n* MeOH) are provided in kJ mol^−1^ at the DSD‐BLYP/def2‐TZVPP level of theory (black) and the subsequent DLPNO‐CCSD(T)/CBS single‐point calculation on the optimized structure (blue).

We now turn to the closest relatives of water, the alcohols MeOH and EtOH. The comparison of the protonated methanol clusters H(MeOH)_*n*_^+^ with *n* = 1–6 (Figure 10 and Figures S15/ S16, Supporting Information) shows an identical energetic ordering of the isomers at the DSD‐BLYP/def2‐TZVPP level and with the subsequent coupled cluster single points. In addition, and unlike the protonated water clusters, the DLPNO‐CCSD(T)/CBS energy ordering fortunately also agrees between the BP86 and DSD‐BLYP functionals. This hints at organic solvent clusters being more consistent than water clusters when using these two density functionals to optimize their structures. Comparing ethanol and methanol, their protonated clusters form analogous structures, linked by O‐H^+^…O and O‐H…O hydrogen bonds that organize into either zigzag chains or cyclic patterns, as illustrated in Figure 10 (MeOH) and Figure S21 (EtOH; *n* = 1–5) in the Supporting Information.

Where possible, the most stable structures feature alternating methyl and ethyl groups arranged along the line created by the O‐H^+^…O and O‐H…O hydrogen bonds. Structures with non‐alternating methyl and ethyl groups were consistently less favorable in the gas‐phase. For each value of *n*, the ethanol clusters resulted in the largest number of isomers of all tested solvents. Several were energetically very close and contributed slightly to the Boltzmann‐weighted solvation energy of the proton; most in the monomer cycle with *n* = 3 by 3 kJ mol^−1^. Also, neutral clusters of methanol and ethanol showed similar structures with alternating methyl or ethyl groups along the O‐H…O bonds (Figures S17 and S20, Supporting Information).

##### 3.8.2.1 Clustering Gibbs Energies: The Influence of Calculated Enthalpic and Entropic Contributions

**FIGURE 11:**
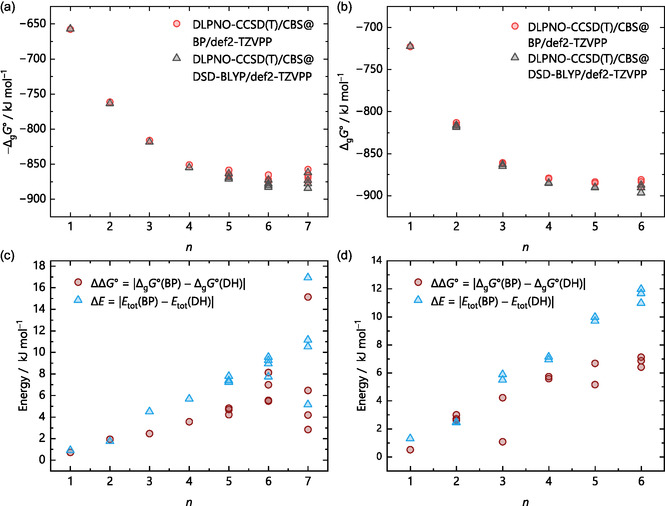
Gas‐phase clustering energies Δ_g_ *G*° (H(S)_*n*_^+^ → H^+^ + *n* S) of the (a) protonated water clusters H(H_2_O)_*n*_^+^ with *n* = 1–7 and (b) the protonated methanol clusters H(MeOH)_*n*_^+^ with *n* = 1–6. The energies result from the DLPNO‐CCSD(T)/CBS single‐point on the on the optimized BP/def2‐TZVPP and DSD‐BLYP/def2‐TZVPP structures, respectively. The two lower graphs illustrate the difference in the electronic energies of the two single‐point calculations (Δ*E* = *E*_tot_(BP) – *E*_tot_(DH)) and the difference between the gas‐phase clustering energies from the same single‐points (ΔΔ*G*° =,Δ_g_ *G*°(BP) − Δ_g_ *G*°(DH)|) for (c) the protonated water and (d) the protonated methanol clusters. BP refers to DLPNO‐CCSD(T)/CBS single‐point energies on optimized BP/def2‐TZVPP structures and DH refers to the DLPNO‐CCSD(T)/CBS single‐point energies on DSD‐BLYP/def2‐TZVPP structures. All energies are given in kJ mol^−1^.

Figure 11 shows the gas‐phase clustering uenergies Δ_g_ *G*° from the DLPNO‐CCSD(T)/CBS single‐point calculations on the respective BP/def2‐TZVPP and DSD‐BLYP/def2‐TZVPP structures of the protonated water clusters H(H_2_O)_*n*_^+^ on the left (a) and the protonated MeOH clusters H(MeOH)_*n*_^+^ on the right (b) in the upper panels of the figure. Starting from *n* = 5, the gas‐phase clustering energies show a clear, albeit small, distinction that increases further with *n*. The difference is insignificant for clusters with less than five solvent molecules and is overall in a similar range for both solvents. Yet, these DLPNO‐CCSD(T)/CBS calculations still include the thermal and entropic corrections from the BP/def2‐TZVPP and the DSD‐BLYP/def2‐TZVPP calculations. Here, the double‐hybrid produces shorter bonds within the harmonic approximation and overestimates the force constants, while BP overestimates the bond lengths and underestimates the force constants, which leads to a coincidental error cancelation within the harmonic description for BP, effectively matching a frequency scaling factor of unity.

To shed more light on this influence, the two lower graphs of Figure 11 illustrate the contribution of the entropic and enthalpic corrections for the protonated water (c) and the protonated methanol clusters (d): the differences between the electronic energies Δ*E* = *E*_tot_(BP) – *E*_tot_(DH) of the two CCSD(T)/CBS single‐point calculations on the BP/def2‐TZVPP (BP) and DSD‐BLYP/def2‐TZVPP structures (DH), as well as the difference in their gas‐phase clustering energies ΔΔ*G*° =,Δ_g_ *G*°(BP) − Δ_g_ *G*°(DH)| are plotted against *n*. At low to moderate *n*, the change between the electronic Δ*E* and the gas‐phase energy differences ΔΔ*G*° is small, but it becomes more pronounced as *n* increases. Thus, the observed differences in the gas‐phase clustering energies between the DLPNO‐CCSD(T)/CBS single‐point calculations for the two functionals are primarily due to methodological differences, rather than solely from errors in the entropy terms. However, the spread of the entropic contributions becomes more significant as *n* increases. In addition, with flexible organic moieties like a methyl group, this effect starts at smaller *n* for MeOH (at 3) than for water (at 6). Despite the limited number of structures, this observed trend extends to most protonated and non‐protonated clusters across the other solvents studied in this work. Hence, especially for larger organic solvent molecules with flexible moieties and low energy vibrations, this might introduce further errors.

#### 3.8.3 Application of the Methodology to Aprotic Solvents

The preceding analysis of methanol and water clusters showed the double‐hybrid functional to be highly reliable, providing gas‐phase energies that are, for small *n,* close to the DLPNO‐CCSD(T)/CBS single‐point calculations on the optimized structures. This strongly suggests that the optimized structures are a good approximation of the real structures. The rest of this section now examines the general bonding situation in the solvent clusters optimized with the double‐hybrid functional, without further investigation of the BP/def2‐TZVPP structures. The Supporting Information, Section 3.2 contains details of any structures not specified here, while the Supporting Information, Section 3.3 provides a solvent‐sorted list of all calculated gas‐phase and solvation energies for the relevant neutral and cationic clusters.

##### 3.8.3.1 Protonated and Neutral Acetonitrile Clusters

**FIGURE 12:**
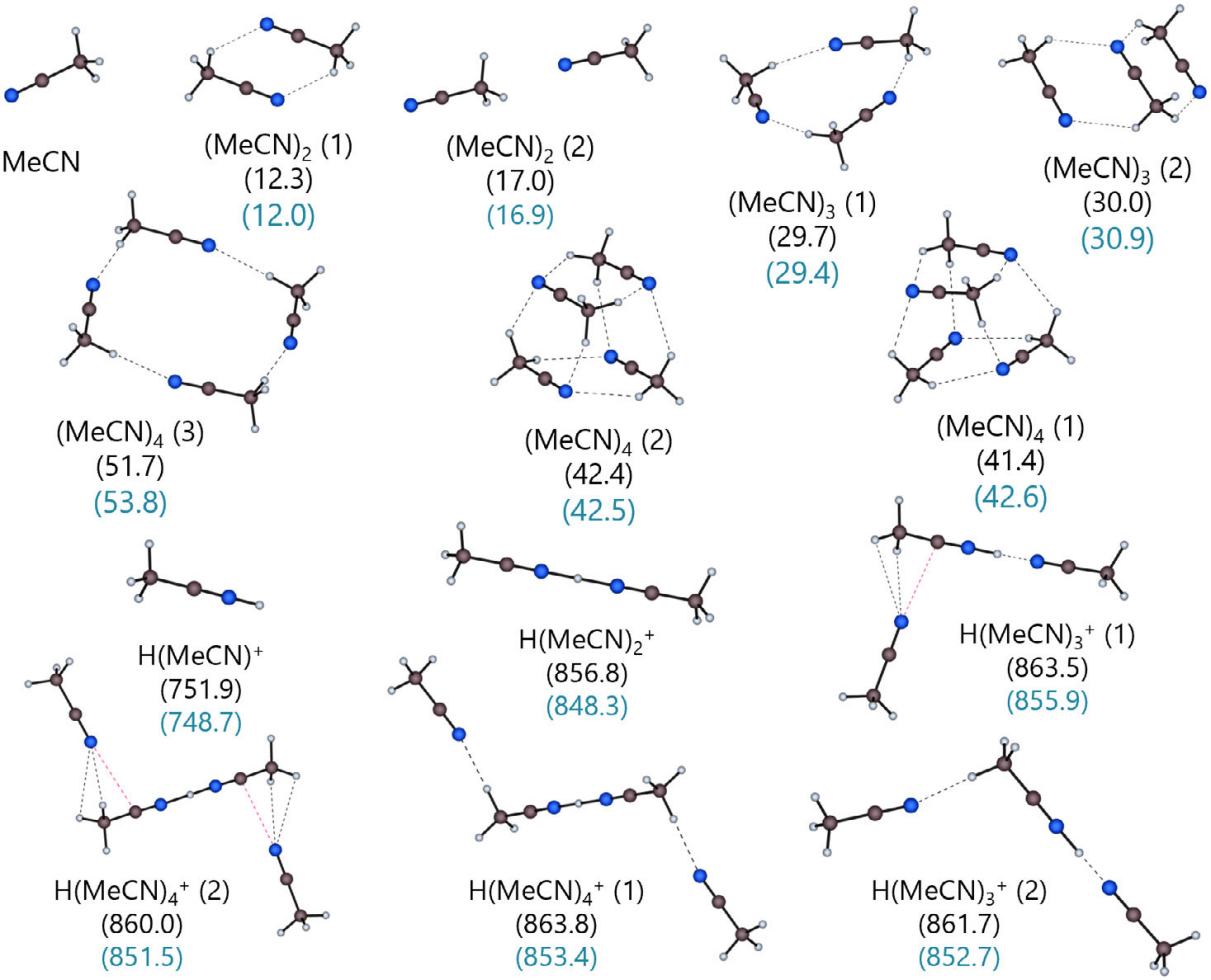
Optimized DSD‐BLYP/def2‐TZVPP gas‐phase structures of the non‐protonated and protonated MeCN clusters with *n* = 1–4. The gas‐phase clustering energies Δ_g_ *G*° (monomer cycle) are provided in kJ mol^−1^ at the DSD‐BLYP/def2‐TZVPP level of theory (black) and the subsequent DLPNO‐CCSD(T)/CBS single‐point calculation on the optimized structure (blue). The N^δ−^…C^δ+^ interactions in the structures of the isomers H(MeCN)_3_^+^ (1) and H(MeCN)_4_^+^ (2) are highlighted with red dotted lines.

Acetonitrile clusters differ principally from protonated and non‐protonated clusters formed by the other investigated solvents: It is the only solvent with a N‐donor atom, and some of its clusters are stabilized by additional N^δ−^…C^δ+^ interactions. Figure 12 shows the optimized gas‐phase structures of the protonated and neutral MeCN clusters up to *n* = 4 at the DSD‐BLYP/def2‐TZVPP level of theory, including the corresponding gas‐phase energies in kJ mol^−1^. Hydrogen bonds of the C‐H…N type stabilize the unprotonated (MeCN)_*n*_ clusters, which adopt cyclic or cage‐like structures with a head‐to‐tail orientation. The dimer (MeCN)_2_ converged in a cyclic form with antiparallel orientation of the dipoles and a collinear structure with the molecules arranged head‐to‐tail, which is slightly less stable than the antiparallel orientation. The protonated dimer H(MeCN)_2_^+^ exists in a single linear orientation that is connected by an N–H^+^…N hydrogen bond. This dimer is at the center of all computed gas‐phase geometries with *n* > 2. Further acetonitrile molecules bind through C‐H…N interactions, occasionally reinforced by N^δ−^…C^δ+^ interactions involving the carbon atom of the dimer and the nitrogen atom of the additional acetonitrile molecules.

**FIGURE 13:**
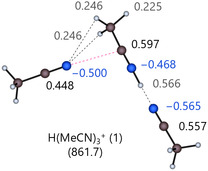
Optimized DSD‐BLYP/def2‐TZVPP gas‐phase structure of the H(MeCN)_3_^+^ cluster with the respective calculated CHELPG partial charges given in elementary charge units *e*.

These N^δ−^…C^δ+^ interactions are also present in several of the non‐protonated structures and are highlighted in red in Figure 12 for the structures of H(MeCN)_3_^+^ (1) and H(MeCN)_4_^+^ (2). Figure 13 shows exemplarily the H(MeCN)_3_^+^ (1) isomer with its atomic charges calculated with the Charges from Electrostatic Potentials using a grid‐based method (CHELPG) scheme at the DSD‐BLYP/def2‐TZVPP level.

The nitrogen atom bound to the two hydrogen atoms in the CH_3_ group of the dimer carries a calculated, high negative partial charge of −0.5*e*, whereas the carbon atom it is connected to exhibits a high positive charge of 0.6*e* due to the protonation of the acetonitrile. Malloum and Conradie have reported many of the structures presented here, as well as additional structures at different levels of theory and have found that structures stabilized by additional N^δ−^…C^δ+^ interactions are more stable [89, 90, 91]. They report that structures with an antiparallel orientation of the molecules, such as the cage‐like structures of the neutral MeCN clusters, are all stabilized by additional N^δ−^…C^δ+^ interactions [91]. Many of the structures they reported left the proton with an unsaturated coordination site. As noted earlier, these structures were intentionally excluded as they disregard the significant interactions within the solute's immediate vicinity. Some structures could not be reproduced accurately due to the presence of imaginary modes. Consequently, these structures were omitted, which includes all collinear structures with *n* > 2. Compared to isomers bound by hydrogen bonds and N^δ−^…C^δ+^ interactions, the collinear structures exhibit smaller gas‐phase clustering energies, making them less significant.

##### 3.8.3.2 Protonated and Neutral DMSO Clusters

For DMSO, the gas‐phase calculations at the DSD‐BLYP/def2‐TZVPP level of theory found two isomers of the H(DMSO)_2_^+^, which are linked through an O‐H^+^…O hydrogen bond between the two sulfinyl groups (*n* = 1–4, Figures S24/ S25, Supporting Information). Isomer H(DMSO)_2_^+^ (1) has a 7.7 kJ mol^−1^ greater gas‐phase clustering energy than isomer (2) and was found to be the main building block of structures with *n* > 2. Additional DMSO molecules form two identical C‐H…O hydrogen bonds with the two methyl groups of one DMSO in the dimer, forming H(DMSO)_3_^+^. Steric hindrance in the H(DMSO)_4_^+^ cluster prevents the formation of symmetric hydrogen bonds. The unprotonated structures are all linked by two C‐H…O hydrogen bonds between the two CH_3_ groups that are symmetric, if the geometrical arrangement allows for it.

##### 3.8.3.3 Protonated and Neutral DMF Clusters

The gas‐phase clusters of the protonated DMF show some similarities to those of DMSO. The two H(DMF)_2_^+^ isomers, connected by O‐H^+^…O bonds, are in a somewhat distorted form part of the larger clusters (*n* = 1–4, Figures S22/ S23, Supporting Information). Through C‐H…O interactions, the protonated dimer binds additional DMF molecules, exhibiting a stronger attraction to the carbonyl hydrogen than to the methyl groups. Non‐protonated clusters primarily form C‐H…O interactions involving carbonyl hydrogen atoms, displaying an antiparallel dipole orientation whenever the geometry permits it.

##### 3.8.3.4 Protonated and Neutral Propylene carbonate (PC) Clusters

**FIGURE 14:**
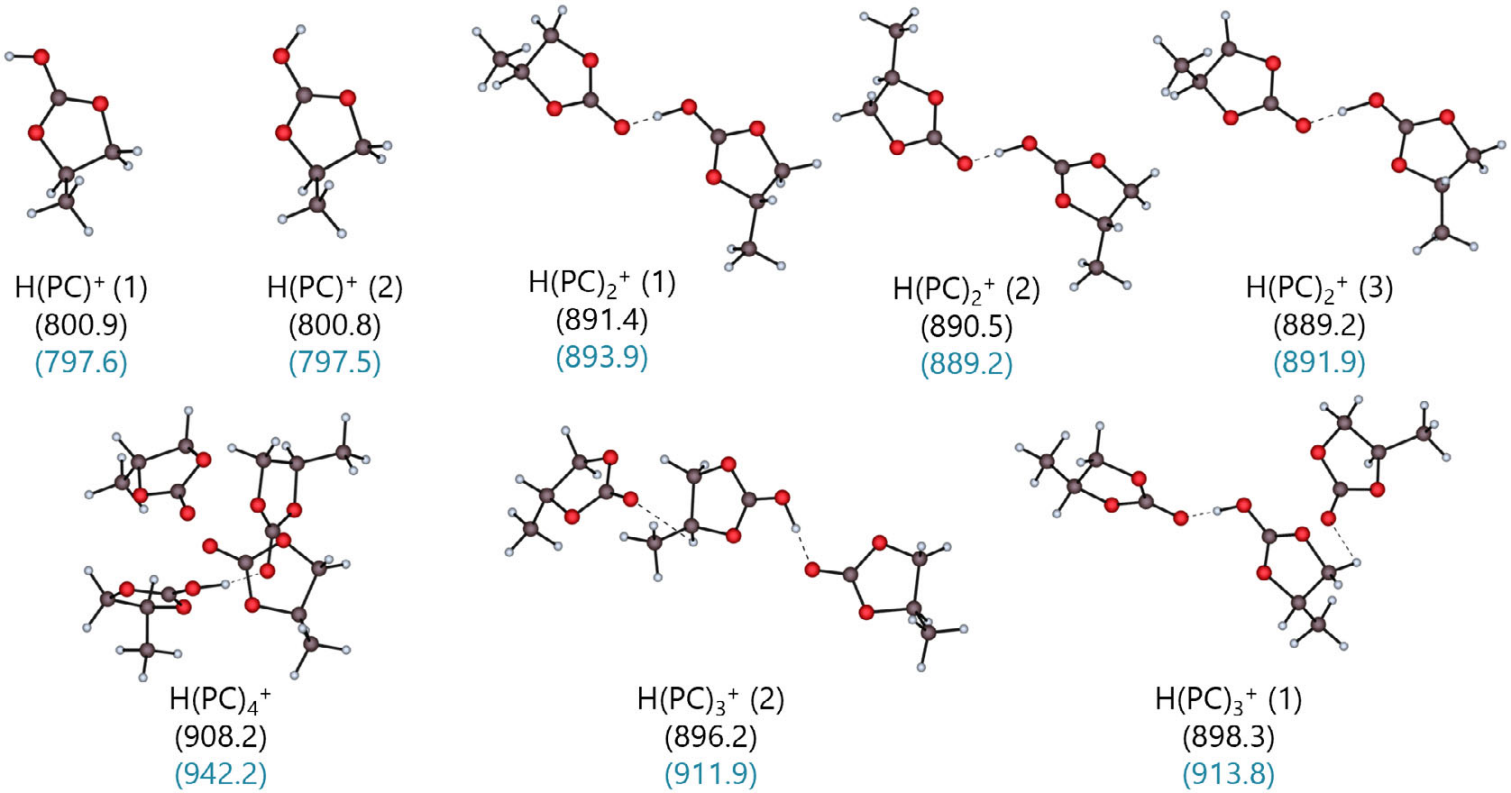
Optimized DSD‐BLYP/def2‐TZVPP gas‐phase structures of the protonated PC clusters H(PC)_*n*_^+^ for *n* = 1–4. The gas‐phase clustering energies Δ_g_ *G*° according to the monomer cycle at 1 bar pressure are provided in kJ mol^−1^ at the DSD‐BLYP/def2‐TZVPP level of theory (black) and the subsequent DLPNO‐CCSD(T)/CBS single‐point calculation on the optimized structure (blue).

PC has three oxygen atoms, each of which can form hydrogen bonds with the CH and CH_2_ groups of the five‐membered ring or with the methyl group. Figure 14 visualizes the protonated propylene carbonate clusters, with the dimer H(PC)_2_^+^ (1), which is stabilized by O‐H^+^…O bonds, again at the center of all the larger protonated clusters.

The carbonyl oxygen in the PC molecule forms a hydrogen bond with a hydrogen atom on the ring system of the protonated dimer. The two neutral dimers are linked by two C‐H…O hydrogen bonds between the carbonyl oxygen and the CH_2_ group of the ring system (Figure S26, Supporting Information). With opposing dipoles, the molecules align to form a distorted head‐to‐tail arrangement between pairs. The formation of cage‐like structures in the larger clusters still maintains the head‐to‐tail bonding pattern, but with more distortion.

##### 3.8.3.5 Protonated and Neutral MeFo Clusters

Methyl formate (MeFo) has two different oxygen atoms, but the calculations show that the structures favor connections through O‐H^+^…O bonds between the carbonyl oxygens (*n* = 1–3, Figures S27/ S28, Supporting Information), mirroring the behavior of the protonated propylene carbonate clusters very much. The protonated and neutral clusters are very similar in their pattern of non‐covalent bonds, with additional molecules binding via CH or CH_3_ groups to the dimers, which vary in their structures due to the flexibility of the molecules.

This overview of the calculated protonated and neutral solvent clusters highlights the main features of the structures, but it is not an exhaustive exploration. Only the most favorable of these structures are relevant, since the thermodynamic cycles focus on the Gibbs energy generated by a proton in this medium. Hence, the following discussion will be limited to the BP86/def2‐TZVPP and DSD‐BLYP/def2‐TZVPP structures. Unless specified otherwise, all subsequent DLPNO‐CCSD(T)/CBS single‐point calculations apply only to the optimized DSD‐BLYP/def2‐TZVPP structures.

### 3.9 Toward Gibbs Energies of Proton Solvation in Solution

Next, the calculation of the Protons’ Gibbs Energy of solvation is investigated in the selected solvents. First, the prerequisites, that is, the preferred number of solvent molecules around the proton, are analyzed, before the thermodynamic monomer and cluster cycles are applied and evaluated.

#### 3.9.1 Is There an Optimal Number of Explicit Solvent Molecules?

The patterns observed in the gas‐phase and in the solvation energies of the protonated water and methanol clusters are, to a varying extent, present in the other solvents. Both the solvation energies Δ_solv_ *G*°(H(S)_*n*_^+^) and the gas‐phase clustering energies Δ_g_ *G*°(H(S)_*n*_^+^) slowly reach a plateau or a minimum, with a clear distinction in *n* between protic and aprotic solvents. This trend provides a way to estimate an optimal number of explicit solvent molecules for each solvent from the plateau of the gas‐phase energies. This raises the question of how many explicit solvent molecules are needed around the proton. The gas‐phase clustering energy is not only the most accurate contribution within the thermodynamic cycle, but it is also highly sensitive to the method, especially for higher values of *n*, unlike the solvation energies of the clusters. Therefore, a practical approach to identifying the optimal *n* is to evaluate the gas‐phase energies for each method.

##### 3.9.1.1 Using the Monomer Cycle

**FIGURE 15:**
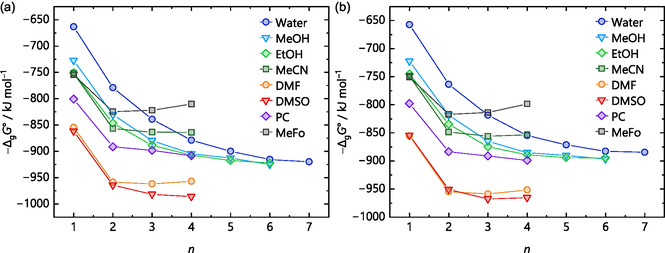
Gas‐phase clustering energies −Δ_g_ *G*° (H(S)_*n*_^+^ → H^+^ + *n* S) of the protonated solvent clusters H(S)_*n*_^+^ of water, methanol, ethanol, acetonitrile, methyl formate, propylene carbonate, DMF, and DMSO obtained from a (a) DSD‐BLYP/def2‐TZVPP optimization and (b) a DLPNO‐CCSD(T)/CBS single‐point calculation on the optimized DSD‐BLYP/def2‐TZVPP structures. Only the isomer with the most favorable gas‐phase clustering energy for each *n* of each solvent is included.

To avoid the additional uncertainty introduced by the neutral solvent clusters, only the gas‐phase energies from the monomer cycle in Figure 1 were considered. However, the energies obtained from the cluster cycle demonstrate analogous trends. Figure 15 displays the gas‐phase clustering energies −Δ_g_ *G*° for all solvents, showing only the isomer structures with the most favorable Δ_g_ *G*° when multiple isomers were found for a given *n* with DSD‐BLYP/def2‐TZVPP structures/energies (a) and or the subsequent DLPNO‐CCSD(T)/CBS single‐point energy (b).

It appears that protic solvents such as water, methanol, and ethanol require more explicit solvent molecules than aprotic solvents in order to reach a plateau. In the framework of the continuum model, the clusters formed are primarily bound by strong hydrogen bonds. The stronger these bonds, the more bound solvent molecules are energetically favorable, and the larger the clusters become, which must be considered in the cluster continuum model. Naturally, water, with its particularly strong hydrogen‐bonded network, requires the largest amount of explicit solvent molecules, about six or seven, to reach the plateau. A similar pattern can be observed for the protonated clusters of methanol and ethanol. Both solvents need about five or six solvent molecules to reach a plateau, whereas the aprotic solvents require only three or four molecules. These numbers can vary depending on the level of theory, but remained consistent for the selected model chemistries DSD‐BLYP/def2‐TZVPP or the DLPNO‐CCSD(T)/CBS single‐point calculation. While this approach provides an estimated range of solvent numbers, it does not provide an exact value for each individual solvent and leaves an uncertainty, since only one isomer is considered for this estimate.

##### 3.9.1.2 Including Continuum Solvation

**FIGURE 16:**
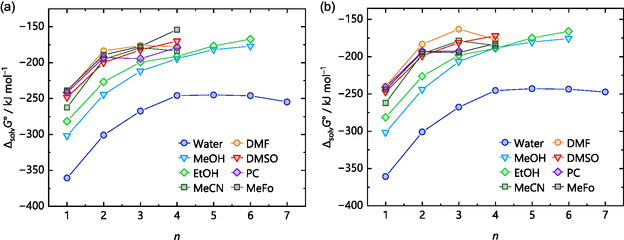
Standard solvation energies Δ_solv_ *G*° of protonated solvent clusters H(S)_*n*_^+^ of water, methanol, ethanol, acetonitrile, methyl formate, propylene carbonate, DMF, and DMSO obtained from a geometry optimization with DSD‐BLYP/def2‐TZVPP (a) and a DLPNO‐CCSD(T)/CBS single‐point calculation on the optimized DSD‐BLYP/def2‐TZVPP structures (b). This graph only includes the solvation energy of isomer (1) in both the gas‐phase and the continuum.

The trends observed in the gas‐phase clustering energies are also present for the calculated Gibbs solvation energies of the clusters, but with more variation. Figure 16 displays their standard Gibbs solvation energies, calculated with DSD‐BLYP/def2‐TZVPP and DLPNO‐CCSD(T)/CBS. Again, the focus is laid exclusively on the solvation energy of the most favorable isomer (1), considering both the gas‐phase and a continuum model, since the solvation energy is calculated as the difference between the electronic energies of the continuum and the gas‐phase structures.

Unlike gas‐phase energies, solvation energies in most solvents do not plateau at the same number of solvent molecules and may not progress as smoothly into the plateau. In addition, continuum solvation energies tend to be less accurate than gas‐phase energies, particularly for small values of *n*. Increasing *n* generally enhances the accuracy of solvation energies as first‐shell effects diminish, which is offset by a decrease in gas‐phase energy accuracy. The gas‐phase and solvation energies exhibit contrasting trends in accuracy, with the solvation energies improving as *n* increases and first solvation shell effects become less prevalent, while the gas‐phase energies become less accurate. The clusters should be of sufficient size to enhance the continuum calculations while maintaining good accuracy in the gas‐phase energies. Therefore, medium‐sized clusters are ideal for maintaining this balance, but the number of explicit solvent molecules can unfortunately not be chosen arbitrarily.

##### 3.9.1.3 Changing the Cycle

Some researchers relate the optimal cluster size directly to the plateau of the single‐ion solvation energy resulting from a thermodynamic cycle [2]. However, these cycles contain additional contributions and may contain experimental rather than calculated values. Unlike the computed gas‐phase energies of the clusters, these energies may not plateau until much larger values of *n*, as will become clear in B.3.3 “Proton Gibbs Solvation Energies” which includes a discussion.

### 3.10 Exemplary Evaluation and Comparison of Cluster and Monomer Cycles for Water

**FIGURE 17:**
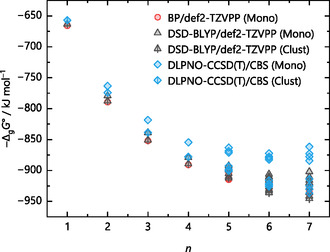
Gas‐phase clustering energies −Δ_g_ *G*° (H(H_2_O)_*n*_^+^ → H^+^ + *n* H_2_O) at 1 bar pressure for protonated water clusters with *n* = 1–7 obtained through the gas‐phase reaction in the monomer and cluster cycles, respectively. The geometry optimizations were conducted at the BP/def2‐TZVPP and DSD‐BLYP/def2‐TZVPP level of theory, followed by DLPNO‐CCSD(T)/CBS single‐points on the optimized DSD‐BLYP/def2‐TZVPP structures.

The monomer and the cluster cycles described in Figure 1 are the two thermodynamic cycles that are commonly combined with the cluster continuum model. The monomer cycle does not consider the clustering of the neutral solvent molecules, neither in the gas‐phase nor in solution. However, the energy difference is significant, depending on whether the energy of an individual solvent molecule is multiplied by its corresponding number *n* or if a solvent cluster is used instead. Figure 17 visualizes this for the protonated water clusters with *n* = 1–7 and the three methods BP/def2‐TZVPP, DSD‐BLYP/def2‐TZVPP, and DLPNO‐CCSD(T)/CBS.

The gas‐phase energies obtained from the cluster cycle are considerably more negative than those obtained from the monomer cycle. Both cycles show the previously described trends, including the widening gap between the methods as *n* increases and the plateau that the energies eventually reach. We assume that the cluster cycle provides an overall higher accuracy due to better error compensation. It can be proposed that energies derived from the cluster cycle at the DLPNO‐CCSD(T)/CBS level of theory provide the most precise representation of the gas‐phase energies, when calculating the solvation energy of the proton.

#### 3.10.1 Fortuitous Error Cancelation With BP86

Interestingly, the overestimated gas‐phase energies from the BP86/def2‐TZVPP method combined with the monomer cycle are remarkably consistent with the energies calculated within the cluster cycle and either DSD‐BLYP/def2‐TZVPP or DLPNO‐CCSD(T)/CBS. This has implications on the performance of the two thermodynamic cycles. As shown in Figure 3, the solvation energies of the protonated clusters are less sensitive to the method than the gas‐phase energies. The inclusion of the gas‐phase energy as a negative term in the proton solvation energy calculation, as shown in Equations (11) and (12), results in a fortunate error cancelation for the BP/def2‐TZVPP method, when combined with the monomer cycle. For some solvents, this combination achieves coincidentally comparable accuracy to the computationally far more demanding double‐hybrid and coupled cluster methods.

#### 3.10.2 Differences Between Monomer and Cluster Cycle

**FIGURE 18:**
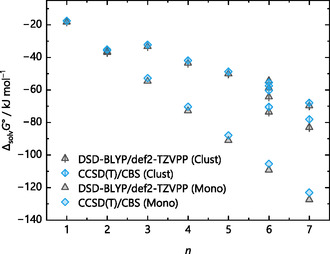
Standard solvation energies Δ_solv_ *G*° for the neutral water clusters with *n* = 1–7 used in the cluster cycle (clust). The solvation energies of the single solvent molecule multiplied by *n* as utilized in the monomer cycle (mono) are provided as a reference. The optimizations were conducted at the DSD‐BLYP/def2‐TZVPP level of theory, along with DLPNO‐CCSD(T)/CBS single‐points on the optimized DSD‐BLYP/def2‐TZVPP structures.

Since the cluster cycle uses the calculated solvation energies of neutral solvent clusters rather than simply multiplying the solvation energy calculated for a single solvent molecule by the number of molecules, this approach captures more directed interactions. The difference between these two contributions is captured in Figure 18 for the neutral solvent clusters of water with DSD‐BLYP/def2‐TZVPP and DLPNO‐CCSD(T)/CBS solvation calculations.

The solvation energies of the solvent clusters are, as expected, lower than the sum of the individual solvent molecule solvation energies. The net effect of these energies in Equations (11) and (12) is a positive contribution to the final proton solvation energy, resulting in a smaller, less negative proton solvation. Therefore, the cluster cycle yields a more negative proton Gibbs solvation energy than the monomer cycle, correlating more closely with the experimental results. Note that this difference can reach values exceeding 50 kJ mol^–1^ or an equivalent of almost 9 orders of magnitude, e.g., pH units.

### 3.11 Proton Solvation Gibbs Energies From Measurements and Their Uncertainty

Direct comparison of the calculated proton solvation energies is difficult because no experimentally measured values are available for any of the solvents studied. However, the proton solvation energies in the organic solvents can be derived from Tissandier's composite value for the proton hydration [27] and the experimentally measured transfer energies according to (Equation 3). At places, where transfer energies from ILSB measurements were not available, energies obtained using the TATB assumption [40] were used instead. Table 5 shows the protons’ solvation and transfer Gibbs energies obtained thereby.

**TABLE 5:** Combined experimental standard proton solvation energies Δ_solv_ *G*°(H^+^, S) in kJ mol^−1^ obtained through Δ_solv_ *G*°(H^+^, S) = −1104.5 [27] + Δ_trans_ *G*°(H^+^, H_2_O → S), with the TATB [40] values, where no ILSB values were available.

Energy / method	WA	MeOH	EtOH	DMF	DMSO	PC	MeCN	MeFo
Δ_solv_ *G*°(H^+^, S)^a^	−1104.5	−1096.9	−1097.8	−1122.5	−1123.9	−1054.5	−1056.0	−1071.2
Δ_trans_ *G*°(H^+^, H_2_O → S) / TATB	—	10.5	11.1	−18.0	−19.4	50.0	46.4	—
Δ_trans_ *G*°(H^+^, H_2_O → S) / ILSB	—	7.7	6.7	—	—	—	48.5	33.3

a Estimated uncertainty of ±14 kJ mol^−1^ for the combined proton solvation energies of all organic solvents.

#### 3.11.1 Estimation of Error Bars for the Experimental Values

Unfortunately, the above procedure also merges the relatively large and mostly estimated error bars. While Tissandier's value for Δ_solv_ *G*°(H^+^, H_2_O) already has a substantial uncertainty of ±8 kJ mol^−1^, variations in the proton hydration energies determined by other methods suggest that this uncertainty could be even larger [5]. The uncertainty associated with the TATB assumption is typically around ±3–6 kJ mol^−1^, but can be considerably higher [6, 7, 8, 9, 10, 11, 92]. The TATB proton transfer Gibbs energies from Marcus et al. [40]. are weighted averages of multiple measurements, primarily TATB data, to which we assign an estimated uncertainty of ±6 kJ mol^−1^ [42]. Given that the uncertainties are approximate, larger uncertainties are possible for individual measurements. As discussed above, the accuracy of the ILSB measurements is estimated to ± 0.6 kJ mol^−1^. Regardless, similar to the TATB assumption, the uncertainties of the individual transfer energies could be higher, but not higher than that of the TATB method, ± 6 kJ mol^−1^. Finally, the total error bar is another estimate from these combined uncertainty estimates of the transfer energies and the proton hydration energies. It amounts to approximately ±14 kJ mol^−1^ for all combined proton solvation energies. While this is a wide range, it is only an estimate and could be exceeded for some values.

The large estimated error bars, coupled with the need for extra‐thermodynamic assumptions, pose a significant challenge in judging the error or quality of the calculated proton Gibbs solvation energies. Unfortunately, the calculated energies can also cover a considerable range.

### 3.12 Calculated Proton Solvation Gibbs Energies and Comparison to Measurements

#### 3.12.1 Evaluation of the Calculated Data

**FIGURE 19:**
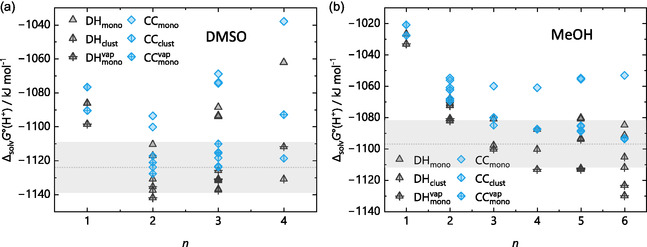
Unweighted standard solvation energy of the proton Δ_solv_ *G*°(H^+^) in DMSO on the left and MeOH on the right determined through an DSD‐BLYP/def2‐TZVPP optimization and a subsequent DLPNO‐CCSD(T)/CBS single‐point calculation on the optimized DSD‐BLYP/def2‐TZVPP structure, along with the experimental value (Table 5) of −1123.9 for DMSO [40] and −1096.9 for methanol, highlighted as reference lines. The combined error bar of ±14 kJ mol^−1^ of the Δ_solv_ *G*°(H^+^, S) value is highlighted as a solid gray area. DH denotes calculations performed using the DSD‐BLYP/def2‐TZVPP method, while CC refers to calculations using the DLPNO‐CCSD(T)/CBS method. The corresponding cycle is indicated by the subscript, and the inclusion of the evaporation energies of the solvents Δ_vap_ *G*°(S) (provided in Section 2.4, Supporting Information) is denoted by the superscript “vap".

Naturally, when all the components are combined to calculate the solvation energies of the proton, a familiar trend emerges as the value of *n* increases. The solvation energies initially increase until they reach a plateau or minimum, similar to the patterns seen in the gas‐phase and the solvation energies of the protonated clusters. Figure 19 illustrates this with the unweighted standard solvation energies of the proton Δ_solv_ *G*°(H^+^) in methanol and DMSO calculated with both the DSD‐BLYP/def2‐TZVPP (DH) and the DLPNO‐CCSD(T)/CBS (CC) methods, respectively. A subscript added to the method abbreviation indicates the type of thermodynamic cycle.

The reference lines represent the combined experimental solvation energies, with the error bar of ±14 kJ mol^−1^ highlighted as a solid gray area. The plots also include a modified monomer cycle, where the calculated self‐solvation energy of the solvent was replaced by its experimental Gibbs energy of evaporation Δ_vap_ *G*°(S). A superscript “vap” appears after each method's abbreviation in these cycles. The evaporation Gibbs energies used in these cycles are summarized in Table S5, Supporting Information, along with all solvent properties used in these calculations.

As *n* increases, there is a sharper drop in the curves toward the minimum, but the following upward trend is less distinct as the number of isomers increases. Methanol, unlike DMSO, shows a plateau rather than a clear minimum, supporting earlier findings that protic solvents require additional solvent molecules. Overall, the protic solvents follow the trend established by methanol on the right side of the graph, while the aprotic solvents track the pattern set by DMSO on the left side. These trends are obscured in the Boltzmann‐weighted proton solvation energies, because the isomer with the highest solvation energy dominates the weighted solvation energy. Section 3.4, Supporting Information, summarizes the weighted and unweighted proton solvation energies for each solvent.

Hence, the following analysis of the weighted solvation energies is divided into three sections: monomer cluster cycle performance (Section 3.12.2), cluster cycle performance (Section 3.12.3), and an overall evaluation of the cluster continuum approach and both thermodynamic cycles (Section 3.12.4).

#### 3.12.2 Evaluation of the Monomer Cycle

Although the monomer cycle is probably less effective because it lacks the error correction inherent in the cluster cycle, it remains practical when paired with an appropriate level of theory. Table 6 summarizes the Boltzmann‐weighted standard solvation Gibbs energies calculated using the monomer cycle and the BP/def2‐TZVPP, DSD‐BLYP/def2‐TZVPP, and DLPNO‐CCSD(T)/CBS methods.

**TABLE 6:** Boltzmann‐weighted standard proton solvation energies Δ_solv_ *G*°(H^+^, S) in kJ mol^−1^ in water, MeCN, DMF, DMSO, EtOH, MeOH, MeFo, and PC, sorted in ascending order by the solvent number *n*.

Method	*n*	WA	MeOH	EtOH	DMF	DMSO	PC	MeCN	MeFo
BP/def2‐TZVPP (vap)	2	−1093.0	−1088.7	−1088.0	−1128.9	−1142.0	−1060.8	−1068.2	−1042.0
	3	−1124.9	−1111.5	−1106.9	−1129.0	−1142.1	−1060.8	−1072.8	−1042.4
	4	−1145.2	−1125.4	−1121.3	−1129.0	−1142.1	−1060.8	−1073.9	−1042.4
BP/def2‐TZVPP	2	−1075.4	−1077.9	−1081.5	−1112.8	−1123.1	−1046.7	−1038.8	−1024.3
	3	−1098.5	−1095.3	−1097.1	−1112.8	−1123.1	−1046.7	−1038.9	−1024.3
	4	−1110.1	−1103.9	−1108.3	−1112.8	−1123.1	−1046.7	−1038.9	−1024.3
DSD‐BLYP/ def2‐TZVPP/ (vap)	2	−1082.8	−1084.3	−1078.5	−1128.0	−1142.1	−1051.9	−1056.1	−1032.2
	3	−1110.7	−1103.1	−1093.2	**−1128.1**	**−1142.1**	**−1052.0**	**−1056.5**	**−1032.2**
	4	−1130.5	−1114.6	−1101.9	**−1128.1**	**−1142.1**	**−1052.0**	**−1057.8**	**−1032.2**
	5	−1152.8	**−1116.2**	**−1102.7**	—	—	—	—	—
	6	**−1170.5**	**−1130.2**	**−1102.8**	—	—	—	—	—
	7	**−1184.6**	—	—	—	—	—	—	—
DSD‐BLYP/ def2‐TZVPP	2	−1063.5	−1071.5	−1070.5	−1109.1	−1117.2	−1033.3	−1028.0	−1012.8
	3	−1081.8	−1083.9	−1081.1	**−1109.1**	**−1117.2**	**−1033.3**	**−1028.0**	**−1012.8**
	4	−1091.9	−1089.2	−1086.1	**−1109.1**	**−1117.2**	**−1033.3**	**−1028.0**	**−1012.** **8**
	5	−1104.5	**−10** **89.3**	**−1086.2**	—	—	—	—	—
	6	**−1** **112.6**	**−1092.5**	**−1086.2**	—	—	—	—	—
	7	**−1117.4**	—	—	—	—	—	—	—
DLPNO‐CCSD(T)/CBS/ (vap)	2	−1067.1	−1071.7	−1066.2	−1124.0	−1127.9	−1044.0	−1048.3	−1025.0
	3	−1090.3	−1082.9	−1078.0	**−1124.0**	**−1127.9**	**−1044.1**	**−1048.** **6**	**−1025.0**
	4	−1105.6	−1089.3	−1081.4	**−1124.0**	**−1127.** **9**	—	**−1049.5**	**−1025.0**
	5	−1121.9	**−1091.6**	**−1081.5**	—	—	—	—	—
	6	**−1135.0**	**−1100.2**	**−1081** **.5**	—	—	—	—	—
	7	**−1141.7**	—	—	—	—	—	—	—
DLPNO‐CCSD(T)/CBS	2	−1000.2	−1058.4	−1057.4	−1102.4	−1100.4	−1024.6	−1020.8	−1005.1
	3	−1039.7	−1063.3	−1064.9	**−1102.4**	**−1100.4**	**−1024.6**	**−1020.8**	**−1005.1**
	4	−1074.2	−1064.6	−1065.8	**−1102.4**	**−1100.4**		**−1020.8**	**−1005** **.1**
	5	−1081.7	**−1064.7**	**−1065.9**	—	—	—	—	—
	6	**−1095.2**	**−1065.0**	**−1065.9**	—	—	—	—	—
	7	**−1068.8**	—	—	—	—	—	—	—
**Literature** a		**−1104.5**	**−1096.8**	**−1097.8**	**−1122.5**	**−1123.9**	**−1054.5**	**−1056.0**	**−1071.** **2**

a Solvation Energies obtained through Δ_solv_ *G*°(H^+^, S) = −1104.5 [27] + Δ_trans_ *G*°(H^+^, H_2_O → S) as summarized in Table 5. A CPCM calculation of the protonated and unprotonated PC cluster with *n* = 4 was not feasible at the DLPNO‐CCSD(T)/CBS level of theory.

*Note:* All energies were calculated with the monomer cycle and BP/def2‐TZVPP, DSD‐BLYP/def2‐TZVPP as well as a DLPNO‐CCSD(T)/CBS single‐point calculation on the optimized DSD‐BLYP/def2‐TZVPP structure. The monomer cycles that include the Gibbs evaporation energy Δ_vap_ *G*°(S) (from Section 2.4, Supporting Information) instead of the calculated solvation energy Δ_solv_ *G*°(S) of the solvent S are marked with “vap". Estimates of the optimal values of *n*, derived from the gas‐phase energies in section 0 are highlighted in bold for each solvent.

One of the more obvious trends overall is the significant decline in the monomer cycle's performance as the level of theory improves. When considering the Boltzmann‐weighted solvation energies of all solvents and all values of *n* given in Table 6, the mean absolute error of the monomer cycle with DLPNO‐CCSD(T)/CBS is 36.6 kJ mol^−1^. The MAE of the double‐hybrid is much lower at 19.9 kJ mol^−1^, but remains high. The performance of the more advanced methods is notably enhanced by replacing the solvation energy of the solvent molecule, which was identified as the main source of error, with the evaporation energy of the solvent Δ_vap_ *G*°(S). This substantially improves the energies resulting from the monomer cycle, bringing the coupled cluster results to an MAE of 16.4 kJ mol^−1^, which is close to the cluster cycle with DLPNO‐CCSD(T)/CBS (13.6 kJ mol^−1^), which is the best performing cycle in this work. Given the substantial errors in the combined experimental solvation energies noted above, this represents a reasonably good performance.

The improvement through the evaporation energy is less apparent in the DSD‐BLYP/def2‐TZVPP calculations, albeit still present when its MAE is considered. While considering evaporation energy slightly lowers the MAE from 19.9 to 18.5 kJ mol^–1^, the effect is not uniform across all solvents.

For some systems, theoretical monomer cycle calculations provide better agreement with experimental proton solvation energies. This is probably due to the error cancelation caused by the overestimation of the gas‐phase clustering energies. This cancelation is at its peak with the BP86/def2 TZVPP method. Its gas‐phase energies show significant differences compared to the coupled cluster benchmark, which coincidentally reduces the mean absolute error for the solvation energies to 14.3 kJ mol^−1^. The combination of BP86/def2‐TZVPP and the monomer cycle does not improve when the calculated solvation energy of the solvent is replaced by its energy of evaporation Δ_vap_ *G*°(S). In fact, the MAE increases slightly to 16.4 kJ mol^−1^, although certain solvents show improved results. The reported MAE values include solvation energies for all *n* values, which differ depending on the method that was used. This makes a direct comparison of these values somewhat challenging. Nevertheless, the MAE values will provide a concise metric for a comparison of the methods in Table 7.

**TABLE 7:** Mean absolute error in kJ mol^−1^ of the Boltzmann‐weighted standard solvation energies of the proton Δ_solv_ *G*°(H^+^, S) calculated with the monomer cycle and BP/def2‐TZVPP, DSD‐BLYP/def2‐TZVPP, as well as a DLPNO‐CCSD(T)/CBS single‐point calculation on the optimized DSD‐BLYP/def2‐TZVPP structure.

BP86/def2‐TZVPPVap	BP86/def2‐TZVPP	DSD‐BLYP/def2‐TZVPPvap	DSD‐BLYP/def2‐TZVPP	DLPNO‐CCSD(T)/CBSVap	DLPNO‐CCSD(T)/CBS
16.4	14.3	18.5	19.9	16.4	36.6
13.0	9.0	9.5	15.7	10.2	30.2

*Note:* The label “vap” refers to the inclusion of the experimental energy of evaporation Δ_vap_ *G*° (Supporting Information, Section 2.4) into the cycle. The top row displays MAE values for all solvents, while the row below excludes water and methyl formate.

Non‐polar MeFo and very polar water appear to be special cases, because most methods under‐/ overestimate their proton solvation energy. The methyl formate issues will be discussed below. Yet, water is a difficult case, as the proton hydration energies can be excellent depending on the specific number of solvent molecules involved. The best estimate for the solvent number, as derived from the gas‐phase clustering energies, fell between six and seven. At this point, the calculated proton solvation energy surpasses the experimental value with most of the methods. In contrast, using DLPNO‐CCSD(T)/CBS with the experimental evaporation energy and *n* = 4 results in an excellent value of −1105.6 kJ mol^−1^, while DSD‐BLYP/def2‐TZVPP accurately replicates the Tissandier value of −1104.5 kJ mol^−1^ at *n* = 5. In fact, water is an exception when determining the optimal cluster size from the plateau of the gas‐phase clustering energies. The unique properties of water, stemming from its compact size and robust hydrogen bond network, may be the reason for the discrepancy in its solvation energies compared to the other solvents, requiring separate rules to determine its ideal cluster size. Excluding both water and MeFo from the data set improves the MAEs of all respective methods notably, which is summarized in Table 7. These MAE values still incorporate all weighted proton solvation energies, even those with non‐ideal values of *n*.

Astonishingly, BP86/def2‐TZVPP calculations gave the best overall results with the purely theoretical monomer cycle without experimental evaporation energies. This is remarkable considering the immense computational time of DLPNO‐CCSD(T)/CBS in contrast to BP86/def2‐TZVPP. Unfortunately, while the results may be acceptable, the reliance on random error correction makes this approach unreliable. Also, these results cannot be further improved because the gas‐phase energy error is necessary to optimize the performance of the monomer cycle. However, as mentioned above, the comparison of the MAE values is only an approximation, since the number of data points used in the mean absolute errors differs between methods. In addition, there is considerable variation in the individual solvation energies resulting from BP86/def2‐TZVPP that is not reflected in the final MAE for each method, as improvements with increasing *n* are not consistent across all solvents. The inability to determine the optimal *n* value for this method is also a major drawback, as it requires experimental data for comparison and prevents prediction of proton solvation energies without prior experiments.

The DLPNO‐CCSD(T)/CBS single‐point calculations significantly underestimate the solvation energies in all solvents except water, unless the calculated self‐solvation energy is replaced by experimental evaporation Gibbs’ energies. In this case, the results agree with the experimental data for methanol, ethanol, DMSO, DMF, acetonitrile, and propylene carbonate. The weak, largely non‐covalent interactions between solvent molecules are difficult to be captured in a continuum model of an isolated molecule. Experimental evaporation energies Δ_vap_ *G*° appear to provide a more accurate description of this aspect. Simply put, the self‐solvation energy contributes as a positive term to the proton solvation energy calculation (see (Equation 11)), and the evaporation energy is typically smaller than the calculated self‐solvation energy, which makes the resulting proton solvation energy more negative. Also, the CPCM error is larger for smaller solutes, an error that is multiplied by *n* with each iteration of the thermodynamic cycle.

#### 3.12.3 Evaluation of the Cluster Cycle

While the cluster cycle improves the results for all solvents, the issues outlined for water and methyl formate persist. Analogous to Table 6, Table 8 shows the Boltzmann‐weighted proton solvation energies obtained from the cluster cycle and the DSD‐BLYP/def2‐TZVPP optimizations followed by a DLPNO‐CCSD(T)/CBS single‐point calculation. The proton solvation energies calculated with both the double‐hybrid and its subsequent coupled cluster single‐point cancellation improve notably with the cluster cycle. The error cancelation when using a cluster on both sides of the cycle improves the solvation energies compared to the monomer cycle. Although methyl formate improves with the cluster cycle, it still has a significant error, a case investigated in the next section.

**TABLE 8:** Boltzmann‐weighted standard solvation energies of the proton Δ_solv_ *G*°(H^+^, S) in kJ mol^−1^ in water, MeCN, DMF, DMSO, EtOH, MeOH, MeFo, and PC, sorted in ascending order by the solvent number *n*.

Method	*n*	WA	MeOH	EtOH	DMF	DMSO	PC	MeCN	MeFo
DSD‐BLYP/def2‐TZVPP	2	−1060.1	−1074.6	−1079.9	−1130.8	−1137.6	−1057.2	−1043.1	−1042.8
	3	−1092.5	−1100.6	−1099.9	**−1132.8**	**−1140.0**	**−1061.0**	**−1043.4**	**−1045.1**
	4	−1098.3	−1102.9	−1106.5	**−1145.2**	**−1140.1**	**−1061.1**	**−1054.1**	**−1045.** **1**
	5	−1114.2	**−1103.0**	**−1107.6**	—	—	—	—	—
	6	**−1129.3**	**−1110.6**	**−1108.** **2**	—	—	—	—	—
	7	**−1136.0**	—	—	—	—	—	—	—
DLPNO‐CCSD(T)/CBS	2	−1048.0	−1064.6	−1069.7	−1126.3	−1124.0	−1050.2	−1035.6	−1035.8
	3	−1081.4	−1087.9	−1091.7	**−1126.3**	**−1126.5**	**−1054.5**	**−1035.9**	**−1038.5**
	4	−1088.4	−1090.0	−1096.8	**−1140.1**	**−1126.6**	b	**−1046.8**	**−1038.5**
	5	−1102.0	**−1090.7**	**−1097** **.6**	—	—	—	—	—
	6	**−1118.3**	**−1100.5**	**−1097.9**	—	—	—	—	—
	7	**−1122.0**	—	—	—	—	—	—	—
**Literature** a	**—**	**−1104.5**	**−1096.8**	**−1097.8**	**−1122.5**	**−1123.9**	**−1054.5**	**−1056.0**	**−1071.2**

a Solvation Energies obtained through Δ_solv_ *G*°(H^+^, S) = −1104.5 [27] + Δ_trans_ *G*°(H^+^, H_2_O → S) as summarized in Table 5.

b A CPCM calculation of the protonated and unprotonated PC cluster with *n* = 4 was not feasible at the DLPNO‐CCSD(T)/CBS level of theory.

*Note:* All energies were calculated with the cluster cycle and DSD‐BLYP/def2‐TZVPP as well as a DLPNO‐CCSD(T)/CBS single‐point calculation on the optimized DSD‐BLYP/def2‐TZVPP structure. Estimates of the optimal values of *n*, derived from the gas‐phase energies in section B.2 are highlighted in bold for each solvent.

##### 3.12.3.1 Methyl Formate as a Problem Case

**FIGURE 20:**
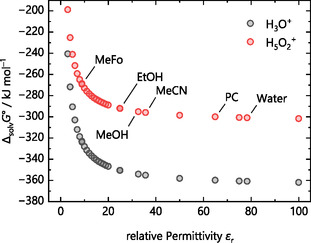
Standard Gibbs solvation energies Δ_solv_ *G*° for the H_3_O^+^ and H_5_O_2_^+^ ions at the DSD‐BLYP/def2‐TZVPP level of theory obtained with the CPCM model and different relative permittivities from 3 to 100, with the position of some solvents highlighted.



Despite following the above‐described trends for aprotic solvents, the proton solvation energies in methyl formate significantly differ from those measured with the ILSB method, regardless of the method or the cycle. It should be emphasized that there is just one experimental measurement for methyl formate determined by the ILSB setup, and there is no published value to compare to. Due to its low relative permittivity of 8.84 [48], it is significantly less polar than the other solvents and could possibly exceed the scope of the cluster continuum model. The CPCM method is more accurate at high permittivities, as the medium approaches the behavior of an ideal conductor when the dielectric constant is infinitely high. However, cluster continuum models are not constrained by a fixed permittivity limit, and their performance can be affected by various method‐specific elements and properties of the system under study. To shed light on this relationship, Figure 20 plots the calculated solvation Gibbs energies of H_3_O^+^ and H_5_O_2_^+^ at the DSD‐BLYP/def2‐TZVPP level of theory for different relative permittivities ranging from 3 to 100. The Born equation ((Equation 18)).

(18)

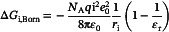




describes the relationship between solvation energy and dielectric constant, visualized in this graph, but it is important to remember that the CPCM model is more complex than the Born model. As the relative permittivity decreases, so does the solvation energy, but the decrease becomes increasingly more pronounced: Hence, the solvation Gibbs energy of H_5_O_2_^+^ changes only by 13 kJ mol^−1^ as the permittivity decreases from 100 to 20, but it changes by 90 kJ mol^−1^ when it decreases from 20 to 3.

Uncertainties are less apparent in the plateau region where the solvation energies remain robust to small changes in the relative permittivity, but for small relative permittivities, little variations can lead to extreme changes in the solvation energies and amplify small uncertainties. Interestingly, increasing the permittivity exemplarily from 8.84 to 9.84 in the MeFo‐calculations resulted in only minimal changes of the calculated solvation energies. The solvation energy increased steadily by 2 kJ mol^−1^ for each isomer compared to the calculations with *ε*_r_ = 8.84 (Supporting Information, Section 3.4). Thus, the underestimation of the proton's solvation energy in methyl formate is unlikely to be attributed to an uncertainty in the experimental relative permittivity value of methyl formate only. Thus, it appears likely that the cluster continuum model for the proton may encounter limitations in the range between the permittivity of EtOH, which provides satisfactory results, and the permittivity of MeFo, at least within the specific approach used in this study.

##### 3.12.3.2 MAE Errors

While both calculation methods in Table 8 substantially improve the proton solvation in water, they still overestimate the solvation energy for *n* = 6–7, which would be considered the optimal values according to B.2. Yet, when both water and methyl formate are once again excluded, the MAE for all weighted solvation energies of the remaining solvents is 10.5 kJ mol^−1^ for DSD‐BLYP/def2‐TZVPP and 8.5 kJ mol^−1^ for coupled cluster. The DLPNO‐CCSD(T)/CBS error is further reduced to 6.0 kJ mol^−1^ when considering only the optimal values of *n*, namely 3–4 for aprotic solvents and 5–6 for protic solvents. By contrast, the proton solvation energies in methanol, ethanol, dimethyl sulfoxide, and propylene carbonate even reach chemical accuracy. While computationally very expensive, DLPNO‐CCSD(T)/CBS with cluster cycle provided the most accurate results among those tested.

Acetonitrile works well enough with the double‐hybrid but shows a slightly larger deviation from the experiment with the DLPNO‐CCSD(T)/CBS calculations. Unlike the other solvents, which are primarily linked by O‐H^+^…O and C‐H…O hydrogen bonds, both protonated and neutral acetonitrile clusters displayed distinct structures with N–H^+^…N and C‐H…N bonds and additional N^δ−^…C^δ+^ interactions (B.2; Figure 13). These additional interactions and its nitrogen‐donor character could contribute to its proton solvation energy having a slightly higher deviation than in the other aprotic solvents. An error in the hydrogen bond lengths would affect the size of the cavity and thus the solvation energies of the clusters. Since the other solvents contain an oxygen donor atom, more consistent errors from the method might be expected, but this is uncertain.

The cluster cycle is more susceptible to uncertainties arising from noncovalent bonding because it requires both protonated and neutral clusters. This includes possible effects from the basis set. In contrast to the protonated solvent clusters, which work well with the triple‐ζ basis set def2‐TZVPP, the structures of the neutral clusters could benefit from a basis set with additional diffuse functions, which could improve the proton solvation energies obtained from the cluster cycle. However, identifying potential sources of error related to gas‐phase energies, dispersion, or solvation energies in the clusters is challenging when comparing only the proton solvation energies. Likewise, improving only one aspect, such as the dispersion, may not automatically improve the overall results, as all contributions within the cycle must work effectively for meaningful outcomes.

It is also noteworthy that the different isomers can have unpredictable effects on the final proton solvation energies and explain the irregularities described above, such as the sudden increase in the proton solvation energy of the DMF cluster at *n* = 4 or the variation from *n* = 2–3 to *n* = 4 in acetonitrile. This is also true for the monomer cycle, although this cycle typically shows more significant deviations.

#### 3.12.4 Performance of the Cluster Continuum Model

In agreement with the benchmark calculations of the gas‐phase energies, DLPNO‐CCSD(T)/CBS single‐point calculations using optimized DSD‐BLYP/def2‐TZVPP structures gave the best overall performance. This method was successful not only for the cluster cycle but also for the monomer cycle, when the self‐solvation energy was replaced by the experimentally determined evaporation energy. The agreement between calculated and experimental proton solvation energies suggests that the DSD‐BLYP/def2‐TZVPP level of theory accurately models the structures. The significant improvement in the proton solvation energies from the monomer cycle, when the experimental evaporation energies are included, suggests compensating errors in the gas‐phase clustering energy and the solvation energies of the protonated clusters. Typically, the solvation energies and the gas‐phase clustering energies show opposite trends with respect to the accuracy of the calculations. The solvation energies of charged systems tend to improve with increasing system size, as the impact of the first solvation shell effects decreases (see above). Conversely, the gas‐phase energy calculations show optimal results for the smallest cluster size. This is particularly evident in the calculated proton solvation energies for the overall better performing cycles with *n* = 1, which differ from the experimental values by roughly 40–60 kJ mol^−1^, although the gas‐phase energies remain highly accurate. However, in contrast to the gas‐phase energies, where chemical accuracy is achieved at least for *n* = 1, as shown in section B.1, assessing the accuracy of the solvent clusters proves to be more challenging. Only the final proton solvation energy allows comparison with experimental data, but the thermodynamic cycle encompasses other contributions and, ideally, beneficial error compensation. In essence, an optimal value of *n* corresponds to the point of maximum error reduction. This work revealed only two distinct trends in the explicit solvent molecule counts, classifying solvents as aprotic or protic, with the latter category requiring a greater number of solvent molecules for ideal error compensation. More trends are likely to exist, as seen with water, which deviates from the trend established by methanol and ethanol for the protic solvents. This trend is observed in both monomer and cluster cycles, except for the simple BP86/def2‐TZVPP method. While the monomer cycle results without experimental evaporation energies differ substantially from the experimental energies, the optimal *n* value derived from the gas‐phase still produces the best results.

Table 9 summarizes the weighted standard solvation energies of the proton calculated using DLPNO‐CCSD(T)/CBS with both cycles and the optimal values of *n*, excluding methyl formate. Since the estimation of *n* from the gas‐phase clustering energies was unsuccessful, the optimal value given for water was revised to match the experimental values as closely as possible, with *n* = 4 in the monomer cycle and *n* = 5 in the cluster cycle.

**TABLE 9:** Boltzmann‐weighted “best” standard solvation Gibbs energies of the proton Δ_solv_ *G*°(H^+^, S) in water, MeCN, DMF, DMSO, EtOH, MeOH, and PC, for the optimal solvent number *n* obtained from the DLPNO‐CCSD(T)/CBS single‐point calculation on optimized DSD‐BLYP/def2‐TZVPP structures with the monomer cycle with the experimental energy of evaporation and the cluster cycle.

Method	*n*	WA	MeOH	EtOH	*n*	DMF	DMSO	PC	MeCN
Monomer cycle (vap)	**4/5**	−1105.6	−1091.6	−1081.5	**3**	−1124.0	−1127.9	−1044.1	−1048.6
	**5/6**	−1121.9	−1100.2	−1081.5	**4**	−1124.0	−1127.9	—	−1049.5
Cluster cycle	**5**	−1102.0	−1090.7	−1097.6	**3**	−1126.3	−1126.5	−1054.5	−1035.9
	**6**	−1118.3	−1100.5	−1097.9	**4**	−1140.1	−1126.6	—	−1046.8
**Literature** a	**Exp.**	**−1104.5**	**−1096.8**	**−1097.8**		**−1122.5**	**−1123.9**	**−1054.5**	**−1056.0**
Kelly et al. [93].	—	—	−1094.5	—	—	—	−1135.5	—	−1080.7
Himmel et al. [2].	—	—	—	—	—	—	−1120	—	−1056
Rossini et al. [94, 95].	—	—	−1112.5	—	—	—	−1114.6	—	−1067.3

a Solvation Energies obtained through Δ_solv_ *G*°(H^+^, S) = −1104.5 [27] + Δ_trans_ *G*°(H^+^, H_2_O → S) as summarized in Table 5. A CPCM calculation of the protonated and unprotonated PC cluster with *n* = 4 was not feasible at the DLPNO‐CCSD(T)/CBS level of theory.

When methyl formate and water are excluded, the monomer and cluster cycles give the most accurate results, with mean absolute errors of 7.0 kJ mol^−1^ and 6.0 kJ mol^−1^, respectively. These results fall comfortably within the margin of the error of the Tissandier value for the proton hydration Gibbs energy and the additional uncertainties introduced by the TATB and/or the ILSB assumptions.

The cluster cycle outperforms the monomer cycle with experimental evaporation energies for both DSD‐BLYP/def2‐TZVPP and DLPNO‐CCSD(T)/CBS single‐point calculations. Compared to DLPNO‐CCSD(T)/CBS, the DSD‐BLYP/def2‐TZVPP method exhibits slightly lower accuracy and overestimates proton solvation energies for most solvents and both cycles. DSD‐BLYP/def2‐TZVPP has higher gas‐phase clustering energies than the coupled cluster benchmark and performs slightly better with a smaller number of explicit solvent molecules than suggested by the gas‐phase clustering energy. Compared to the optimal *n* estimated from the gas‐phase energies, the double‐hybrid performs slightly better with *n* = 2–3 for the aprotic solvents and *n* = 4–5 for the protic solvents, although the estimate remains good for most of the solvents and the improvement with a smaller *n* is negligible. There is a shift to lower values of *n*, which is related to the error of the gas‐phase energies with respect to the coupled cluster benchmark. The higher the uncertainty in the gas‐phase energy, the lower the number of explicit solvent molecules required for the cluster continuum model.

With BP86/def2‐TZVPP, *n* = 2 is the cluster size that determines the final proton solvation energy, which is beneficial because it reduces the number of possible isomers and eliminates the problems associated with larger values of *n* described above. However, this conclusion is based on a comparison with the experimental data. An accurate determination of the ideal cluster size without relying on experimental energies is essential, especially for systems where experimental measurements are challenging. Adding more solvent molecules does not improve the accuracy of the calculated energies for BP86 and may even decrease it. The weighted proton solvation energies in the aprotic solvents remain unchanged when more than two explicit solvent molecules are added, while the protic solvents show a slight improvement with a third molecule. This indicates that the best *n* values are two for aprotic solvents and three for protic solvents. When evaporation energies are incorporated, the use of more than two explicit solvent molecules reduces the accuracy for all solvents. Although the BP86/def2‐TZVPP method yields satisfactory results with reduced computational cost, inherent limitations arise from its dependence on inaccurate gas‐phase energy computations to achieve error compensation.

Even with highly accurate methods such as coupled cluster, the estimation of *n* from the gas‐phase energies is only approximate and, as seen for water, does not always hold once all the contributions of the thermodynamic cycle click into place. Water is an exceptional case among the solvents in this work, and the continuum model is a very simplified view of a solvent. Since this method approximates the solvent as a continuum, which is virtually the same for similar relative permittivities, it requires a similar set of solvents, without exceptional non‐covalent interactions and overall similar relative permittivities. Water molecules are small compared to other solvents, and their interactions are unusually strong. Conversely, methyl formate has a very low relative permittivity, suggesting a permittivity threshold for the cluster continuum model, at least in the specific way it was applied in this study. This conclusion depends, of course, on the accuracy of the experimental value, which is currently based on just one measurement. Based on additional experimental or theoretical findings, limiting cluster continuum models to similar solvents may be advantageous. An alternative method involves categorizing solvents and modifying settings according to these categories, mirroring the treatment of protic and aprotic solvents in the present work. Otherwise, it becomes difficult to find outliers, especially without experimental reference data for comparison.

##### 3.12.4.1 Comparison to Error Bars and Other Determinations

Unfortunately, the experimental reference data, derived from extra‐thermodynamic assumptions, provides only estimated errors for each method, but individual measurements may exhibit greater uncertainties. Furthermore, as used in this context, all values incorporate Tissandier's proton hydration energy and its error bar of ±8 kJ mol^−1^ [28]. Despite the DLPNO‐CCSD(T)/CBS method's excellent performance relative to experimental energies, the inherent uncertainty associated with the experiment unfortunately complicates the comparison. The results from both theoretical and composite methods show considerable variation, with a subset presented in Table 9. Hence, Kelly et al. used a cluster pair approximation, which is the best approach for determining the proton hydration energy, to obtain the standard proton solvation energy Δ_solv_ *G*°(H^+^, S) in methanol, acetonitrile, and DMSO, resulting in −1094.5, −1080.7, and −1135.5 kJ mol^−1^, respectively [93]. Theoretical calculations using different models have also produced a range of different Gibbs energies, including −1056 kJ mol^−1^ and −1120 kJ mol^−1^ calculated with the rCCC model for acetonitrile and DMSO, respectively [2]. Proton solvation energies of −1067.3, −1112.5, and −1114.6 kJ mol^−1^ for acetonitrile, methanol, and DMSO, respectively, were derived from computed and measured solvent p*K*_a_ values [94, 95].

## 4 Conclusion

### 4.1 Experimental Work

The results of the experimental work in terms of Gibbs solvation energies are collected in Table 10, with Δ_solv_ *G*°(H^+^, H_2_O) = −1104.5 kJ mol^−1^ as the anchor [27]. Also included are the corresponding values of the hydrogen electrode and pH values at standard conditions (i.e., relative activity *a* = 1) with respect to the ideal electron gas and proton gas at 298.15 K and 10^5^ Pa, and aligned to the unified aqueous scales $E_{\mathrm{abs}}^{H_{2}O}$ and ${\mathrm{pH}}_{\mathrm{abs}}^{H_{2}O}$, the values of which are directly comparable with each other [3, 24, 96]. Note that these two values are not subject to the uncertainty of ±8 kJ mol^−1^, as per definition the Gibbs hydration energy is subtracted.

**TABLE 10:** Gibbs solvation energies of the proton Δ_solv_ *G*°(H^+^, S), the unified acidity ${\mathrm{pH}}_{\mathrm{abs}}^{H_{2}O}{}^{\circ}$, and the unified hydrogen electrode potential $E_{\mathrm{abs}}^{H_{2}O}{}^{\circ}$, both at standard conditions in the solvents investigated (mol L^−1^ scale).

S	Δ_solv_ *G*°(H^+^, S) / kJ mol^−1^	Δ_tr_ *G*°(H^+^, H_2_O→ S) / kJ mol^−1^	${\mathrm{pH}}_{\mathrm{abs}}^{H_{2}O}{}^{\circ}$	$E_{\mathrm{abs}}^{H_{2}O}{}^{\circ}$ / V
DMSO	−1123.9^b^	−19.4	3.4	−0.201
DMF	−1122.5^b^	−18	3.2	−0.187
H_2_O	−1104.5^a^	0.0	0.0	0.000
EtOH	−1097.8^c^	6.7	−1.2	0.070
MeOH	−1096.9^c^	7.7	−1.3	0.079
MeFo	−1071.2^c^	33.3	−5.8	0.345
MeCN	−1056.0^c^	48.5	−8.5	0.503
PC	−1054.5^b^	50	−8.8	0.518

a Ref [27].

b Obtained with the value for H_2_O from Ref [27] and Δ_tr_ *G*°(H^+^, H_2_O→ S) TATB values from from Ref [40].

c Obtained with the value for H_2_O from Ref [27] and Δ_tr_ *G*°(H^+^, H_2_O→ S) values of Table 1 in this work.

This nicely quantifies the different solvating properties of the solvents for the proton. Hence, the proton is 10^8.5^ times more acidic at standard conditions in MeCN than in water. Yet, they all suffer from the limitations of including the extra‐thermodynamic ILSB‐assumption.

### 4.2 Computations

All efforts to construct cycles with small error bars to assess the single ion magnitude Δ_tr_ *G*°(H^+^, g → S) and hence, to overcome the principle extra‐thermodynamic assumptions necessary due to electroneutrality reasons in condensed phases, were left with error bars of 10–15 kJ mol^–1^, even when used with the most sophisticated methods: Double hybrid based structures followed by single point calculations approximating the complete basis set limit at the coupled cluster CCSD(T) level of theory, both for gas phase as well as under solvation with the CPCM model. The best results were obtained with the cluster cycle and DLPNO‐CCSD(T)/CBS, which came close to the measured ILSB values and the TATB energies. However, there were a few exceptions, such as acetonitrile, which was better described by the DSD‐BLYP functional, and methyl formate, which has a low relative permittivity and appears to be beyond the scope of the cluster continuum model.

Overarching the evaluation of the resulting Gibbs energies is the problem of structure. From all phases, the true structures of solvated species are the least solidly known. Rather, the knowledge of a solvated structure with a quality similar to a solid‐state structure determination (± 1 pm), or a good electron diffraction structure in the gas phase (± 1 pm) is to be wished for the solution state. To the best of our knowledge, rigid solutes might be investigated with multinuclear NMR‐spectroscopy and error bars rather in the range of ± 10 pm but not in a regime needed for benchmarking of the computational methods (± 1 pm). In addition, the protonated species of interest here are rather dynamic on the time scale of NMR spectroscopy and further complicate matters. Hence, we are rather blind if the structures optimized with the typical continuum models really reflect those that are relevant in the framework of the Born‐Oppenheimer treatment. Hence, it is not clear whether the calculations really reflect a close approximation of those needed to reach the goal of chemical accuracy by applying the coupled cluster benchmark method in the solvated state. Rather, the error is enlarged at least by a factor of 3. An alternative could be that the Born‐Oppenheimer Approximation is not suitable to describe the highly dynamic protonated solvent clusters, and the acidic proton should better be described as a proton wave.

Hence, this investigation calls for two aspects: The need for precise experimental structure elucidations of the species relevant for the solvation of protons (or other electroactive ions) to serve as benchmarks for the performance of the computational models to calculate the chemical potential of these ions in their solvent environment. And for fundamental refinements of the computational models. As we have seen above, apparently, the accurate assessment of effects like basis set superposition error, size consistency, dispersive interactions, as well as the overarching need to correctly describe entropic contributions to the chemical potential still presents challenges, especially to be tackled in a solvent environment.

We invite experimentalists and theorists alike to embark on this journey. The “ideal” ILSB values collected above may present some thermodynamic guidelines to judge the quality of the structures derived.

## Supporting Information

Additional supporting information can be found online in the Supporting Information section.

## Funding

This work was supported by the European Research Council (101052935); Deutsche Forschungsgemeinschaft (KR2046/36−1, INST 40/575−1 FUGG).

## Conflicts of Interest

The authors declare no conflicts of interest.

## Supporting information

Supplementary Material

## Data Availability

The data that support the findings of this study are available in the supplementary material of this article.

## Endnotes

1 The gas‐phase energies calculated in this study are at 1 atm (1.013 bar), so the values are adjusted for a transition from 1.013 bar to 1 mol L^−1^. The Gibbs energy required for the transition of an ideal gas from 1 mol L^−1^ to 1 atm (24.79 mol L^−1^) at 298.15 K is 7.96 kJ mol^−1^. The concentration required to convert the solvation energy to a state of 1 bar in the gas‐phase and 1 mol L^−1^ in solution is 24.46 mol L^−1^, giving a factor of 7.90 kJ mol^−1^. This is a difference of only 0.06 kJ mol^−1^, which is negligible compared to the error in continuum solvation energies, especially for single‐ions.

2 Most of the settings remained unchanged, but a few modifications were necessary for specific functionals, which are detailed in the Supporting Information Section 2.5. These changes do not affect the overall comparison of the performance of the different functionals.

3 This also indicates that an extensive exploration of higher energy conformations is not needed, if the global minimum or a nearby geometry was identified. Also, similar to cluster solvation energies, proton solvation energies for the same value of n are very close.

4 However, the structure assigned in solution is only qualitative and lacks any accurate experimentally determined structural details. Yet, it suggests that the description of the bonding situation of the H_13_O_6_^+^ (1) isomer is as complex in the gas‐phase as it is in solution and, since no experimental benchmark structures exist, could benefit from an optimization with coupled cluster methods. In addition, Figure S15/SI Section 2.7 shows, that the optimized BP/def2‐TZVPP gas‐phase structures of the protonated water clusters H(H_2_O)_*n*_^+^ show only minor changes of ≈ 1 pm compared to the double‐hybrid structures, except for the H_13_O_6_^+^ (1) isomer. As expected, the bond lengths from the BP/def2‐TZVPP calculation are slightly longer than those from the more advanced double‐hybrid functional.

## References

[1] D. Himmel , S. K. Goll , I. Leito , and I. Krossing , “A Unified pH Scale for All Phases”, Angewandte Chemie, International Edition 49 (2010): 6885. 20715223 10.1002/anie.201000252

[2] D. Himmel , S. K. Goll , I. Leito , and I. Krossing , “Anchor Points for the Unified Brønsted Acidity Scale: The rCCC Model for the Calculation of Standard Gibbs Energies of Proton Solvation in Eleven Representative Liquid Media,” Chemistry – A European Journal 17 (2011): 5808. 21542031 10.1002/chem.201003164

[3] V. Radtke , D. Himmel , K. Pütz , S. K. Goll , and I. Krossing , “The Protoelectric Potential Map (PPM): An Absolute Two‐Dimensional Chemical Potential Scale for a Global Understanding of Chemistry,” Chemistry – A European Journal 20 (2014): 4194. 24615801 10.1002/chem.201302473

[4] M. Sastre and J. A. Santaballa , “A Note on the Meaning of the Electroneutrality Condition for Solutions,” Journal of Chemical Education 66 (1989): 403.

[5] P. Hünenberger and M. Reif , Single‐Ion Solvation. Experimental and Theoretical Approaches to Elusive Thermodynamic Quantities (RSC Publishing, 2011).

[6] J. F. Coetzee and W. R. Sharpe , “Solute‐Solvent Interactions. VI. Specific Interactions of Tetraphenylarsonium, Tetraphenylphosphonium, and Tetraphenylborate Ions with Water and Other Solvents,” The Journal of Physical Chemistry 75 (1971): 3141.

[7] W. R. Fawcett , “Acidity and Basicity Scales for Polar Solvents,” The Journal of Physical Chemistry 97 (1993): 9540.

[8] Y. Shao , A. A. Stewart , and H. H. Girault , “Determination of the Half‐Wave Potential of the Species Limiting the Potential Window. Measurement of Gibbs Transfer Energies at the Water/1,2‐Dichloroethane Interface,” Journal of the Chemical Society, Faraday Transactions 87 (1991): 2593.

[9] J. Stangret and E. Kamienska‐Piotrowicz , “Effect of tetraphenylphosphonium and tetraphenylborate ions on the water structure in aqueous solutions; FTIR studies of HDO spectra,” Journal of the Chemical Society, Faraday Transactions 93 (1997): 3463.

[10] V. Luzhkov and A. Warshel , “Microscopic Models for Quantum Mechanical Calculations of Chemical Processes in Solutions: LD/AMPAC and SCAAS/AMPAC Calculations of Solvation Energies,” Journal of Computational Chemistry 13 (1992): 199.

[11] R. Schurhammer and G. Wipff , “About the TATB Assumption: Effect of Charge Reversal on Transfer of Large Spherical Ions from Aqueous to Non‐Aqueous Solvents and on Their Interfacial Behaviour,” Journal of Molecular Structure: THEOCHEM 500 (2000): 139.

[12] R. Scheu , B. M. Rankin , Y. Chen , K. C. Jena , D. Ben‐Amotz , and S. Roke , “Charge Asymmetry at Aqueous Hydrophobic Interfaces and Hydration Shells,” Angewandte Chemie, International Edition 53 (2014): 9560. 25045022 10.1002/anie.201310266

[13] L. Onsager , “Reciprocal Relations in Irreversible Processes. I.,” Physical Review 37 (1931): 405.

[14] R. Alexander , A. J. Parker , J. H. Sharp , and W. E. Waghorne , “Solvation of Ions. XVI. Solvent Activity Coefficients of Single Ions. Recommended Extrathermodynamic Assumption,” Journal of the American Chemical Society 94 (1972): 1148.

[15] T. Kakiuchi and T. Yoshimatsu , “A new salt bridge based on the hydrophobic room‐temperature molten salt,” Bulletin of the Chemical Society of Japan 79 (2006): 1017.

[16] T. Kakiuchi , N. Tsujioka , S. Kurita , and Y. Iwami , “Phase‐boundary potential across the nonpolarized interface between the room‐temperature molten salt and water,” Electrochemistry Communications 5 (2003): 159.

[17] Y. Fujino and T. Kakiuchi , “Ionic liquid salt bridge based on N‐alkyl‐N‐methylpyrrolidinium bis (pentafluoroethanesulfonyl) amide for low ionic strength aqueous solutions,” Journal of Electroanalytical Chemistry 651 (2011): 61.

[18] H. Sakaida , Y. Kitazumi , and T. Kakiuchi , “Ionic Liquid Salt Bridge Based On Tributyl (2‐methoxyethyl) Phosphonium Bis (Pentafluoroethanesulfonyl) Amide For Stable Liquid Junction Potentials In Highly Diluted Aqueous Electrolyte Solutions,” Talanta 83 (2010): 663. 21111189 10.1016/j.talanta.2010.10.024

[19] T. Yoshimatsu and T. Kakiuchi , “Ionic liquid salt bridge in dilute aqueous solutions,” Analytical Sciences 23 (2007): 1049. 17878576 10.2116/analsci.23.1049

[20] T. Kakiuchi and M. Yamamoto , “Current stage and perspectives of pH measurements by use of ionic liquid salt bridge,” Bunseki Kagaku 65 (2016): 181.

[21] T. Kakiuchi , “Ionic liquid salt bridge—Current stage and perspectives: A mini review,” Electrochemistry Communications 45 (2014): 37.

[22] V. Radtke , A. Ermantraut , D. Himmel , T. Koslowski , I. Leito , and I. Krossing , “The Ideal Ionic Liquid Salt Bridge for the Direct Determination of Gibbs Energies of Transfer of Single Ions, Part I: The Concept,” Angewandte Chemie International Edition 57 (2018): 2344. 29235713 10.1002/anie.201707333

[23] A. Ermantraut , V. Radtke , N. Gebel , et al., “The Ideal Ionic Liquid Salt Bridge for Direct Determination of Gibbs Energies of Transfer of Single Ions, Part II: Evaluation of the Role of Ion Solvation and Ion Mobilities,” Angewandte Chemie International Edition 57 (2018): 2348. 29235721 10.1002/anie.201707334

[24] V. Radtke , N. Gebel , D. Priester , et al., “Measurements and Utilization of Consistent Gibbs Energies of Transfer of Single Ions: Towards a Unified Redox Potential Scale for All Solvents,” Chemistry ‐ A European Journal 28 (2022): e202200509. 35446995 10.1002/chem.202200509 PMC9401597

[25] V. Radtke , D. Priester , A. Heering , et al., “The Unified Redox Scale for All Solvents: Consistency and Gibbs Transfer Energies of Electrolytes from Their Constituent Single Ions,” Chemistry ‐ A European Journal 29 (2023): e202300609. 37191477 10.1002/chem.202300609

[26] This not necessarily includes the assumption that Δ_tr_ *G*°([N_2225_]^+^) = Δ_tr_ *G*°([TA]^+^) .

[27] M. D. Tissandier , K. A. Cowen , W. Y. Feng , et al., “The Proton's Absolute Aqueous Enthalpy and Gibbs Free Energy of Solvation from Cluster‐Ion Solvation Data,” The Journal of Physical Chemistry A 102 (1998): 7787.

[28] C. P. Kelly , C. J. Cramer , and D. G. Truhlar , “Aqueous Solvation Free Energies of Ions and Ion−Water Clusters Based on an Accurate Value for the Absolute Aqueous Solvation Free Energy of the Proton,” The Journal of Physical Chemistry B 110 (2006): 16066. 16898764 10.1021/jp063552y

[29] D. M. Camaioni and C. A. Schwerdtfeger , “Comment on “Accurate Experimental Values for the Free Energies of Hydration of H^+^, OH^‐^, and H_3_O^+^ ”,” The Journal of Physical Chemistry A 109 (2005): 10795. 16863129 10.1021/jp054088k

[30] A. Kraft , J. Possart , H. Scherer , J. Beck , D. Himmel , and I. Krossing , “The Al (ORF) 3/H2O/Phosphane [RF= C (CF3) 3] System–Protonation of Phosphanes and Absolute Brønsted Acidity.,” European Journal of Inorganic Chemistry 2013 (2013): 3054.

[31] D. Himmel , S. K. Goll , I. Leito , and I. Krossing , “Bulk Gas‐Phase Acidity,” Chemistry ‐ A European Journal 18 (2012): 9333. 22730176 10.1002/chem.201104025

[32] D. Himmel , S. K. Goll , F. Scholz , V. Radtke , I. Leito , and I. Krossing , “Absolute Brønsted acidities and pH scales in ionic liquids,” Chemphyschem : A European Journal of Chemical Physics and Physical Chemistry 16 (2015): 1428. 25853921 10.1002/cphc.201402906

[33] F. Scholz , D. Himmel , L. Eisele , et al., “The acidity of the HBr/AlBr3 system: stabilization of crystalline protonated arenes and their acidity in bromoaluminate ionic liquids,” Chemistry ‐ A European Journal 21 (2015): 7489. 25808398 10.1002/chem.201405952

[34] D. Himmel , R. J. White , E. Jacob , and I. Krossing , “Highly correlated ab initio thermodynamics of oxymethylene dimethyl ethers (OME): formation and extension to the liquid phase,” Sustainable Energy Fuels 1 (2017): 1177.

[35] E. Paenurk , K. Kaupmees , D. Himmel , et al., “A unified view to Brønsted acidity scales: do we need solvated protons?,” Chemical Science 8 (2017): 6964. 29147523 10.1039/c7sc01424d PMC5642146

[36] V. Radtke , K. Pütz , D. Himmel , and I. Krossing , “The Inverted Philosopher's Stone: how to turn silver to a base metal,” Journal of Solid State Electrochemistry 24 (2020): 2847.

[37] J. F. Kögel , T. Linder , F. G. Schröder , et al., “Fluoro‐ and Perfluoralkylsulfonylpentafluoroanilides: Synthesis and Characterization of NH Acids for Weakly Coordinating Anions and Their Gas‐Phase and Solution Acidities,” Chemistry ‐ A European Journal 21 (2015): 5769. 25727401 10.1002/chem.201405391

[38] V. S. Bryantsev , M. S. Diallo , and W. A. Goddard III , “Calculation of Solvation Free Energies of Charged Solutes Using Mixed Cluster/Continuum Models,” The Journal of Physical Chemistry B 112 (2008): 9709. 18646800 10.1021/jp802665d

[39] C. P. Kelly , C. J. Cramer , and D. G. Truhlar , “SM6: A Density Functional Theory Continuum Solvation Model for Calculating Aqueous Solvation Free Energies of Neutrals, Ions, and Solute−Water Clusters,” Journal of Chemical Theory and Computation 1 (2005): 1133. 26631657 10.1021/ct050164b

[40] Y. Marcus , “Thermodynamic functions of transfer of single ions from water to nonaqueous and mixed solvents: Part I‐Gibbs free energies of transfer to nonaqueous solvents,” Pure and Applied Chemistry 55 (1983): 977.

[41] I. M. Kolthoff and M. K. Chantooni , “Critical Study Involving Water, Methanol, Acetonitrile, N,N‐Dimethylformamide, and Dimethyl Sulfoxide of Medium Ion Activity Coefficients, .gamma., on the Basis of the .gamma.AsPh4+ = .gamma.BPh4‐ Assumption,” The Journal of Physical Chemistry 76 (1972): 2024.

[42] Y. Marcus , M. J. Kamlet , and R. W. Taft , “Linear Solvation Energy Relationships: Standard Molar Gibbs Free Energies and Enthalpies of Transfer of Ions from Water into Nonaqueous Solvents,” The Journal of Physical Chemistry 92 (1988): 3613.

[43] CRC handbook of Chemistry and Physics, (CRC Press, 2014).

[44] I. M. Kolthoff and F. G. Thomas , “Electrode Potentials in Acetonitrile. Estimation of the Liquid Junction Potential between Acetonitrile Solutions and the Aqueous Saturated Calomel Electrode ^1^ ,” The Journal of Physical Chemistry 69 (1965): 3049.

[45] A. Macfarlane and H. Hartley , “XXXVI. Standard electrode potentials in ethyl alcohol,” Philosophical Magazine 13 (1932): 425.

[46] P. S. Buckley and H. Hartley , “XXXVIII. Preliminary determinations of standard electrode potentials in methyl alcohol.” Philosophical Magazine 8 (1929): 320.

[47] A. Macfarlane and H. Hartley , “XLVI. The standard electrode potential of lithium in methyl alcohol,” Philosophical Magazine 20 (1935): 611.

[48] P. Winget , D. M. Dolney , D. J. Giesen , C. J. Cramer , and D. G. Truhlar , “Minnesota Solvent Descriptor Database” Can Be Found Under, *Available at:* https://comp.chem.umn.edu/solvation/mnsddb.pdf.

[49] R. Naejus , C. Damas , D. Lemordant , R. Coudert , and P. Willmann , “Excess Thermodynamic Properties of the Ethylene Carbonate–trifluoroethyl Methyl Carbonate and Propylene Carbonate–trifluoroethyl Methyl Carbonate Systems at = (298.15 315.15) K,” The Journal of Chemical Thermodynamics 34 (2002): 795.

[50] J. P. Perdew , “Density‐functional approximation for the correlation energy of the inhomogeneous electron gas,” Physics Review. B 33 (1986): 8822. 10.1103/physrevb.33.8822 9938299

[51] J. P. Perdew , “Erratum: Density‐functional approximation for the correlation energy of the inhomogeneous electron gas,” Physics Review B 34 (1986): 7406. 9949100

[52] A. D. Becke , “Density‐Functional Exchange‐Energy Approximation with Correct Asymptotic Behavior,” Physics Review A 38 (1988): 3098. 10.1103/physreva.38.3098 9900728

[53] S. Kozuch , D. Gruzman , and J. M. L. Martin , “DSD‐BLYP: A General Purpose Double Hybrid Density Functional Including Spin Component Scaling and Dispersion Correction,” The Journal of Physical Chemistry C 114 (2010): 20801.

[54] F. Weigend and R. Ahlrichs , “Balanced Basis Sets of Split Valence, Triple Zeta Valence and Quadruple Zeta Valence Quality for H to Rn: Design and Assessment of Accuracy,” Physical Chemistry Chemical Physics 7 (2005): 3297. 16240044 10.1039/b508541a

[55] S. Grimme , J. Antony , S. Ehrlich , and H. Krieg , “A consistent and accurate ab initio parametrization of density functional dispersion correction (DFT‐D) for the 94 elements H‐Pu,” The Journal of Chemical Physics 132 (2010): 154104. 20423165 10.1063/1.3382344

[56] S. Grimme , S. Ehrlich , and L. Goerigk , “Effect of the damping function in dispersion corrected density functional theory,” Journal of Computational Chemistry 32 (2011): 1456. 21370243 10.1002/jcc.21759

[57] C. Riplinger and F. Neese , “An efficient and near linear scaling pair natural orbital based local coupled cluster method,” The Journal of Chemical Physics 138 (2013): 34106. 10.1063/1.4773581 23343267

[58] C. Riplinger , B. Sandhoefer , A. Hansen , and F. Neese , “Natural triple excitations in local coupled cluster calculations with pair natural orbitals,” The Journal of Chemical Physics 139 (2013): 134101. 24116546 10.1063/1.4821834

[59] T. H. Dunning , “Gaussian Basis Sets for use in Correlated Molecular Calculations. I. The Atoms Boron through Neon and Hydrogen,” The Journal of Chemical Physics 90 (1989): 1007.

[60] D. E. Woon and T. H. Dunning , “Gaussian Basis Sets for use in Correlated Molecular Calculations. III. The Atoms Aluminum through Argon,” The Journal of Chemical Physics 98 (1993): 1358.

[61] L. Goerigk , A. Hansen , C. Bauer , S. Ehrlich , A. Najibi , and S. Grimme , “A Look at the Density Functional Theory Zoo with the Advanced GMTKN55 Database for General Main Group Thermochemistry, Kinetics and Noncovalent Interactions,” Physical Chemistry Chemical Physics 19 (2017): 32184. 29110012 10.1039/c7cp04913g

[62] E. P. L. Hunter and S. G. Lias , “Evaluated Gas Phase Basicities and Proton Affinities of Molecules: An Update,” The Journal of Physical Chemistry . Ref. Data 27 (1998): 413.

[63] P. Schuster , G. Zundel , and C. Sandorfy , The Hydrogen Bond : Recent Developments in Theory and Experiments, North‐Holland Pub. Co.; Distributor, (American Elsevier Pub. Co, 1976).

[64] M. Eigen , “Proton Transfer, Acid‐Base Catalysis, and Enzymatic Hydrolysis. Part I: ELEMENTARY PROCESSES,” Angewandte Chemie, International Edition 3 (1964): 1.,

[65] Y. K. Lau , S. Ikuta , and P. Kebarle , “Thermodynamics and Kinetics of the Gas‐Phase Reactions H3O+(H2O)n‐1 + Water = H3O+(H2O)n,” Journal of the American Chemical Society 104 (1982): 1462.

[66] P. Kebarle , S. K. Searles , A. Zolla , J. Scarborough , and M. Arshadi , “Solvation of the Hydrogen Ion by Water Molecules in the Gas Phase. Heats and Entropies of Solvation of Individual Reactions. H+(H2O)n‐1 + H2O .fwdarw. H+(H2O)n,” Journal of the American Chemical Society 89 (1967): 6393.

[67] C. Lee , W. Yang , and R. G. Parr , “Development of the Colle‐Salvetti Correlation‐Energy Formula into a Functional of the Electron Density,” Physical Review B 37 (1988): 785. 10.1103/physrevb.37.785 9944570

[68] J.‐D. Chai and M. Head‐Gordon , “Long‐range corrected hybrid density functionals with damped atom–atom dispersion corrections,” Physical Chemistry Chemical Physics 10 (2008): 6615. 18989472 10.1039/b810189b

[69] J.‐D. Chai and M. Head‐Gordon , “Systematic optimization of long‐range corrected hybrid density functionals,” The Journal of Chemical Physics 128 (2008): 84106. 10.1063/1.2834918 18315032

[70] A. Najibi and L. Goerigk , “The nonlocal kernel in van der Waals density functionals as an additive correction: An extensive analysis with special emphasis on the B97M‐V and ωB97M‐V approaches,” Journal of Chemical Theory and Computation 14 (2018): 5725. 30299953 10.1021/acs.jctc.8b00842

[71] A. Najibi and L. Goerigk , “DFT‐D4 counterparts of leading meta‐generalized‐gradient approximation and hybrid density functionals for energetics and geometries,” Journal of Computational Chemistry 41 (2020): 2562. 32870518 10.1002/jcc.26411

[72] A. D. Becke , “Density‐functional thermochemistry. III. The role of exact exchange,” The Journal of Chemical Physics 98 (1993): 5648.

[73] P. J. Stephens , F. J. Devlin , C. F. Chabalowski , and M. J. Frisch , “Ab initio calculation of vibrational absorption and circular dichroism spectra using density functional force fields,” The Journal of Physical Chemistry 98 (1994): 11623.

[74] S. H. Vosko , L. Wilk , and M. Nusair , “Accurate spin‐dependent electron liquid correlation energies for local spin density calculations: a critical analysis,” Canadian Journal of Physics 58 (1980): 1200.

[75] J. Tao , J. P. Perdew , V. N. Staroverov , and G. E. Scuseria , “Climbing the density functional ladder: Nonempirical meta–generalized gradient approximation designed for molecules and solids,” Physical Review Letters 91 (2003): 146401. 14611541 10.1103/PhysRevLett.91.146401

[76] V. N. Staroverov , G. E. Scuseria , J. Tao , and J. P. Perdew , “Comparative assessment of a new nonempirical density functional: Molecules and hydrogen‐bonded complexes,” The Journal of Chemical Physics 121 (2004): 11507. 10.1063/1.4971853 28010100

[77] S. Grimme , “Accurate Calculation of the Heats of Formation for Large Main Group Compounds with Spin‐Component Scaled MP2 Methods,” The Journal of Physical Chemistry A 109 (2005): 3067. 16833631 10.1021/jp050036j

[78] Y. Zhao and D. G. Truhlar , “The M06 Suite of Density Functionals for Main Group Thermochemistry, Thermochemical Kinetics, Noncovalent Interactions, Excited States, and Transition Elements: Two New Functionals and Systematic Testing of Four M06‐Class Functionals and 12 Other Functionals,” Theoretical Chemistry Accounts 120 (2008): 215.

[79] S. Kozuch and J. M. L. Martin , “DSD‐PBEP86: in Search of the Best Double‐Hybrid DFT with Spin‐Component Scaled MP2 and Dispersion Corrections,” Physical Chemistry Chemical Physics 13 (2011): 20104. 21993810 10.1039/c1cp22592h

[80] A. L. Parrill and K. B. Lipkowitz , Reviews in Computational Chemistry Ser, (Wiley, 2016).

[81] A. D. Boese , “Density Functional Theory and Hydrogen Bonds: Are We There Yet?” Chemphyschem: A European Journal of Chemical Physics and Physical Chemistry 16 (2015): 978. 25688988 10.1002/cphc.201402786

[82] R. Parthasarathi , V. Subramanian , and N. Sathyamurthy , “Hydrogen bonding in protonated water clusters: an atoms‐in‐molecules perspective,” The Journal of Physical Chemistry A 111 (2007): 13287. 18052052 10.1021/jp0775909

[83] X. Gong , S. Heck , D. Jelovina , et al., “Attosecond spectroscopy of size‐resolved water clusters,” Nature 609 (2022): 507. 35820616 10.1038/s41586-022-05039-8

[84] O. Markovitch , H. Chen , S. Izvekov , F. Paesani , G. A. Voth , and N. Agmon , “Special pair dance and partner selection: Elementary steps in proton transport in liquid water,” The Journal of Physical Chemistry B 112 (2008): 9456. 18630857 10.1021/jp804018y

[85] A. Khan , “Theoretical studies of large water clusters:(H2O) 28,(H2O) 29,(H2O) 30, and (H2O) 31 hexakaidecahedral structures,” The Journal of Chemical Physics 106 (1997): 5537.

[86] T. Wróblewski and G. P. Karwasz , “Protonated water clusters: Hartree‐Fock study of dissociation energies,” European Physical Journal Special Topics 222 (2013): 2217.

[87] P. A. Giguere , “The great fallacy of the H+ ion: and the true nature of H3O+,” Journal of Chemical Education 56 (1979): 571.

[88] E. S. Stoyanov , I. V. Stoyanova , and C. A. Reed , “The Structure of the Hydrogen Ion (H _Aq_ ^+^) in Water,” Journal of the American Chemical Society 132 (2010): 1484. 20078058 10.1021/ja9101826 PMC2946644

[89] A. Malloum and J. Conradie , “Global and local minima of protonated acetonitrile clusters,” New Journal of Chemistry 44 (2020): 17558.

[90] A. Malloum and J. Conradie ,“Solvation free energy of the proton in acetonitrile,” Journal of Molecular Liquids 335 (2021): 116032.

[91] A. Malloum , J. J. Fifen , and J. Conradie , “Binding energies and isomer distribution of neutral acetonitrile clusters,” International Journal of Quantum Chemistry 120 (2020): e26221.

[92] R. Scheu , B. M. Rankin , Y. Chen , K. C. Jena , D. Ben‐Amotz , and S. Roke , “Charge Asymmetry at Aqueous Hydrophobic Interfaces and Hydration Shells,” Angewandte Chemie 126 (2014): 9714. 10.1002/anie.201310266 25045022

[93] C. P. Kelly , C. J. Cramer , and D. G. Truhlar , “Single‐Ion Solvation Free Energies and the Normal Hydrogen Electrode Potential in Methanol, Acetonitrile, and Dimethyl Sulfoxide,” The Journal of Physical Chemistry . B 111 (2007): 408. 17214493 10.1021/jp065403l PMC2528251

[94] E. Rossini and E.‐W. Knapp , “Proton Solvation in Protic and Aprotic Solvents,” Journal of Computational Chemistry 37 (2016): 1082. 26786747 10.1002/jcc.24297

[95] E. Rossini and E.‐W. Knapp , “Erratum: Proton Solvation in Protic and Aprotic Solvents [J. Comput. Chem. 2015, 37, 1082–1091],” Journal of Computational Chemistry 37 (2016): 2163. 27452186 10.1002/jcc.24434

[96] A. Suu , L. Jalukse , J. Liigand , et al., “Unified pH Values of Liquid Chromatography Mobile Phases,” Analytical Chemistry 87 (2015): 2623. 25664372 10.1021/ac504692m

