# Supplementary material for: Proton Solvation in Water and Selected Organic Solvents: A Critical Assessment of Experimental and Theoretical Approaches to Extend the Solvent‐Independent Unified Acidity Scale
Source: Chemphyschem. 2026 Apr 25;27(8):e202500349. doi: 10.1002/cphc.202500349 (PMC13110104; doi:10.1002/cphc.202500349)
Supplement: Supplementary file 1 — Supplementary Material [file CPHC-27-e202500349-s001.pdf]

Supporting Information to

Extending the solvent-independent unified acidity scale: Experimental and theoretical studies on proton solvation in water, methanol, ethanol, acetonitrile and methyl formate

Regina Stroh, Niklas Gebel, Timo Kienzle, Valentin Radtke, and Ingo Krossing*

Contents

1 Experimental Details 2

1.1 General Aspects 2
1.2 Measurement Data 4
1.3 Analysis of the Data 10
1.4 Re-evaluation of the Published Value of $E°_{MeCN}(Ag^+/Ag)$ 10

2 Computational Details 13

2.1 Reference States 13
2.2 Gas-phase Enthalpy and Entropy of the Proton 13
2.3 Point Groups and Symmetry Number 13
2.4 Solvent Properties 14
2.5 Computational and Methodological Details 14
2.6 Benchmarking and Method Selection 16
2.7 Differences between the BP86/def2-TZVPP and DSD-BLYP/def2-TZVPP Structures and Energies 17

3 Optimized Structures and Energies 19

3.1 Mean Absolute Error 19
3.2 Optimized Gas-Phase Structures 19
3.3 Gas-Phase Energies and Solvation Energies of the Clusters 27
3.3.1 Water Clusters 27
3.3.2 Methanol Clusters 38
3.3.3 Ethanol Clusters 44
3.3.4 Acetonitrile Clusters 54
3.3.5 DMF Clusters 58
3.3.6 DMSO Clusters 63
3.3.7 Propylene Carbonate Clusters 68
3.3.8 Methyl Formate Clusters 73
3.4 Gibbs Energies of Solvation of the Proton 80

1 Experimental Details

1.1 General Aspects

Experiments, if not stated otherwise, were carried out under inert argon atmosphere using standard Schlenk techniques and glove boxes with an argon atmosphere containing less than 1 ppm H_2O and O_2. Glass ware was cleaned by immersion and storage overnight in a bath of KOH/iPrOH. After rinsing with H_2O and immersion in a HCl bath for at least 30 min, glass ware was finally washed with deionized H_2O and stored overnight in an oven at 150 °C. Before reactions or cleaning procedures, glass ware was heated under vacuum ($1 \cdot 10^{-3}$ - $5 \cdot 10^{-2}$ mbar) and purged with argon gas, which was repeated three times. Solvents were added to reaction vessels using argon purged syringes and silicon rubber stoppers.

Open Circuit Voltage (OCV) measurements were conducted with a Biologic SP-300 or VMP3 potentiostat controlled with the software EC-Lab (V11.34). Measurements were performed at room temperature, and if not other stated, for constant potential curves the cell potential was read out after about 2 h.

Chemicals were purchased from commercial sources or synthesized in the work group. Solvents were dried over CaH_2 or molecular sieves, distilled and afterwards stored in a Schlenk flask over molecular sieve under argon atmosphere. A list of all used chemicals is shown below in Table S1.

Table S1: List of used chemicals, supplier and purity.

Chemical	Supplier	purity
Acetonitrile	Fisher Scientific	99.9 %, extra dry, Acro Seal
Bis-(trifluormethansulfonyl)-imide	Sigma-Aldrich	99 %
Chloroplatinic acid 8 wt% solution in water	Sigma-Aldrich	–
Ethanol	Fisher Scientific	99.5 %
H_2	SWF	5.0
HCl, conc.	VWR	37 %
HNO_3, conc.	Sigma-Aldrich	65 %
Lead (II) acetate trihydrate	Alfa Aesar	99.995 %
Methanol	Fisher Scientific	99.9 %, extra dry, Acro Seal
Methyl formate	ASG	99.8 %
$[N_{2225}][NTf_2]$	Iolitec	

The NMR-spectrum of the IL of the salt-bridge [N_{2225}][NTf_2] is shown in Figure S1.

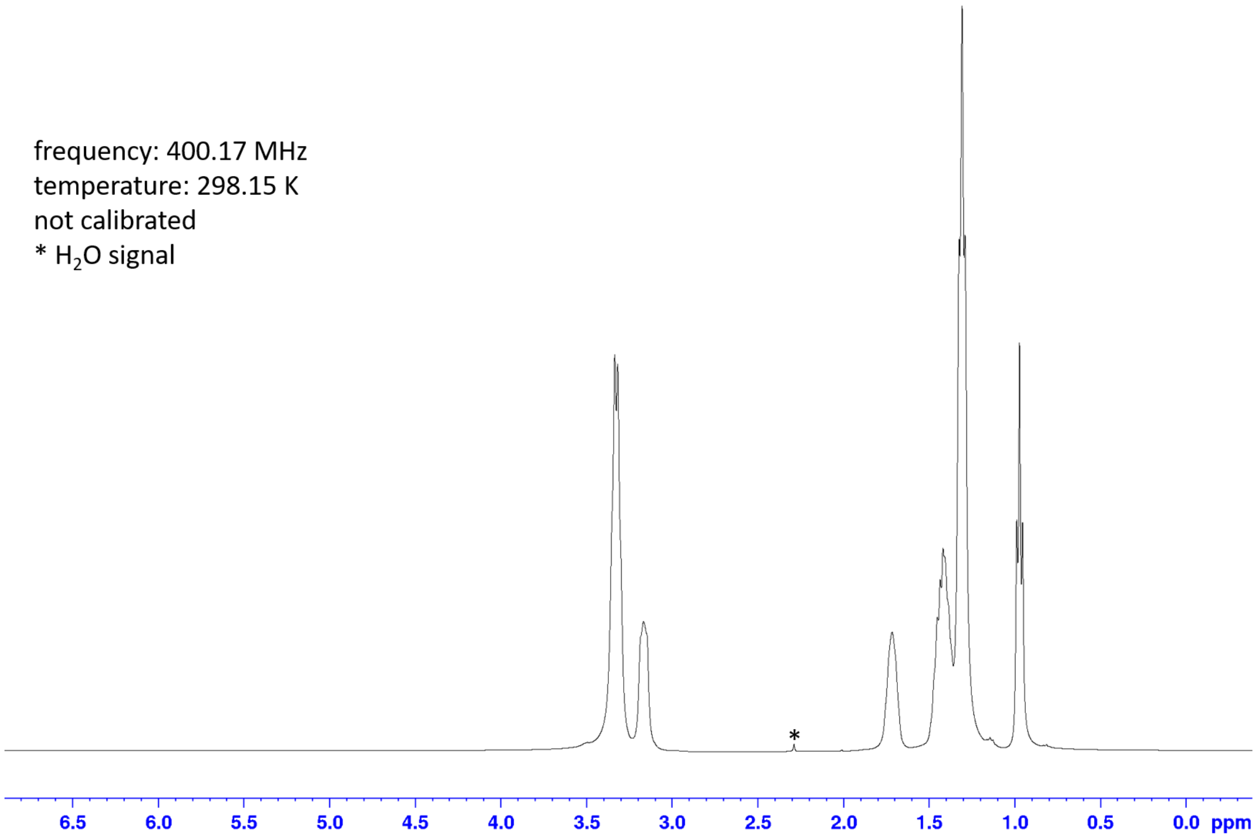

Figure S1: ^{1}H-NMR spectrum of the IL [N_{2225}][NTf_2] used for the salt-bridge.

Hydrogen Electrodes were commercially available platinum electrodes "Platinelektrode 150 x 8 mm" from MBM Lehrmittel und Verlagsgesellschaft mbH. The platinum electrodes were platinized with $H_2[PTCl_6]$ 8 wt % in H_2O (9.0 mL, 1.84 mmol), $Pb(OOCH_3)_2 \cdot 3\ H_2O$ (9.9 mg, 0.03 mmol, 0.01 eq) and H_2O (31.8 mL) at a constant potential of 10 V over the duration of 10 mins. The platinized electrode was washed with deionized water and acetone. For the use as hydrogen electrode, hydrogen gas was fed into the solution via a glass tube flowing over the platinum surface. The hydrogen pressure within the half-cells was maintained at 10^5 Pa.

Silver Electrodes were freshly polished silver wires purchased by MaTecK (99.99%) immersed in a solution of 0.001 mol L^{-1} Ag[*pf*] ([*pf*] $^-$ = $[Al\{OC(CF_3)_3\}_4]^-$) in the solvent under consideration, and used as the half-cell of cell IV.

The **ILSB-Setup** is schematically shown in Figure S2.

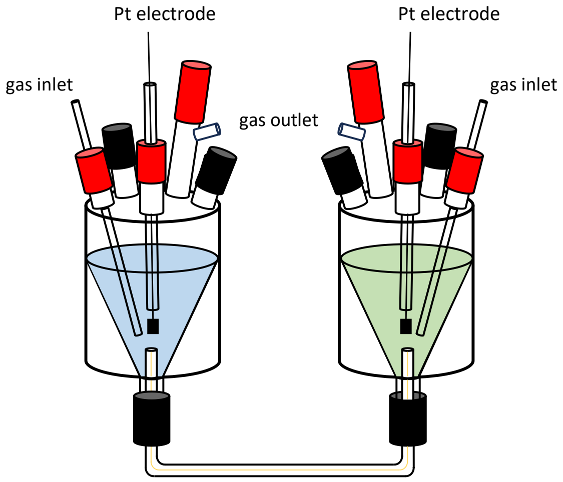

Figure S2: Schematic illustration of the measurement setup. Both platinum electrodes are connected to the potentiostat. The half-cells are connected with a salt-bridge (inner diameter 400 or 800 µm, respectively) filled with the ionic liquid $[N_{2225}][NTf_2]$. If not stated otherwise, the working electrode is always set up in the right half-cell, the counter electrode in the left half-cell.

1.2 Measurement Data

Cell III was assembled in eight different implementations with the solvents S_1 and S_2 vary as well as the counter ions Z^m and Z^n may vary.

$Pt|H_2|HZ^m$ (c, S_2) | $[N_{2225}][NTf_2]$ | HZ^n (yc, S_1)$|H_2|Pt$ cell III

The measured data are given in the Figures S2 – S9. The E_{III} values were read off at constant potential difference (i.e. after 2 hours), or at the maximum value after the completion of hydrogen saturation.

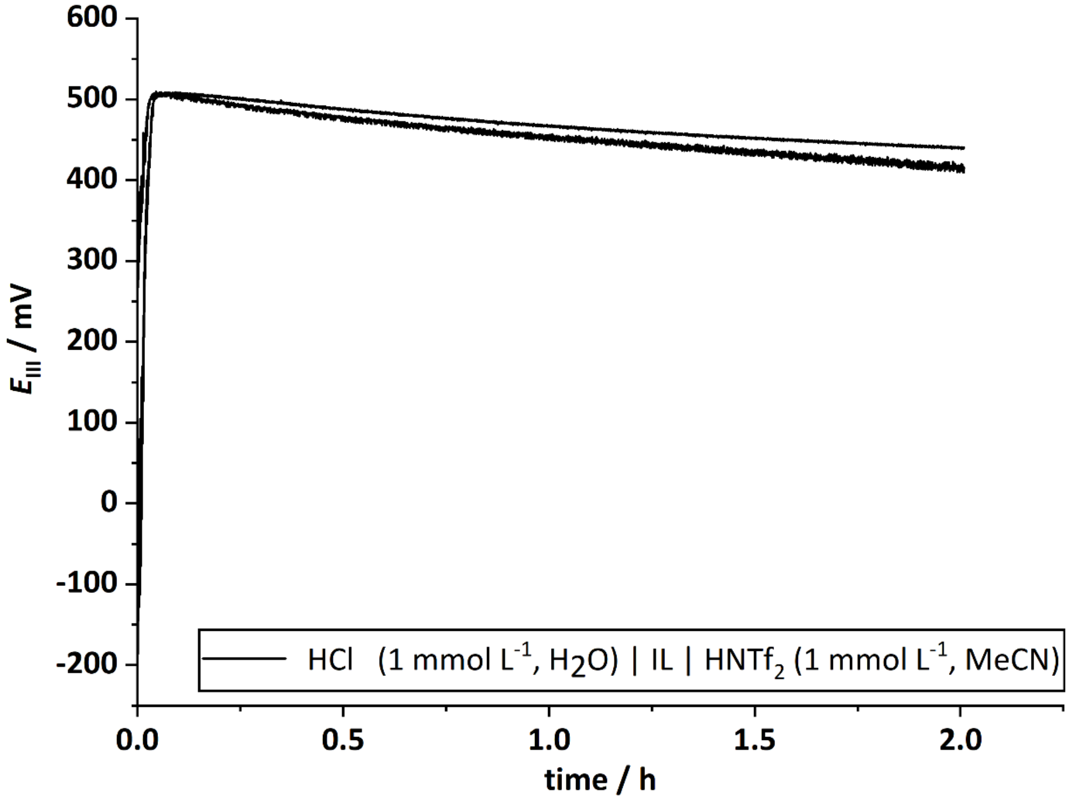

Figure S3: Measured data of cell III as indicated: S_1 = MeCN and S_2 = H_2O (or, according to Figure 2 of the main text: ① = H_2O and ② = MeCN). IL = [N_{2225}][NTf_2]. (Three measurements.) MeCN was used as purchased.

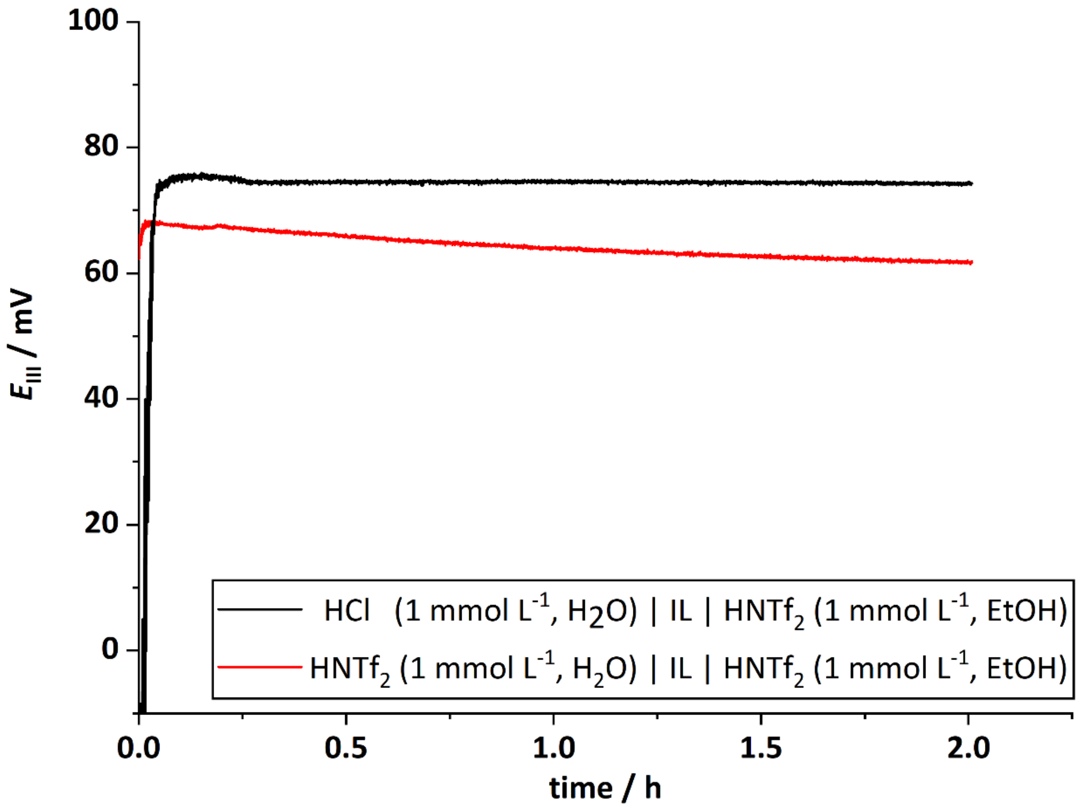

Figure S4: Measured data of cell III as indicated: S_1 = EtOH and S_2 = H_2O (or, according to Figure 2 of the main text: ① = H_2O and ③ = EtOH). IL = [N_{2225}][NTf_2]. (Two measurements.)

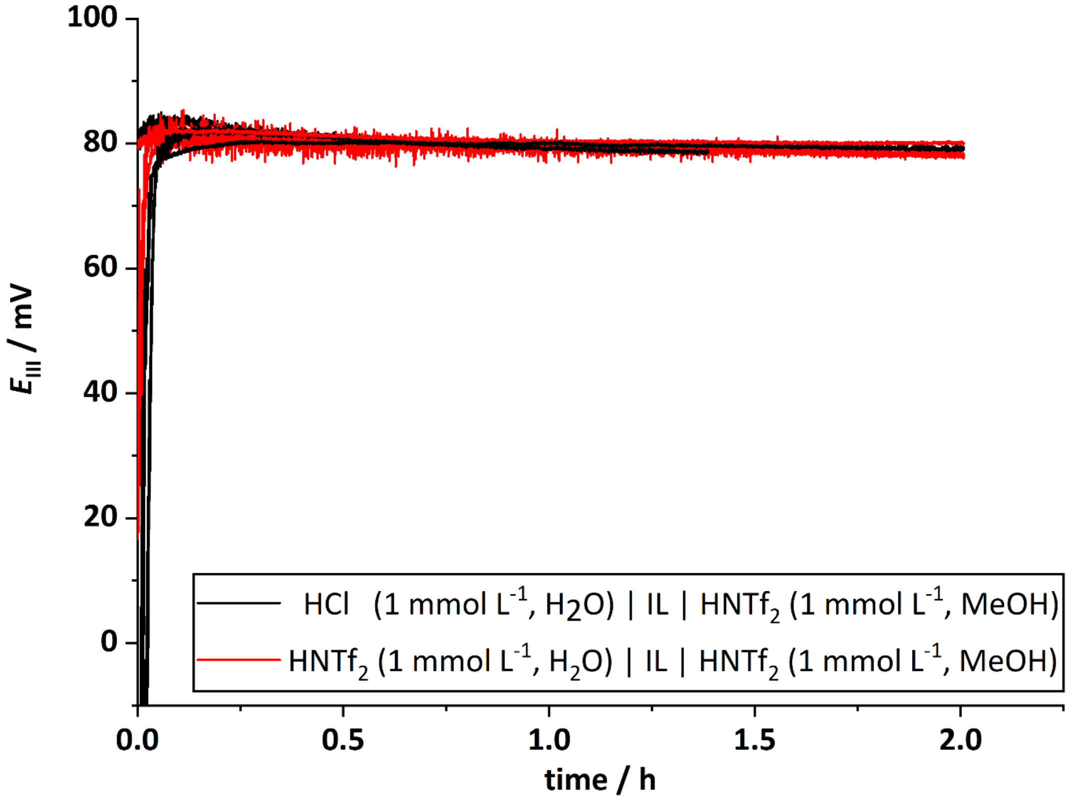

Figure S5: Measured data of cell III as indicated: S_1 = MeOH and S_2 = H_2O (or, according to Figure 2 of the main text: ① = H_2O and ④ = MeOH). IL = $[N_{2225}][NTf_2]$. (Six measurements.)

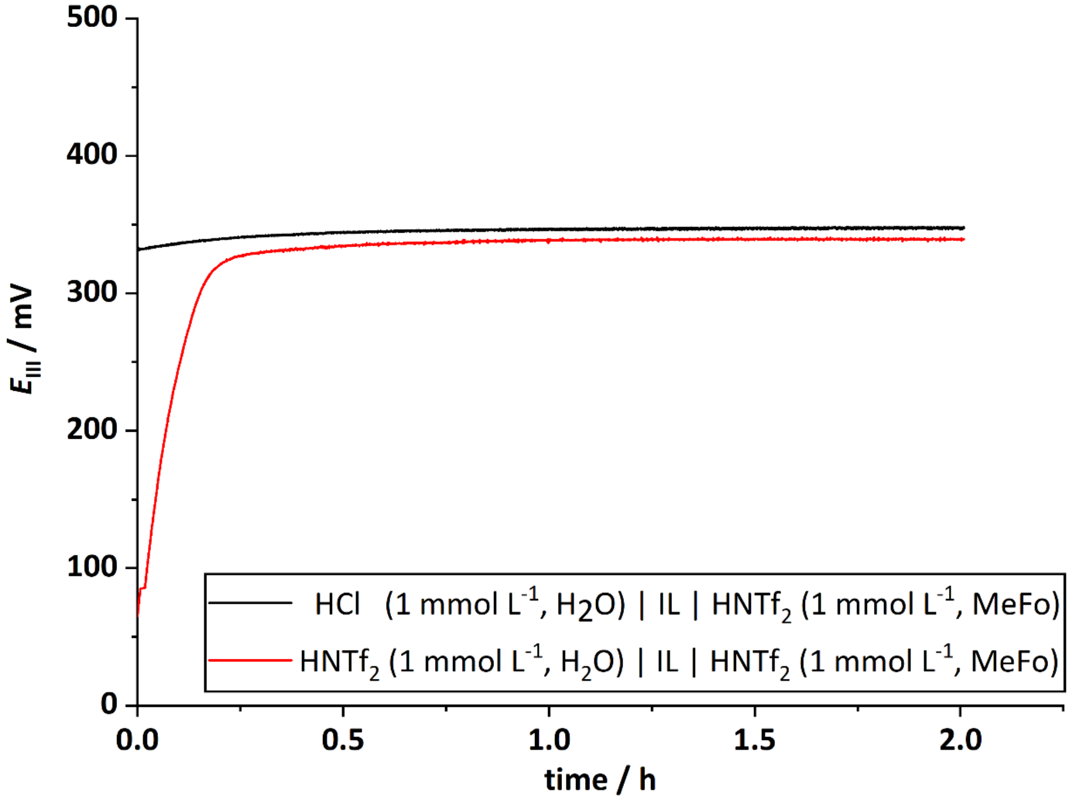

Figure S6: Measured data of cell III as indicated: S_1 = MeFo and S_2 = H_2O (or, according to Figure 2 of the main text: ① = H_2O and ⑤ = MeFo). IL = $[N_{2225}][NTf_2]$. (Two measurements.)

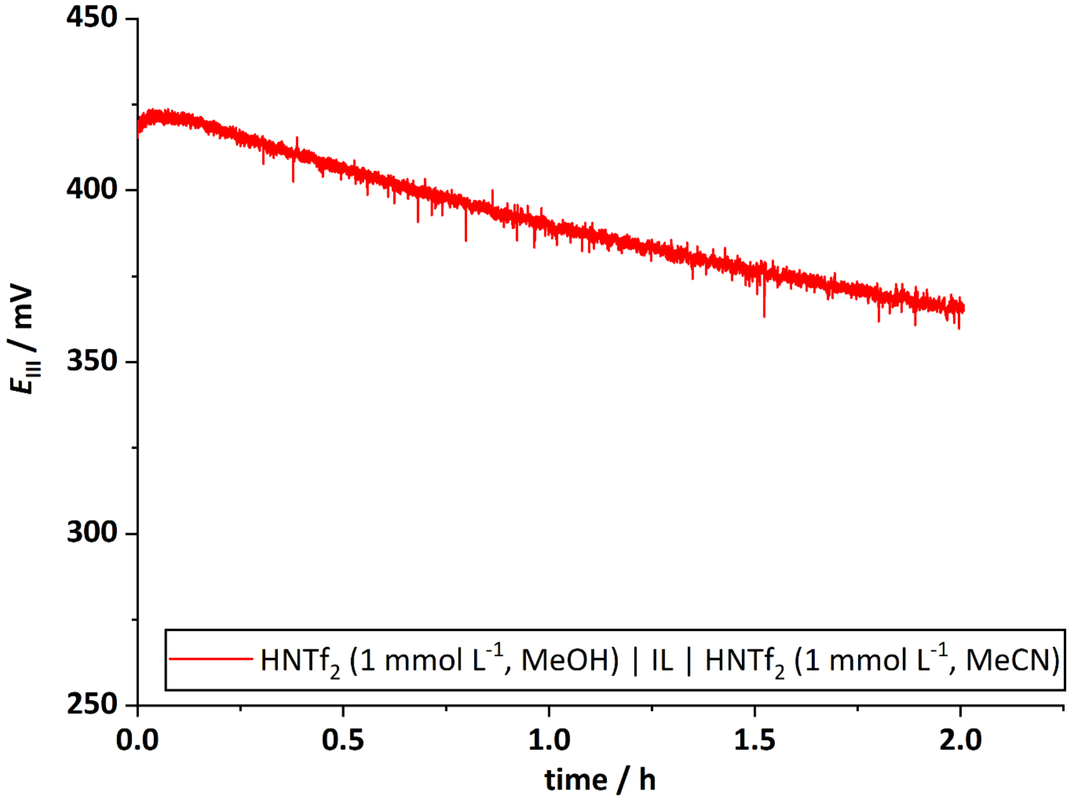

Figure S7: Measured data of cell III as indicated: S_1 = MeCN and S_2 = MeOH (or, according to Figure 2 of the main text: ② = MeCN and ④ = MeOH). IL = [N_{2225}][NTf_2]. (One measurement.) MeCN was used as purchased.

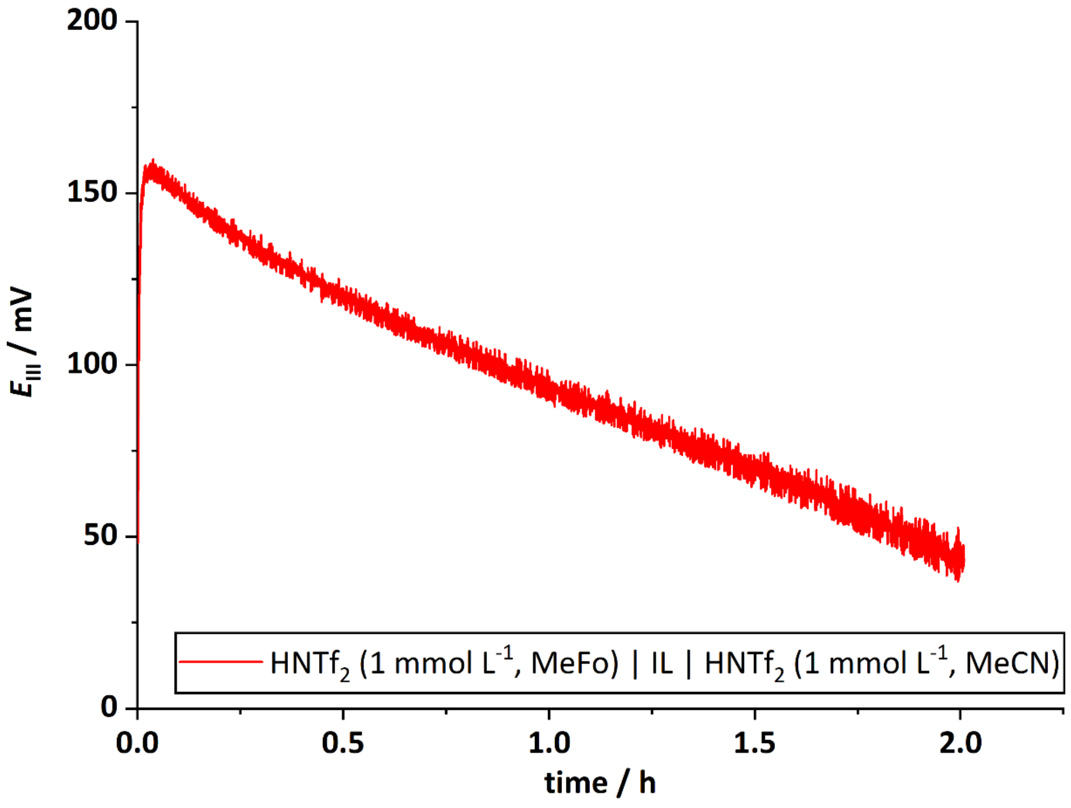

Figure S8: Measured data of cell III as indicated: S_1 = MeCN and S_2 = MeFo (or, according to Figure 2 of the main text: ② = MeCN and ⑤ = MeFo). IL = [N_{2225}][NTf_2]. (One measurement.) MeCN was used as purchased.

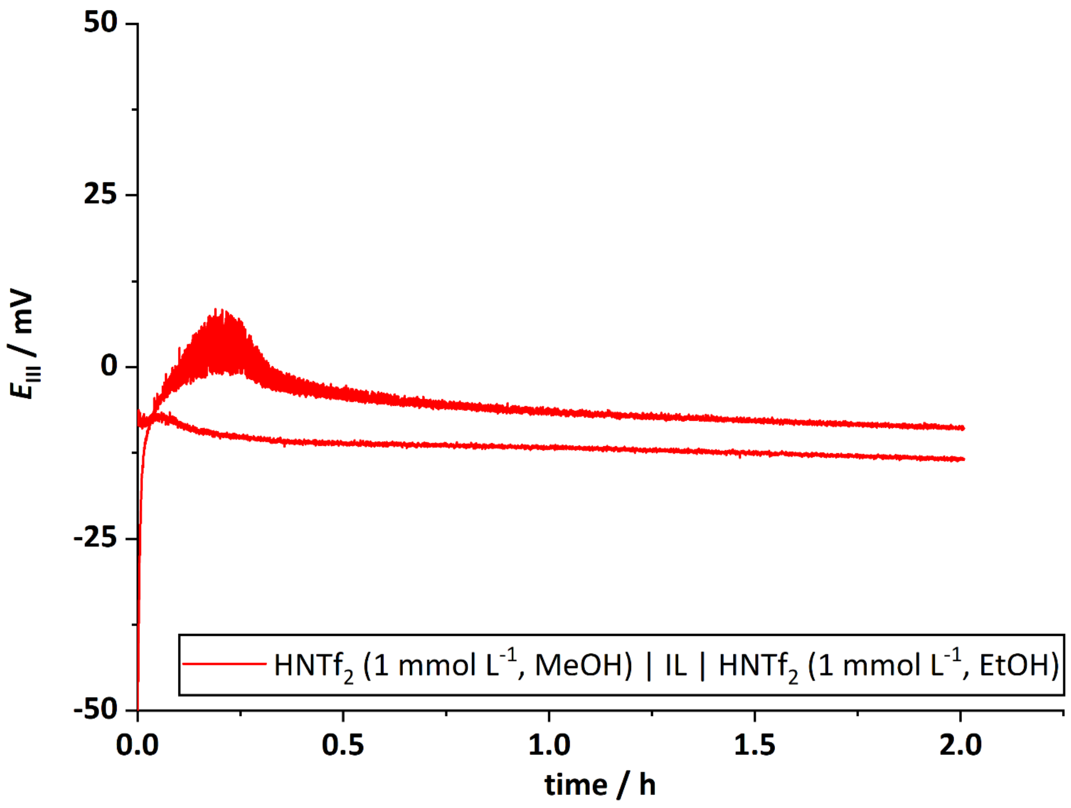

Figure S9: Measured data of cell III as indicated: S_1 = EtOH and S_2 = MeOH (or, according to Figure 2 of the main text: ③ = EtOH and ④ = MeOH). IL = $[N_{2225}][NTf_2]$. (Two measurements.)

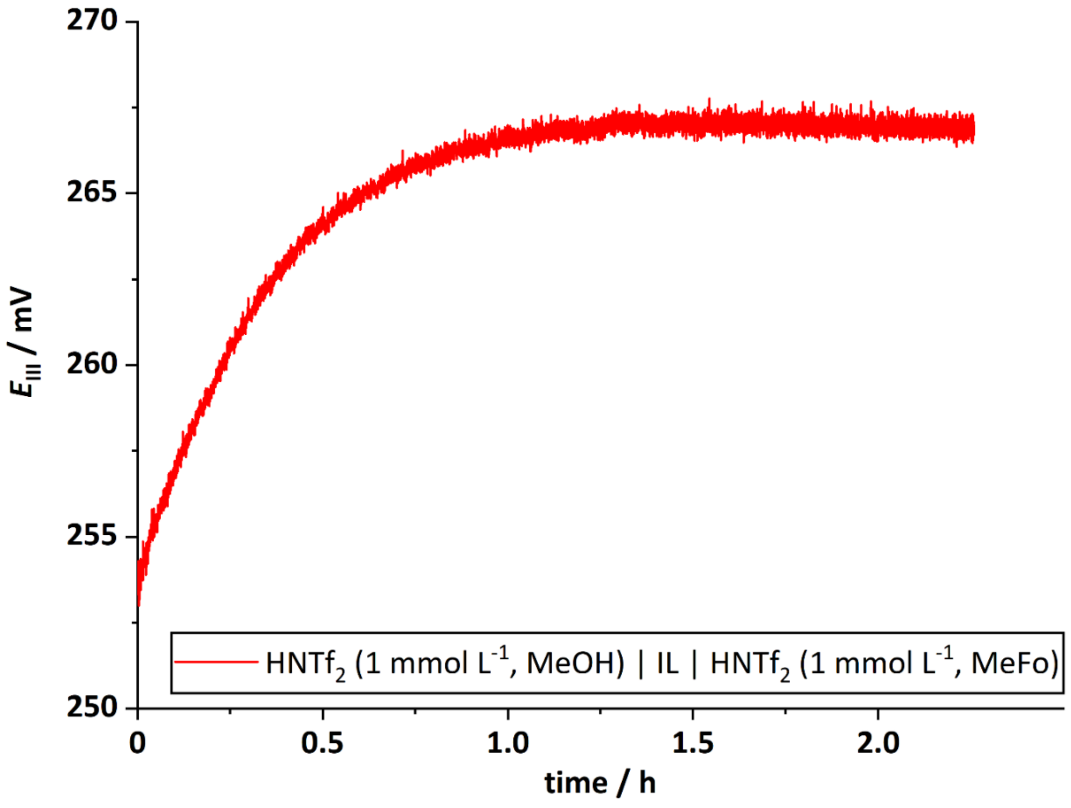

Figure S10: Measured data of cell III as indicated: S_1 = MeFo and S_2 = MeOH (or, according to Figure 2 of the main text: ⑤ = MeFo and ④ = MeOH). IL = $[N_{2225}][NTf_2]$. (One measurement.)

Cell IV was assembled in three different implementations with S was MeCN, EtOH, or MeOH.

Pt|H_2|HZ^m (c, S) | [N_{2225}][NTf_2] | AgZ^n (yc, S)|Ag   cell IV

The counter ion Z^m was the NTf_2^- ion and Z^n was the [pf]$^-$ ion.

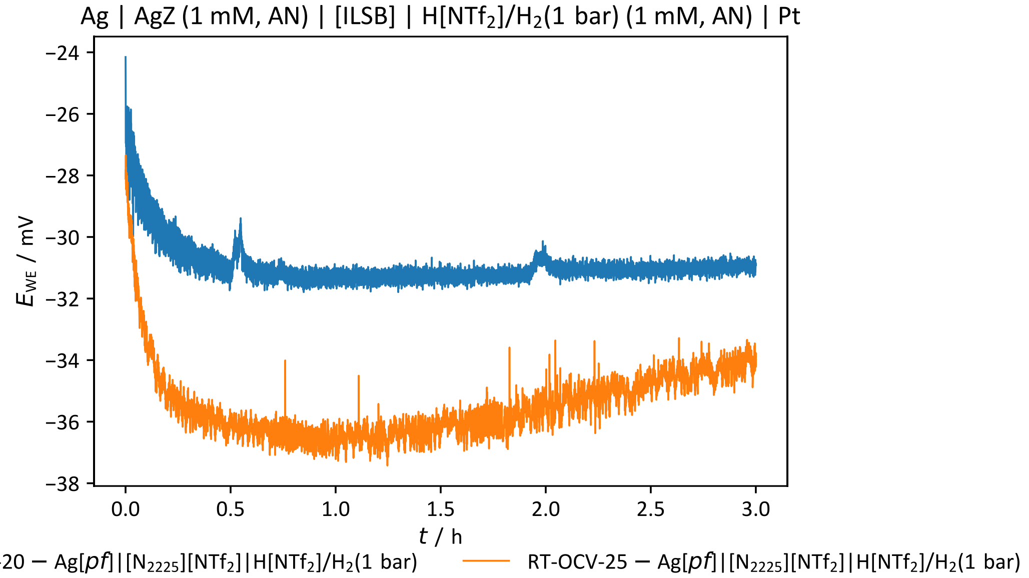

Figure S11: Measured data of cell IV as indicated: S = MeCN. Note, that the reverse of cell IV was measured.

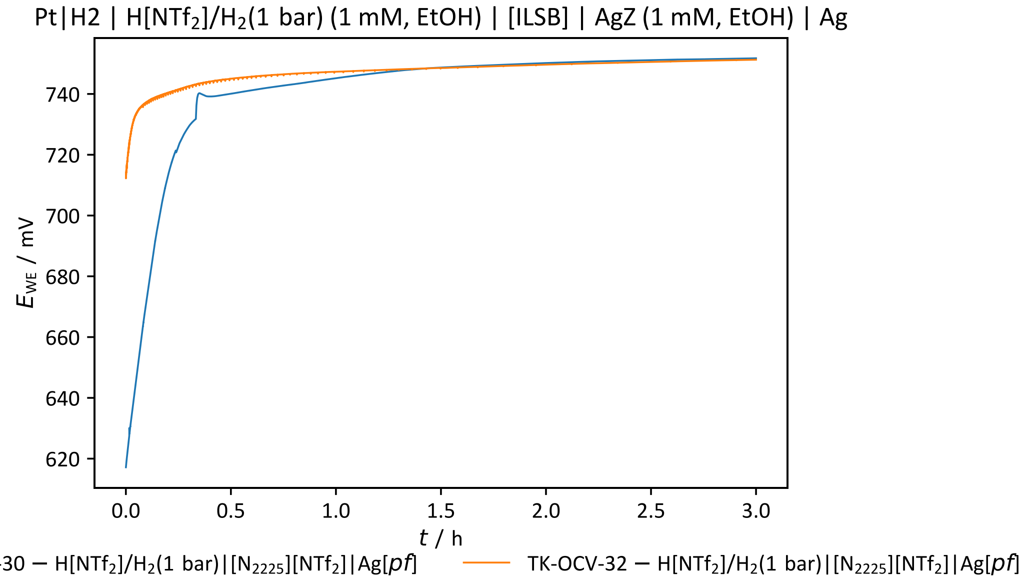

Figure S12: Measured data of cell III as indicated: S = EtOH.

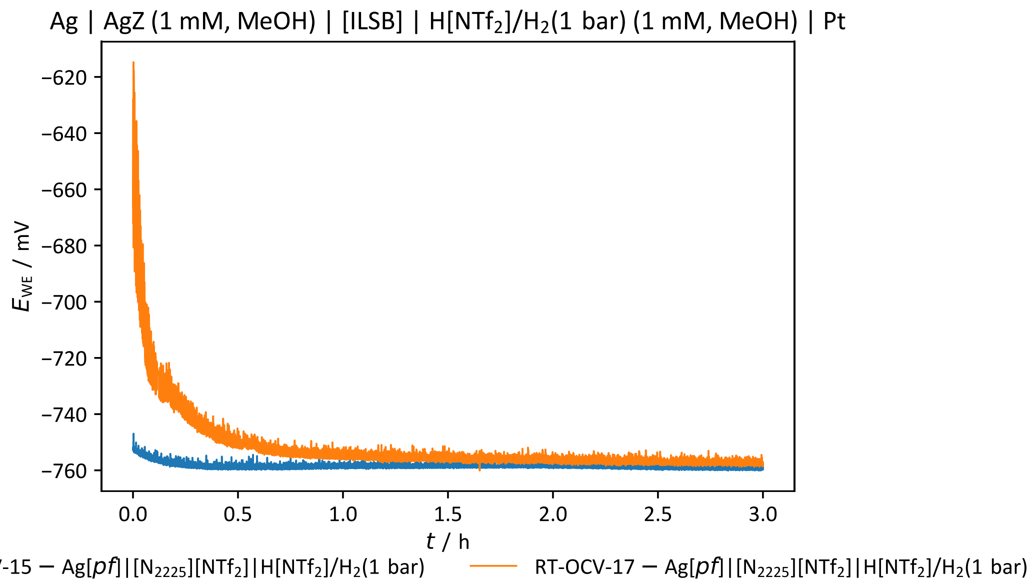

Figure S13: Measured data of cell III as indicated: S = MeOH. Note, that the reverse of cell IV was measured.

1.3 Analysis of the Data

The corresponding equation system to the network from Figure 2 of the main text reads as follows (according to cell 3, the half-cell with solvent S_1 is coded as positive and that with S_2 as negative, the last row represents the zero element):

E_{III} / mV	MeCN	MeFo	EtOH	MeOH	H_2O
504.5	1				−1
342.5		1			−1
69.3			1		−1
79.6				1	−1
157.0	1	−1			
421.5	1			−1	
267.0		1		−1	
-9.1			1	−1	
0					1

It was analysed with the least square method, details can be found in the Supporting Information of doi.org/10.1002/chem.202200509. The result is given in Figure 2 of the main text.

1.4 Re-evaluation of the Published Value of $E°_{MeCN}(Ag^+/Ag)$

To obtain a value for $E°_{MeCN}(Ag^+/Ag)$ Kolthoff *et al.* measured the electric potential difference of Cell K.

Ag | Ag NO_3 (0.01 mol L^{-1}, MeCN) ⁞⁞ NEt_4 HSO_4^- (*c*, MeCN), H_2SO_4 (*c*, MeCN) | H_2 | Pt   cell K

They used different concentrations of the bisulfate buffer, the Table I of their results is reprinted with Table S2.

Table S2: Data as measured and calculated by Kolthoff *et al*.

$[(C_2H_5)_4\text{-}NHSO_4]$ added, m*M*	$[H_2SO_4]$ added, m*M*	$[H^+]$, *M* X 10^5	*f*±	Ionic strength, *I* X 10^3	pH_2, mm	E_{cell}, v.	E''', v.
0.204	5.1	0.848	0.948	0.208	662.8	−0.2600	0.0315
1.02	20.0	2.562	0.897	1.018	662.8	−0.2270	0.0385
2.04	20.0	1.323	0.865	1.954	646.0	−0.2365	0.0418
2.04	50.0	8.33	0.860	2.084	662.8	−0.1917	0.0437
2.04	84.2	22.65	0.856	2.338	662.8	−0.1645	0.0458

E_{cell} is the measured value at cell K, and E''' is calculated according to Eq (S1). The hydrogen pressures pH_2 were corrected and converted to the unit atmosphere, and the proton concentrations $[H^+]$ and the mean activity coefficients *f*± were calculated without going into details.

$$E''' = E_{\text{cell}} - 0.0591\,\text{lg}\frac{a(\text{H}^+)}{\left(p(\text{H}_2)\right)^{1/2}} \qquad \text{(S1)}$$

Then E''' was plotted against the ionic strength *I* and extrapolated to *I* = 0.

Table S3 shows another way of evaluating this data. First, since the correction of the H_2 pressure is not specified, we determined pH_2 by backward calculation (Eq (S1)). Using these values, we calculated the corrected measured value E_{cell}, pH_2 = 1 atm for the pressure 1 atm (Eq (S2), and plotted these values against the pH_{MeCN}, which can be calculated from the specified proton concentrations and the activity coefficient (Eq (S3)).

$$E_{\text{cell, pH}_2} = E_{\text{cell}} + \frac{1}{2}\cdot 0.0591\,\text{lg}\,p(\text{H}_2) \qquad \text{(S2)}$$

$$\text{pH}_{\text{MeCN}} = -\,\text{lg}([H^+]f_{\pm}) = -\,\text{lg}\,a(\text{H}^+) \qquad \text{(S3)}$$

Table S3: Data derived from data of Table S2 with Eqs (S2) and (S3).

$[(C_2H_5)_4\text{-}NHSO_4]$ added, m*M*	$[H_2SO_4]$ added, m*M*	$[H^+]$, *M* X 10^5	*f*±	activity, $a(H^+)$ X 10^5	pH_{MeCN}	pH_2 / atm	$E_{cell,\ pH_2\ =\ 1\ atm}$, / V
0.204	5.1	0.848	0.948	0.80	5.1	0.74	−0.2696
1.02	20.0	2.562	0.897	2.23	4.6	0.51	−0.2356
2.04	20.0	1.323	0.865	1.14	4.9	0.34	−0.2502
2.04	50.0	8.33	0.860	7.16	4.1	0.47	−0.2013
2.04	84.2	22.65	0.856	19.39	3.7	0.47	−0.1741

The extrapolation to pH_{MecN} = 0, whereby entry 3 was considered an outlier and is not taken into account, is shown in Figure S14 (intercept 0.08405; slope −0.06917; R^2 0.9987). From here on we again follow Kolthoff *et al.* (cf. Table IV of their publication): we take into account that the Ag^+ ion concentration was 0.01 mol L^{-1} and that the Ag^+/Ag electrode formed the left half-cell. Accordingly, the value of the standard electrode potential for the Ag+/Ag system in MeCN is $E°_{MeCN}(Ag^+/Ag)$ = 0.046 V.
It can be seen that the values fit well on a straight line. However, the slope of the line corresponds to 0.0692 V pH^{-1} and not the expected value of 0.0591 V pH^{-1}. If one extrapolates with the default of this slope, one obtains a value for $E°_{MeCN}(Ag^+/Ag)$ = 0.090 V (R^2 = 0.97752). (It should be noted at this point that this type of evaluation of the linear fit is not possible when extrapolating to zero ionic strength.) We can only speculate about the reasons why the expected slope is not found. However, the authors stated the measurement accuracy of their instrument as ±0.2 mV, but did not specify how $[H^+]$ and how f± were calculated. Therefore, it is difficult to estimate the uncertainty of the pH_{MeCN} values of the buffer solutions (and, of course, their distances to each other).

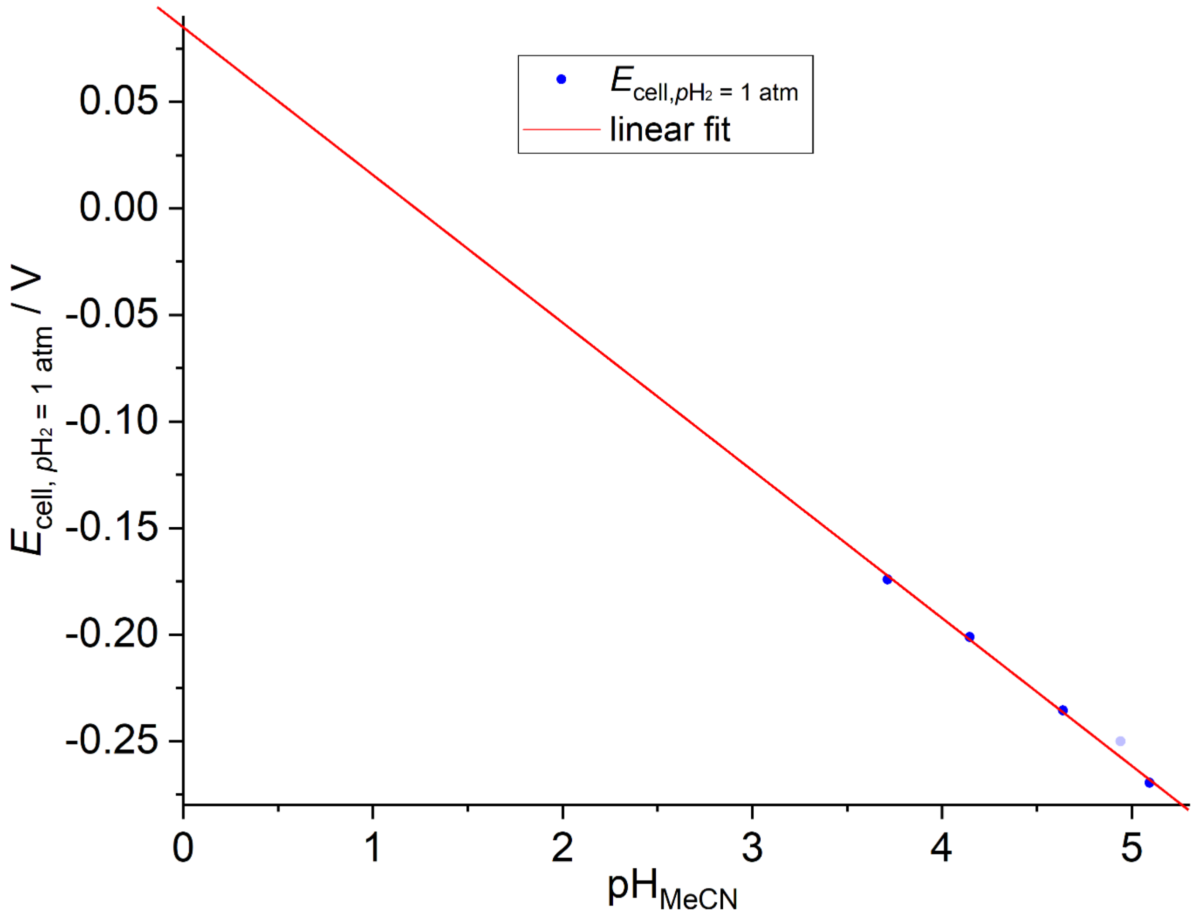

Figure S14: Linear fit of the data from Kolthoff *et al.* in a different manner than originally published. The intercept of 0.084 corresponds to $E°_{MeCN}(Ag^+/Ag)$ = 0.046 V. The faint measurement data is not considered when fitting.

2 Computational Details

2.1 Reference States

The reference state of the Gibbs energy of solvation resulting from both the COSMOtherm and the CPCM calculations is 1 mol L^{-1} in the gas-phase and 1 mol L^{-1} in solution (labeled $\Delta_{\text{solv}}G^{*}$). This was adjusted to a reference of 1.01325 bar gas to align with the gas-phase calculations and 1 mol L^{-1} solution (labeled as $\Delta_{\text{solv}}G°$) by adding 7.96 kJ mol^{-1} to the calculated solvation energies. All energies, constants and quantities either used or obtained from the calculations refer to a temperature of 298.15 K, unless stated otherwise.

2.2 Gas-phase Enthalpy and Entropy of the Proton

The calculations require the Gibbs energy of the proton, which can be determined from principles of statistical thermodynamics. The proton gas is an ideal monoatomic gas, enabling the calculation of its gas-phase entropy using the Sackur-Tetrode equation.

$$S(\mathrm{H}^{+}) = R\ln\left(\frac{\exp\left(\frac{5}{2}\right)\ \mathrm{k_B}T}{p\lambda^{3}}\right) = 0.109\ \mathrm{kJ\ mol^{-1}}. \tag{S4}$$

Here, R represents the ideal gas constant, k_B is the Boltzmann constant, λ is de Broglie wavelength and *p* symbolized the pressure.

The gas-phase enthalpy can be obtained from the ideal gas expression and amounts to

$$H(\mathrm{H}^{+}) = U + pV = \frac{5}{2}RT = 6.201\ \mathrm{kJ\ mol^{-1}}. \tag{S5}$$

By using the Gibbs-Helmholtz equation, the combined contributions result in a free energy of −26.3 kJ mol^{-1} at 298.15 K.

2.3 Point Groups and Symmetry Number

For every geometry optimization, the point group determined by ORCA was verified and adjusted if necessary. The point group defines the symmetry number (sn), which affects the rotational entropy S_{rot}

Adjustments were made to the rotational entropy based on the symmetry number of the selected point group.

Table S4 shows the classification of symmetry numbers and point groups, following the work of Herzberg.[148]

Table S4: Point groups and their associated symmetry numbers sn used to assign the rotational entropy.

sn	Point groups[a]	
**1**	C_1, C_i, C_s	$C_{\infty v}$
**2**	C_2, C_{2v}, C_{2h}	$D_{\infty h}$, S_4
**3**	C_3, C_{3v}, C_{3h}	S_6
**4**	C_4, C_{4v}, C_{4h}	D_2, D_{2d}, $D_{2h} = V_h$
**6**	C_6, C_{6v}, C_{6h}	D_3, D_{3d}, D_{3h}
**8**	D_4, D_{4d}, D_{4h}	
**12**	D_6, D_{6d}, D_{6h}	T, T_d
**24**	O_h	

[a] Allocation of point groups to symmetry numbers was taken from the ORCA manual.[149]

2.4 Solvent Properties

Table S5 presents a summary of all experimental values used to calculate the Gibbs energy of solvation through thermodynamic cycles. The energy of vaporization was calculated from the vapor pressure ρ_{vap} through Eq (S6).

$$\Delta_{vap}G°(S) = -RT\ln\left(\frac{\rho_{vap}}{1\ bar}\right). \tag{S6}$$

The concentration c of the solvent was obtained from the density ρ and the molar mass M.

Table S5: Properties of the solvents used for the calculations at 298.15 K. Relative permittivities used to calculate the solvation energies with the CPCM, vapor pressures ρ_{vap} and the Gibbs energies of evaporation $\Delta_{vap}G°$ calculated from them, densities ρ with the concentrations c and the $RT\ln(c)$ term calculated from them.

Solvent	M<br>g mol⁻¹	ε_r[96]	ρ_{vap}[150]<br>bar	$\Delta_{vap}G°$[a]<br>kJ mol⁻¹	ρ[150]<br>g cm⁻³	c<br>mol L⁻¹	$RT\ln(c)$<br>kJ mol⁻¹
Water	18.015	78.355	0.031700	8.5560	0.997	55.343	9.949
MeCN	41.052	35.688	0.119000	5.2768	0.7767[151]	18.920	7.289
MeOH	32.042	32.613	0.169594[152]	4.3985	0.791[153]	24.686	7.948
EtOH	46.068	24.852	0.078700	6.3018	0.786[154]	17.040	7.029
DMSO	78.133	46.826	0.000797	17.6878	1.101	14.091	6.558
DMF	73.094	37.219	0.004390	13.4569	0.9445	12.922	6.343
PC	102.089	64.92[97]	7.77E-5[155]	23.4575	1.1978[156]	11.733	6.104
MeFo	60.052	8.838	0.781000	0.6127	0.974[157]	16.219	6.907

[a] Calculated through Equation (S6) from ρ_{vap}.

2.5 Computational and Methodological Details

All structures were calculated with the ORCA 5.0.3 program packages.[78–81] The structures were optimized at the DSD-BLYP[63]/def2-TZVPP[91] level of theory with the RIJCOSX approximation and the def2/J[158] and def2-TZVPP/C[159] auxiliary basis sets. The parameterization of the DSD-double-hybrid was consistent with the GMTKN55 benchmark[102] and was combined with the atom-pairwise dispersion

correction with Becke-Johnson damping scheme (D3BJ).[98,99] Numerical frequency calculations confirmed that all gas-phase geometries were true minima on the potential hypersurface through the absence of imaginary modes. The optimization used the largest integration grid (defgrid3) and default SCF settings unless changes were required to reach convergence or to reduce imaginary modes caused by numerical noise. The CPCM[74,75,160] calculations ran with identical settings using the Gaussian charge scheme[161] instead of the point charge scheme. Instead of utilizing tesserae with finite areas, this scheme employs a scaled vdW-surface with a smeared Gaussian charge and Lebedev quadrature to discretize the surface (*vdw_gaussian* surface-type). The respective dielectric constants at 298.15 K are listed in Table S5. The solvation energy of each structure was determined by subtracting the total electronic energy (E_{tot}) of the CPCM structure from the E_{tot} of the gas-phase structure. Some imaginary modes could not be eliminated from the CPCM calculations and are listed as such in Chapter 3.

The optimized gas-phase structures were then used for DLPNO-CCSD(T)[53,54] single-point calculations that were extrapolated to the complete basis set limit (CBS) through the cc-pVQZ[100,101] and cc-pV5Z [100,101] basis sets with the integrated extrapolation method (extrapolate 4/5). The DLPNO approximation used the auxiliary basis cc-pV5Z/C[162] along tight PNO and tight SCF settings.

The optimized CPCM structures were used for DLPNO-CCSD(T)/CBS-CPCM single-points with the *perturbation theory energy singles* (PTES, *CPCMccm 2*) scheme, but otherwise identical settings to the gas-phase calculations used to get the solvation energies at the DLPNO-CCSD(T)/CBS level of theory.

Optimized CPCM structures were employed for DLPNO-CCSD(T)/CBS-CPCM single-point calculations using the PTES (*perturbation theory energy singles*) scheme. Apart from this difference, the calculations mirrored the settings used in the gas-phase calculations. Only the CPCM structure with the highest electronic energy, $E_{\mathrm{tot,cpcm}}$, was chosen for each solvent cluster $(S)_n$ and protonated solvent cluster $H(S)_n^+$, in order to calculate the solvation energy, while different gas-phase structures were used. Therefore, the solvation energies of the different isomers of the clusters refer to the same CPCM structure.

Further computations were performed using the BP[58,60,61] functional and the def2-TZVPP basis set, both in the gas-phase and with the CPCM. In addition, the RI approximation with the def2/J auxiliary basis, DefGrid 3 settings, and the D3BJ dispersion correction were applied. Analytical frequency calculations confirmed the ground state of the structures, unless stated otherwise. Additional DLPNO-CCSD(T)/CBS single-points on the BP structures were conducted with the same settings as described above for the single-points on the DSD-BLYP/def2-TZVPP structures.

Test calculations were conducted on the protonated water clusters with the DSD-BLYP functional in combination with different basis sets, such as def2-QZVPP[91], cc-pV5Z[100,101] and cc-pV6Z. Other settings remained unchanged from the description given above.

Further test calculations employed different functionals with the def2-TZVPP basis set, including DSD-PBEP86[64], ωB97X-D3BJ,[105–108] B3LYP,[59,109–111] TPSSh,[112,113] TPSS0,[114] M06[115] and the BLYP[58,59]

functional. Except for the basis set, all other settings of the functional were kept identical, including the auxiliary basis (def2/J), integration grid (defgrid3), D3BJ dispersion correction, standard SCF settings, and analytical frequency calculations. Variations in the settings where they were necessary are summarized in Table S6 for each functional.

Table S6: Settings of the test calculations conducted with different density functionals. Unless stated otherwise under the settings column, all calculations included the def2-TZVPP basis set with the auxiliary basis def2/J, integration grid defgrid3, D3BJ dispersion correction, normal SCF and optimization settings, and analytical frequency calculation.

Functional	Settings that differ from the defaults
**DSD-PBEP86**	RIJCOSX approximation, numerical frequency calculation
**B3LYP**	RIJCOSX approximation
**ωB97X-D3BJ[a]**	RIJCOSX approximation
**M06**	RI, D3zero (atom-pairwise dispersion correction with zero damping)
**TPSS0**	RI approximation
**TPSSh**	RI approximation
**BLYP**	RI approximation

[a] modified version of ωB97X with adjustments to the D3BJ correction.[107,108]

Minor modifications to convergence radii, including the self-consistent field (SCF) and optimization criteria, were made to address convergence issues or imaginary modes.

Comment on the Methods Used: The theoretical methods chosen for this work were determined by calculations performed in this study and comparisons with data from the GMTKN55 database.[46] Besides accuracy, speed and cost, the main desired feature was the ability to avoid imaginary modes commonly occurring during optimizations of non-covalently bound structures, such as solvent clusters, due to their relatively flat potential energy hypersurface (PES). This issue primarily impacted structures with CH_3-groups, causing small imaginary frequencies, particularly during the CPCM calculations (see below "Isomers and their Weighting"). The lack of experimental solvation energies for both solvent clusters and protonated solvent clusters makes it difficult to directly assess the accuracy of the CPCM calculations.

2.6 Benchmarking and Method Selection

Gas Phase Basicity Calculation: All calculated and experimental[47] GBs are collected in Table S7 together with the associated MAEs for each method and the differences in the bond lengths $\Delta d(H^+–X)$ resulting from the two optimization methods.

Table S7. Gas-phase basicities at 1 bar pressure in kJ mol^{-1} of water, MeCN, DMF, DMSO, EtOH, MeOH, PC and MeFo obtained from optimizations with the BP/def2-TZVPP and the DSD-BLYP/def2-TZVPP methods and the DLPNO-CCSD(T)/CBS single-point calculation on the optimized DSD-BLYP/def2-TZVPP structures. Differences in the $H^+–X$

bond lengths Δ*d*(H^+–X) obtained with BP/def2-TZVPP compared to DSD-BLYP/def2-TZVPP are given in pm.

Solvent	BP/ def2-TZVPP	DSD-BLYP/ def2-TZVPP	Δ*d*(H^+–X) / pm[a] BP ↔ DSD-BLYP	DLPNO-CCSD(T)/ CBS	Exp[47]
H_2O	665.8	663.3	0.8	657.0	660.0
MeCN	764.9	751.9	1.1	748.7	748.0
DMF	852.4	854.6	1.5	855.7	856.6
DMSO	859.0	861.4	1.2	854.3	853.7
EtOH	754.6	750.7	1.1	745.2	746.0
MeOH	725.8	727.4	1.1	722.3	724.5
MeFo	756.8	754.4	1.1	750.6	751.5
PC	803.7	800.9	0.8	797.6	NA
**MAE**	**6.8**	**3.9**	**1.1**[b]	**1.3**	

[a] Δ*d*(H^+–X) = |*d*(H–X, BP/def2-TZVPP) − *d*(H–X, DSD-BLYP/def2-TZVPP)|, with X = O and X = N for MeCN.
[b] The mean absolute error of 1.1 for the H^+–X bond shows a difference between the two methods, unlike the other MAEs, which show differences related to the experiment.

2.7 Differences between the BP86/def2-TZVPP and DSD-BLYP/def2-TZVPP Structures and Energies

Hence, in the BP/def2-TZVPP description, the $H_{15}O_7^+$ isomers have a different arrangement compared to the isomers from the DSD-BLYP/def2-TZVPP optimization. Figure S15 compares the gas-phase clustering energies $-\Delta_g G°$ of the monomer cycle reaction for BP/def2-TZVPP and DSD-BLYP/def2-TZVPP for the most favorable isomers only.

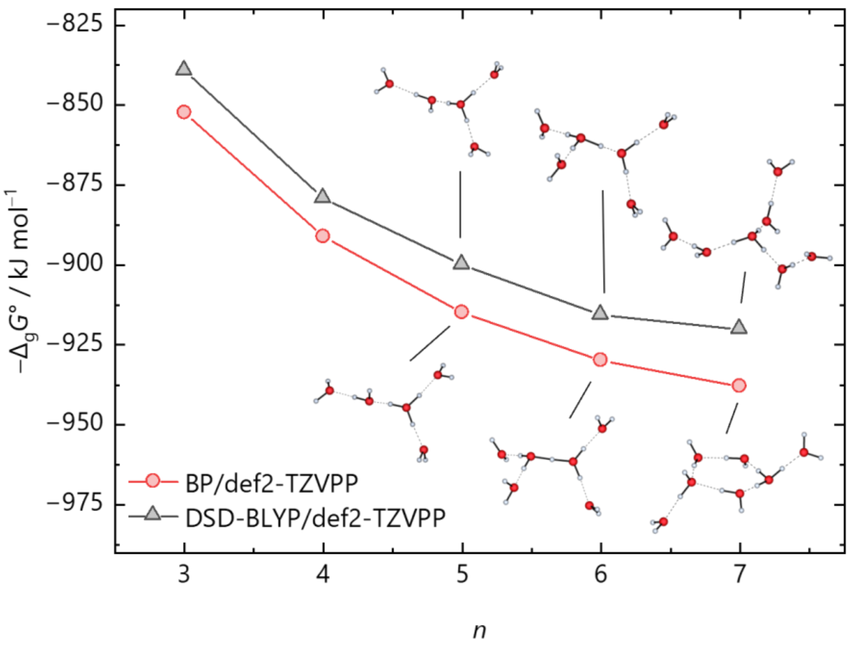

Figure S15. Gas-phase clustering energy $-\Delta_g G°$ at 1 bar pressure for the reaction $H(H_2O)_n^+ \rightarrow H^+ + n\ H_2O$ with n = 1–7 calculated with BP/def2-TZVPP and DSD-BLYP/def2-TZVPP. These energies refer only to the most favorable isomer for each *n*, with the structures of the respective isomers for n = 5–7. All energies are provided in kJ mol^{-1}.

The graph shows the structures of the most favorable isomers with n = 5–7 for each method. It

illustrates the change of the most stable isomer $H_{15}O_7^+$(1) with respect to the method. However, the energy values show a gradual progression, free from sudden changes that would indicate inconsistencies in these structures. Although the smooth energy progression does not rule out structural inconsistencies.

Optimized BP86/def2-TZVPP Structures: The optimized BP/def2-TZVPP gas-phase structures of the protonated water clusters $H(H_2O)_n^+$ show only minor changes of approximately 1 pm compared to the double-hybrid structures, except for the $H_{13}O_6^+$ (1) isomer. As expected, the bond lengths from the BP/def2-TZVPP calculation are slightly longer than those from the more advanced double-hybrid functional.

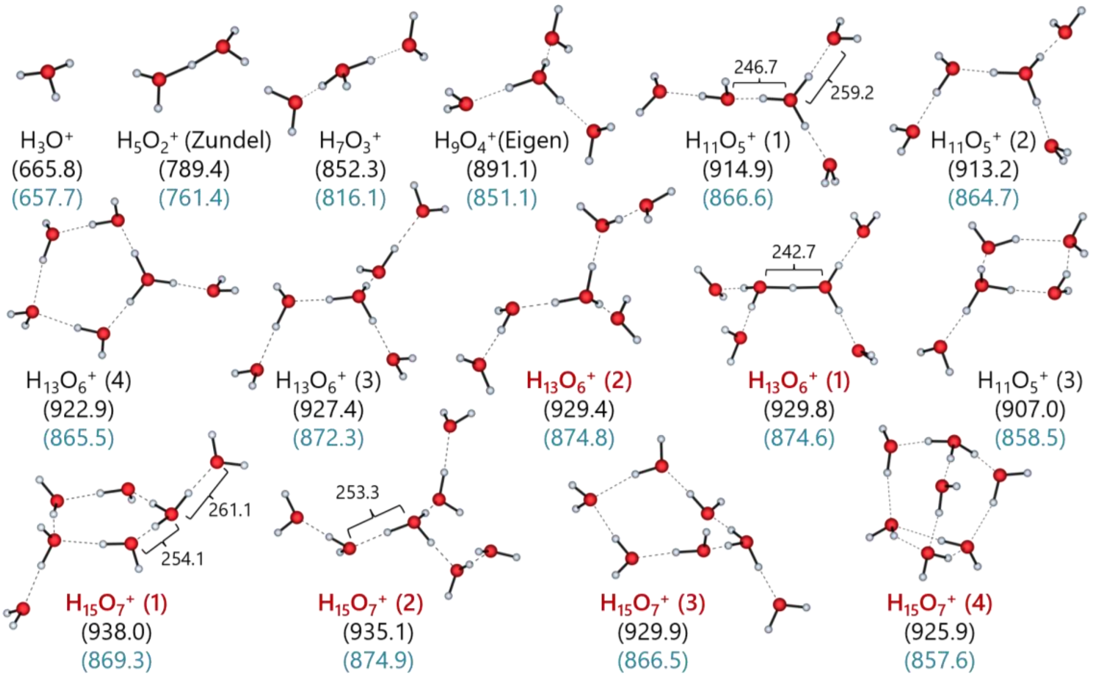

Figure S16. Optimized BP/def2-TZVPP gas-phase structures of the protonated water clusters $H(H_2O)_n^+$ for n = 1–7. The gas-phase clustering energies $\Delta_g G°$ ($H(H_2O)_n^+ \rightarrow H^+ + nH_2O$) under a pressure of 1 bar are provided in kJ mol^{-1} at the BP/def2-TZVPP level of theory (black) and the subsequent DLPNO-CCSD(T)/CBS single-point calculation on the optimized structure (blue). Isomers that change their order depending on whether the DLPNO-CCSD(T)/CBS energies or the BP/def2-TZVPP energies are highlighted in red. Key interatomic distances (in pm) are shown for some of the most stable isomers.

3 Optimized Structures and Energies

Raw data from the all the performed calculations, including frequencies, thermochemical data, and structure files in ASCII format with Cartesian coordinates, are available at 10.5281zenodo.14531121.

3.1 Mean Absolute Error

The mean absolute error (MAE) was calculated according to Eq (S7).

$$\text{MAE} = \frac{1}{j}\sum_{i=1}^{j}|x_i - y_i| \tag{S7}$$

The calculated value is represented by x_i and y_i is the experimental value. The total number of data points is denoted by j.

3.2 Optimized Gas-Phase Structures

These Figures represent all optimized gas-phase structures calculated using DSD-BLYP/def2-TZVPP as well as some exemplary structures from DSD-BLYP/def2-TZVPP-CPCM and BP/def2-TZVPP. Energies depicted in the following figures are given in kJ mol^{-1}. Solvation energies $\Delta_{solv}G°$ refer to standard conditions (transition of 1 bar gas to an ideal solution of 1 mol L^{-1}) and gas-phase clustering energies refer to a state of 1 bar. Hydrogen bonds are illustrated only when their location is clear and omitted when there are multiple possibilities.

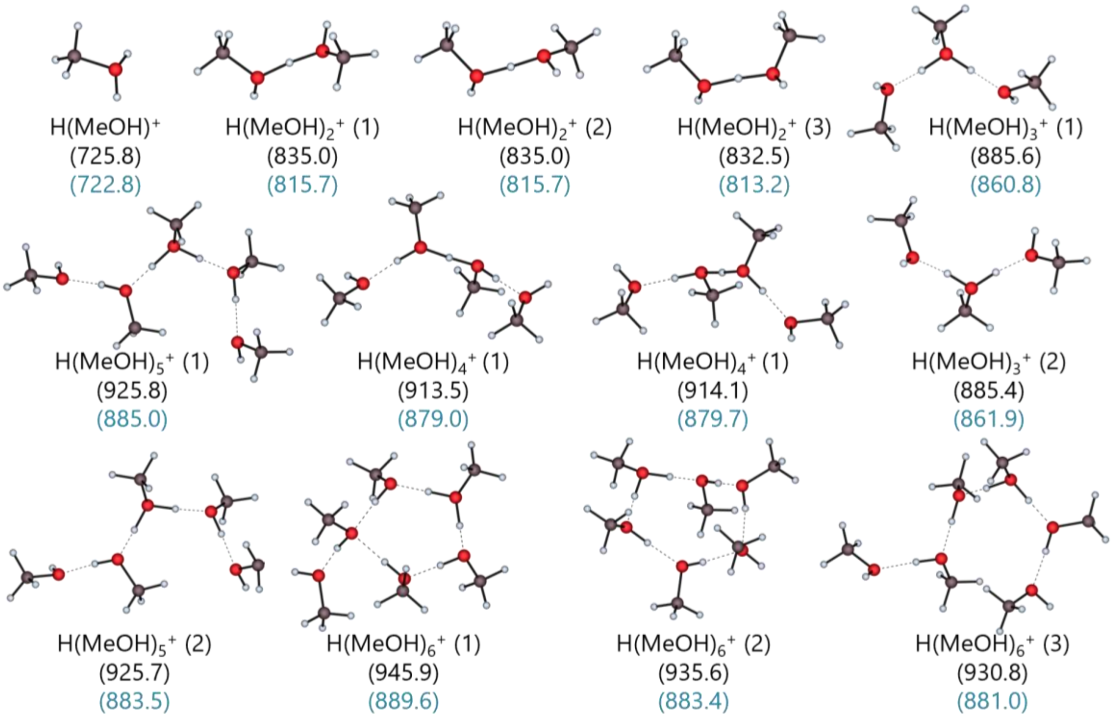

Figure S17: Optimized BP/def2-TZVPP gas-phase structures of the protonated methanol clusters $H(MeOH)_n^+$ for $n = 1–6$ with the gas-phase clustering energies $\Delta_g G°$ from the BP/def2-TZVPP optimization (black) and the subsequent DLPNO-CCSD(T)/CBS single-point (blue) on the optimized structures. All energies given in kJ mol^{-1}.

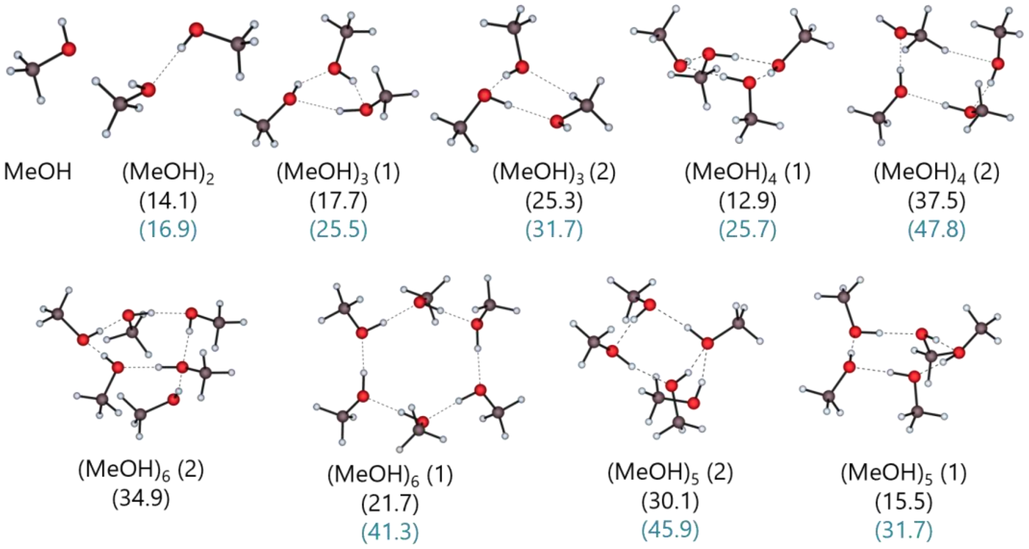

Figure S18: Optimized DSD-BLYP/def2-TZVPP gas-phase structures of the neutral MeOH clusters $(MeOH)_n$ for n = 1–6 with the gas-phase clustering energies $\Delta_g G°$ from the DSD-BLYP /def2-TZVPP optimization (black) and the subsequent DLPNO-CCSD(T)/CBS single-point (blue) on the optimized structures. All energies are provided in kJ mol^{-1}.

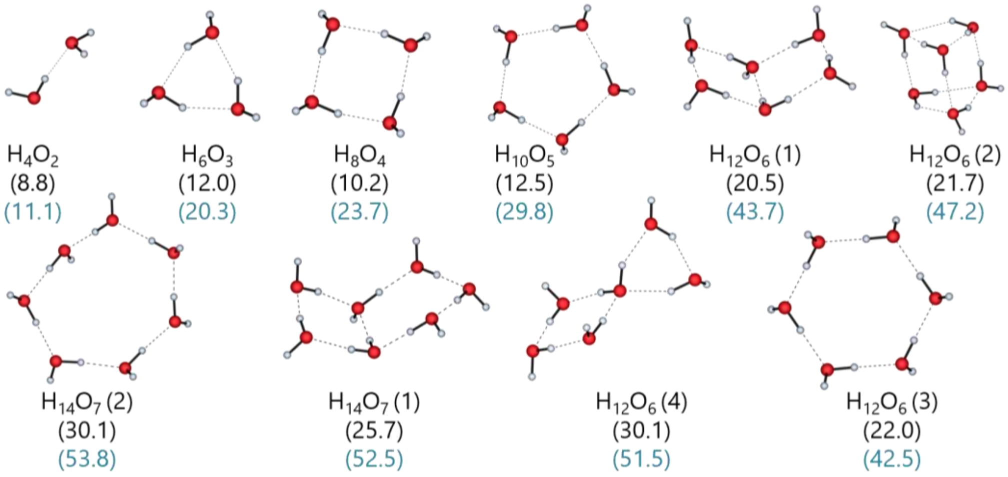

Figure S19: Optimized DSD-BLYP/def2-TZVPP gas-phase structures of the neutral water clusters $(H_2O)_n$ for n = 1–7 with the gas-phase clustering energies $\Delta_g G°$ from the DSD-BLYP /def2-TZVPP optimization (black) and the subsequent DLPNO-CCSD(T)/CBS single-point (blue) on the optimized structures. All energies are provided in kJ mol^{-1}.

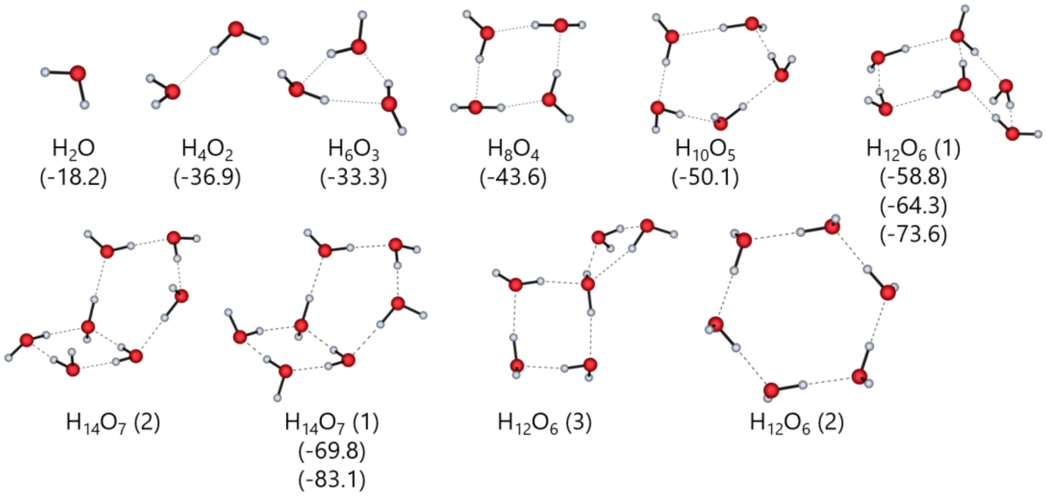

Figure S20: Optimized DSD-BLYP/def2-TZVPP CPCM structures of the neutral water clusters $H_2O)_n$ for n = 1–7 with the standard solvation energies $\Delta_{solv}G°$ from the DSD-BLYP /def2-TZVPP optimization. All energies are provided in kJ mol^{-1}.

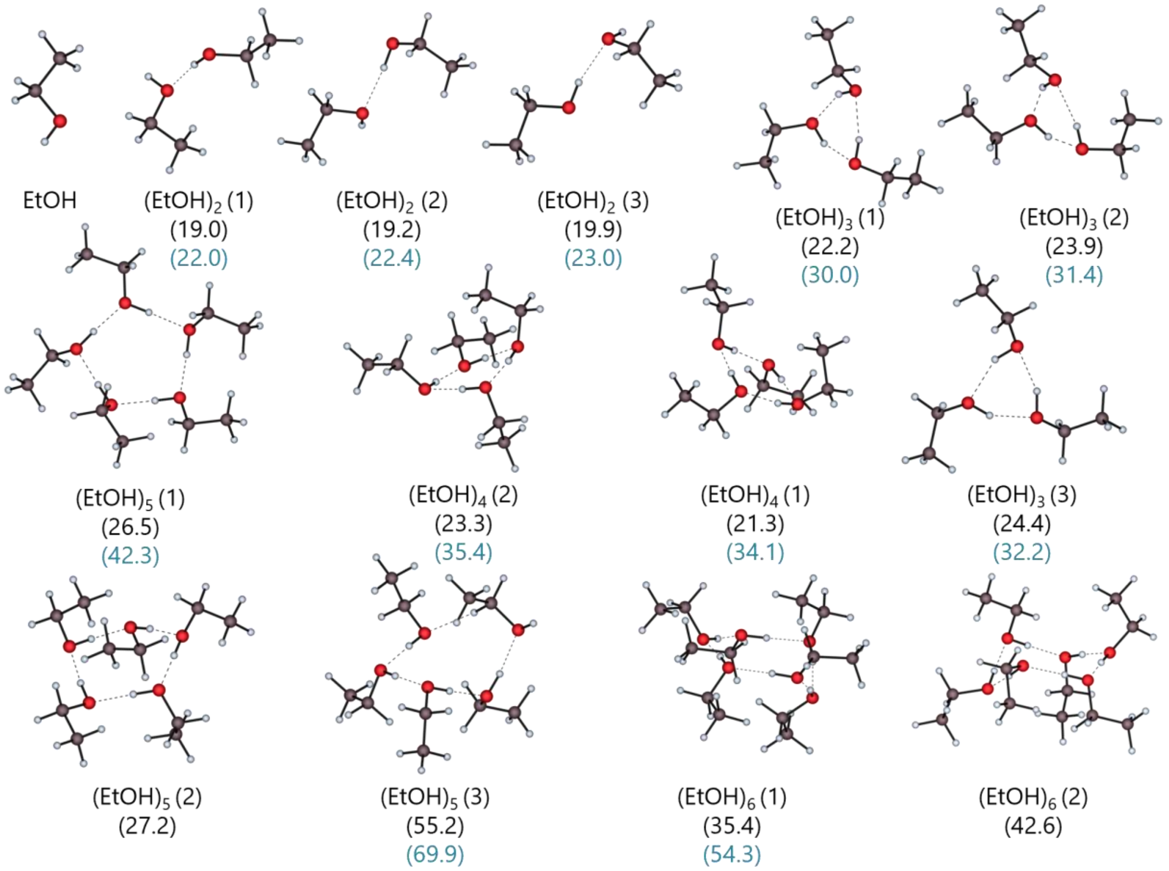

Figure S21: Optimized DSD-BLYP/def2-TZVPP gas-phase structures of the neutral ethanol clusters $(EtOH)_n$ for n = 1–6 with the gas-phase clustering energies $\Delta_gG°$ from the DSD-BLYP /def2-TZVPP optimization (black) and the subsequent DLPNO-CCSD(T)/CBS single-point (blue) on the optimized structures. All energies are provided in kJ mol^{-1}.

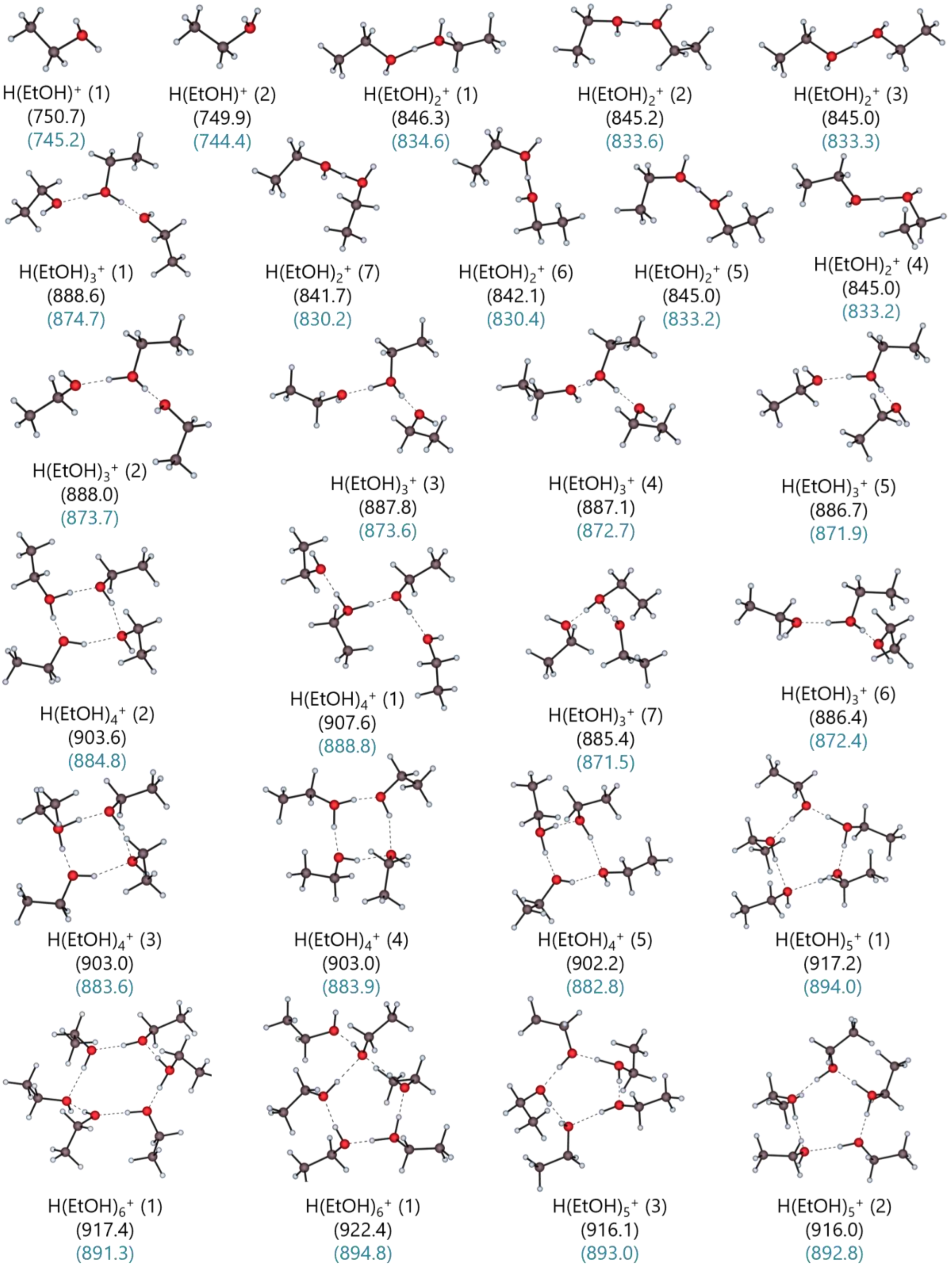

Figure S22: Optimized DSD-BLYP/def2-TZVPP gas-phase structures of the protonated ethanol clusters $H(EtOH)_n^+$ for n = 1–6 with the gas-phase clustering energies $\Delta_g G°$ from the DSD-BLYP /def2-TZVPP optimization (black) and the subsequent DLPNO-CCSD(T)/CBS single-point (blue) on the optimized structures. All energies are provided in kJ mol^{-1}.

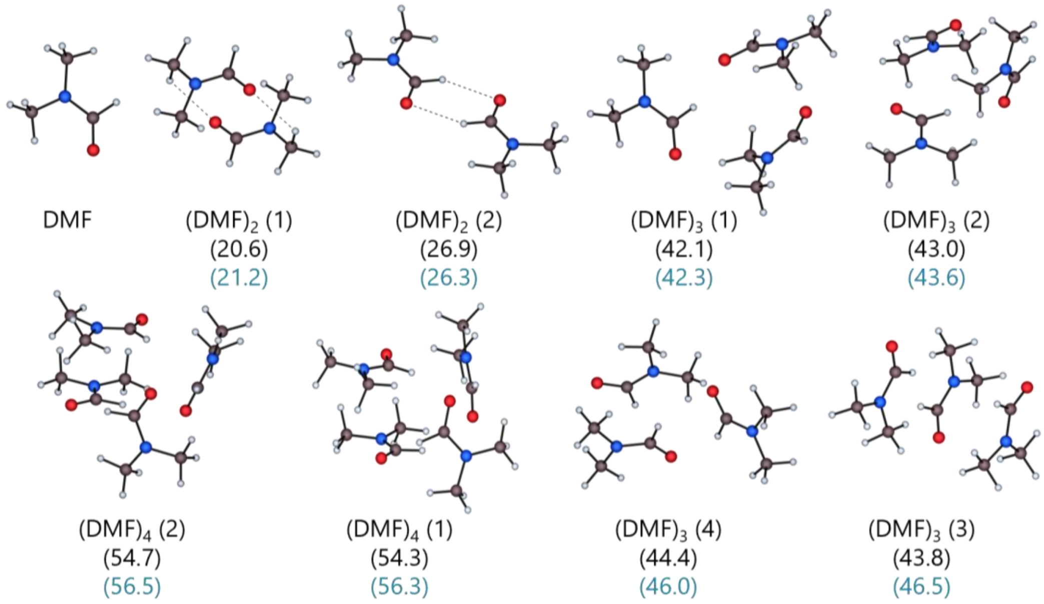

Figure S23: Optimized DSD-BLYP/def2-TZVPP gas-phase structures of the neutral DMF clusters $(DMF)_n$ for n = 1–4 with the gas-phase clustering energies $\Delta_g G°$ from the DSD-BLYP /def2-TZVPP optimization (black) and the subsequent DLPNO-CCSD(T)/CBS single-point (blue) on the optimized structures. All energies are provided in kJ mol^{-1}.

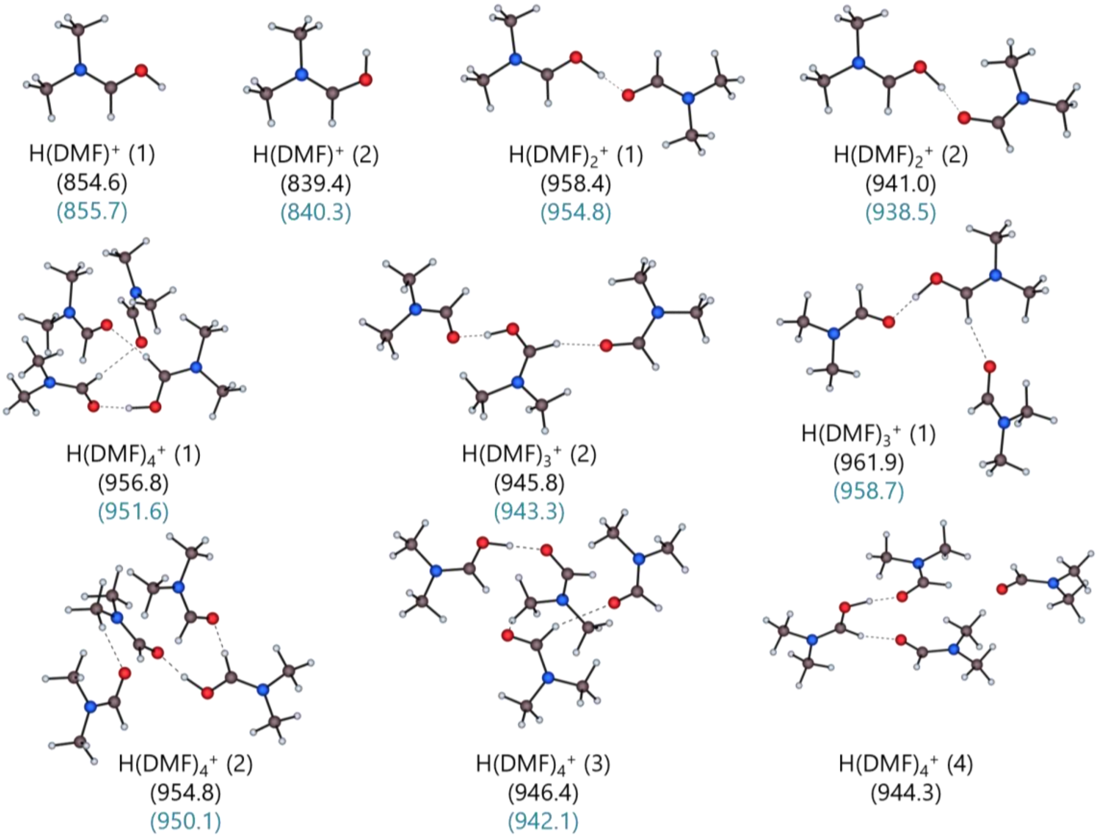

Figure S24: Optimized DSD-BLYP/def2-TZVPP gas-phase structures of the protonated DMF clusters $H(DMF)_n^+$ for n = 1–4 with the gas-phase clustering energies $\Delta_g G°$ from the DSD-BLYP /def2-TZVPP optimization (black) and the subsequent DLPNO-CCSD(T)/CBS single-point (blue) on the optimized structures. All energies are provided in kJ mol^{-1}.

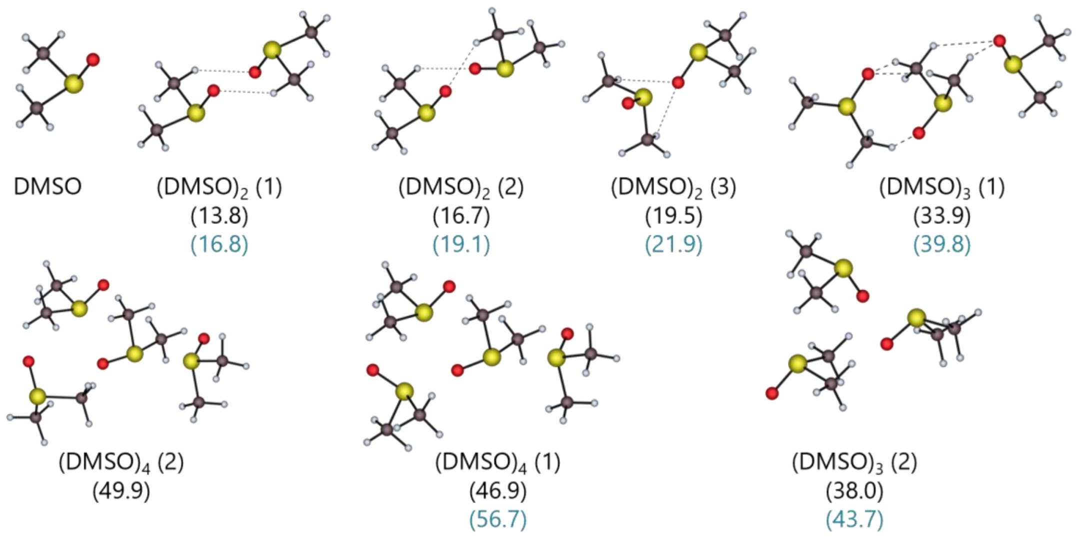

Figure S25: Optimized DSD-BLYP/def2-TZVPP gas-phase structures of the neutral DMSO clusters (DMSO)$_n$ for n = 1–4 with the gas-phase clustering energies $\Delta_g G°$ from the DSD-BLYP /def2-TZVPP optimization (black) and the subsequent DLPNO-CCSD(T)/CBS single-point (blue) on the optimized structures. All energies are provided in kJ mol^{-1}.

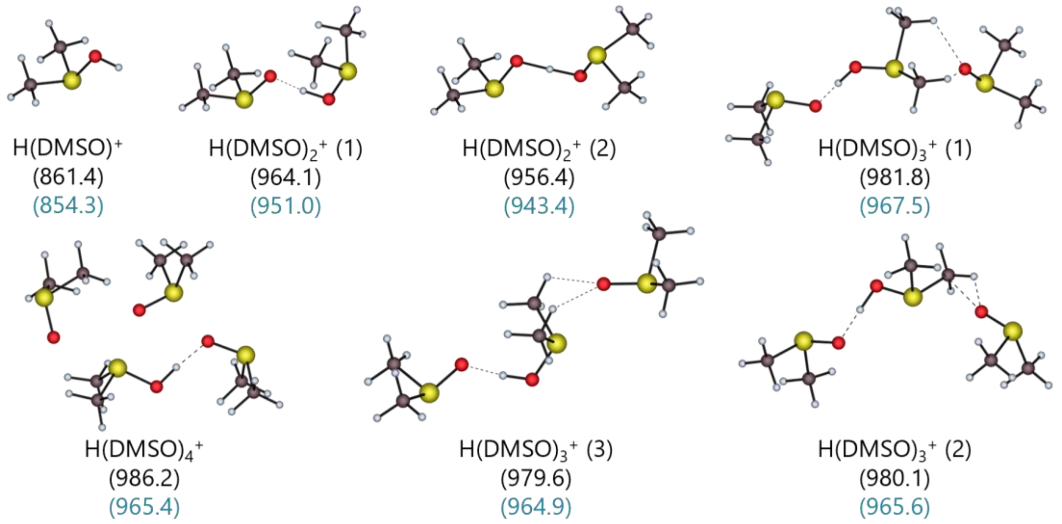

Figure S26: Optimized DSD-BLYP/def2-TZVPP gas-phase structures of the protonated DMSO clusters H(DMSO)$_n^+$ for n = 1–4 with the gas-phase clustering energies $\Delta_g G°$ from the DSD-BLYP /def2-TZVPP optimization (black) and the subsequent DLPNO-CCSD(T)/CBS single-point (blue) on the optimized structures. All energies are provided in kJ mol^{-1}.

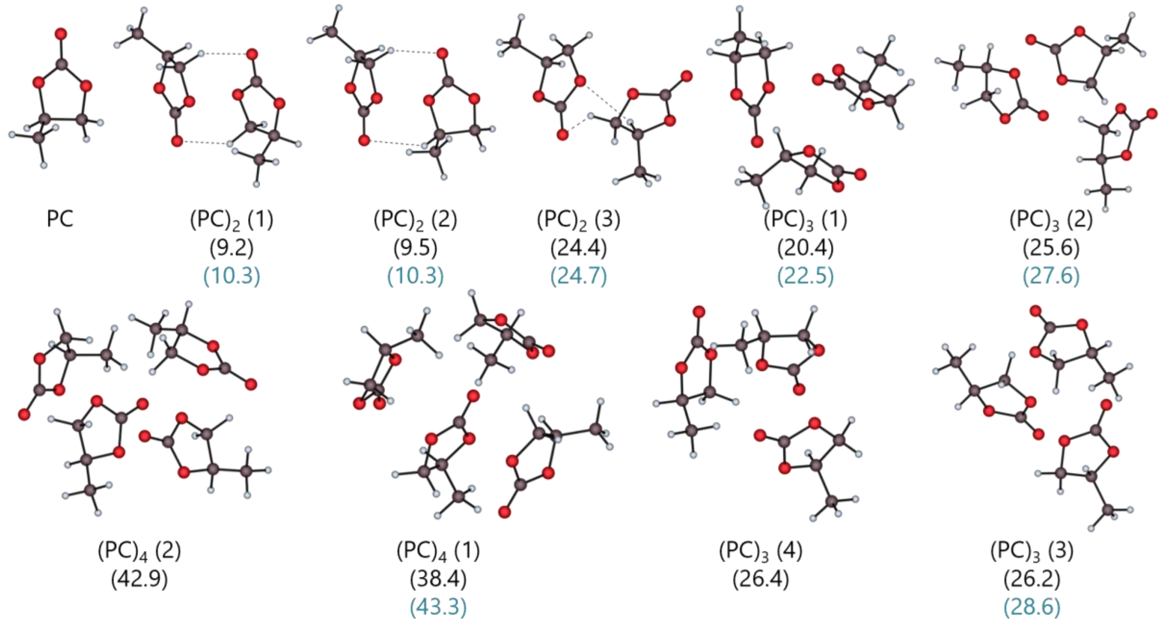

Figure S27: Optimized DSD-BLYP/def2-TZVPP gas-phase structures of the neutral PC clusters (PC)$_n$ for n = 1–4 with the gas-phase clustering energies $\Delta_g G°$ from the DSD-BLYP /def2-TZVPP optimization (black) and the subsequent DLPNO-CCSD(T)/CBS single-point (blue) on the optimized structures. All energies are provided in kJ mol^{-1}.

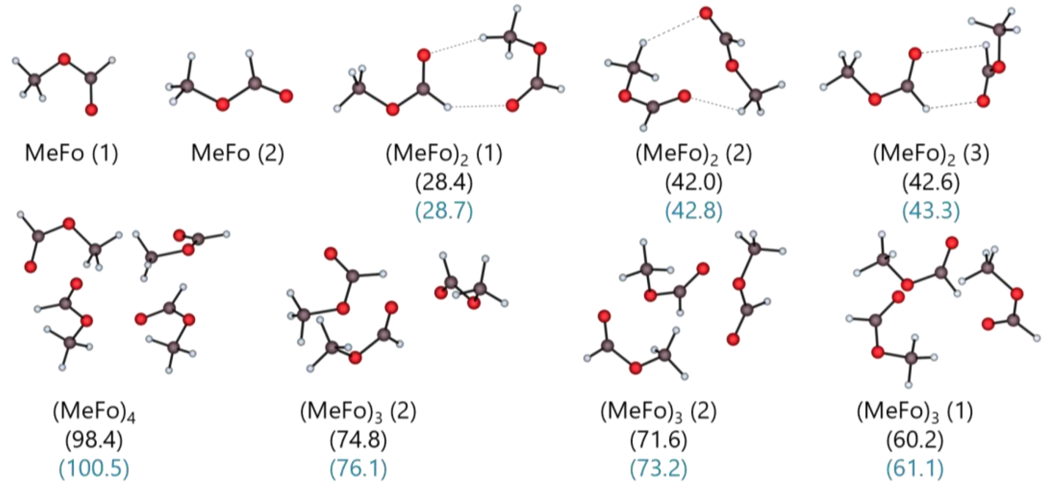

Figure S28: Optimized DSD-BLYP/def2-TZVPP gas-phase structures of the neutral MeFo clusters (MeFo)$_n$ for n = 1–4 with the gas-phase clustering energies $\Delta_g G°$ from the DSD-BLYP /def2-TZVPP optimization (black) and the subsequent DLPNO-CCSD(T)/CBS single-point (blue) on the optimized structures. All energies are provided in kJ mol^{-1}.

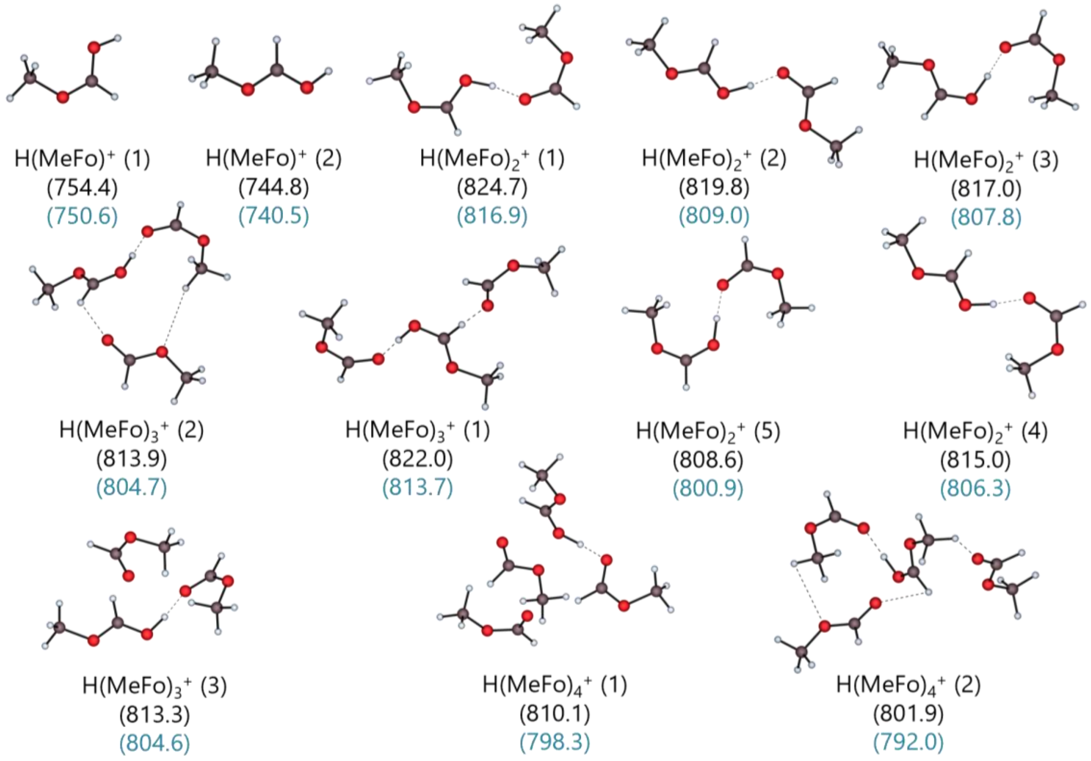

Figure S29: Optimized DSD-BLYP/def2-TZVPP gas-phase structures of the protonated MeFo clusters $H(MeFo)_n^+$ for n = 1–4 with the gas-phase clustering energies $\Delta_g G°$ from the DSD-BLYP /def2-TZVPP optimization (black) and the subsequent DLPNO-CCSD(T)/CBS single-point (blue) on the optimized structures. All energies are provided in kJ mol^{-1}.

3.3 Gas-Phase Energies and Solvation Energies of the Clusters

This section lists all calculated gas-phase and solvation energies for each cluster that are required to complete the monomer and the cluster cycle. Some CPCM structures exhibited minor imaginary modes caused by numerical inaccuracies and are marked as such. Because only the electronic energy from the CPCM calculations was used for the solvation energy calculation, performing frequency calculations are not required.

3.3.1 Water Clusters

Table S8: Calculated gas-phase clustering energies $\Delta_g G°$ (at 1 bar pressure and 298.15 K) according to the monomer and the cluster cycle as well as the solvation energies $\Delta_{solv}G°$ under standard conditions (1 bar gas to 1 mol L^{-1} solution) of the protonated water clusters, labeled by their solvent number *n* and the isomer number in brackets (*i*). The energies were calculated at the DSD-BLYP/def2-TZVPP level of theory, followed by a DLPNO-CCSD(T)/CBS single-point calculation on the optimized DSD-BLYP/def2-TZVPP structure. All energies are given in kJ mol^{-1}.

	DSD-BLYP/def2-TZVPP			DLPNO-CCSD(T)/CBS		
n (*i*)	$\Delta_g G°$(monomer)	$\Delta_g G°$(cluster)	$\Delta_{solv}G°$	$\Delta_g G°$(monomer)	$\Delta_g G°$(cluster)	$\Delta_{solv}G°$
1	-663.35	-663.35	-360.86	-657.00	-657.00	-360.83
2	-779.15	-787.98	-300.84	-763.32	-774.44	-301.01
3	-839.10	-851.11	-267.46	-818.56	-838.85	-267.60
4	-879.19	-889.42	-245.71	-854.68	-878.34	-245.35
5 (1)	-899.90	-912.44	-245.07	-871.26	-901.08	-242.87
5 (2)	-897.57	-910.12	-245.05	-868.88	-898.70	-242.90
5 (3)	-892.78	-905.33	-241.28	-863.29	-893.11	-239.93
6 (1)	-915.72	-936.27	-245.99	-882.72	-926.41	-243.48
6 (2)	-912.54	-933.08	-244.05	-880.23	-923.92	-240.84
6 (3)	-910.28	-930.82	-243.74	-877.87	-921.56	-240.63
6 (4)	-906.29	-926.83	-238.38	-872.47	-916.16	-236.67
7 (1)	-920.12	-945.77	-254.72	-884.46	-936.92	-247.27
7 (2)	-916.15	-941.80	-245.50	-877.71	-930.18	-240.81
7 (3)	-910.62	-936.27	-250.28	-872.99	-925.45	-244.79
7 (4)	-902.28	-927.93	-237.87	-861.79	-914.26	-235.24

Table S9: Calculated gas-phase interaction energies $\Delta_{int}G°$ (at 1 bar pressure and 298.15 K) and solvation energies $\Delta_{solv}G°$ under standard conditions (1 bar gas to 1 mol L^{-1} solution) of the neutral water clusters $(H_2O)_n$, labeled by their solvent number *n* and the isomer number in brackets (*i*). The energies were calculated at the DSD-BLYP/def2-TZVPP level of theory and a subsequent DLPNO-CCSD(T)/CBS single-point calculation on the optimized DSD-BLYP/def2-TZVPP structure. Only the isomer labeled with (1) was used for the calculation of the proton solvation energies through the cluster cycle. All energies are given in kJ mol^{-1}.

	DSD-BLYP/def2-TZVPP		DLPNO-CCSD(T)/CBS	
n (*i*)	$\Delta_{int}G°$	$\Delta_{solv}G°$	$\Delta_{int}G°$	$\Delta_{solv}G°$
1		-18.22		-17.59
2	8.83	-36.94	11.12	-35.71
3	12.02	-33.35	20.29	-32.24
4	10.22	-43.57	23.66	-41.98
5 (1)	12.54	-50.11	29.81	-48.77
6 (1)	20.54	-58.84	43.69	-57.44
6 (2)	21.73	-54.46	47.22	-55.40
6 (3)	21.95	-64.35	42.53	-60.38
6 (4)	30.07	-73.61	51.46	-70.46
7 (1)	25.65	-69.79	52.47	-67.96
7 (2)	30.15	-83.08	53.81	-78.10

Table S10: Calculated DSD-BLYP/def2-TZVPP gas-phase energies in Hartree (1 H = 2625.4996 kJ mol^{-1}) of the protonated water clusters labeled with their solvent number *n* and the isomer number in brackets (*i*). E_{tot}. refers to the total electronic energy. The enthalpy *H*, the Gibbs energy *G*, the entropy (already multiplied with the temperature $T·S$) and the different contributions to the entropy (translational S_{trans}, vibrational S_{vib}, and rotational entropy S_{rot}) are calculated at 298.15 K and 1 bar pressure. The rotational entropy was selected according to the symmetry number of the determined point group (pg).

n (*i*)	pg	E_{tot}	*H*	S_{trans}	S_{vib}	S_{rot}	$T·S$	G
1	C_{3v}	-76.661300	-76.622666	0.016521	0.000085	0.005327	0.021934	-76.644599
2	C_2	-153.104360	-153.041047	0.017465	0.002940	0.009180	0.029585	-153.070632
3	C_1	-229.529335	-229.436579	0.018026	0.008820	0.011966	0.038812	-229.475390
4	C_3	-305.947992	-305.826586	0.018427	0.015401	0.012173	0.046001	-305.872588
5 (1)	C_1	-382.356589	-382.207595	0.018739	0.022050	0.014016	0.054806	-382.262401
5 (2)	C_1	-382.356601	-382.207524	0.018739	0.021298	0.013954	0.053991	-382.261515
5 (3)	C_s	-382.358034	-382.208058	0.018739	0.019248	0.013646	0.051633	-382.259691
6 (1)	C_1	-458.762822	-458.587720	0.018995	0.029140	0.014499	0.062634	-458.650354
6 (2)	C_1	-458.763563	-458.586583	0.018995	0.028868	0.014696	0.062558	-458.649141
6 (3)	C_1	-458.763681	-458.586643	0.018995	0.028040	0.014602	0.061637	-458.648280
6 (4)	C_1	-458.765723	-458.588089	0.018995	0.025412	0.014264	0.058671	-458.646760
7 (1)	C_1	-535.169291	-534.964265	0.019211	0.035221	0.015259	0.069691	-535.033956
7 (2)	C_1	-535.172805	-534.967166	0.019211	0.031273	0.014792	0.065276	-535.032442
7 (3)	C_1	-535.170983	-534.965412	0.019211	0.030999	0.014713	0.064923	-535.030335
7 (4)	C_3	-535.175709	-534.968665	0.019211	0.026010	0.013274	0.058495	-535.027160

Table S11: Calculated DSD-BLYP/def2-TZVPP gas-phase energies in Hartree (1 H = 2625.4996 kJ mol^{-1}) of the neutral water clusters under a pressure of 1 bar, labeled with their solvent number *n* and the isomer number in brackets (*i*). The energies listed are the enthalpy *H*, the Gibbs energy *G*, the entropy (already multiplied with the temperature *T*·*S*) and the different contributions to the entropy (translational S_{trans}, vibrational S_{vib}, and rotational entropy S_{rot}) at 298.15 K, along with the total electronic energy E_{tot}. The rotational entropy was selected according to the symmetry number of the determined point group (pg).

n (*i*)	pg	E_{tot}	*H*	S_{trans}	S_{vib}	S_{rot}	*T*·*S*	*G*
1	C_{2v}	-76.385885	-76.360515	0.016444	0.000003	0.004965	0.021411	-76.381926
2	C_s	-152.780563	-152.727210	0.017425	0.005821	0.010030	0.033277	-152.760487
3	C_1	-229.185646	-229.103501	0.018000	0.007788	0.011912	0.037700	-229.141201
4	C_i	-305.590525	-305.480207	0.018407	0.012364	0.012833	0.043604	-305.523810
5 (1)	C_1	-381.993031	-381.854659	0.018723	0.017836	0.013635	0.050193	-381.904852
6 (1)	C_1	-458.395735	-458.228711	0.018981	0.021966	0.014073	0.055021	-458.283732
6 (2)	C_1	-458.397403	-458.229875	0.018981	0.020667	0.013754	0.053402	-458.283278
6 (3)	S_6	-458.393639	-458.227208	0.018981	0.023721	0.013285	0.055987	-458.283196
6 (4)	C_1	-458.390108	-458.223186	0.018981	0.023708	0.014226	0.056915	-458.280102
7 (1)	C_1	-534.796579	-534.601597	0.019200	0.028310	0.014606	0.062116	-534.663712
7 (2)	C_1	-534.791516	-534.597194	0.019200	0.030749	0.014857	0.064806	-534.661999

Table S12: Calculated DLPNO-CCSD(T)/CBS electronic gas-phase energy E_{tot}. in Hartree (1 H = 2625.4996 kJ mol^{-1}) of the protonated water clusters under a pressure of 1 bar, labeled with their solvent number *n* and the isomer number in brackets (*i*). The Gibbs energy *G* was obtained with the thermal and entropic corrections from the respective DSD-BLYP/def2-TZVPP optimization at 298.15 K and 1 bar pressure.

n (*i*)	pg	E_{tot}	*G*
1	C_{3v}	-76.649535	-76.632835
2	C_2	-153.079639	-153.045912
3	C_1	-229.493476	-229.439531
4	C_3	-305.901275	-305.825871
5 (1)	C_1	-382.298955	-382.204767
5 (2)	C_1	-382.298946	-382.203860
5 (3)	C_s	-382.300075	-382.201731
6 (1)	C_1	-458.694179	-458.581711
6 (2)	C_1	-458.695183	-458.580761
6 (3)	C_1	-458.695263	-458.579862
6 (4)	C_1	-458.696772	-458.577808
7 (1)	C_1	-535.090288	-534.954953
7 (2)	C_1	-535.092747	-534.952384
7 (3)	C_1	-535.091231	-534.950583
7 (4)	C_3	-535.094869	-534.946320

Table S13: Calculated DLPNO-CCSD(T)/CBS electronic gas-phase energy E_{tot}. in Hartree (1 H = 2625.4996 kJ mol^{-1}) of the neutral water clusters under a pressure of 1 bar, labeled with their solvent number *n* and the isomer number in brackets (*i*). The Gibbs energy *G* was obtained with the thermal and entropic corrections from the respective DSD-BLYP/def2-TZVPP optimization at 298.15 K and 1 bar pressure.

n (*i*)	pg	E_{tot}	*G*
1	C_{2v}	-76.376540	-76.372580
2	C_s	-152.761002	-152.740926
3	C_1	-229.154458	-229.110013
4	C_i	-305.548025	-305.481310
5 (1)	C_1	-381.939726	-381.851548
6 (1)	C_1	-458.330846	-458.218843
6 (2)	C_1	-458.331624	-458.217498
6 (3)	S_6	-458.329726	-458.219282
6 (4)	C_1	-458.325889	-458.215882
7 (1)	C_1	-534.720947	-534.588080
7 (2)	C_1	-534.717084	-534.587568

Table S14: Total electronic energies E_{tot} calculated with the CPCM of the protonated water clusters, labeled with their solvent number *n* and the isomer number in brackets (*i*). The energies were obtained from the DSD-BLYP/def2-TZVPP optimization and the DLPNO-CCSD(T)/CBS single-point calculation on the optimized structure in Hartree (1 H = 2625.4996 kJ mol^{-1}). Only the isomers labeled with (1) were used for further calculations of the standard solvation energies of the proton through the thermodynamic cycles. The solvation energies of the clusters were obtained through $\Delta_{solv}G° = E_{tot}(\text{CPCM}) – E_{tot}(\text{gas-phase})+7.96$.

n (*i*)	DSD-BLYP/def2-TZVPP<br>E_{tot}	DLPNO-CCSD(T)/CBS<br>E_{tot}
1	-76.801776	-76.790000
2	-153.221976	-153.197320
3	-229.634237	-229.598433
4	-306.044610	-305.997757
5 (1)	-382.452965	-382.394492
5 (2)	-382.449530	
5 (3)	-382.449367	
6 (1)	-458.859548	-382.391585
6 (2)	-458.857255	-458.789946
6 (3)	-458.854128	
6 (4)	-458.854043	-458.785254
7 (1)	-535.269341	-535.187498
7 (2)	-535.264227	
7 (3)	-535.263599	-535.182969
7 (4)	-535.258660	-535.178981

Table S15: Total electronic energies E_{tot} calculated with the CPCM of the neutral water clusters, labeled with their solvent number *n* and the isomer number in brackets (*i*). The energies were obtained from the DSD-BLYP/def2-TZVPP optimization and the DLPNO-CCSD(T)/CBS single-point calculation on the optimized structure in Hartree (1 H = 2625.4996 kJ mol^{-1}). Only the isomers labeled with (1) were used for further calculations of the standard solvation energies of the proton through the thermodynamic cycles. The solvation energies of the clusters were obtained through $\Delta_{solv}G° = E_{tot}(CPCM) - E_{tot}(gas\text{-}phase)+7.96$.

n (*i*)	DSD-BLYP/def2-TZVPP E_{tot}	DLPNO-CCSD(T)/CBS E_{tot}
1	-76.395855	-76.386271
2	-152.797664	-152.777636
3	-229.201381	-229.169769
4	-305.610153	-305.567046
5 (1)	-382.015147	-381.961335
6 (1)	-458.421178	-458.355756
6 (2)	-458.419579	
6 (3)	-458.418330	-458.354016
6 (4)	-458.415882	
7 (1)	-534.826192	-534.749864
7 (2)	-534.826174	-534.749870

Table S16: Calculated gas-phase clustering energies $\Delta_g G°$ (at 1 bar pressure and 298.15 K) according to the monomer and the cluster cycle as well as the solvation energies $\Delta_{solv}G°$ under standard conditions (1 bar gas to 1 mol L^{-1} solution) of water and the protonated water clusters, labeled by their solvent number *n* and the isomer number in brackets (*i*). The energies were calculated at the BP/def2-TZVPP level of theory, followed by a DLPNO-CCSD(T)/CBS single-point calculation on the optimized BP/def2-TZVPP structure. All energies are given in kJ mol^{-1}.

	BP/def2-TZVPP		DLPNO-CCSD(T)/CBS
n (*i*)	$\Delta_g G°$	$\Delta_{solv}G°$	$\Delta_g G°$
H_2O		-17.34	
1	-665.78	-360.07	-657.70
2	-789.42	-300.80	-761.41
3	-852.31	-268.39	-816.10
4	-891.10	-248.49	-851.13
5 (1)	-914.88	-239.67	-866.58
5 (2)	-913.24	-239.03	-864.68
5 (3)	-907.03	-235.92	-858.47
6 (1)	-929.84	-250.74	-874.60
6 (2)	-929.41	-250.54	-874.76
6 (3)	-927.45	-249.25	-872.34
6 (4)	-922.94	-243.25	-865.48
7 (1)	-938.05	-250.80	-869.33
7 (2)	-935.10	-264.62	-874.89
7 (3)	-929.90	-259.04	-866.52
7 (4)	-925.90	-245.21	-857.62

Table S17: Calculated BP/def2-TZVPP gas-phase energies in Hartree (1 H = 2625.4996 kJ mol^{-1}) of water and the protonated water clusters under a pressure of 1 bar, labeled with their solvent number *n* and the isomer number in brackets (*i*). The energies listed are the enthalpy *H*, the Gibbs energy *G*, the entropy (already multiplied with the temperature $T{\cdot}S$) and the different contributions to the entropy (translational S_{trans}, vibrational S_{vib}, and rotational entropy S_{rot}) at 298.15 K, along with the total electronic energy E_{tot}. The rotational entropy was selected according to the symmetry number of the determined point group (pg).

n (*i*)	pg	E_{tot}	*H*	S_{trans}	S_{vib}	S_{rot}	$T{\cdot}S$	*G*

Water	C_{3v}	-76.468684	-76.444169	0.016444	0.000004	0.005002	0.021449	-76.465618
1	C_{3v}	-76.744673	-76.707245	0.016521	0.000092	0.005361	0.021974	-76.729218
2	C_2	-153.274446	-153.212384	0.017465	0.002861	0.009220	0.029546	-153.241929
3	C_1	-229.782744	-229.693334	0.018026	0.008176	0.011965	0.038167	-229.731501
4	C_3	-306.283921	-306.166791	0.018427	0.014515	0.012159	0.045101	-306.211893
5 (1)	C_1	-382.776366	-382.632857	0.018739	0.020971	0.014001	0.053710	-382.686567
5 (2)	C_1	-382.776608	-382.632935	0.018739	0.020354	0.013917	0.053010	-382.685945
5 (3)	C_s	-382.777794	-382.633223	0.018739	0.017989	0.013630	0.050357	-382.683581
6 (1)	C_1	-459.266651	-459.096922	0.018995	0.027498	0.014471	0.060963	-459.157885
6 (2)	C_1	-459.266731	-459.096291	0.018995	0.027740	0.014694	0.061429	-459.157720
6 (3)	C_1	-459.267219	-459.096607	0.018995	0.026829	0.014543	0.060366	-459.156973
6 (4)	C_1	-459.269506	-459.098394	0.018995	0.023612	0.014257	0.056863	-459.155257
7 (1)	C_1	-535.761268	-535.563320	0.019211	0.029342	0.014757	0.063310	-535.626630
7 (2)	C_3	-535.756005	-535.558363	0.019211	0.033769	0.014166	0.067146	-535.625509
7 (3)	C_1	-535.758133	-535.560065	0.019211	0.029571	0.014680	0.063462	-535.623526
7 (4)	C_1	-535.763397	-535.564048	0.019211	0.024458	0.014286	0.057955	-535.622003

Table S18: Total electronic energies E_{tot} calculated with the CPCM of the protonated water clusters and water, labeled with their solvent number n and the isomer number in brackets (i). The energies were obtained from the BP/def2-TZVPP optimization in Hartree (1 H = 2625.4996 kJ mol^{-1}). Only the isomers labeled with (1) were used for further calculations of the standard solvation energies of the proton through the thermodynamic cycles. The solvation energies of the clusters were obtained through $\Delta_{solv}G° = E_{tot}(CPCM) - E_{tot}(gas\text{-}phase)+7.96$.

n (i)	E_{tot}
Water	-76.478319
1	-76.884848
2	-153.392045
3	-229.887998
4	-306.381597
5	-382.870683
6 (1)	-459.365186
6 (2)	-459.359268
6 (3)	-459.359268
7 (1)	-535.859826
7 (2)	-535.854423
7 (3)	-535.853532
7 (4)	-535.847531

Table S19: Calculated DSD-BLYP/def2-QZVPP gas-phase energies in Hartree (1 H = 2625.4996 kJ mol^{-1}) of water and the protonated water clusters under a pressure of 1 bar, labeled with their solvent number *n* and the isomer number in brackets (*i*). The energies listed are the enthalpy *H*, the Gibbs energy *G*, the entropy (already multiplied with the temperature *T·S*) and the different contributions to the entropy (translational S_{trans}, vibrational S_{vib}, and rotational entropy S_{rot}) at 298.15 K, along with the total electronic energy E_{tot}. The rotational entropy was selected according to the symmetry number of the determined point group (pg).

n (*i*)	pg	E_{tot}	*H*	S_{trans}	S_{vib}	S_{rot}	*T·S*	*G*
Water	C_{3v}	-76.400910	-76.375556	0.016444	0.000003	0.004962	0.021409	-76.396964
1	C_{3v}	-76.674474	-76.635853	0.016521	0.000085	0.005325	0.021931	-76.657784
2	C_2	-153.131036	-153.067920	0.017465	0.003009	0.009180	0.029654	-153.097574
3	C_1	-229.570096	-229.477504	0.018026	0.009250	0.011970	0.039245	-229.516749
4	C_3	-306.002871	-305.881660	0.018427	0.015621	0.012178	0.046225	-305.927886
5	C_1	-382.425683	-382.276898	0.018739	0.022165	0.014016	0.054920	-382.331818
6	C_1	-458.846951	-458.670218	0.018995	0.028622	0.014697	0.062313	-458.732531
7	C_s	-535.267008	-535.062280	0.019211	0.035411	0.015266	0.069889	-535.132169

Table S20: Calculated DSD-BLYP/cc-pVQZ gas-phase energies in Hartree (1 H = 2625.4996 kJ mol^{-1}) of water and the protonated water clusters under a pressure of 1 bar, labeled with their solvent number *n* and the isomer number in brackets (*i*). The energies listed are the enthalpy *H*, the Gibbs energy *G*, the entropy (already multiplied with the temperature *T·S*) and the different contributions to the entropy (translational S_{trans}, vibrational S_{vib}, and rotational entropy S_{rot}) at 298.15 K, along with the total electronic energy E_{tot}. The rotational entropy was selected according to the symmetry number of the determined point group (pg).

n (*i*)	pg	E_{tot}	*H*	S_{trans}	S_{vib}	S_{rot}	*T·S*	*G*
Water	C_{3v}	-76.398591	-76.373234	0.016444	0.000003	0.004962	0.021409	-76.394643
1	C_{3v}	-76.673478	-76.634878	0.016521	0.000085	0.005326	0.021932	-76.656809
2	C_2	-153.128938	-153.065785	0.017465	0.002971	0.009181	0.029616	-153.095401
3	C_1	-229.566588	-229.473988	0.018026	0.009052	0.011967	0.039046	-229.513034
4	C_3	-305.997921	-305.876693	0.018427	0.015520	0.012174	0.046120	-305.922814
5	C_1	-382.419199	-382.270429	0.018739	0.022822	0.014019	0.055580	-382.326009
6	C_1	-458.838885	-458.662144	0.018995	0.029113	0.014692	0.062800	-458.724944
7	C_s	-535.257327	-535.052579	0.019211	0.035416	0.015258	0.069885	-535.122464

Table S21: Calculated DSD-BLYP/cc-pV5Z gas-phase energies in Hartree (1 H = 2625.4996 kJ mol^{-1}) of water and the protonated water clusters under a pressure of 1 bar, labeled with their solvent number *n* and the isomer number in brackets (*i*). The energies listed are the enthalpy *H*, the Gibbs energy *G*, the entropy (already multiplied with the temperature $T{\cdot}S$) and the different contributions to the entropy (translational S_{trans}, vibrational S_{vib}, and rotational entropy S_{rot}) at 298.15 K, along with the total electronic energy E_{tot}. The rotational entropy was selected according to the symmetry number of the determined point group (pg).

n (*i*)	pg	E_{tot}	*H*	S_{trans}	S_{vib}	S_{rot}	$T{\cdot}S$	*G*
Water	C_{3v}	-76.405471	-76.380138	0.016444	0.000003	0.004962	0.021409	-76.401546
1	C_{3v}	-76.678625	-76.640022	0.016521	0.000085	0.005325	0.021931	-76.661953
2	C_2	-153.139547	-153.076485	0.017465	0.003033	0.009180	0.029678	-153.106163
3	C_1	-229.583042	-229.490517	0.018026	0.009367	0.011970	0.039363	-229.529880
4	C_3	-306.020249	-305.899128	0.018427	0.015681	0.011906	0.046014	-305.945142
5	C_1	-382.447511	-382.299768	0.018739	0.019602	0.014016	0.052356	-382.352124
6[a]	C_1	-458.873221	-458.697577	0.018995	0.026539	0.014708	0.060242	-458.757819
7	C_s	-535.297784	-535.093219	0.019211	0.035550	0.015271	0.070032	-535.163251

[a] imaginary mode

Table S22: Calculated DSD-PBEP86/def2-TZVPP gas-phase energies in Hartree (1 H = 2625.4996 kJ mol^{-1}) of water and the protonated water clusters under a pressure of 1 bar, labeled with their solvent number *n* and the isomer number in brackets (*i*). The energies listed are the enthalpy *H*, the Gibbs energy *G*, the entropy (already multiplied with the temperature $T{\cdot}S$) and the different contributions to the entropy (translational S_{trans}, vibrational S_{vib}, and rotational entropy S_{rot}) at 298.15 K, along with the total electronic energy E_{tot}. The rotational entropy was selected according to the symmetry number of the determined point group (pg).

n (*i*)	pg	E_{tot}	*H*	S_{trans}	S_{vib}	S_{rot}	$T{\cdot}S$	*G*
Water	C_{3v}	-76.348251	-76.322914	0.016444	0.000003	0.004968	0.021415	-76.344328
1	C_{3v}	-76.624573	-76.585933	0.016521	0.000081	0.005330	0.021932	-76.607865
2	C_2	-153.030262	-152.966874	0.017465	0.002909	0.009184	0.029558	-152.996432
3	C_1	-229.417598	-229.324967	0.018026	0.008780	0.011962	0.038768	-229.363735
4	C_3	-305.798631	-305.677374	0.018427	0.015275	0.012166	0.045868	-305.723242
5	C_1	-382.169772	-382.020978	0.018739	0.022361	0.014008	0.055108	-382.076086
6	C_1	-458.539247	-458.363454	0.018995	0.026218	0.014687	0.059899	-458.423353
7	C_s	-534.907453	-534.702685	0.019211	0.035287	0.015246	0.069743	-534.772428

Table S23: Calculated B3LYP/def2-TZVPP gas-phase energies in Hartree (1 H = 2625.4996 kJ mol^{-1}) of water and the protonated water clusters under a pressure of 1 bar, labeled with their solvent number *n* and the isomer number in brackets (*i*). The energies listed are the enthalpy *H*, the Gibbs energy *G*, the entropy (already multiplied with the temperature *T*·*S*) and the different contributions to the entropy (translational S_{trans}, vibrational S_{vib}, and rotational entropy S_{rot}) at 298.15 K, along with the total electronic energy E_{tot}. The rotational entropy was selected according to the symmetry number of the determined point group (pg).

n (*i*)	pg	E_{tot}	*H*	S_{trans}	S_{vib}	S_{rot}	*T*·*S*	*G*
Water	C_{3v}	-76.430325	-76.405253	0.016444	0.000003	0.004973	0.021420	-76.426672
1	C_{3v}	-76.705866	-76.667754	0.016521	0.000103	0.005337	0.021960	-76.689714
2	C_2	-153.195577	-153.132877	0.017465	0.003015	0.009191	0.029671	-153.162548
3	C_1	-229.665773	-229.574322	0.018026	0.008917	0.011967	0.038909	-229.613232
4	C_3	-306.129317	-306.009480	0.018427	0.015404	0.012180	0.046011	-306.055491
5	C_1	-382.583163	-382.436101	0.018739	0.021904	0.014020	0.054663	-382.490764
6	C_1	-459.035253	-458.860538	0.018995	0.028652	0.014701	0.062348	-458.922885
7	C_s	-535.486015	-535.283575	0.019211	0.035785	0.015254	0.070250	-535.353825

Table S24: Calculated BLYP/def2-TZVPP gas-phase energies in Hartree (1 H = 2625.4996 kJ mol^{-1}) of water and the protonated water clusters under a pressure of 1 bar, labeled with their solvent number *n* and the isomer number in brackets (*i*). The energies listed are the enthalpy *H*, the Gibbs energy *G*, the entropy (already multiplied with the temperature *T*·*S*) and the different contributions to the entropy (translational S_{trans}, vibrational S_{vib}, and rotational entropy S_{rot}) at 298.15 K, along with the total electronic energy E_{tot}. The rotational entropy was selected according to the symmetry number of the determined point group (pg).

n (*i*)	pg	E_{tot}	*H*	S_{trans}	S_{vib}	S_{rot}	*T*·*S*	*G*
Water	C_{3v}	-76.449873	-76.425503	0.016444	0.000004	0.005004	0.021452	-76.446955
1	C_{3v}	-76.723530	-76.686324	0.016521	0.000097	0.005364	0.021982	-76.708306
2	C_2	-153.233479	-153.172045	0.017465	0.002937	0.009226	0.029627	-153.201673
3	C_1	-229.722930	-229.633876	0.018026	0.008461	0.011987	0.038474	-229.672350
4	C_3	-306.205653	-306.088971	0.018427	0.014936	0.012187	0.045550	-306.134521
5	C_1	-382.679158	-382.536063	0.018739	0.021296	0.014019	0.054054	-382.590118
6	C_1	-459.150780	-458.980809	0.018995	0.027866	0.014694	0.061555	-459.042364
7	C_s	-535.621003	-535.424051	0.019211	0.034763	0.015240	0.069214	-535.493265

Table S25: Calculated TPSSh/def2-TZVPP gas-phase energies in Hartree (1 H = 2625.4996 kJ mol^{-1}) of water and the protonated water clusters under a pressure of 1 bar, labeled with their solvent number *n* and the isomer number in brackets (*i*). The energies listed are the enthalpy *H*, the Gibbs energy *G*, the entropy (already multiplied with the temperature $T{\cdot}S$) and the different contributions to the entropy (translational S_{trans}, vibrational S_{vib}, and rotational entropy S_{rot}) at 298.15 K, along with the total electronic energy E_{tot}. The rotational entropy was selected according to the symmetry number of the determined point group (pg).

n (*i*)	pg	E_{tot}	H	S_{trans}	S_{vib}	S_{rot}	$T{\cdot}S$	G
Water	C_{3v}	-76.459279	-76.434298	0.016444	0.000003	0.004981	0.021428	-76.455726
1	C_{3v}	-76.738290	-76.700074	0.016521	0.000083	0.005342	0.021946	-76.722020
2	C_2	-153.257139	-153.194332	0.017465	0.002891	0.009204	0.029559	-153.223891
3	C_1	-229.756219	-229.664802	0.018026	0.008327	0.011967	0.038320	-229.703122
4	C_3	-306.248481	-306.128806	0.018427	0.014699	0.012162	0.045288	-306.174094
5	C_1	-382.731233	-382.584458	0.018739	0.020967	0.013995	0.053701	-382.638160
6	C_1	-459.212172	-459.037887	0.018995	0.027533	0.014673	0.061201	-459.099087
7	C_s	-535.691733	-535.489821	0.019211	0.034370	0.015221	0.068802	-535.558623

Table S26: Calculated TPSS0/def2-TZVPP gas-phase energies in Hartree (1 H = 2625.4996 kJ mol^{-1}) of water and the protonated water clusters under a pressure of 1 bar, labeled with their solvent number *n* and the isomer number in brackets (*i*). The energies listed are the enthalpy *H*, the Gibbs energy *G*, the entropy (already multiplied with the temperature $T{\cdot}S$) and the different contributions to the entropy (translational S_{trans}, vibrational S_{vib}, and rotational entropy S_{rot}) at 298.15 K, along with the total electronic energy E_{tot}. The rotational entropy was selected according to the symmetry number of the determined point group (pg).

n (*i*)	pg	E_{tot}	H	S_{trans}	S_{vib}	S_{rot}	$T{\cdot}S$	G
Water	C_{3v}	-76.447529	-76.422083	0.016444	0.000003	0.004961	0.021407	-76.443491
1	C_{3v}	-76.727649	-76.688850	0.016521	0.000086	0.005324	0.021931	-76.710782
2	C_2	-153.234086	-153.170467	0.017465	0.002943	0.009182	0.029589	-153.200055
3	C_1	-229.721404	-229.628432	0.018026	0.008674	0.011956	0.038655	-229.667087
4	C_3	-306.201964	-306.080231	0.018427	0.014982	0.012159	0.045568	-306.125798
5	C_1	-382.672700	-382.523335	0.018739	0.021370	0.013998	0.054107	-382.577442
6	C_1	-459.141719	-458.964323	0.018995	0.028000	0.014679	0.061674	-459.025996
7	C_s	-535.609405	-535.403907	0.019211	0.034939	0.015232	0.069382	-535.473290

Table S27: Calculated M06/def2-TZVPP gas-phase energies in Hartree (1 H = 2625.4996 kJ mol^{-1}) of water and the protonated water clusters under a pressure of 1 bar, labeled with their solvent number *n* and the isomer number in brackets (*i*). The energies listed are the enthalpy *H*, the Gibbs energy *G*, the entropy (already multiplied with the temperature $T{\cdot}S$) and the different contributions to the entropy (translational S_{trans}, vibrational S_{vib}, and rotational entropy S_{rot}) at 298.15 K, along with the total electronic energy E_{tot}. The rotational entropy was selected according to the symmetry number of the determined point group (pg).

n (*i*)	pg	E_{tot}	*H*	S_{trans}	S_{vib}	S_{rot}	$T{\cdot}S$	*G*
Water	C_{3v}	-76.426741	-76.401284	0.016444	0.000003	0.004959	0.021406	-76.422690
1	C_{3v}	-76.704214	-76.665930	0.016521	0.000129	0.005324	0.021973	-76.687903
2	C_2	-153.188210	-153.123970	0.017465	0.003010	0.009171	0.029646	-153.153616
3	C_1	-229.651646	-229.559185	0.018026	0.010123	0.011980	0.040128	-229.599313
4	C_3	-306.110611	-305.989309	0.018427	0.016309	0.012204	0.046940	-306.036248
5	C_1	-382.559111	-382.410379	0.018739	0.022701	0.014047	0.055487	-382.465866
6	C_1	-459.006142	-458.829254	0.018995	0.029482	0.014735	0.063211	-458.892465
7	C_s	-535.452002	-535.246989	0.019211	0.036706	0.015294	0.071211	-535.318200

Table S28: Calculated wB97M-D3BJ/def2-TZVPP gas-phase energies in Hartree (1 H = 2625.4996 kJ mol^{-1}) of water and the protonated water clusters under a pressure of 1 bar, labeled with their solvent number *n* and the isomer number in brackets (*i*). The energies listed are the enthalpy *H*, the Gibbs energy *G*, the entropy (already multiplied with the temperature $T{\cdot}S$) and the different contributions to the entropy (translational S_{trans}, vibrational S_{vib}, and rotational entropy S_{rot}) at 298.15 K, along with the total electronic energy E_{tot}. The rotational entropy was selected according to the symmetry number of the determined point group (pg).

n (*i*)	pg	E_{tot}	*H*	S_{trans}	S_{vib}	S_{rot}	$T{\cdot}S$	*G*
Water	C_{3v}	-76.477782	-76.452435	0.016444	0.000003	0.004968	0.021415	-76.473850
1	C_{3v}	-76.755588	-76.717095	0.016521	0.000096	0.005332	0.021949	-76.739044
2	C_2	-153.291007	-153.227761	0.017465	0.003000	0.009187	0.029651	-153.257412
3	C_1	-229.807555	-229.715173	0.018026	0.009116	0.011970	0.039112	-229.754285
4	C_3	-306.317809	-306.196783	0.018427	0.015551	0.012183	0.046160	-306.242943
5	C_1	-382.818182	-382.669639	0.018739	0.022096	0.014027	0.054862	-382.724501
6	C_1	-459.316911	-459.140440	0.018995	0.028900	0.014714	0.062609	-459.203049
7	C_s	-535.814396	-535.609927	0.019211	0.036133	0.015276	0.070620	-535.680547

3.3.2 Methanol Clusters

Table S29: Calculated gas-phase clustering energies $\Delta_g G°$ (at 1 bar pressure and 298.15 K) according to the monomer and the cluster cycle as well as the solvation energies $\Delta_{solv}G°$ under standard conditions (1 bar gas to 1 mol L^{-1} solution) of the protonated $H(MeOH)_n^+$ clusters, labeled by their solvent number *n* and the isomer number in brackets (*i*). The energies were calculated at the DSD-BLYP/def2-TZVPP level of theory, followed by a DLPNO-CCSD(T)/CBS single-point calculation on the optimized DSD-BLYP/def2-TZVPP structure. All energies are given in kJ mol^{-1}.

	DSD-BLYP/def2-TZVPP			DLPNO-CCSD(T)/CBS		
n (*i*)	$\Delta_g G°$(monomer)	$\Delta_g G°$(cluster)	$\Delta_{solv}G°$	$\Delta_g G°$(monomer)	$\Delta_g G°$(cluster)	$\Delta_{solv}G°$
1	-727.43	-727.43	-301.98	-722.26	-722.26	-301.68
2 (1)	-830.49	-844.57	-244.45	-818.42	-835.33	-243.93
2 (2)	-830.38	-844.47	-244.46	-818.32	-835.23	-243.93
2 (3)	-828.15	-842.23	-245.37	-816.24	-833.14	-244.70
3 (1)	-879.68	-897.42	-211.87	-865.01	-890.46	-206.36
3 (2)	-877.79	-895.54	-211.71	-862.96	-888.42	-206.36
4 (1)	-904.44	-917.39	-194.46	-885.33	-911.00	-188.11
4 (2)	-904.02	-916.97	-194.27	-884.76	-910.43	-188.07
5 (1)	-912.81	-928.30	-181.76	-890.16	-921.90	-180.33
5 (2)	-912.80	-928.29	-182.41	-890.19	-921.92	-180.94
6 (1)	-925.09	-946.58	-177.13	-896.49	-937.82	-175.59
6 (2)	-919.04	-940.52	-182.96	-890.53	-931.86	-181.32
6 (3)	-914.32	-935.81	-194.26	-887.40	-928.73	-191.04

Table S30: Calculated gas-phase interaction energies $\Delta_{int}G°$ (at 1 bar pressure and 298.15 K) and solvation energies $\Delta_{solv}G°$ under standard conditions (1 bar gas to 1 mol L^{-1} solution) of the neutral water clusters $(MeOH)_n$, labeled by their solvent number *n* and the isomer number in brackets (*i*). The energies were calculated at the DSD-BLYP/def2-TZVPP level of theory and a subsequent DLPNO-CCSD(T)/CBS single-point calculation on the optimized DSD-BLYP/def2-TZVPP structure. Only the isomer labeled with (1) was used for the calculation of the proton solvation energies through the cluster cycle. All energies are given in kJ mol^{-1}.

	DSD-BLYP/def2-TZVPP		DLPNO-CCSD(T)/CBS	
n (*i*)	$\Delta_{int}G°$	$\Delta_{solv}G°$	$\Delta_{int}G°$	$\Delta_{solv}G°$
1		-10.82		-11.08
2	14.08	-22.95	16.91	-23.16
3 (1)	17.75	-14.83	25.45	-15.04
3 (2)	25.30	-28.26	31.69	-27.16
4 (1)	12.95	-16.19	25.67	-16.37
4 (2)	37.47	-48.06	47.79	-45.83
5 (1)	15.49	-20.81	31.74	-21.17
5 (2)	30.06	-31.96	45.86	-31.87
6 (1)	21.48	-23.39	41.33	-22.05
6 (2)	34.94	-28.55		

Table S31: Calculated DSD-BLYP/def2-TZVPP gas-phase energies in Hartree (1 H = 2625.4996 kJ mol^{-1}) of the protonated MeOH clusters at 1 bar pressure, labeled with their solvent number *n* and the isomer number in brackets (*i*). The energies listed are the enthalpy *H*, the Gibbs energy *G*, the entropy (already multiplied with the temperature $T{\cdot}S$) and the different contributions to the entropy (translational S_{trans}, vibrational S_{vib}, and rotational entropy S_{rot}) at 298.15 K, along with the total electronic energy E_{tot}. The rotational entropy was selected according to the symmetry number of the determined point group (pg).

n (*i*)	pg	E_{tot}	*H*	S_{trans}	S_{vib}	S_{rot}	$T{\cdot}S$	G
1	C_s	-115.936511	-115.866932	0.017303	0.001213	0.009264	0.027780	-115.894712
2 (1)	C_1	-231.627354	-231.502405	0.018263	0.008822	0.012103	0.039188	-231.541593
2 (2)	C_1	-231.627352	-231.502405	0.018263	0.008782	0.012103	0.039148	-231.541553
2 (3)	C_2	-231.627003	-231.502091	0.018263	0.008863	0.011486	0.038611	-231.540702
3 (1)	C_1	-347.301847	-347.116364	0.018830	0.019031	0.013733	0.051594	-347.167958
3 (2)	C_1	-347.301906	-347.116431	0.018830	0.018244	0.013735	0.050809	-347.167239
4 (1)	C_1	-462.964736	-462.722183	0.019234	0.028974	0.014628	0.062836	-462.785019
4 (2)	C_1	-462.964811	-462.722683	0.019234	0.028266	0.014679	0.062179	-462.784862
5 (1)	C_1	-578.625019	-578.323046	0.019548	0.037905	0.015340	0.072793	-578.395839
5 (2)	C_1	-578.624770	-578.322963	0.019548	0.037950	0.015375	0.072872	-578.395835
6 (1)	C_1	-694.288114	-693.927586	0.019804	0.045167	0.015590	0.080561	-694.008147
6 (2)	C_1	-694.285895	-693.925184	0.019804	0.045309	0.015544	0.080657	-694.005840
6 (3)	C_1	-694.281589	-693.921483	0.019804	0.047042	0.015717	0.082563	-694.004046

Table S32: Calculated DSD-BLYP/def2-TZVPP gas-phase energies in Hartree (1 H = 2625.4996 kJ mol^{-1}) of the neutral MeOH clusters at 1 bar pressure, labeled with their solvent number *n* and the isomer number in brackets (*i*). The energies listed are the enthalpy *H*, the Gibbs energy *G*, the entropy (already multiplied with the temperature $T{\cdot}S$) and the different contributions to the entropy (translational S_{trans}, vibrational S_{vib}, and rotational entropy S_{rot}) at 298.15 K, along with the total electronic energy E_{tot}. The rotational entropy was selected according to the symmetry number of the determined point group (pg).

n (*i*)	pg	E_{tot}	*H*	S_{trans}	S_{vib}	S_{rot}	$T{\cdot}S$	*G*
1	C_s	-115.636775	-115.580552	0.017259	0.000804	0.009015	0.027078	-115.607630
2	C_1	-231.283631	-231.168626	0.018241	0.010829	0.012200	0.041270	-231.209896
3 (1)	C_1	-346.940310	-346.766055	0.018815	0.017623	0.013637	0.050075	-346.816130
3 (2)	C_1	-346.935195	-346.761331	0.018815	0.019439	0.013668	0.051923	-346.813253
4 (1)	S_4	-462.599324	-462.366313	0.019223	0.026306	0.013746	0.059275	-462.425589
4 (2)	C_1	-462.587184	-462.354579	0.019223	0.028044	0.014401	0.061668	-462.416247
5 (1)	C_1	-578.253118	-577.961547	0.019539	0.036093	0.015072	0.070704	-578.032251
5 (2)	C_1	-578.248870	-577.957173	0.019539	0.035078	0.014913	0.069529	-578.026703
6 (1)	C_1	-693.906265	-693.555998	0.019797	0.046151	0.015653	0.081600	-693.637598
6 (2)	C_1	-693.904299	-693.553955	0.019797	0.043359	0.015364	0.078520	-693.632475

Table S33: Calculated DLPNO-CCSD(T)/CBS electronic gas-phase energy E_{tot}. in Hartree (1 H = 2625.4996 kJ mol^{-1}) of the protonated MeOH clusters under a pressure of 1 bar, labeled with their solvent number *n* and the isomer number in brackets (*i*). The Gibbs energy *G* was obtained with the thermal and entropic corrections from the respective DSD-BLYP/def2-TZVPP optimization at 298.15 K and 1 bar pressure.

n (*i*)	pg	E_{tot}	*G*
1	C_s	-115.909243	-115.867443
2 (1)	C_1	-231.572164	-231.486403
2 (2)	C_1	-231.572163	-231.486363
2 (3)	C_2	-231.571871	-231.485570
3 (1)	C_1	-347.220368	-347.086479
3 (2)	C_1	-347.220366	-347.085699
4 (1)	C_1	-462.856268	-462.676551
4 (2)	C_1	-462.856282	-462.676333
5 (1)	C_1	-578.489904	-578.260724
5 (2)	C_1	-578.489669	-578.260734
6 (1)	C_1	-694.125433	-693.845467
6 (2)	C_1	-694.123253	-693.843198
6 (3)	C_1	-694.119550	-693.842006

Table S34: Calculated DLPNO-CCSD(T)/CBS electronic gas-phase energy E_{tot}. in Hartree (1 H = 2625.4996 kJ mol^{-1}) of the neutral MeOH clusters under a pressure of 1 bar, labeled with their solvent number *n* and the isomer number in brackets (*i*). The Gibbs energy *G* was obtained with the thermal and entropic corrections from the respective DSD-BLYP/def2-TZVPP optimization at 298.15 K and 1 bar pressure.

n (*i*)	pg	E_{tot}	*G*
1	C_s	-115.611477	-115.582332
2	C_1	-231.231960	-231.158225
3 (1)	C_1	-346.861482	-346.737303
3 (2)	C_1	-346.856868	-346.734927
4 (1)	S_4	-462.493287	-462.319552
4 (2)	C_1	-462.482066	-462.311129
5 (1)	C_1	-578.120441	-577.899574
5 (2)	C_1	-578.116365	-577.894197
6 (1)	C_1	-693.746920	-693.478253
6 (2)	C_1		

Table S35: Total electronic energies E_{tot} calculated with the CPCM of the protonated MeOH clusters, labeled with their solvent number *n* and the isomer number in brackets (*i*). The energies were obtained from the DSD-BLYP/def2-TZVPP optimization and the DLPNO-CCSD(T)/CBS single-point calculation on the optimized structure in Hartree (1 H = 2625.4996 kJ mol^{-1}). Only the isomers labeled with (1) were used for further calculations of the standard solvation energies of the proton through the thermodynamic cycles. The solvation energies of the clusters were obtained through $\Delta_{solv}G° = E_{tot}(\text{CPCM}) - E_{tot}(\text{gas-phase})+7.96$.

n (*i*)	DSD-BLYP/def2-TZVPP	DLPNO-CCSD(T)/CBS
	E_{tot}	E_{tot}
1	-116.054560	-116.027179
2	-231.723493	-231.668104
2	-231.723433	-231.668027
3	-347.385575	-347.301997
4	-463.041836	-462.930945
5 (1)	-578.697278	-578.561618
5 (2)	-578.696922	-578.561375
6 (1)	-694.358611	-694.195345
6 (2)	-694.352888	

Table S36: Total electronic energies E_{tot} calculated with the CPCM of the neutral MeOH clusters, labeled with their solvent number *n* and the isomer number in brackets (*i*). The energies were obtained from the DSD-BLYP/def2-TZVPP optimization and the DLPNO-CCSD(T)/CBS single-point calculation on the optimized structure in Hartree (1 H = 2625.4996 kJ mol^{-1}). Only the isomers labeled with (1) were used for further calculations of the standard solvation energies of the proton through the thermodynamic cycles. The solvation energies of the clusters were obtained through $\Delta_{solv}G° = E_{tot}(CPCM) - E_{tot}(gas\text{-}phase)+7.96$.

n (*i*)	DSD-BLYP/def2-TZVPP E_{tot}	DLPNO-CCSD(T)/CBS E_{tot}
1	-115.643927	-115.618729
2	-231.295403	-231.243814
3 (1)	-346.948990	-346.870243
3 (2)	-346.948526	
4 (1)	-462.608520	-462.502553
4 (2)	-462.603043	
5 (1)	-578.264076	-578.131535
5 (2)	-578.261590	
6 (1)	-693.918206	-693.758794
6 (2)	-693.917712	-693.758349

Table S37: Calculated gas-phase clustering energies $\Delta_gG°$ (under a pressure of 1 bar) according to the monomer cycle as well as the solvation energies $\Delta_{solv}G°$ under standard conditions (1 bar gas to 1 mol L^{-1} solution) of MeOH and the protonated MeOH clusters, labeled by their solvent number *n* and the isomer number in brackets (*i*). The energies were calculated at the BP/def2-TZVPP level of theory, followed by a DLPNO-CCSD(T)/CBS single-point calculation on the optimized BP/def2-TZVPP structure. All energies are given in kJ mol^{-1}.

	BP/def2-TZVPP		DLPNO-CCSD(T)/CBS
n (*i*)	$\Delta_gG°$	$\Delta_{solv}G°$	$\Delta_gG°$
MeOH		-9.79	
1	-725.77	-301.39	-722.76
2 (1)	-834.97	-244.39	-815.71
2 (2)	-834.97	-244.39	-815.71
2 (3)	-832.54	-245.14	-813.24
3 (1)	-885.63	-213.14	-860.79
3 (2)	-885.36	-214.11	-861.89
4 (1)	-914.09	-195.62	-879.74
4 (2)	-913.45	-195.71	-879.04
5 (1)	-925.82	-183.48	-885.01
5 (2)	-925.70	-183.00	-883.51
6 (1)	-945.89	-177.34	-889.62
6 (2)	-935.57	-186.17	-883.42
6 (3)	-930.84	-198.89	-881.00

Table S38: Calculated BP/def2-TZVPP gas-phase energies in Hartree (1 H = 2625.4996 kJ mol^{-1}) of MeOH and the protonated MeOH clusters under 1 bar pressure, labeled with their solvent number *n* and the isomer number in brackets (*i*). The energies listed are the enthalpy *H*, the Gibbs energy *G*, the entropy (already multiplied with the temperature $T{\cdot}S$) and the different contributions to the entropy (translational S_{trans}, vibrational S_{vib}, and rotational entropy S_{rot}) at 298.15 K, along with the total electronic energy E_{tot}. The rotational entropy was selected according to the symmetry number of the determined point group (pg).

n (*i*)	pg	E_{tot}	*H*	S_{trans}	S_{vib}	S_{rot}	$T{\cdot}S$	*G*
MeOH		-115.778969	-115.725029	0.017259	0.000855	0.009040	0.027154	-115.752183
1	C_s	-116.077809	-116.010760	0.017303	0.001276	0.009292	0.027871	-116.038631
2 (1)	C_1	-231.914389	-231.793327	0.018263	0.008688	0.012130	0.039081	-231.832408
2 (2)	C_1	-231.914389	-231.793327	0.018263	0.008689	0.012130	0.039082	-231.832409
2 (3)	C_2	-231.914103	-231.793062	0.018263	0.008645	0.011510	0.038419	-231.831481
3 (1)	C_1	-347.731323	-347.553330	0.018830	0.018004	0.013720	0.050555	-347.603885
3 (2)	C_1	-347.730954	-347.552934	0.018830	0.018284	0.013737	0.050851	-347.603784
4 (1)	C_1	-463.538355	-463.305303	0.019234	0.027725	0.014649	0.061607	-463.366910
4 (2)	C_1	-463.538322	-463.305251	0.019234	0.027577	0.014605	0.061415	-463.366666
5 (1)	C_1	-579.341316	-579.051820	0.019548	0.036884	0.015307	0.071739	-579.123558
5 (2)	C_1	-579.341498	-579.051983	0.019548	0.036689	0.015294	0.071531	-579.123513
6 (1)	C_1	-695.149776	-694.804101	0.019804	0.043940	0.015542	0.079286	-694.883388
6 (2)	C_1	-695.146409	-694.800251	0.019804	0.043882	0.015518	0.079205	-694.879456
6 (3)	C_1	-695.141565	-694.796367	0.019804	0.045797	0.015687	0.081288	-694.877655

Table S39: Total electronic energies E_{tot} calculated with the CPCM of the protonated MeOH clusters, labeled with their solvent number *n* and the isomer number in brackets (*i*). The energies were obtained from the BP/def2-TZVPP optimization in Hartree (1 H = 2625.4996 kJ mol^{-1}). Only the isomers labeled with (1) were used for further calculations of the standard solvation energies of the proton through the thermodynamic cycles. The solvation energies of the clusters were obtained through $\Delta_{solv}G° = E_{tot}(\text{CPCM}) - E_{tot}(\text{gas-phase}) + 7.96$.

n (*i*)	E_{tot}
MeOH	-115.785731
1	-116.195635
2 (1)	-232.010503
2 (2)	-232.010453
2 (3)	-232.010399
3	-347.815536
4	-463.615897
5 (1)	-579.414231
5 (2)	-579.414222
6 (1)	-695.220351
6 (2)	-695.213441
6 (3)	-695.213441

3.3.3 Ethanol Clusters

Table S40: Calculated gas-phase clustering energies $\Delta_g G°$ (at 1 bar) according to the monomer and the cluster cycle as well as the solvation energies $\Delta_{solv}G°$ under standard conditions (1 bar gas to 1 mol L^{-1} solution) of the protonated $H(EtOH)_n^+$ clusters, labeled by their solvent number *n* and the isomer number in brackets (*i*). The energies were calculated at the DSD-BLYP/def2-TZVPP level of theory, followed by a DLPNO-CCSD(T)/CBS single-point calculation on the optimized DSD-BLYP/def2-TZVPP structure. All energies are given in kJ mol^{-1}.

	DSD-BLYP/def2-TZVPP			DLPNO-CCSD(T)/CBS		
n (*i*)	$\Delta_g G°$(monomer)	$\Delta_g G°$(cluster)	$\Delta_{solv}G°$	$\Delta_g G°$(monomer)	$\Delta_g G°$(cluster)	$\Delta_{solv}G°$
1 (1)	-750.73	-750.73	-281.81	-745.21	-745.21	-281.51
1 (2)	-749.85	-749.85	-283.00	-744.38	-744.38	-282.65
2 (1)	-846.32	-865.31	-226.86	-834.63	-856.65	-226.24
2 (2)	-845.22	-864.21	-227.57	-833.58	-855.59	-226.91
2 (3)	-844.96	-863.96	-226.23	-833.30	-855.31	-225.59
2 (4)	-844.96	-863.96	-227.04	-833.20	-855.21	-226.50
2 (5)	-844.96	-863.95	-227.62	-833.25	-855.26	-227.03
2 (6)	-842.11	-861.10	-228.92	-830.43	-852.45	-228.29
2 (7)	-841.68	-860.68	-230.66	-830.20	-852.22	-229.84
3 (1)	-888.58	-910.77	-199.38	-874.74	-904.70	-197.98
3 (2)	-888.02	-910.22	-197.98	-873.72	-903.68	-197.05
3 (3)	-887.76	-909.96	-199.18	-873.62	-903.58	-198.09
3 (4)	-887.12	-909.32	-198.05	-872.69	-902.65	-197.25
3 (5)	-886.67	-908.86	-198.55	-871.86	-901.82	-198.13
3 (6)	-886.45	-908.64	-199.41	-872.42	-902.38	-198.21
3 (7)	-885.41	-907.61	-198.91	-871.50	-901.46	-197.59
4 (1)	-907.58	-928.92	-191.24	-888.84	-922.93	-188.75
4 (2)	-903.59	-924.93	-183.91	-884.80	-918.90	-181.46
4 (3)	-903.04	-924.38	-183.74	-883.57	-917.66	-181.98
4 (4)	-903.03	-924.37	-184.98	-883.95	-918.04	-182.83
4 (5)	-902.15	-923.49	-184.70	-882.84	-916.93	-182.78
5 (1)	-917.21	-943.67	-176.83	-893.96	-936.26	-174.90
5 (2)	-916.05	-942.51	-176.53	-892.83	-935.13	-174.57
5 (3)	-916.06	-942.52	-175.37	-892.99	-935.29	-173.26
6 (1)	-922.36	-957.73	-167.36	-894.84	-949.16	-165.98
6 (2)	-917.44	-952.81	-172.69	-891.31	-945.64	-169.92

Table S41: Calculated gas-phase interaction energies $\Delta_{int}G°$ (at 1 bar) and solvation energies $\Delta_{solv}G°$ under standard conditions (1 bar gas to 1 mol L^{-1} solution) of the neutral water clusters $(EtOH)_n$, labeled by their solvent number n and the isomer number in brackets (i). The energies were calculated at the DSD-BLYP/def2-TZVPP level of theory and a subsequent DLPNO-CCSD(T)/CBS single-point calculation on the optimized DSD-BLYP/def2-TZVPP structure. Only the isomer labeled with (1) was used for the calculation of the proton solvation energies through the cluster cycle. All energies are given in kJ mol^{-1}.

	DSD-BLYP/def2-TZVPP		DLPNO-CCSD(T)/CBS	
n (i)	$\Delta_{int}G°$	$\Delta_{solv}G°$	$\Delta_{int}G°$	$\Delta_{solv}G°$
1		-10.34		-10.70
2 (1)	18.99	-21.54	22.01	-22.47
2 (2)	19.16	-23.86	22.43	-25.04
2 (3)	19.90	-21.62	23.03	-22.65
3 (1)	22.19	-17.61	29.96	-18.39
3 (2)	23.94	-16.33	31.41	-16.80
3 (3)	24.39	-17.58	32.18	-18.39
4 (1)	21.34	-17.54	34.09	-18.96
4 (2)	23.33	-20.13	35.40	-20.87
5 (1)	26.46	-20.05	42.30	-21.41
5 (2)	27.18	-17.13		
5 (3)	55.16	-47.01	69.87	-47.24
6 (1)	35.37	-25.47	54.32	-26.92
6 (2)	42.64	-27.54		

Table S42: Calculated DSD-BLYP/def2-TZVPP gas-phase energies in Hartree (1 H = 2625.4996 kJ mol^{-1}) of the protonated EtOH clusters at 1 bar pressure, labeled with their solvent number *n* and the isomer number in brackets (*i*). The energies listed are the enthalpy *H*, the Gibbs energy *G*, the entropy (already multiplied with the temperature *T*·*S*) and the different contributions to the entropy (translational S_{trans}, vibrational S_{vib}, and rotational entropy S_{rot}) at 298.15 K, along with the total electronic energy E_{tot}. The rotational entropy was selected according to the symmetry number of the determined point group (pg).

n (*i*)	pg	E_{tot}	*H*	S_{trans}	S_{vib}	S_{rot}	*T*·*S*	*G*
1 (1)	C_1	-155.221848	-155.122744	0.017804	0.002885	0.010742	0.031430	-155.154175
1 (2)	C_s	-155.221396	-155.122305	0.017804	0.002970	0.010760	0.031534	-155.153839
2 (1)	C_1	-310.188298	-310.003857	0.018771	0.012942	0.013229	0.044942	-310.048799
2 (2)	C_1	-310.188027	-310.003604	0.018771	0.012655	0.013349	0.044775	-310.048380
2 (3)	C_2	-310.188538	-310.004101	0.018771	0.012818	0.012593	0.044181	-310.048282
2 (4)	C_1	-310.188229	-310.003771	0.018771	0.012439	0.013302	0.044511	-310.048282
2 (5)	C_1	-310.188008	-310.003596	0.018771	0.012659	0.013255	0.044685	-310.048281
2 (6)	C_1	-310.187513	-310.002671	0.018771	0.012511	0.013243	0.044524	-310.047195
2 (7)	C_1	-310.186851	-310.002391	0.018771	0.012582	0.013289	0.044642	-310.047033
3 (1)	C_1	-465.138618	-464.863698	0.019340	0.025163	0.014912	0.059415	-464.923113
3 (2)	C_1	-465.139149	-464.864130	0.019340	0.024558	0.014873	0.058771	-464.922901
3 (3)	C_1	-465.138692	-464.863638	0.019340	0.024927	0.014897	0.059164	-464.922802
3 (4)	C_1	-465.139121	-464.864126	0.019340	0.024232	0.014860	0.058431	-464.922558
3 (5)	C_1	-465.138933	-464.863888	0.019340	0.024367	0.014790	0.058497	-464.922385
3 (6)	C_1	-465.138604	-464.863617	0.019340	0.024459	0.014885	0.058684	-464.922301
3 (7)	C_1	-465.138796	-464.863808	0.019340	0.023962	0.014796	0.058098	-464.921907
4 (1)	C_1	-620.078627	-619.716835	0.019745	0.036341	0.015649	0.071735	-619.788570
4 (2)	C_1	-620.081416	-619.717526	0.019745	0.034287	0.015490	0.069522	-619.787047
4 (3)	C_1	-620.081482	-619.717634	0.019745	0.034050	0.015410	0.069204	-619.786839
4 (4)	C_1	-620.081011	-619.717114	0.019745	0.034521	0.015457	0.069722	-619.786837
4 (5)	C_1	-620.081116	-619.717297	0.019745	0.034007	0.015452	0.069203	-619.786501
5 (1)	C_1	-775.019882	-774.568859	0.020059	0.045476	0.016059	0.081594	-774.650453
5 (2)	C_1	-775.019994	-774.568741	0.020059	0.045152	0.016059	0.081270	-774.650011
5 (3)	C_1	-775.020437	-774.569410	0.020059	0.044506	0.016042	0.080607	-774.650017
6 (1)	C_1	-929.957556	-929.417569	0.020316	0.056283	0.016466	0.093065	-929.510633
6 (2)	C_1	-929.955527	-929.415559	0.020316	0.056318	0.016566	0.093200	-929.508759

Table S43: Calculated DSD-BLYP/def2-TZVPP gas-phase energies in Hartree (1 H = 2625.4996 kJ mol^{-1}) of the neutral EtOH clusters at 1 bar pressure, labeled with their solvent number *n* and the isomer number in brackets (*i*). The energies listed are the enthalpy *H*, the Gibbs energy *G*, the entropy (already multiplied with the temperature $T{\cdot}S$) and the different contributions to the entropy (translational S_{trans}, vibrational S_{vib}, and rotational entropy S_{rot}) at 298.15 K, along with the total electronic energy E_{tot}. The rotational entropy was selected according to the symmetry number of the determined point group (pg).

n (*i*)	pg	E_{tot}	*H*	S_{trans}	S_{vib}	S_{rot}	$T{\cdot}S$	*G*
1	C_s	-154.913669	-154.827556	0.017774	0.002297	0.010592	0.030663	-154.858218
2 (1)	C_1	-309.838291	-309.663495	0.018755	0.013607	0.013344	0.045706	-309.709201
2 (2)	C_1	-309.837409	-309.662606	0.018755	0.014398	0.013382	0.046534	-309.709140
2 (3)	C_1	-309.838263	-309.663475	0.018755	0.013258	0.013369	0.045382	-309.708857
3 (1)	C_1	-464.771038	-464.507090	0.019330	0.025009	0.014772	0.059111	-464.566200
3 (2)	C_1	-464.771526	-464.507655	0.019330	0.023910	0.014640	0.057879	-464.565534
3 (3)	C_1	-464.771050	-464.507092	0.019330	0.024166	0.014776	0.058272	-464.565364
4 (1)	C_1	-619.708424	-619.355757	0.019737	0.033860	0.015390	0.068987	-619.424744
4 (2)	C_1	-619.707439	-619.354974	0.019737	0.033975	0.015301	0.069013	-619.423988
5 (1)	C_1	-774.639649	-774.198677	0.020053	0.046198	0.016083	0.082334	-774.281011
5 (2)	C_1	-774.640762	-774.199765	0.020053	0.044995	0.015926	0.080973	-774.280738
5 (3)	C_1	-774.629383	-774.188473	0.020053	0.045647	0.015909	0.081609	-774.270082
6 (1)	C_1	-929.571127	-929.041663	0.020311	0.057475	0.016386	0.094173	-929.135836
6 (2)	C_1	-929.570342	-929.040794	0.020311	0.055613	0.016350	0.092274	-929.133069

Table S44: Calculated DLPNO-CCSD(T)/CBS electronic gas-phase energy E_{tot}. in Hartree (1 H = 2625.4996 kJ mol^{-1}) of the protonated EtOH clusters under a pressure of 1 bar, labeled with their solvent number *n* and the isomer number in brackets (*i*). The Gibbs energy *G* was obtained with the thermal and entropic corrections from the respective DSD-BLYP/def2-TZVPP optimization at 298.15 K and 1 bar pressure.

n (*i*)	pg	E_{tot}	*G*
1 (1)	C_1	-155.179850	-155.112176
1 (2)	C_s	-155.179414	-155.111858
2 (1)	C_1	-310.104055	-309.964556
2 (2)	C_1	-310.103800	-309.964153
2 (3)	C_2	-310.104304	-309.964048
2 (4)	C_1	-310.103956	-309.964010
2 (5)	C_1	-310.103755	-309.964028
2 (6)	C_1	-310.103274	-309.962957
2 (7)	C_1	-310.102687	-309.962869
3 (1)	C_1	-465.013660	-464.798155
3 (2)	C_1	-465.014014	-464.797766
3 (3)	C_1	-465.013618	-464.797727
3 (4)	C_1	-465.013938	-464.797375
3 (5)	C_1	-465.013604	-464.797056
3 (6)	C_1	-465.013573	-464.797270
3 (7)	C_1	-465.013809	-464.796920
4 (1)	C_1	-619.911904	-619.621847
4 (2)	C_1	-619.914678	-619.620310
4 (3)	C_1	-619.914484	-619.619841
4 (4)	C_1	-619.914158	-619.619984
4 (5)	C_1	-619.914178	-619.619562
5 (1)	C_1	-774.811547	-774.442117
5 (2)	C_1	-774.811671	-774.441688
5 (3)	C_1	-774.812170	-774.441750
6 (1)	C_1	-929.707698	-929.260775
6 (2)	C_1	-929.706200	-929.259433

Table S45: Calculated DLPNO-CCSD(T)/CBS electronic gas-phase energy E_{tot}. in Hartree (1 H = 2625.4996 kJ mol^{-1}) of the neutral EtOH clusters under a pressure of 1 bar, labeled with their solvent number *n* and the isomer number in brackets (*i*). The Gibbs energy *G* was obtained with the thermal and entropic corrections from the respective DSD-BLYP/def2-TZVPP optimization at 298.15 K and 1 bar pressure.

n (*i*)	pg	E_{tot}	*G*
1	C_s	-154.873774	-154.818322
2 (1)	C_1	-309.757350	-309.628260
2 (2)	C_1	-309.756370	-309.628101
2 (3)	C_1	-309.757278	-309.627872
3 (1)	C_1	-464.648392	-464.443555
3 (2)	C_1	-464.648997	-464.443005
3 (3)	C_1	-464.648394	-464.442709
4 (1)	C_1	-619.543983	-619.260304
4 (2)	C_1	-619.543256	-619.259805
5 (1)	C_1	-774.434137	-774.075499
5 (2)	C_1		
5 (3)	C_1	-774.424299	-774.064998
6 (1)	C_1	-929.324533	-928.889242
6 (2)	C_1		

Table S46: Total electronic energies E_{tot} calculated with the CPCM of the protonated EtOH clusters, labeled with their solvent number *n* and the isomer number in brackets (*i*). The energies were obtained from the DSD-BLYP/def2-TZVPP optimization and the DLPNO-CCSD(T)/CBS single-point calculation on the optimized structure in Hartree (1 H = 2625.4996 kJ mol^{-1}). Only the isomers labeled with (1) were used for further calculations of the standard solvation energies of the proton through the thermodynamic cycles. The solvation energies of the clusters were obtained through $\Delta_{solv}G°$ = E_{tot}(CPCM) – E_{tot}(gas-phase)+7.96.

n (*i*)	DSD-BLYP/def2-TZVPP E_{tot}	DLPNO-CCSD(T)/CBS E_{tot}
1	-155.332217	-155.290103
1	-155.332207	-155.290037
2 (1)	-310.277735	-310.193259
2 (2)	-310.277628	
3 (1)	-465.217588	-465.092098
3 (2)	-465.217432	
3 (3)	-465.217360	
3 (4)	-465.217261	
3 (5)	-465.216957	-465.091451
3 (6)	-465.216957	-465.091451
4 (1)	-620.154497	-619.986826
4 (2)	-620.154457	-619.985621
5 (1)	-775.090264	-774.881194
5 (2)	-775.089975	
6 (1)	-930.024332	-929.773950
6 (2)	-930.023050	

Table S47: Total electronic energies E_{tot} calculated with the CPCM of the neutral EtOH clusters, labeled with their solvent number *n* and the isomer number in brackets (*i*). The energies were obtained from the DSD-BLYP/def2-TZVPP optimization and the DLPNO-CCSD(T)/CBS single-point calculation on the optimized structure in Hartree (1 H = 2625.4996 kJ mol^{-1}). Only the isomers labeled with (1) were used for further calculations of the standard solvation energies of the proton through the thermodynamic cycles. The solvation energies of the clusters were obtained through $\Delta_{solv}G°$ = E_{tot}(CPCM) – E_{tot}(gas-phase)+7.96.

n (*i*)	DSD-BLYP/def2-TZVPP E_{tot}	DLPNO-CCSD(T)/CBS E_{tot}

1	-154.920641	-154.880882
2 (1)	-309.849529	-309.768938
2 (2)	-309.849254	-309.768389
2 (3)	-309.849254	-309.768389
3 (1)	-464.780778	-464.658429
3 (2)	-464.780449	
3 (3)	-464.780236	-464.657908
3 (4)	-464.780203	-464.657876
4 (1)	-619.718137	
4 (2)	-619.717358	
5 (1)	-774.650319	-619.554235
5 (2)	-774.643828	-619.553217
6 (1)	-929.583861	-774.445323
6 (2)	-929.583278	-774.439215

Table S48: Calculated gas-phase clustering energies $\Delta_g G°$ (under a pressure of 1 bar) according to the monomer cycle as well as the solvation energies $\Delta_{solv}G°$ under standard conditions (1 bar gas to 1 mol L^{-1} solution) of EtOH and the protonated EtOH clusters, labeled by their solvent number *n* and the isomer number in brackets (*i*). The energies were calculated at the BP/def2-TZVPP level of theory. All energies are given in kJ mol^{-1}.

n (*i*)	$\Delta_g G°$	$\Delta_{solv}G°$
EtOH		-9.55
1 (1)	-754.64	-279.92
1 (2)	-754.15	-280.44
2 (1)	-856.45	-225.65
2 (2)	-856.07	-226.10
2 (3)	-855.87	-226.41
2 (4)	-854.81	-226.68
2 (5)	-854.44	-226.85
2 (6)	-852.26	-228.62
2 (7)	-851.58	-229.58
3 (1)	-901.77	-198.68
3 (2)	-901.61	-199.60
3 (3)	-900.41	-199.72
3 (4)	-901.29	-198.06
3 (5)	-900.65	-199.08
3 (6)	-900.35	-198.34
3 (7)	-899.96	-198.19
4 (1)	-924.90	-193.33
4 (2)	-922.31	-185.16
4 (3)	-922.15	-186.17
4 (4)	-921.84	-186.01
4 (5)	-921.33	-186.42
5 (1)	-940.95	-178.26
5 (2)	-940.44	-177.15

Table S49: Calculated BP/def2-TZVPP gas-phase energies in Hartree (1 H = 2625.4996 kJ mol^{-1}) of the protonated water EtOH under 1 bar pressure, labeled with their solvent number *n* and the isomer number in brackets (*i*). The energies listed are the enthalpy *H*, the Gibbs energy *G*, the entropy (already multiplied with the temperature *T·S*) and the different contributions to the entropy (translational S_{trans}, vibrational S_{vib}, and rotational entropy S_{rot}) at 298.15 K, along with the total electronic energy E_{tot}. The rotational entropy was selected according to the symmetry number of the determined point group (pg).

n (*i*)	pg	E_{tot}	*H*	S_{trans}	S_{vib}	S_{rot}	*T·S*	*G*
EtOH	C_1	-155.113820	-155.031059	0.017774	0.002465	0.010613	0.030852	-155.061911
1 (1)	C_1	-155.422962	-155.327651	0.017804	0.003130	0.010771	0.031705	-155.359357
1 (2)	C_s	-155.422763	-155.327416	0.017804	0.003159	0.010788	0.031752	-155.359168
2 (1)	C_1	-310.593264	-310.415004	0.018771	0.013005	0.013263	0.045039	-310.460043
2 (2)	C_1	-310.593091	-310.414827	0.018771	0.013050	0.013252	0.045073	-310.459900
2 (3)	C_1	-310.592975	-310.414687	0.018771	0.012998	0.013368	0.045137	-310.459824
2 (4)	C_1	-310.592872	-310.414572	0.018771	0.012751	0.013327	0.044849	-310.459421
2 (5)	C_1	-310.592806	-310.414504	0.018771	0.012723	0.013281	0.044775	-310.459279
2 (6)	C_1	-310.592134	-310.413827	0.018771	0.012583	0.013266	0.044620	-310.458447
2 (7)	C_1	-310.591766	-310.413558	0.018771	0.012565	0.013296	0.044631	-310.458189
3 (1)	C_1	-465.744210	-465.479999	0.019340	0.024992	0.014885	0.059217	-465.539216
3 (2)	C_1	-465.743860	-465.479785	0.019340	0.025115	0.014918	0.059372	-465.539157
3 (3)	C_1	-465.743816	-465.479506	0.019340	0.024949	0.014905	0.059193	-465.538699
3 (4)	C_1	-465.744447	-465.480221	0.019340	0.024620	0.014853	0.058813	-465.539034
3 (5)	C_1	-465.744059	-465.479842	0.019340	0.024730	0.014879	0.058949	-465.538791
3 (6)	C_1	-465.744342	-465.480118	0.019340	0.024424	0.014792	0.058556	-465.538674
3 (7)	C_1	-465.744399	-465.480097	0.019340	0.024301	0.014790	0.058430	-465.538527
4 (1)	C_1	-620.886231	-620.538141	0.019745	0.036402	0.015650	0.071796	-620.609938
4 (2)	C_1	-620.889343	-620.539754	0.019745	0.034038	0.015416	0.069198	-620.608952
4 (3)	C_1	-620.888960	-620.539388	0.019745	0.034270	0.015489	0.069503	-620.608891
4 (4)	C_1	-620.889021	-620.539473	0.019745	0.034111	0.015441	0.069297	-620.608771
4 (5)	C_1	-620.888863	-620.539299	0.019745	0.034100	0.015434	0.069279	-620.608577
5 (1)	C_1	-776.029661	-775.596232	0.020059	0.045653	0.016017	0.081729	-775.677961
5 (2)	C_1	-776.030084	-775.596660	0.020059	0.044995	0.016053	0.081108	-775.677768

Table S50: Total electronic energies E_{tot} calculated with the CPCM of the protonated EtOH clusters, labeled with their solvent number n and the isomer number in brackets (i). The energies were obtained from the BP/def2-TZVPP optimization in Hartree (1 H = 2625.4996 kJ mol^{-1}). Only the isomers labeled with (1) were used for further calculations of the standard solvation energies of the proton through the thermodynamic cycles. The solvation energies of the clusters were obtained through $\Delta_{solv}G° = E_{tot}(CPCM) - E_{tot}(gas\text{-}phase)+7.96$.

n (i)	E_{tot}
EtOH	-155.120490
1 (1)	-155.532608
1 (2)	-155.532397
2 (1)	-310.682241
2 (2)	-310.682140
2 (3)	-310.682001
2 (4)	-310.681820
2 (5)	-310.682001
2 (6)	-310.681696
3 (1)	-465.822916
3 (2)	-465.822703
3 (3)	-465.822674
3 (4)	-465.822520
3 (5)	-465.822440
4 (1)	-620.962899
4 (2)	-620.962658
4 (3)	-620.962640
4 (4)	-620.962311
5 (1)	-776.100589
5 (2)	-776.100244

3.3.4 Acetonitrile Clusters

Table S51: Calculated gas-phase clustering energies $\Delta_g G°$ (at 1 bar) according to the monomer and the cluster cycle as well as the solvation energies $\Delta_{solv}G°$ under standard conditions (1 bar gas to 1 mol L^{-1} solution) of the protonated $H(MeCN)_n^+$ clusters, labeled by their solvent number *n* and the isomer number in brackets (*i*). The energies were calculated at the DSD-BLYP/def2-TZVPP level of theory, followed by a DLPNO-CCSD(T)/CBS single-point calculation on the optimized DSD-BLYP/def2-TZVPP structure. All energies are given in kJ mol^{-1}.

	DSD-BLYP/def2-TZVPP			DLPNO-CCSD(T)/CBS		
n (*i*)	$\Delta_g G°$(monomer)	$\Delta_g G°$(cluster)	$\Delta_{solv}G°$	$\Delta_g G°$(monomer)	$\Delta_g G°$(cluster)	$\Delta_{solv}G°$
1	-751.88	-751.88	-262.35	-748.70	-748.70	-262.00
2	-856.77	-869.06	-195.33	-848.30	-860.34	-195.97
3 (1)	-863.53	-893.18	-179.28	-855.90	-885.27	-178.69
3 (2)	-861.65	-891.31	-182.82	-852.72	-882.09	-183.53
4 (1)	-863.77	-905.13	-183.68	-853.38	-896.02	-185.03
4 (2)	-860.03	-901.39	-180.36	-851.53	-894.17	-179.81

Table S52: Calculated gas-phase interaction energies $\Delta_{int}G°$ (at 1 bar) and solvation energies $\Delta_{solv}G°$ under standard conditions (1 bar gas to 1 mol L^{-1} solution) of the neutral water clusters $(MeCN)_n$, labeled by their solvent number *n* and the isomer number in brackets (*i*). The energies were calculated at the DSD-BLYP/def2-TZVPP level of theory and a subsequent DLPNO-CCSD(T)/CBS single-point calculation on the optimized DSD-BLYP/def2-TZVPP structure. Only the isomer labeled with (1) was used for the calculation of the proton solvation energies through the cluster cycle. All energies are given in kJ mol^{-1}.

	DSD-BLYP/def2-TZVPP		DLPNO-CCSD(T)/CBS	
n (*i*)	$\Delta_{int}G°$	$\Delta_{solv}G°$	$\Delta_{int}G°$	$\Delta_{solv}G°$
1		-19.32		-19.03
2 (1)	12.29	-26.82	12.04	-26.26
2 (2)	16.96	-40.44	16.93	-40.10
3 (1)	29.66	-42.41	29.37	-41.09
3 (2)	29.96	-40.93	30.90	-40.83
4 (1)	41.36	-38.75	42.64	-38.23
4 (2)	42.44	-38.84	42.51	-37.12
4 (3)	51.69	-61.49	53.79	-61.79

Table S53: Calculated DSD-BLYP/def2-TZVPP gas-phase energies in Hartree (1 H = 2625.4996 kJ mol^{-1}) of the protonated MeCN clusters at 1 bar pressure, labeled with their solvent number *n* and the isomer number in brackets (*i*). The energies listed are the enthalpy *H*, the Gibbs energy *G*, the entropy (already multiplied with the temperature *T·S*) and the different contributions to the entropy (translational S_{trans}, vibrational S_{vib}, and rotational entropy S_{rot}) at 298.15 K, along with the total electronic energy E_{tot}. The rotational entropy was selected according to the symmetry number of the determined point group (pg).

n (*i*)	pg	E_{tot}	*H*	S_{trans}	S_{vib}	S_{rot}	*T·S*	*G*
1	C_{3v}	-132.946702	-132.885365	0.017645	0.001544	0.008869	0.028058	-132.913423
2	C_3	-265.638152	-265.528065	0.018609	0.012267	0.011466	0.042343	-265.570408
3 (1)	C_1	-398.297594	-398.132915	0.019178	0.022904	0.015015	0.057097	-398.190012
3 (2)	C_s	-398.296249	-398.131982	0.019178	0.022975	0.015164	0.057317	-398.189299
4 (1)	C_1	-530.950464	-530.736046	0.019582	0.035433	0.016076	0.071092	-530.807138
4 (2)	C_1	-530.951728	-530.737521	0.019582	0.032721	0.015889	0.068192	-530.805713

Table S54: Calculated DSD-BLYP/def2-TZVPP gas-phase energies in Hartree (1 H = 2625.4996 kJ mol^{-1}) of the neutral MeCN clusters at 1 bar pressure, labeled with their solvent number *n* and the isomer number in brackets (*i*). The energies listed are the enthalpy *H*, the Gibbs energy *G*, the entropy (already multiplied with the temperature *T·S*) and the different contributions to the entropy (translational S_{trans}, vibrational S_{vib}, and rotational entropy S_{rot}) at 298.15 K, along with the total electronic energy E_{tot}. The rotational entropy was selected according to the symmetry number of the determined point group (pg).

n (*i*)	pg	E_{tot}	*H*	S_{trans}	S_{vib}	S_{rot}	*T·S*	*G*
1	C_{3v}	-132.639624	-132.589475	0.017610	0.001151	0.008796	0.027556	-132.617032
2 (1)	C_{2h}	-265.289123	-265.186817	0.018592	0.011582	0.012392	0.042566	-265.229383
2 (2)	C_1	-265.283936	-265.181742	0.018592	0.014522	0.012749	0.045863	-265.227606
3 (1)	C_1	-397.937256	-397.782825	0.019166	0.023333	0.014475	0.056974	-397.839799
3 (2)	C_{3h}	-397.937823	-397.783284	0.019166	0.023577	0.013656	0.056399	-397.839683
4 (1)	S_4	-530.592973	-530.386275	0.019574	0.032295	0.014232	0.066101	-530.452376
4 (2)	D_{2d}	-530.592937	-530.386305	0.019574	0.032501	0.013585	0.065660	-530.451965
4 (3)	C_2	-530.584311	-530.377597	0.019574	0.036056	0.015213	0.070843	-530.448441

Table S55: Calculated DLPNO-CCSD(T)/CBS electronic gas-phase energy E_{tot}. in Hartree (1 H = 2625.4996 kJ mol^{-1}) of the protonated MeCN clusters under a pressure of 1 bar, labeled with their solvent number *n* and the isomer number in brackets (*i*). The Gibbs energy *G* was obtained with the thermal and entropic corrections from the respective DSD-BLYP/def2-TZVPP optimization at 298.15 K and 1 bar pressure.

n (*i*)	pg	E_{tot}	*G*
1	C_{3v}	-132.892369	-132.859091
2	C_3	-265.528683	-265.460938
3 (1)	C_1	-398.135323	-398.027741
3 (2)	C_s	-398.133480	-398.026530
4 (1)	C_1	-530.734017	-530.590690
4 (2)	C_1	-530.736003	-530.589989

Table S56: Calculated DLPNO-CCSD(T)/CBS electronic gas-phase energy E_{tot}. in Hartree (1 H = 2625.4996 kJ mol^{-1}) of the neutral MeCN clusters under a pressure of 1 bar, labeled with their solvent number *n* and the isomer number in brackets (*i*). The Gibbs energy *G* was obtained with the thermal and entropic corrections from the respective DSD-BLYP/def2-TZVPP optimization at 298.15 K and 1 bar pressure.

n (*i*)	pg	E_{tot}	*G*
1	C_{3v}	-132.586503	-132.563910
2 (1)	C_{2h}	-265.182974	-265.123233
2 (2)	C_1	-265.177701	-265.121370
3 (1)	C_1	-397.777999	-397.680542
3 (2)	C_{3h}	-397.778099	-397.679959
4 (1)	S_4	-530.379996	-530.239399
4 (2)	D_{2d}	-530.380420	-530.239448
4 (3)	C_2	-530.371023	-530.235152

Table S57: Total electronic energies E_{tot} calculated with the CPCM of the protonated MeCN clusters, labeled with their solvent number *n* and the isomer number in brackets (*i*). The energies were obtained from the DSD-BLYP/def2-TZVPP optimization and the DLPNO-CCSD(T)/CBS single-point calculation on the optimized structure in Hartree (1 H = 2625.4996 kJ mol^{-1}). Only the isomers labeled with (1) were used for further calculations of the standard solvation energies of the proton through the thermodynamic cycles. The solvation energies of the clusters were obtained through $\Delta_{solv}G° = E_{tot}(CPCM) – E_{tot}(gas\text{-}phase)+7.96$.

n (*i*)	DSD-BLYP/def2-TZVPP	DLPNO-CCSD(T)/CBS
	E_{tot}	E_{tot}
1	-133.049656	-132.995192
2	-265.715581	-265.606355
3 (1)	-398.368911	-398.206415
3 (2)	-398.368334	-398.204905
4	-531.023456	-530.807523

Table S58: Total electronic energies E_{tot} calculated with the CPCM of the neutral MeCN clusters, labeled with their solvent number *n* and the isomer number in brackets (*i*). The energies were obtained from the DSD-BLYP/def2-TZVPP optimization and the DLPNO-CCSD(T)/CBS single-point calculation on the optimized structure in Hartree (1 H = 2625.4996 kJ mol^{-1}). Only the isomers labeled with (1) were used for further calculations of the standard solvation energies of the proton through the thermodynamic cycles. The solvation energies of the clusters were obtained through $\Delta_{solv}G° = E_{tot}(CPCM) – E_{tot}(gas\text{-}phase)+7.96$.

n (*i*)	DSD-BLYP/def2-TZVPP	DLPNO-CCSD(T)/CBS
	E_{tot}	E_{tot}
1	-132.650014	-132.596783
2	-265.302369	-265.196007
3	-397.956443	-397.796680
4	-530.610762	-530.397590

Table S59: Calculated gas-phase clustering energies $\Delta_g G°$ (under a pressure of 1 bar) according to the monomer cycle as well as the solvation energies $\Delta_{solv}G°$ under standard conditions (1 bar gas to 1 mol L^{-1} solution) of MeCN and the protonated MeCN clusters, labeled by their solvent number *n* and the isomer number in brackets (*i*). The energies were calculated at the BP/def2-TZVPP level of theory. All energies are given in kJ mol^{-1}.

n (*i*)	$\Delta_g G°$	$\Delta_{solv}G°$
MeCN		-19.97
1	-764.89	-260.84
2	-871.61	-192.57
3 (1)	-884.91	-179.24
3 (2)	-884.09	-180.96
4 (1)	-885.71	-173.21
4 (2)	-885.44	-177.51

Table S60: Calculated BP/def2-TZVPP gas-phase energies in Hartree (1 H = 2625.4996 kJ mol^{-1}) of MeCN and the protonated MeCN clusters under 1 bar pressure, labeled with their solvent number *n* and the isomer number in brackets (*i*). The energies listed are the enthalpy *H*, the Gibbs energy *G*, the entropy (already multiplied with the temperature $T{\cdot}S$) and the different contributions to the entropy (translational S_{trans}, vibrational S_{vib}, and rotational entropy S_{rot}) at 298.15 K, along with the total electronic energy E_{tot}. The rotational entropy was selected according to the symmetry number of the determined point group (pg).

n (*i*)	pg	E_{tot}	*H*	S_{trans}	S_{vib}	S_{rot}	$T{\cdot}S$	G
MeCN	C_{3v}	-132.811218	-132.762713	0.017610	0.001196	0.008807	0.027614	-132.790327
1	C_{3v}	-133.122557	-133.063410	0.017645	0.001743	0.008877	0.028265	-133.091675
2	C_3	-265.989321	-265.883362	0.018609	0.009200	0.011479	0.039288	-265.922650
3 (1)	C_1	-398.819216	-398.660892	0.019178	0.022813	0.015158	0.057148	-398.718040
3 (2)	C_s	-398.818561	-398.659770	0.019178	0.023761	0.015020	0.057959	-398.717728
4 (1)	C_1	-531.646111	-531.438625	0.019582	0.034406	0.016059	0.070048	-531.508673
4 (2)	C_1	-531.644475	-531.436506	0.019582	0.036587	0.015896	0.072064	-531.508570

Table S61: Total electronic energies E_{tot} calculated with the CPCM of the protonated MeCN clusters, labeled with their solvent number *n* and the isomer number in brackets (*i*). The energies were obtained from the BP/def2-TZVPP optimization in Hartree (1 H = 2625.4996 kJ mol^{-1}). Only the isomers labeled with (1) were used for further calculations of the standard solvation energies of the proton through the thermodynamic cycles. The solvation energies of the clusters were obtained through $\Delta_{solv}G° = E_{tot}(\text{CPCM}) - E_{tot}(\text{gas-phase})+7.96$.

n (*i*)	E_{tot}
MeCN	-132.821856
1	-133.224936
2 (1)	-266.065701
3 (1)	-398.890517
3 (2)[a]	-398.889477
4 (1)	-531.715117
4 (2)[a]	-531.712702

[a] structures with imaginary modes

3.3.5 DMF Clusters

Table S62: Calculated gas-phase clustering energies $\Delta_g G°$ (at 1 bar) according to the monomer and the cluster cycle as well as the solvation energies $\Delta_{solv}G°$ under standard conditions (1 bar gas to 1 mol L^{-1} solution) of the protonated $H(DMF)_n^+$ clusters, labeled by their solvent number *n* and the isomer number in brackets (*i*). The energies were calculated at the DSD-BLYP/def2-TZVPP level of theory, followed by a DLPNO-CCSD(T)/CBS single-point calculation on the optimized DSD-BLYP/def2-TZVPP structure. All energies are given in kJ mol^{-1}.

	DSD-BLYP/def2-TZVPP			DLPNO-CCSD(T)/CBS		
n (*i*)	$\Delta_g G°$(monomer)	$\Delta_g G°$(cluster)	$\Delta_{solv}G°$	$\Delta_g G°$(monomer)	$\Delta_g G°$(cluster)	$\Delta_{solv}G°$
1 (1)	-854.57	-854.57	-240.41	-855.68	-855.68	-239.67
1 (2)	-839.38	-839.38	-255.67	-840.31	-840.31	-255.11
2 (1)	-958.44	-979.04	-183.70	-954.81	-976.00	-183.25
2 (2)	-941.01	-961.61	-194.12	-938.49	-959.68	-192.56
3 (1)	-961.94	-1004.04	-176.21	-958.72	-1000.98	-162.93
3 (2)	-945.85	-987.94	-190.35	-943.33	-985.59	-176.37
4 (1)	-956.80	-1011.07	-177.60	-951.59	-1007.88	-176.94
4 (2)	-954.83	-1009.10	-180.69	-950.06	-1006.35	-179.60
4 (3)	-946.40	-1000.66	-183.33	-942.13	-998.42	-181.74
4 (4)	-944.27	-998.54	-186.51			

Table S63: Calculated gas-phase interaction energies $\Delta_{int}G°$ (at 1 bar) and solvation energies $\Delta_{solv}G°$ under standard conditions (1 bar gas to 1 mol L^{-1} solution) of the neutral water clusters $(DMF)_n$, labeled by their solvent number *n* and the isomer number in brackets (*i*). The energies were calculated at the DSD-BLYP/def2-TZVPP level of theory and a subsequent DLPNO-CCSD(T)/CBS single-point calculation on the optimized DSD-BLYP/def2-TZVPP structure. Only the isomer labeled with (1) was used for the calculation of the proton solvation energies through the cluster cycle. All energies are given in kJ mol^{-1}.

	DSD-BLYP/def2-TZVPP		DLPNO-CCSD(T)/CBS	
n (*i*)	$\Delta_{int}G°$	$\Delta_{solv}G°$	$\Delta_{int}G°$	$\Delta_{solv}G°$
1		-22.95		-24.23
2 (1)	20.60	-36.70	21.19	-37.70
2 (2)	26.91	-46.50	26.28	-46.28
3 (1)	42.10	-53.49	42.26	-52.71
3 (2)	43.00	-52.33	43.56	-51.95
3 (3)	43.81	-51.60	46.47	-53.32
3 (4)	44.43	-53.50	45.99	-54.12
4 (1)	54.27	-49.10	56.29	-50.15
4 (2)	54.69	-47.51	56.49	-48.33

Table S64: Calculated DSD-BLYP/def2-TZVPP gas-phase energies in Hartree (1 H = 2625.4996 kJ mol^{-1}) of the protonated DMF clusters at 1 bar pressure, labeled with their solvent number *n* and the isomer number in brackets (*i*). The energies listed are the enthalpy *H*, the Gibbs energy *G*, the entropy (already multiplied with the temperature $T{\cdot}S$) and the different contributions to the entropy (translational S_{trans}, vibrational S_{vib}, and rotational entropy S_{rot}) at 298.15 K, along with the total electronic energy E_{tot}. The rotational entropy was selected according to the symmetry number of the determined point group (pg).

n (*i*)	pg	E_{tot}	*H*	S_{trans}	S_{vib}	S_{rot}	$T{\cdot}S$	*G*
1 (1)	C_s	-248.655999	-248.531532	0.018447	0.005800	0.012140	0.036387	-248.567919
1 (2)	C_s	-248.650186	-248.525794	0.018447	0.005743	0.012150	0.036340	-248.562134
2 (1)	C_1	-497.018386	-496.784255	0.019419	0.021520	0.014696	0.055635	-496.839890
2 (2)	C_1	-497.014416	-496.778400	0.019419	0.020824	0.014609	0.054852	-496.833252
3 (1)	C_1	-745.348992	-744.999583	0.019990	0.037797	0.016268	0.074055	-745.073637
3 (2)	C_1	-745.343603	-744.994142	0.019990	0.037094	0.016281	0.073365	-745.067507
4 (1)	C_1	-993.676507	-993.214266	0.020396	0.052909	0.016519	0.089824	-993.304090
4 (2)	C_1	-993.675329	-993.213288	0.020396	0.052942	0.016715	0.090053	-993.303341
4 (3)	C_1	-993.674323	-993.211650	0.020396	0.051452	0.016630	0.088478	-993.300128
4 (4)	C_1	-993.673113	-993.210988	0.020396	0.051310	0.016626	0.088331	-993.299319

Table S65: Calculated DSD-BLYP/def2-TZVPP gas-phase energies in Hartree (1 H = 2625.4996 kJ mol^{-1}) of the neutral DMF clusters at 1 bar pressure, labeled with their solvent number *n* and the isomer number in brackets (*i*). The energies listed are the enthalpy *H*, the Gibbs energy *G*, the entropy (already multiplied with the temperature $T{\cdot}S$) and the different contributions to the entropy (translational S_{trans}, vibrational S_{vib}, and rotational entropy S_{rot}) at 298.15 K, along with the total electronic energy E_{tot}. The rotational entropy was selected according to the symmetry number of the determined point group (pg).

n (*i*)	pg	E_{tot}	*H*	S_{trans}	S_{vib}	S_{rot}	$T{\cdot}S$	*G*
1	C_s	-248.306662	-248.196203	0.018427	0.005683	0.012098	0.036208	-248.232412
2 (1)	C_i	-496.626162	-496.402848	0.019409	0.020482	0.014238	0.054129	-496.456978
2 (2)	C_{2h}	-496.622427	-496.399059	0.019409	0.022052	0.014055	0.055516	-496.454574
3 (1)	C_1	-744.944829	-744.608480	0.019983	0.036947	0.015791	0.072722	-744.681202
3 (2)	C_1	-744.945268	-744.609057	0.019983	0.036319	0.015499	0.071801	-744.680858
3 (3)	C_1	-744.945548	-744.609513	0.019983	0.035527	0.015528	0.071038	-744.680550
3 (4)	C_1	-744.944824	-744.608533	0.019983	0.036100	0.015698	0.071780	-744.680314
4 (1)	C_1	-993.272055	-992.822460	0.020391	0.049923	0.016204	0.086518	-992.908978
4 (2)	C_1	-993.272661	-992.823075	0.020391	0.049155	0.016197	0.085743	-992.908817

Table S66: Calculated DLPNO-CCSD(T)/CBS electronic gas-phase energy E_{tot}. in Hartree (1 H = 2625.4996 kJ mol^{-1}) of the protonated DMF clusters under a pressure of 1 bar, labeled with their solvent number *n* and the isomer number in brackets (*i*). The Gibbs energy *G* was obtained with the thermal and entropic corrections from the respective DSD-BLYP/def2-TZVPP optimization at 298.15 K and 1 bar pressure.

n (*i*)	pg	E_{tot}	*G*
1 (1)	C_s	-248.576454	-248.488373
1 (2)	C_s	-248.570572	-248.482520
2 (1)	C_1	-496.857069	-496.678573
2 (2)	C_1	-496.853521	-496.672357
3 (1)	C_1	-745.107862	-744.832507
3 (2)	C_1	-745.102741	-744.826645
4 (1)	C_1	-993.354653	-992.982237
4 (2)	C_1	-993.353641	-992.981653
4 (3)	C_1	-993.352828	-992.978632
4 (4)	C_1		

Table S67: Calculated DLPNO-CCSD(T)/CBS electronic gas-phase energy E_{tot}. in Hartree (1 H = 2625.4996 kJ mol^{-1}) of the neutral DMF clusters under a pressure of 1 bar, labeled with their solvent number *n* and the isomer number in brackets (*i*). The Gibbs energy *G* was obtained with the thermal and entropic corrections from the respective DSD-BLYP/def2-TZVPP optimization at 298.15 K and 1 bar pressure.

n (*i*)	pg	E_{tot}	*G*
1	C_s	-248.226695	-248.152444
2 (1)	C_i	-496.466000	-496.296816
2 (2)	C_{2h}	-496.462733	-496.294880
3 (1)	C_1	-744.704862	-744.441236
3 (2)	C_1	-744.705155	-744.440744
3 (3)	C_1	-744.704630	-744.439632
3 (4)	C_1	-744.704327	-744.439816
4 (1)	C_1	-992.951414	-992.588337
4 (2)	C_1	-992.952106	-992.588262

Table S68: Total electronic energies E_{tot} calculated with the CPCM of the protonated DMF clusters, labeled with their solvent number n and the isomer number in brackets (i). The energies were obtained from the DSD-BLYP/def2-TZVPP optimization and the DLPNO-CCSD(T)/CBS single-point calculation on the optimized structure in Hartree (1 H = 2625.4996 kJ mol^{-1}). Only the isomers labeled with (1) were used for further calculations of the standard solvation energies of the proton through the thermodynamic cycles. The solvation energies of the clusters were obtained through $\Delta_{solv}G° = E_{tot}(\text{CPCM}) – E_{tot}(\text{gas-phase})+7.96$.

n (i)	DSD-BLYP/def2-TZVPP	DLPNO-CCSD(T)/CBS
	E_{tot}	E_{tot}
1 (1)	-248.750598	-248.670769
1 (2)	-248.746962	-248.667051
2 (1)	-497.091384	-496.929896
2 (2)	-497.088389	-496.927602
3 (1)	-745.419138	-745.172949
3 (2)	-745.416751	
3 (3)	-745.416751	
4 (1)	-993.747182	-993.425079
4 (2)	-993.746961	

Table S69: Total electronic energies E_{tot} calculated with the CPCM of the neutral DMF clusters, labeled with their solvent number n and the isomer number in brackets (i). The energies were obtained from the DSD-BLYP/def2-TZVPP optimization and the DLPNO-CCSD(T)/CBS single-point calculation on the optimized structure in Hartree (1 H = 2625.4996 kJ mol^{-1}). Only the isomers labeled with (1) were used for further calculations of the standard solvation energies of the proton through the thermodynamic cycles. The solvation energies of the clusters were obtained through $\Delta_{solv}G° = E_{tot}(\text{CPCM}) – E_{tot}(\text{gas-phase})+7.96$.

n (i)	DSD-BLYP/def2-TZVPP	DLPNO-CCSD(T)/CBS
	E_{tot}	E_{tot}
1	-248.318434	-248.238957
2	-496.643171	-496.483392
3 (1)	-744.968233	-744.727971
3 (2)	-744.967202	-744.727509
3 (3)	-744.966667	
3 (4)	-744.965814	-744.725909
3 (5)	-744.961625	
4 (1)	-993.293789	-992.973545
4 (2)	-993.293028	
4 (3)	-993.291599	
4 (4)	-993.291211	

Table S70: Calculated gas-phase clustering energies $\Delta_g G°$ (under a pressure of 1 bar) according to the monomer cycle as well as the solvation energies $\Delta_{solv}G°$ under standard conditions (1 bar gas to 1 mol L^{-1} solution) of DMF and the protonated DMF clusters, labeled by their solvent number *n* and the isomer number in brackets (*i*). The energies were calculated at the BP/def2-TZVPP level of theory. All energies are given in kJ mol^{-1}.

n (*i*)	$\Delta_g G°$	$\Delta_{solv}G°$
DMF		-21.48
1 (1)	-852.36	-238.35
1 (2)	-839.08	-252.21
2 (1)	-962.14	-180.52
2 (2)	-945.05	-193.41
3 (1)	-965.44	-175.79
3 (2)	-952.28	-190.40
4 (1)	-958.78	-179.42
4 (2)	-958.60	-177.76
4 (3)	-949.11	-183.90
4 (4)	-948.30	-184.62

Table S71: Calculated BP/def2-TZVPP gas-phase energies in Hartree (1 H = 2625.4996 kJ mol^{-1}) of DMF and the protonated DMF clusters under 1 bar pressure, labeled with their solvent number *n* and the isomer number in brackets (*i*). The energies listed are the enthalpy *H*, the Gibbs energy *G*, the entropy (already multiplied with the temperature $T{\cdot}S$) and the different contributions to the entropy (translational S_{trans}, vibrational S_{vib}, and rotational entropy S_{rot}) at 298.15 K, along with the total electronic energy E_{tot}. The rotational entropy was selected according to the symmetry number of the determined point group (pg).

n (*i*)	pg	E_{tot}	*H*	S_{trans}	S_{vib}	S_{rot}	$T{\cdot}S$	G
DMF	C_s	-248.631115	-248.524957	0.018427	0.005637	0.012114	0.036179	-248.561136
1 (1)	C_1	-248.979057	-248.859220	0.018447	0.005977	0.012155	0.036579	-248.895799
1 (2)	C_1	-248.973780	-248.854103	0.018447	0.006026	0.012166	0.036639	-248.890743
2 (1)	C_1	-497.668973	-497.443539	0.019419	0.021079	0.014714	0.055211	-497.498750
2 (2)	C_1	-497.664061	-497.437586	0.019419	0.020614	0.014621	0.054654	-497.492239
3 (1)	C_1	-746.322326	-745.986652	0.019990	0.038183	0.016315	0.074488	-746.061140
3 (2)	C_1	-746.316761	-745.981130	0.019990	0.038716	0.016294	0.074999	-746.056129
4 (1)	C_1	-994.973222	-994.529349	0.020396	0.053319	0.016677	0.090392	-994.619741
4 (2)	C_1	-994.973855	-994.529719	0.020396	0.053030	0.016527	0.089952	-994.619671
4 (3)	C_1	-994.971515	-994.526943	0.020396	0.052082	0.016638	0.089115	-994.616058
4 (4)	C_1	-994.971242	-994.527245	0.020396	0.051498	0.016611	0.088505	-994.615750

Table S72: Total electronic energies E_{tot} calculated with the CPCM of the protonated DMF clusters, labeled with their solvent number *n* and the isomer number in brackets (*i*). The energies were obtained from the BP/def2-TZVPP optimization in Hartree (1 H = 2625.4996 kJ mol^{-1}). Only the isomers labeled with (1) were used for further calculations of the standard solvation energies of the proton through the thermodynamic cycles. The solvation energies of the clusters were obtained through $\Delta_{solv}G° = E_{tot}(CPCM) - E_{tot}(gas\text{-}phase)+7.96$.

n (*i*)	E_{tot}
DMF	-248.642329
1 (1)	-249.072871
1 (2)	-249.069761
2 (1)	-497.740760
2 (2)	-497.736996
3 (1)	-746.392312
3 (2)	-746.386634
4 (1)	-995.044590
4 (2)	-995.044560
4 (3)	-995.038278
4 (4)[a]	-995.037954

[a] structures with imaginary modes

3.3.6 DMSO Clusters

Table S73: Calculated gas-phase clustering energies $\Delta_g G°$ (at 1 bar) according to the monomer and the cluster cycle as well as the solvation energies $\Delta_{solv}G°$ under standard conditions (1 bar gas to 1 mol L^{-1} solution) of the protonated $H(DMSO)_n^+$ clusters, labeled by their solvent number *n* and the isomer number in brackets (*i*). The energies were calculated at the DSD-BLYP/def2-TZVPP level of theory, followed by a DLPNO-CCSD(T)/CBS single-point calculation on the optimized DSD-BLYP/def2-TZVPP structure. All energies are given in kJ mol^{-1}.

	DSD-BLYP/def2-TZVPP			DLPNO-CCSD(T)/CBS		
n (*i*)	$\Delta_g G°$(monomer)	$\Delta_g G°$(cluster)	$\Delta_{solv}G°$	$\Delta_g G°$(monomer)	$\Delta_g G°$(cluster)	$\Delta_{solv}G°$
1	-861.43	-861.43	-248.21	-854.32	-854.32	-247.24
2 (1)	-964.11	-977.95	-200.03	-951.05	-967.81	-198.94
2 (2)	-956.42	-970.27	-201.11	-943.42	-960.19	-199.96
3 (1)	-981.77	-1015.65	-182.30	-967.48	-1007.31	-180.98
3 (2)	-980.07	-1013.95	-184.58	-965.63	-1005.46	-183.40
3 (3)	-979.65	-1013.52	-179.47	-964.87	-1004.69	-178.64
4	-986.15	-1033.05	-170.09	-965.45	-1022.16	-171.98

Table S74: Calculated gas-phase interaction energies $\Delta_{int}G°$ (at 1 bar) and solvation energies $\Delta_{solv}G°$ under standard conditions (1 bar gas to 1 mol L^{-1} solution) of the neutral water clusters $(DMSO)_n$, labeled by their solvent number *n* and the isomer number in brackets (*i*). The energies were calculated at the DSD-BLYP/def2-TZVPP level of theory and a subsequent DLPNO-CCSD(T)/CBS single-point calculation on the optimized DSD-BLYP/def2-TZVPP structure. Only the isomer labeled with (1) was used for the calculation of the proton solvation energies through the cluster cycle. All energies are given in kJ mol^{-1}.

	DSD-BLYP/def2-TZVPP		DLPNO-CCSD(T)/CBS	
n (*i*)	$\Delta_{int}G°$	$\Delta_{solv}G°$	$\Delta_{int}G°$	$\Delta_{solv}G°$
1		-30.10		-31.44
2 (1)	13.84	-45.41	16.77	-47.77
2 (2)	16.73	-45.05	19.06	-46.82
2 (3)	19.46	-50.35	21.91	-52.24
3 (1)	33.87	-65.06	39.83	-68.84
3 (2)	38.01	-66.53	43.68	-70.02
4 (1)	46.89	-75.39	56.71	-78.60
4 (2)	49.64	-78.18	16.77	

Table S75: Calculated DSD-BLYP/def2-TZVPP gas-phase energies in Hartree (1 H = 2625.4996 kJ mol^{-1}) of the protonated DMSO clusters at 1 bar pressure, labeled with their solvent number *n* and the isomer number in brackets (*i*). The energies listed are the enthalpy *H*, the Gibbs energy *G*, the entropy (already multiplied with the temperature $T{\cdot}S$) and the different contributions to the entropy (translational S_{trans}, vibrational S_{vib}, and rotational entropy S_{rot}) at 298.15 K, along with the total electronic energy E_{tot}. The rotational entropy was selected according to the symmetry number of the determined point group (pg).

n (*i*)	pg	E_{tot}	*H*	S_{trans}	S_{vib}	S_{rot}	$T{\cdot}S$	*G*
1	C_s	-553.225495	-553.126224	0.018540	0.005395	0.011876	0.035811	-553.162035
2 (1)	C_1	-1106.157436	-1105.972439	0.019512	0.018617	0.014495	0.052624	-1106.025063
2 (2)	C_1	-1106.157026	-1105.970008	0.019512	0.018192	0.014423	0.052128	-1106.022136
3 (1)	C_1	-1659.062743	-1658.786303	0.020084	0.033311	0.016011	0.069406	-1658.855709
3 (2)	C_1	-1659.061877	-1658.785485	0.020084	0.033452	0.016041	0.069576	-1658.855061
3 (3)	C_1	-1659.063822	-1658.786656	0.020084	0.032387	0.015774	0.068244	-1658.854900
4	C_1	-2211.965152	-2211.599053	0.020490	0.045372	0.016380	0.082241	-2211.681294

Table S76: Calculated DSD-BLYP/def2-TZVPP gas-phase energies in Hartree (1 H = 2625.4996 kJ mol^{-1}) of the neutral DMSO clusters at 1 bar pressure, labeled with their solvent number *n* and the isomer number in brackets (*i*). The energies listed are the enthalpy *H*, the Gibbs energy *G*, the entropy (already multiplied with the temperature $T{\cdot}S$) and the different contributions to the entropy (translational S_{trans}, vibrational S_{vib}, and rotational entropy S_{rot}) at 298.15 K, along with the total electronic energy E_{tot}. The rotational entropy was selected according to the symmetry number of the determined point group (pg).

n (*i*)	pg	E_{tot}	*H*	S_{trans}	S_{vib}	S_{rot}	$T{\cdot}S$	*G*
1	C_1	-552.875981	-552.789237	0.018522	0.004346	0.011813	0.034681	-552.823918
2 (1)	C_i	-1105.765876	-1105.590242	0.019503	0.018571	0.014247	0.052322	-1105.642564
2 (2)	C_2	-1105.766011	-1105.590363	0.019503	0.018003	0.013592	0.051099	-1105.641463
2 (3)	C_1	-1105.763993	-1105.588289	0.019503	0.018317	0.014315	0.052136	-1105.640425
3 (1)	C_1	-1658.654902	-1658.390197	0.020078	0.032943	0.015634	0.068655	-1658.458852
3 (2)	C_1	-1658.654344	-1658.389668	0.020078	0.031896	0.015635	0.067608	-1658.457276
4 (1)	C_1	-2211.548058	-2211.194327	0.020485	0.046351	0.016648	0.083484	-2211.277811
4 (2)	C_1	-2211.546997	-2211.193256	0.020485	0.046354	0.016671	0.083509	-2211.276765

Table S77: Calculated DLPNO-CCSD(T)/CBS electronic gas-phase energy E_{tot}. in Hartree (1 H = 2625.4996 kJ mol^{-1}) of the protonated DMSO clusters under a pressure of 1 bar, labeled with their solvent number *n* and the isomer number in brackets (*i*). The Gibbs energy *G* was obtained with the thermal and entropic corrections from the respective DSD-BLYP/def2-TZVPP optimization at 298.15 K and 1 bar pressure.

n (*i*)	pg	E_{tot}	*G*
1	C_s	-552.968830	-552.905370
2 (1)	C_1	-1105.644548	-1105.512175
2 (2)	C_1	-1105.644160	-1105.509270
3 (1)	C_1	-1658.295429	-1658.088396
3 (2)	C_1	-1658.294508	-1658.087692
3 (3)	C_1	-1658.296322	-1658.087400
4	C_1	-2210.941439	-2210.657581

Table S78: Calculated DLPNO-CCSD(T)/CBS electronic gas-phase energy E_{tot}. in Hartree (1 H = 2625.4996 kJ mol^{-1}) of the neutral DMSO clusters under a pressure of 1 bar, labeled with their solvent number *n* and the isomer number in brackets (*i*). The Gibbs energy *G* was obtained with the thermal and entropic corrections from the respective DSD-BLYP/def2-TZVPP optimization at 298.15 K and 1 bar pressure.

n (*i*)	pg	E_{tot}	*G*
1	C_1	-552.622024	-552.569961
2 (1)	C_i	-1105.256849	-1105.133537
2 (2)	C_2	-1105.257211	-1105.132663
2 (3)	C_1	-1105.255146	-1105.131578
3 (1)	C_1	-1657.890765	-1657.694715
3 (2)	C_1	-1657.890315	-1657.693246
4 (1)	C_1	-2210.528492	-2210.258245
4 (2)	C_1		

Table S79: Total electronic energies E_{tot} calculated with the CPCM of the protonated DMSO clusters, labeled with their solvent number *n* and the isomer number in brackets (*i*). The energies were obtained from the DSD-BLYP/def2-TZVPP optimization and the DLPNO-CCSD(T)/CBS single-point calculation on the optimized structure in Hartree (1 H = 2625.4996 kJ mol^{-1}). Only the isomers labeled with (1) were used for further calculations of the standard solvation energies of the proton through the thermodynamic cycles. The solvation energies of the clusters were obtained through $\Delta_{solv}G° = E_{tot}(CPCM) - E_{tot}(gas\text{-}phase)+7.96$.

n (*i*)	DSD-BLYP/def2-TZVPP	DLPNO-CCSD(T)/CBS
	E_{tot}	E_{tot}
1	-553.323064	-553.066030
2 (1)	-1106.236657	-1105.723351
2 (2)	-1106.236460	
3 (1)	-1659.135210	-1658.367392
3 (2)	-1659.134905	-1658.367193
4	-2212.032969	-2211.009975

Table S80: Total electronic energies E_{tot} calculated with the CPCM of the neutral DMSO clusters, labeled with their solvent number *n* and the isomer number in brackets (*i*). The energies were obtained from the DSD-BLYP/def2-TZVPP optimization and the DLPNO-CCSD(T)/CBS single-point calculation on the optimized structure in Hartree (1 H = 2625.4996 kJ mol^{-1}). Only the isomers labeled with (1) were used for further calculations of the standard solvation energies of the proton through the thermodynamic cycles. The solvation energies of the clusters were obtained through $\Delta_{solv}G° = E_{tot}(CPCM) - E_{tot}(gas\text{-}phase)+7.96$.

n (*i*)	DSD-BLYP/def2-TZVPP	DLPNO-CCSD(T)/CBS
	E_{tot}	E_{tot}
1	-552.890479	-552.637030
2	-1105.786201	-1105.278075
3 (1)	-1658.682716	-1657.920015
3 (2)	-1658.681932	-1657.918790
4 (1)[a]	-2211.579806	-2210.561459
4 (2)[a]	-2211.578557	

[a] These structures contained imaginary modes

Table S81: Calculated gas-phase clustering energies $\Delta_g G°$ (under a pressure of 1 bar) according to the monomer cycle as well as the solvation energies $\Delta_{solv}G°$ under standard conditions (1 bar gas to 1 mol L^{-1} solution) of DMSO and the protonated DMSO clusters, labeled by their solvent number *n* and the isomer number in brackets (*i*). The energies were calculated at the BP/def2-TZVPP level of theory. All energies are given in kJ mol^{-1}.

n (*i*)	$\Delta_g G°$	$\Delta_{solv}G°$
DMSO		-27.17
1	-858.99	-245.69
2 (1)	-967.49	-196.61
2 (2)	-959.63	-198.49
3 (1)	-982.34	-180.47
3 (2)	-980.34	-186.73
4	-989.17	-179.16

Table S82: Calculated BP/def2-TZVPP gas-phase energies in Hartree (1 H = 2625.4996 kJ mol^{-1}) of the protonated water DMSO under 1 bar pressure, labeled with their solvent number *n* and the isomer number in brackets (*i*). The energies listed are the enthalpy *H*, the Gibbs energy *G*, the entropy (already multiplied with the temperature *T·S*) and the different contributions to the entropy (translational S_{trans}, vibrational S_{vib}, and rotational entropy S_{rot}) at 298.15 K, along with the total electronic energy E_{tot}. The rotational entropy was selected according to the symmetry number of the determined point group (pg).

n (*i*)	pg	E_{tot}	*H*	S_{trans}	S_{vib}	S_{rot}	*T·S*	*G*
	C_s	-553.351992	-553.268545	0.018522	0.004790	0.011849	0.035161	-553.303705
1	C_s	-553.700308	-553.604683	0.018540	0.005765	0.011905	0.036210	-553.640893
2 (1)	C_1	-1107.110212	-1106.932154	0.019512	0.019739	0.014519	0.053770	-1106.985924
2 (2)	C_1	-1107.109495	-1106.929939	0.019512	0.019031	0.014451	0.052995	-1106.982934
3 (1)	C_1	-1660.491135	-1660.224548	0.020084	0.034896	0.015760	0.070740	-1660.295289
3 (2)	C_1	-1660.488753	-1660.222836	0.020084	0.035612	0.015995	0.071692	-1660.294527
4	C_1	-2213.870548	-2213.518066	0.020490	0.046679	0.016360	0.083528	-2213.601593

Table S83: Total electronic energies E_{tot} calculated with the CPCM of the protonated DMSO clusters, labeled with their solvent number *n* and the isomer number in brackets (*i*). The energies were obtained from the BP/def2-TZVPP optimization in Hartree (1 H = 2625.4996 kJ mol^{-1}). Only the isomers labeled with (1) were used for further calculations of the standard solvation energies of the proton through the thermodynamic cycles. The solvation energies of the clusters were obtained through $\Delta_{solv}G° = E_{tot}(\text{CPCM}) - E_{tot}(\text{gas-phase})+7.96$.

n (*i*)	E_{tot}
DMSO	-553.365371
1	-553.796920
2 (1)	-1107.188127
2 (2)[a]	-1107.188830
3 (1)	-1660.562905
3 (2)	-1660.565381
4	-2213.941819

[a] structures with imaginary modes

3.3.7 Propylene Carbonate Clusters

Table S84: Calculated gas-phase clustering energies $\Delta_g G°$ (at 1 bar) according to the monomer and the cluster cycle as well as the solvation energies $\Delta_{solv}G°$ under standard conditions (1 bar gas to 1 mol L^{-1} solution) of the protonated $H(PC)_n^+$ clusters, labeled by their solvent number *n* and the isomer number in brackets (*i*). The energies were calculated at the DSD-BLYP/def2-TZVPP level of theory, followed by a DLPNO-CCSD(T)/CBS single-point calculation on the optimized DSD-BLYP/def2-TZVPP structure. All energies are given in kJ mol^{-1}.

	DSD-BLYP/def2-TZVPP			DLPNO-CCSD(T)/CBS		
n (*i*)	$\Delta_g G°$(monomer)	$\Delta_g G°$(cluster)	$\Delta_{solv}G°$	$\Delta_g G°$(monomer)	$\Delta_g G°$(cluster)	$\Delta_{solv}G°$
1 (1)	-800.91	-800.91	-241.61	-797.63	-797.63	-245.11
1 (2)	-800.79	-800.79	-241.52	-797.50	-797.50	-245.11
2 (1)	-891.39	-900.60	-192.94	-883.56	-893.89	-205.48
2 (2)	-890.51	-899.72	-193.60	-882.79	-893.12	-205.48
2 (3)	-889.24	-898.45	-193.66	-881.62	-891.95	-205.48
3 (1)	-898.31	-918.70	-194.50	-891.27	-913.78	-239.51
3 (2)	-896.25	-916.64	-198.58	-889.35	-911.85	-239.51
4	-908.63	-947.00	-179.10	-898.94	-942.21	-245.11

Table S85: Calculated gas-phase interaction energies $\Delta_{int}G°$ (at 1 bar) and solvation energies $\Delta_{solv}G°$ under standard conditions (1 bar gas to 1 mol L^{-1} solution) of the neutral water clusters $(PC)_n$, labeled by their solvent number *n* and the isomer number in brackets (*i*). The energies were calculated at the DSD-BLYP/def2-TZVPP level of theory and a subsequent DLPNO-CCSD(T)/CBS single-point calculation on the optimized DSD-BLYP/def2-TZVPP structure. Only the isomer labeled with (1) was used for the calculation of the proton solvation energies through the cluster cycle. All energies are given in kJ mol^{-1}.

	DSD-BLYP/def2-TZVPP		DLPNO-CCSD(T)/CBS	
n (*i*)	$\Delta_{int}G°$	$\Delta_{solv}G°$	$\Delta_{int}G°$	$\Delta_{solv}G°$
1		-32.76		-33.17
2 (1)	9.21	-42.96	10.33	-43.22
2 (2)	9.47	-45.34	10.30	-45.31
2 (3)	24.44	-65.17	24.69	-64.56
3 (1)	20.40	-59.12	22.50	-58.82
3 (2)	25.60	-64.58	27.57	-64.14
3 (3)	26.17	-64.43	28.57	-64.43
3 (4)	26.38	-64.58		
4 (1)	38.37	-74.99	43.27	
4 (2)	42.90	-84.70	10.33	

Table S86: Calculated DSD-BLYP/def2-TZVPP gas-phase energies in Hartree (1 H = 2625.4996 kJ mol^{-1}) of the protonated PC clusters at 1 bar pressure, labeled with their solvent number *n* and the isomer number in brackets (*i*). The energies listed are the enthalpy *H*, the Gibbs energy *G*, the entropy (already multiplied with the temperature *T·S*) and the different contributions to the entropy (translational S_{trans}, vibrational S_{vib}, and rotational entropy S_{rot}) at 298.15 K, along with the total electronic energy E_{tot}. The rotational entropy was selected according to the symmetry number of the determined point group (pg).

n (*i*)	pg	E_{tot}	*H*	S_{trans}	S_{vib}	S_{rot}	*T·S*	*G*
1 (1)	C_1	-381.775306	-381.651012	0.018914	0.006050	0.012873	0.037838	-381.688850
1 (2)	C_1	-381.775341	-381.651074	0.018914	0.005945	0.012871	0.037730	-381.688804
2 (1)	C_1	-763.275174	-763.040607	0.019889	0.021182	0.015418	0.056489	-763.097096
2 (2)	C_1	-763.274925	-763.040547	0.019889	0.020909	0.015419	0.056217	-763.096763
2 (3)	C_1	-763.274900	-763.040099	0.019889	0.020870	0.015420	0.056179	-763.096278
3 (1)	C_1	-1144.748536	-1144.398645	0.020461	0.037582	0.016827	0.074870	-1144.473515
3 (2)	C_1	-1144.746983	-1144.397473	0.020461	0.037873	0.016924	0.075257	-1144.472730
4	C_1	-1526.225835	-1525.762141	0.020867	0.051365	0.016856	0.089089	-1525.851229

Table S87: Calculated DSD-BLYP/def2-TZVPP gas-phase energies in Hartree (1 H = 2625.4996 kJ mol^{-1}) of the neutral PC clusters at 1 bar pressure, labeled with their solvent number *n* and the isomer number in brackets (*i*). The energies listed are the enthalpy *H*, the Gibbs energy *G*, the entropy (already multiplied with the temperature *T·S*) and the different contributions to the entropy (translational S_{trans}, vibrational S_{vib}, and rotational entropy S_{rot}) at 298.15 K, along with the total electronic energy E_{tot}. The rotational entropy was selected according to the symmetry number of the determined point group (pg).

n (*i*)	pg	E_{tot}	*H*	S_{trans}	S_{vib}	S_{rot}	*T·S*	*G*
1	C_1	-381.447535	-381.336479	0.018901	0.005569	0.012835	0.037305	-381.373784
2 (1)	C_1	-762.913189	-762.688626	0.019882	0.020599	0.014952	0.055433	-762.744059
2 (2)	C_1	-762.912279	-762.687826	0.019882	0.021225	0.015026	0.056134	-762.743960
2 (3)	C_1	-762.904727	-762.680431	0.019882	0.022563	0.015381	0.057826	-762.738258
3 (1)	C_1	-1144.377864	-1144.039821	0.020456	0.037048	0.016256	0.073760	-1144.113581
3 (2)	C_1	-1144.375784	-1144.037705	0.020456	0.037071	0.016366	0.073894	-1144.111599
3 (3)	C_1	-1144.375840	-1144.037726	0.020456	0.036909	0.016290	0.073656	-1144.111382
3 (4)	C_1	-1144.375784	-1144.037925	0.020456	0.036770	0.016151	0.073377	-1144.111302
4 (1)	C_1	-1525.843034	-1525.391461	0.020864	0.051273	0.016921	0.089058	-1525.480519
4 (2)	C_1	-1525.839335	-1525.388027	0.020864	0.052844	0.017061	0.090768	-1525.478796

Table S88: Calculated DLPNO-CCSD(T)/CBS electronic gas-phase energy E_{tot}. in Hartree (1 H = 2625.4996 kJ mol^{-1}) of the protonated PC clusters under a pressure of 1 bar, labeled with their solvent number *n* and the isomer number in brackets (*i*). The Gibbs energy *G* was obtained with the thermal and entropic corrections from the respective DSD-BLYP/def2-TZVPP optimization at 298.15 K and 1 bar pressure.

n (*i*)	pg	E_{tot}	*G*
1 (1)	C_1	-381.657933	-381.571476
1 (2)	C_1	-381.657965	-381.571428
2 (1)	C_1	-763.039938	-762.861861
2 (2)	C_1	-763.039732	-762.861570
2 (3)	C_1	-763.039743	-762.861122
3 (1)	C_1	-1144.397479	-1144.122457
3 (2)	C_1	-1144.395976	-1144.121723
4	C_1	-1525.757640	-1525.383035

Table S89: Calculated DLPNO-CCSD(T)/CBS electronic gas-phase energy E_{tot}. in Hartree (1 H = 2625.4996 kJ mol^{-1}) of the neutral PC clusters under a pressure of 1 bar, labeled with their solvent number *n* and the isomer number in brackets (*i*). The Gibbs energy *G* was obtained with the thermal and entropic corrections from the respective DSD-BLYP/def2-TZVPP optimization at 298.15 K and 1 bar pressure.

n (*i*)	pg	E_{tot}	*G*
1	1	-381.331409	-381.257657
2 (1)	2 (1)	-762.680510	-762.511380
2 (2)	2 (2)	-762.679712	-762.511392
2 (3)	2 (3)	-762.672381	-762.505912
3 (1)	3 (1)	-1144.028682	-1143.764400
3 (2)	3 (2)	-1144.026657	-1143.762472
3 (3)	3 (3)	-1144.026547	-1143.762089
3 (4)	3 (4)		
4 (1)	4 (1)	-1525.376664	-1525.014149
4 (2)	4 (2)		

Table S90: Total electronic energies E_{tot} calculated with the CPCM of the protonated PC clusters, labeled with their solvent number n and the isomer number in brackets (i). The energies were obtained from the DSD-BLYP/def2-TZVPP optimization and the DLPNO-CCSD(T)/CBS single-point calculation on the optimized structure in Hartree (1 H = 2625.4996 kJ mol^{-1}). Only the isomers labeled with (1) were used for further calculations of the standard solvation energies of the proton through the thermodynamic cycles. The solvation energies of the clusters were obtained through $\Delta_{solv}G° = E_{tot}(CPCM) - E_{tot}(gas\text{-}phase)+7.96$.

n (i)	DSD-BLYP/def2-TZVPP E_{tot}	DLPNO-CCSD(T)/CBS E_{tot}
1	-381.870364	-381.752753
2 (1)	-763.351694	-763.116447
2 (2)	-763.351413	-763.115227
3	-1144.825649	-1144.473918
4	-1526.297081	

Table S91: Total electronic energies E_{tot} calculated with the CPCM of the neutral PC clusters, labeled with their solvent number n and the isomer number in brackets (i). The energies were obtained from the DSD-BLYP/def2-TZVPP optimization and the DLPNO-CCSD(T)/CBS single-point calculation on the optimized structure in Hartree (1 H = 2625.4996 kJ mol^{-1}). Only the isomers labeled with (1) were used for further calculations of the standard solvation energies of the proton through the thermodynamic cycles. The solvation energies of the clusters were obtained through $\Delta_{solv}G° = E_{tot}(CPCM) - E_{tot}(gas\text{-}phase)+7.96$.

n (i)	DSD-BLYP/def2-TZVPP E_{tot}	DLPNO-CCSD(T)/CBS E_{tot}
1	-381.463043	-381.347075
2 (1)	-762.932582	-762.700003
2 (2)	-762.932359	-762.699911
3 (1)	-1144.403413	-1144.054119
3 (2)	-1144.402939	
3 (3)	-1144.402836	-1144.053647
3 (4)	-1144.400669	
4	-1525.874627	

Table S92: Calculated gas-phase clustering energies $\Delta_g G°$ (under a pressure of 1 bar) according to the monomer cycle as well as the solvation energies $\Delta_{solv}G°$ under standard conditions (1 bar gas to 1 mol L^{-1} solution) of PC and the protonated PC clusters, labeled by their solvent number *n* and the isomer number in brackets (*i*). The energies were calculated at the BP/def2-TZVPP level of theory. All energies are given in kJ mol^{-1}.

n (*i*)	$\Delta_g G°$	$\Delta_{solv}G°$
PC		-30.47
1 (1)	-803.76	-238.67
1 (2)	-803.79	-238.69
2 (1)	-903.09	-189.62
2 (2)	-903.15	-189.64
2 (3)	-902.56	-190.14
3 (1)	-905.40	-185.12
3 (2)	-905.49	-187.78
4	-904.11	-175.62

Table S93: Calculated BP/def2-TZVPP gas-phase energies in Hartree (1 H = 2625.4996 kJ mol^{-1}) of PC and the protonated PC clusters under 1 bar pressure, labeled with their solvent number *n* and the isomer number in brackets (*i*). The energies listed are the enthalpy *H*, the Gibbs energy *G*, the entropy (already multiplied with the temperature $T{\cdot}S$) and the different contributions to the entropy (translational S_{trans}, vibrational S_{vib}, and rotational entropy S_{rot}) at 298.15 K, along with the total electronic energy E_{tot}. The rotational entropy was selected according to the symmetry number of the determined point group (pg).

n (*i*)	pg	E_{tot}	*H*	S_{trans}	S_{vib}	S_{rot}	$T{\cdot}S$	*G*
PC	C_1	-381.907141	-381.800445	0.018901	0.005942	0.012859	0.037702	-381.838147
1 (1)	C_1	-382.235630	-382.116147	0.018914	0.006342	0.012896	0.038152	-382.154300
1 (2)	C_1	-382.235621	-382.116115	0.018914	0.006384	0.012898	0.038196	-382.154311
2 (1)	C_1	-764.198051	-763.972955	0.019889	0.022005	0.015433	0.057327	-764.030282
2 (2)	C_1	-764.198043	-763.972942	0.019889	0.022016	0.015456	0.057361	-764.030303
2 (3)	C_1	-764.197851	-763.972725	0.019889	0.022022	0.015444	0.057355	-764.030080
3 (1)	C_1	-1146.127687	-1145.791772	0.020461	0.040234	0.016839	0.077534	-1145.869306
3 (2)	C_1	-1146.126676	-1145.791168	0.020461	0.040815	0.016897	0.078174	-1145.869342
4	C_1	-1528.061334	-1527.615982	0.020867	0.053235	0.016877	0.090980	-1527.706962

Table S94: Total electronic energies E_{tot} calculated with the CPCM of the protonated PC clusters, labeled with their solvent number n and the isomer number in brackets (i). The energies were obtained from the BP/def2-TZVPP optimization in Hartree (1 H = 2625.4996 kJ mol⁻¹). Only the isomers labeled with (1) were used for further calculations of the standard solvation energies of the proton through the thermodynamic cycles. The solvation energies of the clusters were obtained through $\Delta_{solv}G° = E_{tot}(CPCM) - E_{tot}(gas\text{-}phase)+7.96$.

n (i)	E_{tot}
PC	-381.921778
1 (1)	-382.329566
1 (2)	-382.329490
2 (1)	-764.273304
2 (1)	-764.272429
2 (2)	-764.272340
3 (1)[a]	-1146.201228
3 (2)[a]	-1146.197065
4[a]	-1528.131254

[a] structures with imaginary modes

3.3.8 Methyl Formate Clusters

Table S95: Calculated gas-phase clustering energies $\Delta_gG°$ (at 1 bar) according to the monomer and the cluster cycle as well as the solvation energies $\Delta_{solv}G°$ under standard conditions (1 bar gas to 1 mol L⁻¹ solution) of the protonated $H(MeFo)_n^+$ clusters, labeled by their solvent number n and the isomer number in brackets (i). The energies were calculated at the DSD-BLYP/def2-TZVPP level of theory, followed by a DLPNO-CCSD(T)/CBS single-point calculation on the optimized DSD-BLYP/def2-TZVPP structure. All energies are given in kJ mol⁻¹.

	DSD-BLYP/def2-TZVPP			DLPNO-CCSD(T)/CBS		
n (i)	$\Delta_gG°$(monomer)	$\Delta_gG°$(cluster)	$\Delta_{solv}G°$	$\Delta_gG°$(monomer)	$\Delta_gG°$(cluster)	$\Delta_{solv}G°$
1 (1)	-754.41	-754.41	-238.81	-750.55	-750.55	-238.82
1 (2)	-744.81	-744.81	-249.10	-740.47	-740.47	-249.60
2 (1)	-824.69	-853.07	-189.24	-816.89	-845.64	-189.85
2 (2)	-819.79	-848.17	-197.51	-809.01	-837.76	-201.10
2 (3)	-817.04	-845.42	-198.99	-807.77	-836.52	-201.06
2 (4)	-815.00	-843.38	-199.67	-806.33	-835.08	-201.16
2 (5)	-808.63	-837.01	-205.65	-800.86	-829.61	-206.23
3 (1)	-822.02	-882.26	-177.47	-813.65	-874.75	-177.67
3 (2)	-813.88	-874.11	-184.42	-804.67	-865.77	-185.45
3 (3)	-813.30	-873.53	-182.29	-804.60	-865.70	-182.82
4 (1)	-810.07	-908.44	-154.15	-798.26	-898.80	-155.99
4 (2)	-801.93	-900.30	-163.55	-792.02	-892.56	-163.48

Table S96: Calculated gas-phase interaction energies $\Delta_{int}G°$ (at 1 bar) and solvation energies $\Delta_{solv}G°$ under standard conditions (1 bar gas to 1 mol L^{-1} solution) of the neutral water clusters $(MeFo)_n$, labeled by their solvent number *n* and the isomer number in brackets (*i*). The energies were calculated at the DSD-BLYP/def2-TZVPP level of theory and a subsequent DLPNO-CCSD(T)/CBS single-point calculation on the optimized DSD-BLYP/def2-TZVPP structure. Only the isomer labeled with (1) was used for the calculation of the proton solvation energies through the cluster cycle. All energies are given in kJ mol^{-1}.

	DSD-BLYP/def2-TZVPP		DLPNO-CCSD(T)/CBS	
n (*i*)	$\Delta_{int}G°$	$\Delta_{solv}G°$	$\Delta_{int}G°$	$\Delta_{solv}G°$
1 (1)		-10.30		-10.56
1 (2)		-31.74		-33.10
2 (1)	28.4	-10.38	28.7	-10.56
2 (2)	42.0	-24.47	42.8	-25.06
2 (3)	42.6	-25.74	43.3	-26.29
3 (1)	60.2	-21.57	61.1	-20.61
3 (2)	71.6	-34.45	73.2	-34.26
3 (3)	74.8	-38.20	76.1	-37.72
4	98.4	-38.82	100.5	-38.56

Table S97: Calculated DSD-BLYP/def2-TZVPP gas-phase energies in Hartree (1 H = 2625.4996 kJ mol^{-1}) of the protonated MeFo clusters at 1 bar pressure, labeled with their solvent number *n* and the isomer number in brackets (*i*). The energies listed are the enthalpy *H*, the Gibbs energy *G*, the entropy (already multiplied with the temperature $T{\cdot}S$) and the different contributions to the entropy (translational S_{trans}, vibrational S_{vib}, and rotational entropy S_{rot}) at 298.15 K, along with the total electronic energy E_{tot}. The rotational entropy was selected according to the symmetry number of the determined point group (pg).

n (*i*)	pg	E_{tot}	*H*	S_{trans}	S_{vib}	S_{rot}	$T{\cdot}S$	*G*
1 (1)	C_1	-229.214495	-229.132988	0.018173	0.003242	0.011286	0.032701	-229.165689
1 (2)	C_s	-229.210574	-229.129195	0.018173	0.003545	0.011120	0.032837	-229.162032
2 (1)	C_s	-458.163843	-458.013279	0.019143	0.014417	0.013951	0.047511	-458.060790
2 (2)	C_1	-458.160693	-458.010970	0.019143	0.014903	0.013909	0.047955	-458.058925
2 (3)	C_s	-458.160129	-458.010488	0.019143	0.014361	0.013883	0.047387	-458.057875
2 (4)	C_1	-458.159869	-458.009548	0.019143	0.014479	0.013932	0.047553	-458.057101
2 (5)	C_1	-458.157591	-458.007579	0.019143	0.014105	0.013845	0.047093	-458.054673
3 (1)	C_1	-687.085747	-686.864208	0.019713	0.028787	0.015399	0.063898	-686.928107
3 (2)	C_1	-687.083099	-686.861939	0.019713	0.028144	0.015207	0.063064	-686.925003
3 (3)	C_1	-687.083910	-686.862308	0.019713	0.027576	0.015189	0.062477	-686.924785
4 (1)	C_1	-916.006430	-915.715772	0.020118	0.040148	0.015850	0.076116	-915.791888
4 (2)	C_1	-916.002848	-915.711020	0.020118	0.041478	0.016169	0.077765	-915.788785

Table S98: Calculated DSD-BLYP/def2-TZVPP gas-phase energies in Hartree (1 H = 2625.4996 kJ mol^{-1}) of the neutral MeFo clusters at 1 bar pressure, labeled with their solvent number *n* and the isomer number in brackets (*i*). The energies listed are the enthalpy *H*, the Gibbs energy *G*, the entropy (already multiplied with the temperature *T·S*) and the different contributions to the entropy (translational S_{trans}, vibrational S_{vib}, and rotational entropy S_{rot}) at 298.15 K, along with the total electronic energy E_{tot}. The rotational entropy was selected according to the symmetry number of the determined point group (pg).

n (*i*)	pg	E_{tot}	*H*	S_{trans}	S_{vib}	S_{rot}	*T·S*	*G*
1 (1)	C_s	-228.904390	-228.836175	0.018149	0.002802	0.011207	0.032158	-228.868332
1 (2)	C_s	-228.896224	-228.828424	0.018149	0.003666	0.011065	0.032881	-228.861304
2 (1)	C_1	-457.815622	-457.676971	0.019131	0.015757	0.013997	0.048885	-457.725855
2 (2)	C_1	-457.810255	-457.671988	0.019131	0.015727	0.013813	0.048671	-457.720659
2 (3)	C_1	-457.809770	-457.671487	0.019131	0.015922	0.013893	0.048946	-457.720432
3 (1)	C_1	-686.727461	-686.518524	0.019705	0.028760	0.015067	0.063532	-686.582056
3 (2)	C_1	-686.722552	-686.513997	0.019705	0.028893	0.015133	0.063731	-686.577728
3 (3)	C_1	-686.721126	-686.512566	0.019705	0.029082	0.015153	0.063940	-686.576506
4	C_1	-915.637672	-915.358695	0.020112	0.041289	0.015767	0.077168	-915.435863

Table S99: Calculated DLPNO-CCSD(T)/CBS electronic gas-phase energy E_{tot}. in Hartree (1 H = 2625.4996 kJ mol^{-1}) of the protonated MeFo clusters under a pressure of 1 bar, labeled with their solvent number *n* and the isomer number in brackets (*i*). The Gibbs energy *G* was obtained with the thermal and entropic corrections from the respective DSD-BLYP/def2-TZVPP optimization at 298.15 K and 1 bar pressure.

n (*i*)	pg	E_{tot}	*G*
1 (1)	C_1	-229.147553	-229.098746
1 (2)	C_s	-229.143449	-229.094907
2 (1)	C_s	-458.029926	-457.926874
2 (2)	C_1	-458.025641	-457.923873
2 (3)	C_s	-458.025656	-457.923402
2 (4)	C_1	-458.025619	-457.922850
2 (5)	C_1	-458.023688	-457.920769
3 (1)	C_1	-686.886140	-686.728500
3 (2)	C_1	-686.883174	-686.725078
3 (3)	C_1	-686.884178	-686.725053
4 (1)	C_1	-915.740038	-915.525496
4 (2)	C_1	-915.737185	-915.523122

Table S100: Calculated DLPNO-CCSD(T)/CBS electronic gas-phase energy E_{tot}. in Hartree (1 H = 2625.4996 kJ mol^{-1}) of the neutral MeFo clusters under a pressure of 1 bar, labeled with their solvent number *n* and the isomer number in brackets (*i*). The Gibbs energy *G* was obtained with the thermal and entropic corrections from the respective DSD-BLYP/def2-TZVPP optimization at 298.15 K and 1 bar pressure.

n (*i*)	pg	E_{tot}	*G*
1 (1)	C_s	-228.838917	-228.802860
1 (2)	C_s	-228.830331	-228.795411
2 (1)	C_1	-457.684536	-457.594769
2 (2)	C_1	-457.679012	-457.589415
2 (3)	C_1	-457.678545	-457.589208
3 (1)	C_1	-686.530713	-686.385308
3 (2)	C_1	-686.525515	-686.380690
3 (3)	C_1	-686.524199	-686.379579
4	C_1	-915.374954	-915.173145

Table S101: Total electronic energies E_{tot} calculated with the CPCM of the protonated MeFo clusters, labeled with their solvent number *n* and the isomer number in brackets (*i*). The energies were obtained from the DSD-BLYP/def2-TZVPP optimization and the DLPNO-CCSD(T)/CBS single-point calculation on the optimized structure in Hartree (1 H = 2625.4996 kJ mol^{-1}). Only the isomers labeled with (1) were used for further calculations of the standard solvation energies of the proton through the thermodynamic cycles. The solvation energies of the clusters were obtained through $\Delta_{solv}G°$ = E_{tot}(CPCM) – E_{tot}(gas-phase)+7.96.

n (*i*)	DSD-BLYP/def2-TZVPP	DLPNO-CCSD(T)/CBS
	E_{tot}	E_{tot}
1 (1)	-229.308484	-229.241548
1 (2)	-229.306502	-229.239302
2 (1)	-458.238951	-458.105267
2 (2)	-458.236101	-458.102124
2 (3)	-458.235760	-458.100927
3 (1)	-687.156374	-686.956842
3 (2)	-687.155538	-686.956062
3 (3)	-687.153122	-686.953439
4	-916.068172	-915.802485

Table S102: Total electronic energies E_{tot} calculated with the CPCM of the neutral MeFo clusters, labeled with their solvent number n and the isomer number in brackets (i). The energies were obtained from the DSD-BLYP/def2-TZVPP optimization and the DLPNO-CCSD(T)/CBS single-point calculation on the optimized structure in Hartree (1 H = 2625.4996 kJ mol^{-1}). Only the isomers labeled with (1) were used for further calculations of the standard solvation energies of the proton through the thermodynamic cycles. The solvation energies of the clusters were obtained through $\Delta_{solv}G° = E_{tot}(\text{CPCM}) - E_{tot}(\text{gas-phase})+7.96$.

n (i)	DSD-BLYP/def2-TZVPP E_{tot}	DLPNO-CCSD(T)/CBS E_{tot}
1 (1)	-228.911346	-228.845971
1 (2)	-228.906968	-228.840940
2 (1)	-457.822606	-457.691589
2 (2)	-457.822439	-457.690849
3 (1)	-686.738707	-686.541597
3 (2)	-686.737501	
4	-915.655489	-915.392672

Table S103: Calculated gas-phase clustering energies $\Delta_g G°$ (under a pressure of 1 bar) according to the monomer cycle as well as the solvation energies $\Delta_{solv}G°$ under standard conditions (1 bar gas to 1 mol L^{-1} solution) of MeFo and the protonated MeFo clusters, labeled by their solvent number n and the isomer number in brackets (i). The energies were calculated at the BP/def2-TZVPP level of theory. All energies are given in kJ mol^{-1}.

n (i)	$\Delta_g G°$	$\Delta_{solv}G°$
MeFo		-9.47
1 (1)	-756.84	-236.92
1 (2)	-748.41	-245.68
2 (1)	-836.02	-185.71
2 (2)	-835.52	-189.43
2 (3)	-833.90	-192.92
2 (4)	-833.72	-193.32
2 (5)	-828.12	-194.32
3 (1)	-850.35	-166.42
3 (2)	-841.09	-176.05
3 (3)	-832.84	-179.23
4 (1)	-819.63	-163.19
4 (2)	-809.76	-175.54

Table S104: Total electronic energies E_{tot} calculated with the CPCM of the protonated MeFo clusters, with a dielectric constant of 9.84. The clusters are labeled with their solvent number *n* and the isomer number in brackets (*i*). The energies were obtained from the DSD-BLYP/def2-TZVPP optimization in Hartree (1 H = 2625.4996 kJ mol^{-1}). The solvation energies of the clusters were obtained through $\Delta_{solv}G° = E_{tot}(CPCM) - E_{tot}(gas\text{-}phase)+7.96$ in kJ mol^{-1}.

n (*i*)	E_{tot}	$\Delta_{solv}G°$
MeFo	-228.9114503	-10.58
1 (1)	-229.3097193	-242.05
2 (1)	-458.2399490	-191.86
3 (1)	-687.1573527	-180.04
4 (1)	-916.0690768	-156.52

Table S105: Calculated BP/def2-TZVPP gas-phase energies in Hartree (1 H = 2625.4996 kJ mol^{-1}) of MeFo and the protonated MeFo clusters under 1 bar pressure, labeled with their solvent number *n* and the isomer number in brackets (*i*). The energies listed are the enthalpy *H*, the Gibbs energy *G*, the entropy (already multiplied with the temperature *T·S*) and the different contributions to the entropy (translational S_{trans}, vibrational S_{vib}, and rotational entropy S_{rot}) at 298.15 K, along with the total electronic energy E_{tot}. The rotational entropy was selected according to the symmetry number of the determined point group (pg).

n (*i*)	pg	E_{tot}	*H*	S_{trans}	S_{vib}	S_{rot}	*T·S*	*G*
MeFo	C_s	-229.176339	-229.110893	0.018149	0.003051	0.011233	0.032433	-229.143326
1 (1)	C_s	-229.486988	-229.408664	0.018173	0.003462	0.011310	0.032944	-229.441609
1 (2)	C_1	-229.483651	-229.405447	0.018173	0.003634	0.011143	0.032950	-229.438397
2 (1)	C_1	-458.711237	-458.567342	0.019143	0.014645	0.013964	0.047751	-458.615093
2 (2)	C_1	-458.709822	-458.566577	0.019143	0.015237	0.013946	0.048325	-458.614902
2 (3)	C_s	-458.708490	-458.566224	0.019143	0.015024	0.013893	0.048059	-458.614283
2 (4)	C_1	-458.708340	-458.565773	0.019143	0.015530	0.013770	0.048442	-458.614216
2 (5)	C_1	-458.707956	-458.564267	0.019143	0.014728	0.013948	0.047818	-458.612085
3 (1)	C_1	-687.912126	-687.700943	0.019713	0.027838	0.015384	0.062935	-687.763878
3 (2)	C_1	-687.908458	-687.696592	0.019713	0.028572	0.015474	0.063758	-687.760350
3 (3)	C_1	-687.907248	-687.694893	0.019713	0.027395	0.015206	0.062314	-687.757207
4 (1)	C_1	-917.097329	-916.818513	0.020118	0.041003	0.015868	0.076989	-916.895502
4 (2)	C_1	-917.092628	-916.812892	0.020118	0.042632	0.016102	0.078852	-916.891744

Table S106: Total electronic energies E_{tot} calculated with the CPCM of the protonated MeFo clusters, labeled with their solvent number n and the isomer number in brackets (i). The energies were obtained from the BP/def2-TZVPP optimization in Hartree (1 H = 2625.4996 kJ mol^{-1}). Only the isomers labeled with (1) were used for further calculations of the standard solvation energies of the proton through the thermodynamic cycles. The solvation energies of the clusters were obtained through $\Delta_{solv}G° = E_{tot}(CPCM) – E_{tot}(gas\text{-}phase)+7.96$.

n (i)	E_{tot}
MeFo (1)	-229.182976
MeFo (2)	-229.179520
1 (1)	-229.580257
1 (2)	-229.578476
2 (1)	-458.785002
2 (2)	-458.783277
2 (3)	-458.783259
2 (4)	-458.782619
2 (5)	-458.782619
3 (1)	-687.978545
3 (2)	-687.974953
3 (3)	-687.973537
4 (1)	-917.162518
4 (2)[a]	-917.157411

[a] structures with imaginary modes

3.4 Gibbs Energies of Solvation of the Proton

Table S107: Boltzmann weighted (wt., at the bottom) and unweighted energies of the proton solvation $\Delta_{solv}G°(H^+)$ in water under standard conditions (1 bar gas to 1 mol L^{-1} solution), labeled with their solvent number *n* and the isomer number in brackets (*i*). The solvation energies were obtained from the monomer (mono) cycle, with and without the energy of evaporation $\Delta_{vap}G°$ (vap) as well as the cluster cycle. All energies required to close these thermodynamic cycles are provided in Appendix 3.3.1. The energies were calculated with BP/def2-TZVPP, DSD-BLYP/def2-TZVPP and DLPNO-CCSD(T)/CBS and are given in kJ mol^{-1}.

n (*i*)	BP/def2-TZVPP		DSD-BLYP/def2-TZVPP			DLPNO-CCSD(T)/CBS		
	mono vap	mono	mono vap	mono	cluster	mono vap	mono	cluster
1	-1027.2	-1018.5	-1025.6	-1015.9	-1015.9	-1019.2	-1010.2	-1010.2
2	-1093.0	-1075.4	-1082.8	-1063.5	-1060.1	-1067.1	-1049.1	-1048.0
3	-1124.9	-1098.5	-1110.7	-1081.8	-1092.5	-1090.3	-1063.2	-1081.4
4	-1145.2	-1110.0	-1130.5	-1091.8	-1098.1	-1105.6	-1069.5	-1088.2
5 (1)	-1161.5	-1117.6	-1151.9	-1103.6	-1113.4	-1121.1	-1075.9	-1101.1
5 (2)	-1159.2	-1115.3	-1149.6	-1101.3	-1111.0	-1118.7	-1073.6	-1098.8
5 (3)	-1149.9	-1106.0	-1141.0	-1092.7	-1102.5	-1110.2	-1065.0	-1090.2
6 (1)	-1188.9	-1136.3	-1170.1	-1112.1	-1128.9	-1134.6	-1080.4	-1118.0
6 (2)	-1188.3	-1135.6	-1164.9	-1107.0	-1123.8	-1129.4	-1075.2	-1112.8
6 (3)	-1185.1	-1132.4	-1162.4	-1104.4	-1121.2	-1126.9	-1072.7	-1110.3
6 (4)	-1174.6	-1121.9	-1153.0	-1095.1	-1111.9	-1117.5	-1063.3	-1100.9
7 (1)	-1198.6	-1137.1	-1184.6	-1117.0	-1135.8	-1141.5	-1078.3	-1121.4
7 (2)	-1209.5	-1148.0	-1171.4	-1103.8	-1122.6	-1128.3	-1065.1	-1108.2
7 (3)	-1198.7	-1137.2	-1170.7	-1103.0	-1121.9	-1127.5	-1064.3	-1107.4
7 (4)	-1180.9	-1119.4	-1149.9	-1082.3	-1101.1	-1106.8	-1043.6	-1086.7
wt.								
2	-1093.0	-1075.4	-1082.8	-1063.5	-1060.1	-1067.1	-1049.1	-1048.0
3	-1124.9	-1098.5	-1110.7	-1081.8	-1092.5	-1090.3	-1063.3	-1081.4
4	-1145.2	-1110.1	-1130.5	-1091.9	-1098.3	-1105.6	-1069.7	-1088.4
5	-1162.4	-1118.5	-1152.8	-1104.5	-1114.2	-1121.9	-1076.9	-1102.0
6	-1190.6	-1138.0	-1170.5	-1112.6	-1129.3	-1135.0	-1081.2	-1118.3
7	-1209.5	-1148.1	-1184.6	-1117.4	-1136.0	-1141.7	-1081.9	-1122.0

Table S108: Boltzmann weighted (wt., at the bottom) and unweighted energies of the proton solvation $\Delta_{solv}G°(H^+)$ in MeOH under standard conditions (1 bar gas to 1 mol L^{-1} solution), labeled with their solvent number *n* and the isomer number in brackets (*i*). The solvation energies were obtained from the monomer (mono) cycle, with and without the energy of evaporation $\Delta_{vap}G°$ (vap) as well as the cluster cycle. All energies required to close these thermodynamic cycles are provided in Appendix A.3.2. The energies were calculated with BP/def2-TZVPP, DSD-BLYP/def2-TZVPP and DLPNO-CCSD(T)/CBS and are given in kJ mol^{-1}.

n (*i*)	BP/def2-TZVPP		DSD-BLYP/def2-TZVPP			DLPNO-CCSD(T)/CBS		
	mono vap	mono	mono vap	mono	cluster	mono vap	mono	cluster
1	-1030.71	-1025.31	-1032.96	-1026.54	-1026.54	-1027.49	-1020.81	-1020.81
2 (1)	-1086.46	-1075.67	-1082.04	-1069.20	-1072.30	-1069.45	-1056.09	-1062.33
2 (2)	-1086.46	-1075.67	-1081.94	-1069.10	-1072.20	-1069.35	-1055.99	-1062.23
2 (3)	-1084.77	-1073.98	-1080.62	-1067.78	-1070.88	-1068.03	-1054.67	-1060.91
3 (1)	-1109.42	-1093.23	-1102.19	-1082.93	-1099.69	-1082.02	-1061.97	-1087.00
3 (2)	-1110.12	-1093.94	-1100.15	-1080.89	-1097.64	-1079.97	-1059.93	-1084.96
4 (1)	-1123.92	-1102.34	-1113.10	-1087.42	-1100.18	-1087.63	-1060.91	-1087.25
4 (2)	-1123.37	-1101.78	-1112.49	-1086.81	-1099.57	-1087.02	-1060.30	-1086.64
5 (1)	-1127.04	-1100.07	-1112.32	-1080.22	-1093.20	-1088.23	-1054.83	-1085.02
5 (2)	-1126.45	-1099.47	-1112.96	-1080.86	-1093.85	-1088.88	-1055.48	-1085.66
6 (1)	-1144.53	-1112.15	-1123.52	-1085.00	-1103.82	-1093.38	-1053.30	-1094.87
6 (2)	-1143.04	-1110.67	-1123.29	-1084.77	-1103.59	-1093.15	-1053.06	-1094.64
6 (3)	-1151.03	-1118.66	-1129.88	-1091.37	-1110.19	-1099.74	-1059.66	-1101.23
wt								
2	-1088.74	-1077.95	-1084.33	-1071.49	-1074.60	-1071.75	-1058.39	-1064.62
3	-1111.51	-1095.33	-1103.09	-1083.85	-1100.59	-1082.94	-1063.25	-1087.91
4	-1125.38	-1103.87	-1114.56	-1089.16	-1102.87	-1089.27	-1064.56	-1090.04
5	-1129.11	-1104.68	-1116.19	-1089.31	-1102.98	-1091.55	-1064.67	-1090.70
6	-1151.30	-1118.93	-1130.23	-1092.51	-1110.64	-1100.16	-1065.02	-1101.60

Table S109: Boltzmann weighted (bottom) and unweighted energies of the proton solvation $\Delta_{solv}G°(H^+)$ in EtOH under standard conditions (1 bar gas to 1 mol L^{-1} solution), labeled by their solvent number *n* and the isomer number in brackets (*i*). The solvation energies were obtained from the monomer (mono) cycle, with and without the energy of evaporation $\Delta_{vap}G°$ (vap) as well as the cluster cycle. All energies required to close these thermodynamic cycles are provided in Appendix A.3.3. The energies were calculated with BP/def2-TZVPP, DSD-BLYP/def2-TZVPP and DLPNO-CCSD(T)/CBS and are given in kJ mol^{-1}.

n (*i*)	BP/def2-TZVPP		DSD-BLYP/def2-TZVPP			DLPNO-CCSD(T)/CBS		
	mono vap	mono	mono vap	mono	cluster	mono vap	mono	cluster
1 (1)	-1035.29	-1032.04	-1033.28	-1029.24	-1029.24	-1027.45	-1023.05	-1023.05
1 (2)	-1035.32	-1032.07	-1033.59	-1029.54	-1029.54	-1027.76	-1023.36	-1023.36
2 (1)	-1083.56	-1077.06	-1074.64	-1066.55	-1075.94	-1062.34	-1053.53	-1065.74
2 (2)	-1083.64	-1077.14	-1074.25	-1066.16	-1075.55	-1061.95	-1053.14	-1065.35
2 (3)	-1083.74	-1077.24	-1072.65	-1064.56	-1073.95	-1060.35	-1051.55	-1063.75
2 (4)	-1082.95	-1076.45	-1073.46	-1065.37	-1074.76	-1061.16	-1052.36	-1064.56
2 (5)	-1082.75	-1076.25	-1074.04	-1065.95	-1075.34	-1061.74	-1052.93	-1065.14
2 (6)	-1082.33	-1075.83	-1072.49	-1064.40	-1073.79	-1060.19	-1051.38	-1063.59
2 (7)	-1082.62	-1076.12	-1073.80	-1065.71	-1075.10	-1061.50	-1052.70	-1064.90
3 (1)	-1102.64	-1092.89	-1090.15	-1078.02	-1096.85	-1074.91	-1061.70	-1088.60
3 (2)	-1103.41	-1093.66	-1088.19	-1076.07	-1094.89	-1072.96	-1059.75	-1086.65
3 (3)	-1102.32	-1092.57	-1089.13	-1077.01	-1095.83	-1073.90	-1060.69	-1087.59
3 (4)	-1101.54	-1091.79	-1087.37	-1075.24	-1094.07	-1072.13	-1058.93	-1085.82
3 (5)	-1101.92	-1092.17	-1087.41	-1075.28	-1094.11	-1072.17	-1058.97	-1085.86
3 (6)	-1100.87	-1091.12	-1088.05	-1075.92	-1094.75	-1072.82	-1059.61	-1086.50
3 (7)	-1100.34	-1090.59	-1086.51	-1074.38	-1093.21	-1071.28	-1058.07	-1084.97
4 (1)	-1121.16	-1108.15	-1101.74	-1085.57	-1106.21	-1080.51	-1062.90	-1096.32
4 (2)	-1110.40	-1097.40	-1090.42	-1074.25	-1094.89	-1069.19	-1051.58	-1085.00
4 (3)	-1111.24	-1098.24	-1089.70	-1073.53	-1094.17	-1068.47	-1050.86	-1084.28
4 (4)	-1110.77	-1097.77	-1090.93	-1074.76	-1095.40	-1069.70	-1052.09	-1085.51
4 (5)	-1110.67	-1097.67	-1089.77	-1073.60	-1094.25	-1068.54	-1050.93	-1084.35
5 (1)	-1122.86	-1106.61	-1097.69	-1077.47	-1103.48	-1072.51	-1050.49	-1092.79
5 (2)	-1121.24	-1104.99	-1096.23	-1076.02	-1102.03	-1071.05	-1049.04	-1091.34
5 (3)			-1095.09	-1074.87	-1100.88	-1069.91	-1047.89	-1090.19
6 (1)			-1094.10	-1069.84	-1102.21	-1065.20	-1038.79	-1090.81
6 (2)			-1094.51	-1070.25	-1102.61	-1065.61	-1039.20	-1091.22
wt								
2	-1087.96	-1081.46	-1078.55	-1070.46	-1079.85	-1066.25	-1057.44	-1069.65
3	-1106.88	-1097.13	-1093.21	-1081.11	-1099.91	-1077.99	-1064.89	-1091.66
4	-1121.31	-1108.33	-1101.94	-1086.05	-1106.51	-1081.37	-1065.85	-1096.78
5	-1124.65	-1109.73	-1102.67	-1086.20	-1107.62	-1081.50	-1065.86	-1097.58
6			-1102.83	-1086.20	-1108.16	-1081.50	-1065.85	-1097.90

Table S110: Boltzmann weighted (wt., at the bottom) and unweighted energies of the proton solvation $\Delta_{solv}G°(H^+)$ in MeCN under standard conditions (1 bar gas to 1 mol L^{-1} solution), labeled with their solvent number *n* and the isomer number in brackets (*i*). The solvation energies were obtained from the monomer (mono) cycle, with and without the energy of evaporation $\Delta_{vap}G°$ (vap) as well as the cluster cycle. All energies required to close these thermodynamic cycles are provided in Appendix A.3.4. The energies were calculated with BP/def2-TZVPP, DSD-BLYP/def2-TZVPP and DLPNO-CCSD(T)/CBS and are given in kJ mol^{-1}.

n (*i*)	BP/def2-TZVPP		DSD-BLYP/def2-TZVPP			DLPNO-CCSD(T)/CBS		
	mono vap	mono	mono vap	mono	cluster	mono vap	mono	cluster
1	-1027.74	-1013.05	-1016.23	-1002.19	-1002.19	-1012.71	-998.96	-998.96
2	-1068.21	-1038.82	-1056.13	-1028.05	-1043.14	-1048.30	-1020.79	-1035.62
3 (1)	-1070.18	-1026.10	-1048.85	-1006.72	-1034.62	-1040.63	-999.36	-1027.44
3 (2)	-1071.09	-1027.00	-1050.50	-1008.38	-1036.28	-1042.29	-1001.02	-1029.10
4 (1)	-1066.97	-1008.20	-1055.50	-999.34	-1053.92	-1046.45	-991.44	-1046.66
4 (2)	-1071.00	-1012.22	-1048.44	-992.28	-1046.86	-1039.40	-984.38	-1039.61
wt								
2	-1068.21	-1038.82	-1056.13	-1028.05	-1043.14	-1048.30	-1020.79	-1035.62
3	-1072.82	-1038.86	-1056.49	-1028.05	-1043.37	-1048.61	-1020.79	-1035.88
4	-1073.94	-1038.86	-1057.82	-1028.05	-1054.09	-1049.52	-1020.79	-1046.83

Table S111: Boltzmann weighted (wt., at the bottom) and unweighted energies of the proton solvation $\Delta_{solv}G°(H^+)$ in DMF under standard conditions (1 bar gas to 1 mol L^{-1} solution), labeled with their solvent number *n* and the isomer number in brackets (*i*). The solvation energies were obtained from the monomer (mono) cycle, with and without the energy of evaporation $\Delta_{vap}G°$ (vap) as well as the cluster cycle. All energies required to close these thermodynamic cycles are provided in Appendix A.3.5. The energies were calculated with BP/def2-TZVPP, DSD-BLYP/def2-TZVPP and DLPNO-CCSD(T)/CBS and are given in kJ mol^{-1}.

n (*i*)	BP/def2-TZVPP		DSD-BLYP/def2-TZVPP			DLPNO-CCSD(T)/CBS		
	mono vap	mono	mono vap	mono	cluster	mono vap	mono	cluster
1 (1)	-1083.60	-1075.57	-1087.87	-1078.38	-1078.38	-1088.23	-1077.46	-1077.46
1 (2)	-1084.18	-1076.15	-1087.94	-1078.46	-1078.46	-1088.31	-1077.53	-1077.53
2 (1)	-1128.43	-1112.38	-1127.91	-1108.93	-1130.66	-1123.83	-1102.27	-1126.17
2 (2)	-1124.24	-1108.19	-1120.90	-1101.93	-1123.66	-1116.82	-1095.27	-1119.17
3 (1)	-1119.89	-1095.81	-1116.81	-1088.34	-1130.38	-1100.30	-1067.97	-1114.82
3 (2)	-1121.34	-1097.26	-1114.86	-1086.40	-1128.43	-1098.36	-1066.03	-1112.87
4 (1)	-1109.74	-1077.64	-1105.94	-1067.99	-1142.47	-1100.08	-1056.97	-1137.59
4 (2)	-1107.90	-1075.80	-1107.07	-1069.11	-1143.59	-1101.21	-1058.10	-1138.72
4 (3)	-1104.56	-1072.46	-1101.27	-1063.32	-1137.80	-1095.41	-1052.30	-1132.92
4 (4)	-1104.46	-1072.36	-1102.33	-1064.37	-1138.85			
wt								
2	-1128.85	-1112.80	-1128.05	-1109.07	-1130.80	-1123.97	-1102.42	-1126.31
3	-1129.03	-1112.81	-1128.09	-1109.07	-1132.79	-1123.97	-1102.42	-1126.35
4	-1129.03	-1112.81	-1128.09	-1109.07	-1145.17	-1123.97	-1102.42	-1140.09

Table S112: Boltzmann weighted (wt., at the bottom) and unweighted energies of the proton solvation $\Delta_{solv}G°(H^+)$ in DMSO under standard conditions (1 bar gas to 1 mol L^{-1} solution), labeled with their solvent number *n* and the isomer number in brackets (*i*). The solvation energies were obtained from the monomer (mono) cycle, with and without the energy of evaporation $\Delta_{vap}G°$ (vap) as well as the cluster cycle. All energies required to close these thermodynamic cycles are provided in Appendix A.3.6. The energies were calculated with BP/def2-TZVPP, DSD-BLYP/def2-TZVPP and DLPNO-CCSD(T)/CBS and are given in kJ mol^{-1}.

n (*i*)	BP/def2-TZVPP		DSD-BLYP/def2-TZVPP			DLPNO-CCSD(T)/CBS		
	mono vap	mono	mono vap	mono	cluster	mono vap	mono	cluster
1	-1093.55	-1084.07	-1098.51	-1086.09	-1086.09	-1090.42	-1076.67	-1076.67
2 (1)	-1141.83	-1122.88	-1141.88	-1117.05	-1137.42	-1127.73	-1100.23	-1123.82
2 (2)	-1135.86	-1116.91	-1135.28	-1110.44	-1130.81	-1121.12	-1093.62	-1117.21
3 (1)	-1129.43	-1100.99	-1130.69	-1093.44	-1136.72	-1115.07	-1073.83	-1123.29
3 (2)	-1133.68	-1105.25	-1131.26	-1094.01	-1137.29	-1115.64	-1074.40	-1123.86
3 (3)			-1125.73	-1088.48	-1131.76	-1110.11	-1068.87	-1118.33
4	-1123.81	-1085.90	-1111.73	-1062.06	-1130.87	-1092.91	-1037.91	-1118.67
wt								
2	-1142.05	-1123.09	-1142.05	-1117.22	-1137.59	-1127.89	-1100.39	-1123.99
3	-1142.15	-1123.09	-1142.11	-1117.22	-1140.04	-1127.93	-1100.39	-1126.54
4	-1142.15	-1123.09	-1142.11	-1117.22	-1140.10	-1127.93	-1100.39	-1126.65

Table S113: Boltzmann weighted (wt., at the bottom) and unweighted energies of the proton solvation $\Delta_{solv}G°(H^+)$ in PC under standard conditions (1 bar gas to 1 mol L^{-1} solution), labeled with their solvent number *n* and the isomer number in brackets (*i*). The solvation energies were obtained from the monomer (mono) cycle, with and without the energy of evaporation $\Delta_{vap}G°$ (vap) as well as the cluster cycle. All energies required to close these thermodynamic cycles are provided in Appendix A.3.7. The energies were calculated with BP/def2-TZVPP, DSD-BLYP/def2-TZVPP and DLPNO-CCSD(T)/CBS and are given in kJ mol^{-1}.

n (*i*)	BP/def2-TZVPP		DSD-BLYP/def2-TZVPP			DLPNO-CCSD(T)/CBS		
	mono vap	mono	mono vap	mono	cluster	mono vap	mono	cluster
1 (1)	-1025.07	-1018.06	-1025.17	-1015.87	-1015.87	-1021.27	-1011.56	-1011.56
1 (2)	-1025.13	-1018.11	-1024.96	-1015.66	-1015.66	-1021.06	-1011.34	-1011.34
2 (1)	-1058.01	-1043.98	-1049.63	-1031.03	-1054.97	-1041.77	-1022.34	-1047.97
2 (2)	-1058.08	-1044.06	-1049.41	-1030.81	-1054.75	-1041.54	-1022.12	-1047.75
2 (3)	-1058.00	-1043.98	-1048.20	-1029.60	-1053.55	-1040.34	-1020.91	-1046.54
3 (1)	-1038.46	-1017.43	-1040.74	-1012.85	-1057.46	-1031.95	-1002.80	-1051.07
3 (2)	-1041.20	-1020.17	-1042.76	-1014.87	-1059.48	-1033.96	-1004.82	-1053.08
4	-1010.31	-982.27	-1018.31	-981.12	-1053.78			
wt								
2	-1060.75	-1046.73	-1051.88	-1033.28	-1057.22	-1044.01	-1024.61	-1050.22
3	-1060.75	-1046.73	-1051.96	-1033.29	-1061.00	-1044.08	-1024.61	-1054.48
4	-1060.75	-1046.73	-1051.96	-1033.29	-1061.13			

Table S114: Boltzmann weighted (wt., at the bottom) and unweighted energies of the proton solvation $\Delta_{solv}G°(H^+)$ in MeFo under standard conditions (1 bar gas to 1 mol L^{-1} solution), labeled with their solvent number *n* and the isomer number in brackets (*i*). The solvation energies were obtained from the monomer (mono) cycle, with and without the energy of evaporation $\Delta_{vap}G°$ (vap) as well as the cluster cycle. All energies required to close these thermodynamic cycles are provided in Appendix A.3.8. The energies were calculated with BP/def2-TZVPP, DSD-BLYP/def2-TZVPP and DLPNO-CCSD(T)/CBS and are given in kJ mol^{-1}.

n (*i*)	BP/def2-TZVPP		DSD-BLYP/def2-TZVPP			DLPNO-CCSD(T)/CBS		
	mono vap	mono	mono vap	mono	cluster	mono vap	mono	cluster
1 (1)	-1000.05	-991.20	-999.51	-989.82	-989.82	-995.67	-985.72	-985.72
1 (2)	-1000.38	-991.53	-1000.21	-990.52	-990.52	-996.36	-986.42	-986.42
2 (1)	-1034.32	-1016.62	-1026.52	-1007.14	-1037.12	-1019.33	-999.43	-1030.12
2 (2)	-1037.53	-1019.83	-1029.89	-1010.51	-1040.49	-1022.70	-1002.81	-1033.49
2 (3)	-1039.40	-1021.70	-1028.61	-1009.23	-1039.22	-1021.42	-1001.53	-1032.22
2 (4)	-1039.62	-1021.92	-1027.26	-1007.88	-1037.87	-1020.07	-1000.18	-1030.87
2 (5)	-1035.04	-1017.33	-1026.87	-1007.49	-1037.47	-1019.68	-999.79	-1030.47
3 (1)	-1035.66	-1009.10	-1018.38	-989.31	-1042.34	-1010.20	-980.36	-1035.99
3 (2)	-1036.03	-1009.47	-1017.18	-988.11	-1041.15	-1009.00	-979.17	-1034.79
3 (3)	-1030.95	-1004.40	-1014.48	-985.41	-1038.45	-1006.30	-976.47	-1032.09
4 (1)	-1008.00	-972.59	-989.40	-950.63	-1027.24	-979.43	-939.65	-1019.70
4 (2)	-1010.48	-975.07	-990.65	-951.89	-1028.50	-980.69	-940.90	-1020.96
wt								
2	-1042.01	-1024.31	-1032.16	-1012.78	-1042.76	-1024.97	-1005.08	-1035.76
3	-1042.42	-1024.32	-1032.17	-1012.78	-1045.07	-1024.98	-1005.08	-1038.48
4	-1042.42	-1024.32	-1032.17	-1012.78	-1045.08	-1024.98	-1005.08	-1038.49
